# A systematic review using a multi-layered criteria framework for assessing the validity and reliability of velocity monitoring devices in resistance training

**DOI:** 10.1371/journal.pone.0324606

**Published:** 2025-09-08

**Authors:** Youssef J. Wannouch, Samuel R. Leahey, Rodrigo Ramírez-Campillo, Harry G. Banyard, Harry S. Dorrell, Steven Stern, Gordon J. Ross, Jason C. Laffer, Kelvin Y.H. Chua, Paul Comfort

**Affiliations:** 1 Precision Sport Science, Redwood City, California, United States of America; 2 Exercise and Rehabilitation Sciences Institute, Faculty of Rehabilitation Sciences, Universidad Andres Bello, Santiago, Chile; 3 Department of Health and Medical Sciences, Swinburne University of Technology, Hawthorn Campus, Melbourne, Australia; 4 School of Sport and Exercise Science, University of Lincoln, Lincoln, United Kingdom; 5 Centre for Data Analytics, Bond University, Gold Coast, Queensland, Australia; 6 School of Mathematics, The University of Edinburgh, Edinburgh, United Kingdom; 7 School of Health & Society, University of Salford, Salford, Greater Manchester, United Kingdom; 8 Strength and Power Research Group, Edith Cowan University, Joondalup, Western Australia, Australia; Aston University, UNITED KINGDOM OF GREAT BRITAIN AND NORTHERN IRELAND

## Abstract

**Background:**

Velocity-Based Training (VBT) is an emerging method in resistance training for objectively prescribing and monitoring training intensity and neuromuscular function. Given its growing popularity, assessing the validity and reliability of VBT devices is critical for strength and conditioning coaches.

**Objective:**

The primary purpose of this review was twofold: (1) to identify and address methodological gaps in current assessments of VBT device validity and reliability, and (2) to propose and apply a novel, multi-layered, criterion-based framework—developed in collaboration with statisticians and domain experts—for evaluating these devices.

**Methods:**

A systematic search was conducted in PubMed, Scopus, and SPORTDiscus following PRISMA guidelines, focusing on original research studies published before February 2024 that assessed VBT device validity or reliability. Out of 568 studies identified, 75 met the inclusion criteria.

**Results:**

Among the included studies, 66 investigated device validity and 56 examined reliability, with some studies addressing both aspects. Notably, only 5 of the 66 validity studies met all of the proposed criteria, while just 16 of the 56 reliability investigations satisfied the required statistical thresholds defined by our framework. These findings highlight significant methodological variability and underscore the need for more standardized evaluation practices.

**Conclusions:**

This review systematically evaluated the validity and reliability of various VBT devices and introduced a robust, multi-layered framework for their assessment. By integrating statistician-led and domain expert-led criteria, the framework offers a standardized approach that enhances the precision of device evaluation. Promising tools identified include the GymAware LPT, Perch Motion Capture Single Camera System, Flex optical device, and VmaxPro. Future research should build upon and refine this methodology to further standardize study designs, improve data reporting, and ultimately support more informed decision-making in sports technology and training practice.

## Introduction

### Velocity monitoring devices

Technological advancements over the past decade have fueled the surge in Velocity-Based Training (VBT), making sophisticated velocity-monitoring tools accessible to Strength and Conditioning (S&C) coaches across various settings. The efficacy of VBT is contingent upon the validity and reliability of velocity-monitoring devices [1,2]. Validity in this context refers to the device’s ability to accurately measure what it is intended to measure, often benchmarked against a “gold-standard” criterion from existing literature [1] in the case of barbell velocity for VBT the gold standard is three-dimensional motion capture. Reliability denotes the device’s ability to produce consistent results over repeated measures [1]. Researchers previously assessing the validity or reliability of technological devices have used different statistical approaches to determine acceptable validity and reliability, such as a high correlation (r > 0.70), low coefficient of variation (CV < 10%), and small effect size (ES < 0.60) for validity [1,3,4], and a high intra-class correlation (ICC ≥ 0.90), low CV (< 10%), and a standardized mean bias < 0.60 for reliability [4,5]. Both intra- and inter-device reliability are crucial for meaningful progress tracking, particularly when the same device is used consistently [1,6]. Furthermore, it is essential to differentiate between biological variations—like an athlete’s physical condition and readiness—and the technological inconsistencies of the device [4]. The current landscape of sport science literature reveals a lack of evidence-based standardized measures for assessing these parameters [7–15].

### Philosophical and empirical considerations of validity & reliability in sport science

While statistical validity and reliability serve as essential tools for empirically addressing epistemological questions of knowledge, the operationalization of these constructs has historically been somewhat arbitrary [16–20]. This lack of standardization is not unique to sport science; for example, the once-coveted “P-value” has come under scrutiny, leading to some academic journals to reject it altogether due to its alleged misuse and the growing awareness of its limitations in accurately reflecting the strength of evidence in scientific studies [21–23]. Others have opted to use P-values alongside other statistical measures such as effect sizes with confidence intervals to enhance the certainty of their inferences [24–27]. Similarly, theories of validity and reliability are not immune to critical examination [28–30]. In the context of this systematic review, validity should be benchmarked against a recognized ‘gold standard,’ providing a semblance of objectivity [1], such as three-dimensional motion capture for velocity-based metrics. However, subjectivity arises not in the gold standards, which serve as benchmarks for objective truth, but in the methods used to assess how closely these standards are approximated [28]. The thresholds for determining validity and reliability have varied over time, often based on arbitrary criteria [1,15,30,31]. Thus, traditionally used approaches to the assessment of validity and reliability, specifically in this context of technological devices, often lack a comprehensive framework that balances statistical rigor with domain expertise for improved context. Additionally, there is widespread practice of employing correlational coefficients as a primary tool for assessing validity [1,3,4]. While correlations can indicate the presence of an association between variables, or, in this context, between different measurement devices, this statistical relationship when used solely offers a superficial understanding at best [32]. This poses a fundamental limitation as a high correlation between devices may suggest a strong association, yet it provides no critical information on the aspects of accuracy and precision. For instance, two devices could consistently yield measurements that are highly correlated but systematically biased, wherein one device consistently overestimates or underestimates values compared to a gold standard [33]. Similarly, proportional bias, where the discrepancy between measurements varies across the range of values, remains unknown by the sole use of correlation measures and thus it is advisable to employ a direct measure of error for more accurate assessment. The sole use of effect sizes to assess validity is also inherently flawed in nature. Effect sizes can indicate how substantial the differences are between groups or conditions, providing a quantitative measure of the impact of an intervention or in this context the comparative performance of different devices [34]. However, neither of these approaches directly addresses the question of whether a device accurately measures what it intends to measure. Furthermore, the use of any criterion device not considered a gold standard in the assessment of validity simply investigates the level of agreement or association between those devices, and not true validity.

**Fig 1:**
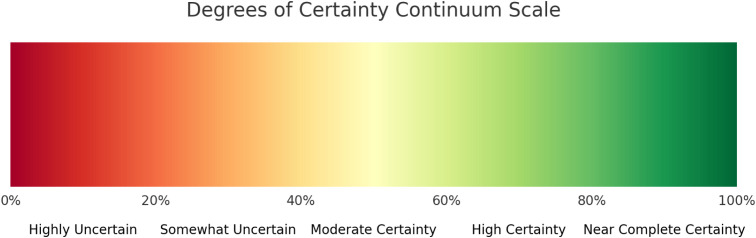
Degrees of certainty continuum scale.

It seems that in the landscape of sport science research, a methodological shortcut is too often adopt validity or reliability criteria from previous studies that have assessed tests or devices, without critical evaluation of their contextual appropriateness. This approach does not consider whether that specific criteria effectively increases practitioner confidence in making practical decisions. Essentially, the simple replication of broad statistical benchmarks for validity and reliability does not provide the necessary assurance that these tools or tests will perform as expected in new practical scenarios or environments. This methodological oversight highlights the need for a more thoughtful examination of how to determine and apply criteria for assessing the validity and reliability of research tools, ensuring that these measures are genuinely informative and applicable to the decisions practitioners must make. Kyprianou, Lolli [35] recently highlighted the importance of consulting domain experts in validity assessments to define practical equivalence margins when comparing devices or tests to gold standards. The authors of that study proposed this approach as a novel method for validity assessment. The authors of the present review concur with this perspective and acts on these suggestions, recognizing that strictly adhering to standardized statistical thresholds can pose a preventable limitation in practical assessments aimed at achieving an acceptable level of certainty [35]. To the author’s knowledge, this review proposes the first framework that uses multi-layered criteria and domain expert consultation to assess validity and reliability within this specific context. While there are existing frameworks that evaluate the methodological quality of studies [36], this proposed framework is unique in its specific focus on assessing validity and reliability at the methodological level (i.e., the use of a gold standard criterion or differentiation between technological and biological reliability), the statistical level (i.e., the statistician recommended testing measures) and domain-specific interpretation of said measures (i.e., the domain expert input on the relative statistical thresholds required for increased certainty). Due to its comprehensive coverage of multiple dimensions, the authors contend that the proposed framework enhances certainty (see Fig 1) compared to previously established approaches—such as those utilizing arbitrary statistical thresholds [1,3–5]—for assessing validity and reliability in this context. While the concept of certainty can be quantified using various objective measures (e.g., percentages, degrees, or levels), it ultimately exists on a continuum between absolute certainty and complete uncertainty. Although this framework does not claim to provide ultimate certainty, it represents an improvement over what is currently applied. Fig 1 helps visually demonstrate how different degrees of certainty might be situated along this continuum. Ultimately, although we do not assign an exact numerical increase in certainty before or after applying this framework, its enhanced methodological rigor offers a more robust assessment of validity and reliability. Thus, logically enabling practitioners to develop a greater sense of certainty in the evidence they apply.

The primary purpose of this review was twofold: first, to identify and address methodological gaps within the assessment of validity and reliability of velocity monitoring devices used in velocity-based training; second, to propose and transparently apply a multi-layered, criterion-based framework—developed in collaboration with statisticians and domain experts—for assessing RT variables measured via VBT technological devices. By systematically evaluating the latest research using this comprehensive framework, we aim to assist S&C coaches in identifying the most valid and reliable devices for their specific needs, thereby enabling more accurate and effective use of VBT in both practice and research.

## Methods

### Search strategy

A search strategy was implemented following PRISMA guidelines for systematic reviews [37]. The academic databases PubMed, Scopus, and SPORTDiscus were systematically searched in February 2024 to identify peer-reviewed original research studies on the validity and reliability of technological devices used to quantify velocity, displacement, and additional RT variables. These additional variables include metrics such as time spent at isokinetic velocity, time to reach isokinetic velocity, total work, exercise recognition, repetition count, 1RM prediction, and full waveform velocity, which are listed in Table S6 in S1 File. Furthermore, the original search was limited to studies published in English. To verify that this language restriction did not introduce bias, we applied the same search criteria to non-English studies within the same date range across all databases searched. This approach identified eight non-English studies (six in Chinese and two in Spanish), whose titles and abstracts were in English and subsequently evaluated for eligibility. No non-English studies met the inclusion criteria for the review, thereby ensuring that the language restriction did not bias the review’s results. The search was guided by the PICO strategy [38], and utilized pre-determined search terms, keywords, and Boolean operators (AND/OR). The search results were extracted and imported into a reference manager (EndNote 20.4.1, Clarivate Analytics, Philadelphia, PA, USA) and analyzed for relevance to the scope of this systematic review. The specific search strategies and predetermined search terms for each database are presented in the electronic supplementary material (Table S1 in S1 File)*.*

### Searching other resources

In addition to the electronic searches, the reference lists of the included full text articles were screened with publications that met the inclusion criteria included in the review.

### Study selection & data extraction

All articles were screened using pre-determined eligibility criteria, which included the requirement for the studies to be original research investigations, published prior to February 2024, and focused on the validity or reliability of VBT devices. The screening process was conducted independently by two reviewers (YW, RC) to minimize any potential biases, and any conflicts were resolved through discussion or by consulting a third reviewer (SL). The data extraction process was conducted by YW and SL using a systematic review software package, *(Covidence systematic review software, Veritas Health Innovation, Melbourne, Australia),* which eliminated duplicates and allowed for the extraction of relevant information from the included studies.

Additionally, this review intentionally excluded any studies or data that aimed to validate force or power-related metrics, even if they involved comparisons to a gold-standard force plate. This method of assessment has major flaws and limitations when contrasted with a VBT device. The fundamental difference lies in the approach to measurement, as force plates directly measure force and time, while VBT devices such as LPT’s measure displacement and time, with the latter permitting calculation of both mean and peak velocity of the barbell. This distinction is critical to understand as the velocity of the barbell has been shown to be substantially different from the velocity of the athletes center of mass and the system center of mass (center of mass of the combined mass of the athlete and barbell), during the back squat [39,40], jump squat [40] and power clean [40,41]. As such, it is not feasible to validate the power output from a VBT device to that calculated using force plates, as only the velocity of the system can be calculated from the resulting force-time data. Additionally, attempting to validate forces predicted from barbell velocity to forces directly measured on force plates are inherently flawed, as the velocity of the barbell, center of mass and system center of mass differ [39–42], with greater differences where the displacement of the barbell is substantially greater than the center of mass (e.g., power clean) [40,41]. As such the relative force required to accelerate the system center of mass and the barbell, given their different displacements and velocities, would differ substantially. It is therefore recommended that VBT devices should be used to determine displacement and velocity, but not force or power, and thus the decision was made to exclude such studies and data from this review.

### Quality assessment tool

To assess the quality of each study included in this review, a modified version of the Downs and Black checklist was used to better suit the nature of studies included [36] using Covidence software (Veritas Health Innovation, Melbourne, Australia). This specific instrument was deliberately selected over more conventional tools (e.g., Cochrane Risk of Bias Tool), which are primarily intended for randomized controlled trials. This choice was made due to the inherently context-specific nature of validity and reliability assessments, particularly given the diversity of devices, metrics, and observational designs encountered in this review. Traditional trial-based assessment tools typically prioritize sources of heterogeneity less relevant to our research aims. Conversely, the modified Downs and Black checklist permits context-sensitive and meaningful comparisons across diverse study designs, thereby facilitating a more precise evaluation of methodological quality within this domain [36]. This checklist was validated for reporting the quality of observational study designs and has been previously used in sport science systematic reviews [1,43]. However, not all checklist criteria were applicable to the studies included in this review. Nine of the 27 criteria were used to assess the studies included. The modified Downs and Black questions can be found in Table S2 in S1 File. The reporting quality was scored on a scale using 0 (unable to determine or no) or 1 (yes). A total score of 9 indicated the highest reporting quality with scores above 6 considered “good”, scores of 4–6 considered “moderate”, and scores below 4 considered “poor”.

### Technological efficacy – validity and reliability criteria

**Fig 2:**
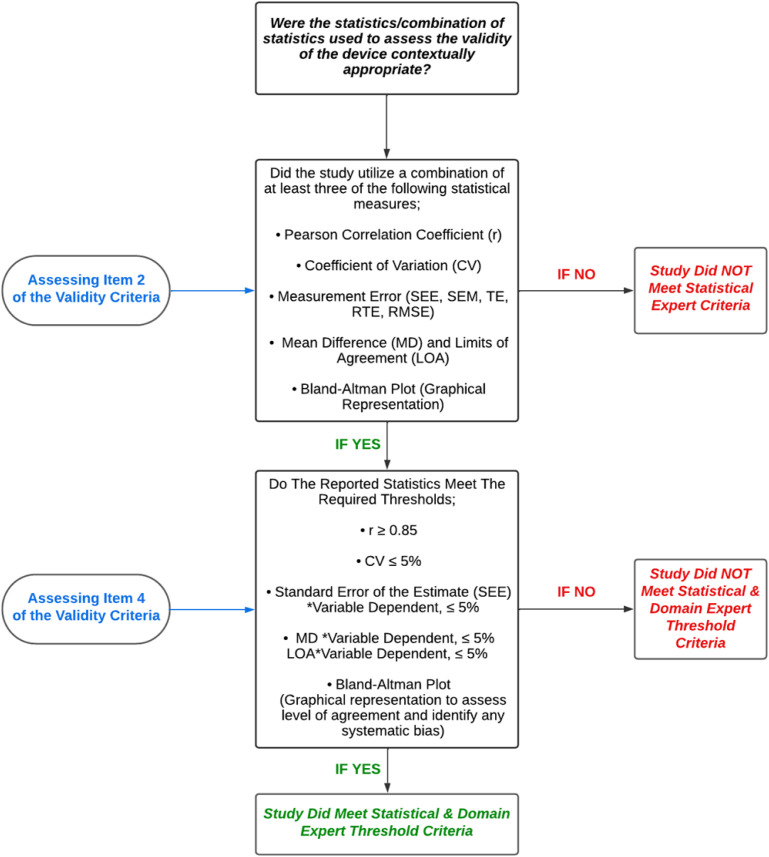
Validity statistics decision tree process for items 2 & 4 of the validity of velocity based training device criteria.

**Fig 3:**
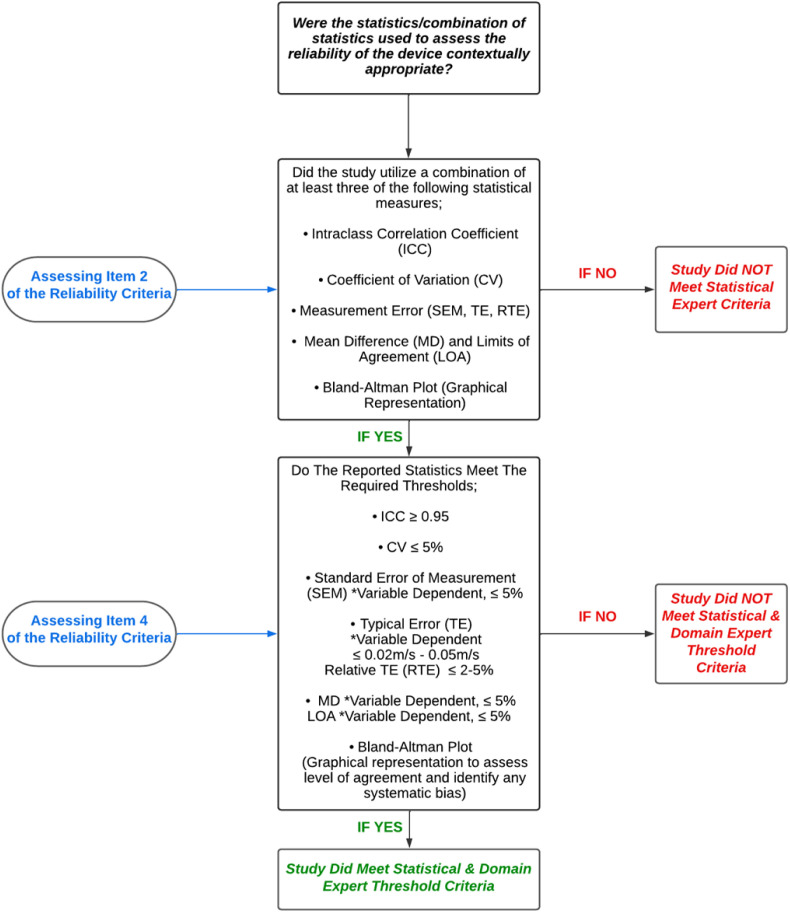
Reliability statistics decision tree process for items 2 & 4 of the reliability of velocity based training device criteria.

Following extensive discussions with domain experts—practitioners and researchers who have published extensively on the validity, reliability, and application methods of VBT devices—and statistical experts, the authors established stringent validity criteria (Table 1) and reliability criteria (Table 2) for assessing technological devices. Additionally, to ensure that the threshold values used to categorize device validity and reliability were both statistically robust and practically relevant, we individually consulted three domain experts. Each expert was asked to provide their expert perspective on the statistical values they deemed acceptable for establishing validity and reliability in VBT devices across different practical scenarios. Their individual responses were then collated, and through an iterative discussion process between the experts and the authors, a consensus was reached. This consensus informed the specific threshold values presented in Figs 2 and 3 and aimed to provide a more comprehensive and objective approach for evaluating the technological efficacy of VBT devices.

**Table 1:** Validity of velocity based training device criteria.

**1. Was a gold standard criterion used?**	YES/ NO
**2. Were the statistics/combination of statistics used to confirm the validity of the device contextually appropriate?**	YES/ NO
**3. Did the original study claim the device was valid?**	YES/ NO
**4. Does this device validly measure what was measured?**	YES/ NO

**Table 2:** Reliability of VBT device criteria.

1. *Was both biological and technological reliability reported?	YES/ NO
**2. Were the statistics/combination of statistics used to confirm the reliability of the device contextually appropriate?**	YES/ NO
**3. Did the original study claim the device was reliable?**	YES/ NO
**4. Does this device reliably measure what was measured?**	YES/ NO

Notes: *Additional bonus criteria.

Table 1 outlines the criteria for studies focused on validity. This 4-item checklist includes:

1. Verification of the use of a gold standard criterion, such as a multi-camera 3D motion capture system.
2. Evaluation of the appropriateness of the statistical methods used for assessing device validity, based on a multi-layered decision-making process that incorporates both statistical measures and domain expert validation (Fig 2). Selecting at least three statistical measures (such as measurement error, coefficient of variation, and mean difference) is a required criterion to advance to the next item on the checklist. This approach ensures greater contextual certainty regarding the true nature of statistical validity given its complexity.
3. Examination of the original study’s interpretation of statistical outputs to determine whether it claimed the device was valid.
4. A final assessment to confirm that the device and study meet the required thresholds set by the domain experts outlined in Fig 2, thereby validating the device’s intended measurements.

Studies meeting the criteria for items 1, 2, and 4 are deemed to have met the validity criteria for this review. While item 3 does not directly impact the final assessment, it is included to acknowledge the complexities and variations in interpreting statistical data, as well as the diverse perspectives that different researchers and practitioners may bring to this interpretation. The statisticians consulted recommended selecting at least three different statistical measures (Fig 2) to account for the various dimensions of validity when compared to a gold standard [44]. These dimensions include: ^1^Measurement Concordance to a gold standard—can be assessed using metrics such as the Pearson correlation coefficient and coefficient of variation, which evaluate the relative strength and consistency of the relationship between the device’s measurements and the gold standard criterion [17,28,44]. ^2^Accuracy of the measures to the gold standard—can be assessed using metrics such as standard error of the estimate, typical error, and root mean square error, which respectively quantify the predictive accuracy, degree of absolute error, or deviation of the device’s measurements from the true values provided by the gold standard [17,28,44]. ^3^Assessment of Bias—can be assessed using metrics such as limits of agreement, mean difference, and graphically represented via Bland-Altman plots, which identify and quantify any systematic bias or differences between the device’s measurements and the gold standard [17,28,44]. Thus, by selecting a minimum of three statistical measures from the list provided in Fig 2, at least two dimensions of validity will be covered. This approach ensures sufficient certainty in making inferences about the validity of the device measurements.

Table 2 outlines the criteria for studies assessing reliability, also organized as a 4-item checklist:

1. Assessment of the study’s ability to differentiate between technological and biological reliability. This is considered a bonus criterion due to the inherent challenges in isolating biological variation from reliability values. However, its inclusion is crucial as such variations can potentially inflate error rates.
2. Evaluation of the statistical methods used for assessing device reliability, with specific requirements outlined in Fig 3. The selection of at least three statistical measures (i.e., such as intraclass correlation coefficient, coefficient of variation, and limits of agreement) as indicated in Fig 3, is a required criterion to advance to the next item on the checklist. This approach ensures greater contextual clarity regarding the reliability of the devices.
3. Examination of the original study’s interpretation of statistical outputs to determine whether it claimed the device was reliable.
4. A final assessment to confirm that the device and study meet the required thresholds set by the domain experts for reliability.

Given the resource-intensive nature of investigating true technological reliability—often requiring a specially calibrated rig programmed to travel at predefined velocities—many studies may lack the necessary resources or funding to undertake this task. Items 2, 3, and 4 follow the same principles as those outlined for the validity criteria. Similar to validity, the justification behind the criteria to select no less than three different statistical measures outlined in Fig 3 was recommended by statisticians to account for the different dimensions of measurement reliability when assessing intra- or inter-device reliability [44]. These dimensions include: ^1^Consistency of Measures—can be assessed using metrics such as the intraclass correlation coefficient and coefficient of variation, which evaluate the relative strength and consistency of the relationship between repeated measurements [17,28,44]. ^2^Accuracy of Repeated Measures—can be assessed using metrics such as typical error and relative typical error, which quantify the degree of absolute error or deviation in repeated measurements [17,28,44]. ^3^Assessment of Bias or Systematic Variability—can be assessed using metrics such as mean difference, limits of agreement, and Bland-Altman plots, which identify and quantify any systematic bias or variability within the measurements [17,28,44]. Therefore, by choosing at least three statistical measures from the list provided in Fig 3, at least two dimensions of reliability will be covered. This approach will provide adequate assurance when drawing conclusions on the reliability of the measurements.

## Results

### Identification of studies

**Fig 4:**
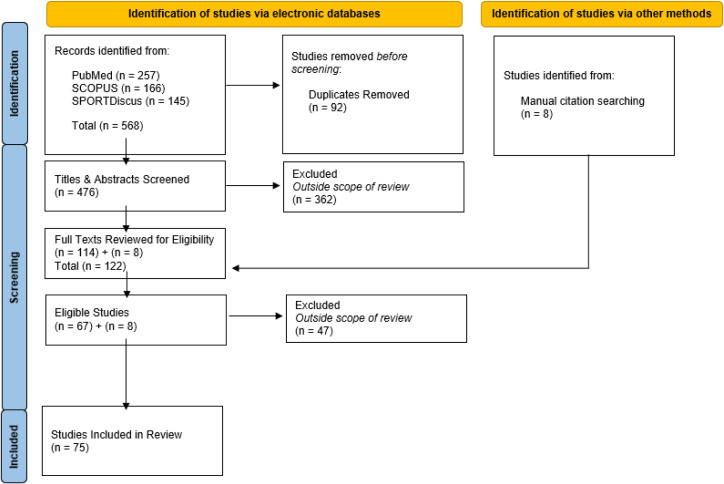
PRISMA flow diagram of study selection process for eligible studies included in review.

The systematic search retrieved 568 studies (Fig 4). After removing duplicates, the titles, and abstracts of the remaining 476 studies were screened for eligibility with 67 studies included from the original search and an additional 8 studies included from the references list of the included studies, totaling 75 studies. Table 3 provides a summary of technological efficacy studies identified in this review.

**Table 3:** Summary of technological efficacy studies identified in this review.

TYPE	DEVICE NAME	VALIDITY	RELIABILITY
Linear Position Transducer	GymAware	Appleby, Banyard [45], Askow, Stone [49], Banyard, Nosaka [3], Dorrell, Moore [50], Fritschi, Seiler [51], Janicijevic, García-Ramos [52], Lorenzetti, Lamparter [10], Menrad and Edelmann-Nusser [53], Mitter, Hölbling [54], Thompson, Rogerson [55],	Askow, Stone [49], Beckham, Layne [56], Dorrell, Moore [50], Appleby, Banyard [45], Janicijevic, García-Ramos [52] Lorenzetti, Lamparter [10], Orange, Metcalfe [5], Thompson, Rogerson [55], Jovanovic and Jukic [57], Oleksy, Kuchciak [58], Suchomel, Techmanski [59]
	Rep One	N/A	N/A
	Tendo	Garnacho-Castaño, López-Lastra [60], Lorenzetti, Lamparter [10] McGrath, Flanagan [61], Chéry and Ruf [62], Goldsmith, Trepeck [63], Suchomel, Techmanski [59]	Stock, Beck [64], Garnacho-Castaño, López-Lastra [60], Lorenzetti, Lamparter [10], Martinopoulou, Tsoukos [65], Suchomel, Techmanski [59]
	Vitruve	N/A	N/A
	T-Force	Lorenzetti, Lamparter [10], Pérez-Castilla, Piepoli [66], Janicijevic, García-Ramos [52]	Lorenzetti, Lamparter [10], García-Ramos, Pérez-Castilla [67], Pérez-Castilla, Piepoli [66], Courel-Ibáñez, Martínez-Cava [68], García-Pinillos, Latorre-Román [69], Martínez-Cava, Hernández-Belmonte [70], Muniz-Pardos, Lozano-Berges [71], Peña García-Orea, Belando-Pedreño [72], Janicijevic, García-Ramos [52], Pérez-Castilla, Miras-Moreno [73], Feuerbacher, Jacobs [74], Gomez-Piriz, Sanchez [75], Lopez-Torres, Fernandez-Elias [76]
	SmartCoach	Bardella, Carrasquilla García [77]*	Balsalobre-Fernández, Marchante [78]
	1080Q	Boehringer and Whyte [79], Fritschi, Seiler [51]	Boehringer and Whyte [79]
	FitroDyne (fitronic)	Fernandes, Lamb [80], Mitter, Hölbling [54]	Fernandes, Lamb [80]
	Open Barbell System	Goldsmith, Trepeck [63], Gonzalez, Mangine [81]	N/A
	Musclelab (Ergotest)	N/A	Van Den Tillaar and Ball [82]
	ChronoJump	Pérez-Castilla, Piepoli [66] Courel-Ibáñez, Martínez-Cava [68]	Pérez-Castilla, Piepoli [66] Courel-Ibáñez, Martínez-Cava [68]
	Speed4Lift	Callaghan, Guy [83], Pérez-Castilla, Piepoli [66], Martínez-Cava, Hernández-Belmonte [70]	Callaghan, Guy [83], Pérez-Castilla, Piepoli [66], Martínez-Cava, Hernández-Belmonte [70], Held, Rappelt [84], Lopez-Torres, Fernandez-Elias [76]
	Functional electro mechanical dynamometer (FEMD)	Rodriguez-Perea, Jerez-Mayorga [85]	Rodriguez-Perea, Jerez-Mayorga [85]
	Full-waveform resistance training monitoring system (FRTMS)	Lu, Zhang [46]	N/A
	Jueying (Beijing, China)	Qu, Qian [86]	Qu, Qian [86]
	ADR Encoder	Lopez-Torres, Fernandez-Elias [76], Moreno-Villanueva, Rico-González [87], Pérez-Castilla, Miras-Moreno [73]	Lopez-Torres, Fernandez-Elias [76], Moreno-Villanueva, Rico-González [87]
IMU/ Accelerometer	Push Band	Sato, K. Beckham [88], Balsalobre-Fernández, Kuzdub [89], McGrath, Flanagan [61], Chéry and Ruf [62], Orange, Metcalfe [11], Banyard, Nosaka [3], Pérez-Castilla, Piepoli [66], Courel-Ibáñez, Martínez-Cava [68], Van Den Tillaar and Ball [82], Thompson, Rogerson [55], Mitter, Hölbling [54], Fritschi, Seiler [51]	Balsalobre-Fernández, Kuzdub [89], Orange, Metcalfe [11], Pérez-Castilla, Piepoli [66] Courel-Ibáñez, Martínez-Cava [68], Van Den Tillaar and Ball [82], Thompson, Rogerson [55]
	Push Band 2.0	Callaghan, Guy [83], Gilic, Gabrilo [90], Jovanovic and Jukic [57], Lake, Augustus [91], Orser, Agar-Newman [92], Menrad and Edelmann-Nusser [53], Suchomel, Techmanski [59]	Callaghan, Guy [83], Gilic, Gabrilo [90], Jovanovic and Jukic [57], Lake, Augustus [91], Suchomel, Techmanski [59]
	MyoTest	Gomez-Piriz, Sanchez [75], Lorenzetti, Lamparter [10]	Lorenzetti, Lamparter [10]
	VmaxPro	Dragutinovic, Jacobs [93], Feuerbacher, Jacobs [74], Fritschi, Seiler [51], Held, Rappelt [84], Menrad and Edelmann-Nusser [53], Olaya-Cuartero, Villalón-Gasch [48]	Dragutinovic, Jacobs [93], Feuerbacher, Jacobs [74], Held, Rappelt [84], Olaya-Cuartero, Villalón-Gasch [48]
	WIMU System	Muyor, Granero-Gil [15], García-Pinillos, Latorre-Román [69], Pino-Ortega, Bastida-Castillo [94]	Ferro, Floría [95], Muyor, Granero-Gil [15], García-Pinillos, Latorre-Román [69]
	RehaGait	Mateo [96]	Mateo [96]
	Beast Sensor	Balsalobre-Fernández, Marchante [78], Pérez-Castilla, Piepoli [66], Mitter, Hölbling [54], Thompson, Rogerson [55]	Pérez-Castilla, Piepoli [66], Balsalobre-Fernández, Marchante [78], Thompson, Rogerson [55]
	Bar Sensei	Beckham, Layne [56] Thompson, Rogerson [55], Abbott, Wagle [97]	Beckham, Layne [56], Thompson, Rogerson [55], Abbott, Wagle [97]
	Output Sports Unit	Merrigan and Martin [98]	Merrigan and Martin [98]
	Accelerometer mobile basic program (MBP) via Huawei G620S smartphone	Pelaez Barrajon and San Juan [99]	Pelaez Barrajon and San Juan [99]
	Apple Watch Sport (1st generation) via Phone 6s with iOS 11.4.1 installed. + StrengthControl App	Oberhofer, Erni [100]	
2D Motion Analysis	Powerlift	Balsalobre-Fernández, Marchante [78], Balsalobre-Fernández, Marchante [101], Pérez-Castilla, Piepoli [66], Courel-Ibáñez, Martínez-Cava [68], Martínez-Cava, Hernández-Belmonte [70], Thompson, Rogerson [55]	Balsalobre-Fernández, Marchante [78], Balsalobre-Fernández, Marchante [101], Pérez-Castilla, Piepoli [66], Courel-Ibáñez, Martínez-Cava [68], Martínez-Cava, Hernández-Belmonte [70], Thompson, Rogerson [55]
	MyLift	Cetin and Isik [102]	Cetin and Isik [102]
	My Jump Lab v 3.0 with iPhone 12 Pro running iOS 15.5 (previously My Lift)		Balsalobre-Fernández, Xu [103]
	iLoad v1.0	De Sá, Medeiros [104], Pérez-Castilla, Boullosa [105], Pérez-Castilla, Boullosa [106]	Pérez-Castilla, Boullosa [105]
	Iron Path (version 1.9) App Via iPhone 8	Kasovic, Martin [107]	
	Kinovea via Samsung S6	Sánchez-Pay, Courel-Ibáñez [108]	
	Kinovea via Xiaomi A1	Sánchez-Pay, Courel-Ibáñez [108]	
	Kinovea via Casio FH20	Sánchez-Pay, Courel-Ibáñez [108]	
	Kinovea via iPhone X	Sánchez-Pay, Courel-Ibáñez [108]	
	Kinovea via Smartphone (Redmi Note 8)	Jiménez-Olmedo, Penichet-Tomás [109]	Jiménez-Olmedo, Penichet-Tomás [109]
	Kinovea via Digital Camera	Sañudo, Rueda [110]	
	Novel video system via Pocophone F1	Pueo, Lopez [111]	Pueo, Lopez [111]
	Tracker 5.0.6 software via Casio Exilim Pro EX-F1	Martinopoulou, Tsoukos [65]	Martinopoulou, Tsoukos [65]
3D Motion Analysis/Advanced Camera Systems	Elite Form Training System (EFTS)		Tomasevicz, Hasenkamp [112]
	Perch	Weakley, Munteanu [47]	Weakley, Munteanu [47]
Optic Devices/ Laser	Flex	Weakley, Chalkley [4], Fritschi, Seiler [51]	Weakley, Chalkley [4]
	Velowin	García-Ramos, Pérez-Castilla [67], Laza-Cagigas, Goss-Sampson [113], Pérez-Castilla, Piepoli [66], Courel-Ibáñez, Martínez-Cava [68], Muniz-Pardos, Lozano-Berges [71], Peña García-Orea, Belando-Pedreño [114], Peña García-Orea, Belando-Pedreño [72]	García-Ramos, Pérez-Castilla [67], Laza-Cagigas, Goss-Sampson [113], Pérez-Castilla, Piepoli [66], Courel-Ibáñez, Martínez-Cava [68], Muniz-Pardos, Lozano-Berges [71], Peña García-Orea, Belando-Pedreño [114], Peña García-Orea, Belando-Pedreño [72]

### Reporting quality

The reporting quality of the included studies was generally high (mean ± standard deviation 7.93 ± 0.98) (Table 4). Of the 75 studies, 25 reported a perfect score of 9, while one study achieved the lowest score of 5. However, 11 studies failed to provide full details of the technological device in accordance with item 3 of the checklist, and 6 studies did not report actual/ relevant statistics according to item 10. The most common item not met was item 18, which relates to the use of contextually appropriate statistical analyses, and 46 studies did not meet these criteria.

**Table 4:** Methodological reporting quality of eligible studies used in systematic review.

Study	*Items assessed using modified Downs and Black checklist*									
	Reporting						Internal Validity			*Total*
	1	2	3	6	7	10	16	18	20	
Abbott, Wagle [97]	1	1	1	1	1	1	1	0	1	***8***
Appleby, Banyard [45]	1	1	1	1	1	1	1	1	1	***9***
Askow, Stone [49]	1	1	1	0	1	1	1	0	0	***6***
Balsalobre-Fernández, Kuzdub [89]	1	1	1	1	1	1	1	1	1	***9***
Balsalobre-Fernández, Marchante [78]	1	1	1	1	1	1	1	0	1	***8***
Balsalobre-Fernández, Marchante [101]	1	1	1	1	1	1	1	1	1	***9***
Balsalobre-Fernández, Xu [103]	1	1	1	1	1	1	1	1	1	***9***
Banyard, Nosaka [3]	1	1	1	1	1	0	1	1	1	***8***
Bardella, Carrasquilla García [77]	1	1	1	1	1	1	1	1	1	***9***
Beckham, Layne [56]	1	1	0	1	1	0	1	0	1	***6***
Boehringer and Whyte [79]	1	1	0	1	1	1	1	0	1	***7***
Callaghan, Guy [83]	1	1	1	1	1	1	1	1	1	***9***
Cetin and Isik [102]	1	1	1	1	1	1	1	0	0	***7***
Chéry and Ruf [62]	0	1	0	1	1	1	1	0	1	***6***
Courel-Ibáñez, Martínez-Cava [68]	1	1	1	1	0	1	1	1	1	***8***
De Sá, Medeiros [104]	1	1	1	1	1	1	1	0	1	***8***
Dorrell, Moore [50]	1	1	1	1	1	1	1	0	1	***8***
Dragutinovic, Jacobs [93]	1	1	1	1	1	1	1	1	1	***9***
Fernandes, Lamb [80]	1	1	1	1	1	1	1	0	1	***8***
Ferro, Floría [95]	1	1	1	1	1	1	1	1	1	***9***
Feuerbacher, Jacobs [74]	1	1	1	1	1	1	1	1	1	***9***
Fritschi, Seiler [51]	1	1	1	1	1	1	1	0	1	***8***
García-Pinillos, Latorre-Román [69]	1	1	1	1	1	1	1	0	1	***8***
García-Ramos, Pérez-Castilla [67]	1	1	1	1	1	1	1	1	1	***9***
Garnacho-Castaño, López-Lastra [60]	1	1	0	1	1	1	1	0	1	***7***
Gilic, Gabrilo [90]	1	1	0	1	0	1	1	0	0	***5***
Goldsmith, Trepeck [63]	1	1	0	1	1	1	1	0	1	***7***
Gomez-Piriz, Sanchez [75]	1	1	1	1	0	1	1	0	1	***7***
Gonzalez, Mangine [81]	1	1	0	1	1	1	1	0	1	***7***
Held, Rappelt [84]	1	1	1	1	1	1	1	1	0	***8***
Janicijevic, García-Ramos [52]	1	1	1	1	1	1	1	0	1	***8***
Jiménez-Olmedo, Penichet-Tomás [109]	1	1	1	1	1	1	1	0	0	***7***
Jovanovic and Jukic [57]	1	1	1	1	1	1	1	1	1	***9***
Kasovic, Martin [107]	1	1	1	1	1	1	1	0	1	***8***
Lake, Augustus [91]	1	1	1	1	1	1	1	0	1	***8***
Laza-Cagigas, Goss-Sampson [113]	1	1	1	1	1	1	1	1	1	***9***
Lopez-Torres, Fernandez-Elias [76]	1	1	1	1	1	1	1	1	1	***9***
Lorenzetti, Lamparter [10]	1	1	1	1	0	0	1	0	1	***6***
Lu, Zhang [46]	1	1	1	1	1	1	1	1	1	***9***
Martínez-Cava, Hernández-Belmonte [70]	1	1	1	1	1	1	1	1	1	***9***
Martinopoulou, Tsoukos [65]	1	1	1	1	1	1	1	0	0	***7***
Mateo [96]	1	1	1	1	1	1	1	0	0	***7***
McGrath, Flanagan [61]	1	1	0	1	1	0	1	0	1	***6***
Menrad and Edelmann-Nusser [53]	1	1	1	1	1	1	1	0	1	***8***
Merrigan and Martin [98]	1	1	1	1	1	1	1	0	1	***8***
Mitter, Hölbling [54]	1	1	0	1	1	1	1	0	1	***7***
Moreno-Villanueva, Rico-González [87]	1	1	1	1	1	1	1	1	1	***9***
Muniz-Pardos, Lozano-Berges [71]	1	1	1	1	1	1	1	0	0	***7***
Muyor, Granero-Gil [15]	1	1	1	1	1	1	1	1	1	***9***
Olaya-Cuartero, Villalón-Gasch [48]	1	1	1	1	1	1	1	1	1	***9***
Oleksy, Kuchciak [58]	1	1	1	1	1	1	1	1	1	***9***
Orange, Metcalfe [11]	1	1	1	1	1	1	1	0	1	***8***
Orange, Metcalfe [5]	1	1	1	1	1	1	1	1	1	***9***
Orser, Agar-Newman [92]	1	1	1	1	1	1	1	1	1	***9***
Pelaez Barrajon and San Juan [99]	1	1	1	1	1	1	1	0	1	***8***
Pérez-Castilla, Piepoli [66]	1	1	1	1	1	1	1	0	1	***8***
Pérez-Castilla, Boullosa [105]	1	1	1	1	1	1	1	0	1	***8***
Pérez-Castilla, Boullosa [106]	1	1	1	1	1	1	1	0	0	***7***
Pérez-Castilla, Miras-Moreno [73]	1	1	1	1	1	1	1	0	1	***8***
Peña García-Orea, Belando-Pedreño [114]	1	1	1	1	1	0	1	0	1	***7***
Peña García-Orea, Belando-Pedreño [72]	1	1	1	1	1	0	1	0	1	***7***
Pino-Ortega, Bastida-Castillo [94]	1	1	1	1	1	1	1	0	1	***8***
Pueo, Lopez [111]	1	1	1	1	1	1	1	1	0	***8***
Qu, Qian [86]	1	1	1	1	1	1	1	1	1	***9***
Rodriguez-Perea, Jerez-Mayorga [85]	1	1	1	1	1	1	1	0	1	***8***
Sánchez-Pay, Courel-Ibáñez [108]	1	1	1	1	1	1	1	0	1	***8***
Sañudo, Rueda [110]	1	1	1	1	1	1	1	0	1	***8***
Sato, K. Beckham [88]	1	1	0	1	1	1	1	0	1	***7***
Stock, Beck [64]	1	1	0	1	0	1	1	0	1	***6***
Suchomel, Techmanski [59]	1	1	1	1	1	1	1	0	1	***8***
Thompson, Rogerson [55]	1	1	1	1	1	1	1	0	1	***8***
Tomasevicz, Hasenkamp [112]	1	1	1	1	1	1	1	1	1	***9***
Van Den Tillaar and Ball [82]	1	1	1	1	1	1	1	0	1	***8***
Weakley, Chalkley [4]	1	1	1	1	1	1	1	1	1	***9***
Weakley, Munteanu [47]	1	1	1	1	1	1	1	1	1	***9***

### Study characteristics

In this review, a total of 75 studies were included. Among these, 66 studies investigated the validity of a VBT device, and 56 studies assessed reliability. Notably, 47 studies examined both validity and reliability, while 19 studies focused exclusively on validity, and 9 studies addressed only reliability. Across all validity studies, a total of 40 different devices were investigated (Tables S7–S11 in S1 File), resulting in 105 validity investigations (Table S3 in S1 File). Of the 66 validity studies, only 24 used a gold standard criterion device to assess validity. After applying the framework proposed in this review, we found that only five studies [4,45–48] out of the 66 included met all the validity criteria (Fig 2), and thus, only these specific studies could classify the devices as valid based on our assessment. Additionally, 56 studies were identified that investigated the reliability of a VBT device, with a total of 33 different device types investigated (Tables S12–S16 in S1 File). The total number of reliability investigations per device type was 94 (Table S4 in S1 File). Of the 56 studies that investigated the reliability of a VBT device, only two studies [4,47] met the bonus criteria (Item 1) in the reliability assessment (Table 2) referring to the reporting of both biological and technological reliability. A total of 33 studies met Item 2 of the reliability criteria referring to the use of contextually appropriate statistics (Fig 3). Out of these 33 studies 16 were able to meet Item 4 of the criteria referring to meeting the required statistical thresholds (Fig 3), and therefore classified as reliable for their respective devices according to the assessment framework proposed in this review. Table 5 outlines these 16 studies, additionally Table 5 also details if that device had any supporting evidence for validity via other studies.

**Table 5:** Summary of the studies and their devices that were deemed reliable along with their validity status.

Study	Device/s	Reliability	Exercise/s	Sample Size	Intensity/ Load	Variable/s Measured	Supporting Evidence of Validity
Askow, Stone [49]	GymAware (LPT)	Intra-device	F/W Back Squat	9	75-90% 1RM	Mean Velocity	**YES** **for** **Vertical Barbell Displacement, Mean & Peak Force,** **Mean & Peak Power**
Courel-Ibáñez, Martínez-Cava [68]	T-Force (LPT) Chronojump (LPT) Velowin (Optic)	Intra-device, Inter-device	S/M Bench Press, S/M Back Squat, S/M Prone Bench Pull	25	20 kg, 30 kg, 40 kg, 50 kg, 60 kg, 70 kg, 80 kg	Mean Velocity, Mean Propulsive Velocity, Peak Velocity	**NO**
García-Ramos, Pérez-Castilla [67]	T-Force (LPT)	Intra-device	F/W Back Squat	31	20 kg, 30 kg, 40 kg, 50 kg, 60 kg, 70 kg	Mean Velocity, Mean Propulsive Velocity, Maximum Velocity	**NO**
Held, Rappelt [84]	Speed4Lift (LPT)	Within-day	F/W Back Squat, F/W Hip Thrust	19	75% 1RM	Mean Velocity, Barbell Displacement	**NO**
Jiménez-Olmedo, Penichet-Tomás [109]	Kinovea (v.0.9.1) via Smartphone (Redmi Note 8, Xiaomi, Beijing, China)	Intra-device	S/M Half ROM Back Squat	15	<40% 1RM, 40 to 70% 1RM, >70% 1RM, (20 kg and 50 kg)	Mean Velocity, Maximum Velocity	**NO**
Laza-Cagigas, Goss-Sampson [113]	Velowin (Optic)	Intra-device	F/W Back Squat	23	<30–90% 1RM	Barbell Displacement, Mean Velocity, Peak Velocity, Mean Force, Peak Force, Mean Power, Peak Power	**NO**
Martínez-Cava, Hernández-Belmonte [70]	T-Force (LPT) Speed4Lift (LPT)	Inter-device	S/M Back Squat, S/M Bench Press	15	25-95 kg	Peak Velocity, Mean Propulsive Velocity, Mean Velocity	**NO**
Muyor, Granero-Gil [15]	Tendo (LPT) WIMU System (IMU)	Intra-device	S/M Back Squat	23	40%, 80% 1RM	Mean Velocity, Eccentric Mean Velocity	**NO**
Moreno-Villanueva, Rico-González [87]	ADR Encoder	Inter-device	S/M Bench Press	11	5% to 100% 1RM	Mean Propulsive Velocity	**NO**
Olaya-Cuartero, Villalón-Gasch [48]	VmaxPro (IMU/Accelerometer)	Within-day	F/W Back Squat	20	75%, 85%, 90%, 95% 1RM	Mean Velocity, Displacement	**YES**
Peña García-Orea, Belando-Pedreño [114]	Velowin (Optic)	Intra-device	S/M Back Squat	26	20 kg, 30 kg, 40 kg, 50 kg, 60 kg, 70 kg	Mean Velocity, Mean Propulsive Velocity, Peak Velocity	**NO**
Peña García-Orea, Belando-Pedreño [72]	Velowin (Optic)	Intra-device	Loaded CMJ	21	3.5-43.5 kg	Mean Velocity, Peak Velocity	**NO**
Pueo, Lopez [111]	Novel Video System via Pocophone F1, Xiaomi, Pekin, China)	Intra-device	S/M Back Squat	20	75%, 85%, 90%, 95% 1RM	Mean Velocity, Mean Force, Mean Power, Range	**NO**
Suchomel, Techmanski [59]	GymAware (LPT)	Test-retest	Barbell Jump Shrug, Barbell Hang High Pull	15	40%, 60%, 80%, 100% 1RM	Mean Velocity, Peak Velocity	**NO**
Weakley, Munteanu [47]	Perch (3D Motion Capture)	Intra-device (Technological & Biological Variability)	F/W Back Squat, F/W Bench Press	16	20%, 40%, 60%, 80%, 90–100% 1RM and	Mean Velocity, Peak Velocity	**YES**
Weakley, Chalkley [4]	Flex (Optic)	Intra-device, Inter-device, (Technological and Biological error)	F/W Back Squat, F/W Bench Press, Calibrated Rig	18	0.53 ± 0.27 m/s 0.99 ± 0.00 m/s 0.84 ± 0.00 m/s 0.78 ± 0.00 m/s 0.71 ± 0.00 m/s 0.60 ± 0.00 m/s 0.54 ± 0.00 m/s 0.47 ± 0.00 m/s 0.38 ± 0.00 m/s 0.28 ± 0.00 m/s 0.17 ± 0.00 m/s 0.09 ± 0.00 m/s	Mean Velocity	**YES**

*Abbreviations: S/M: Smith Machine, F/W: Free Weight, kg: Kilograms, LPT: Linear Position Transducer, IMU: Inertial Measurement Unit, m/s: Meters Per Second, 1RM: One Repetition Maximum, CMJ: Counter Movement Jump.*

Across the included studies, a total of 358 measures were recorded. The most commonly used VBT exercises were the F/W back squat (n = 94, 26.3%), S/M bench press (n = 57, 15.9%), S/M back squat (n = 36, 10.1%), and F/W bench press respectively (n = 44, 12.3%) (Table S5 in S1 File). The variables of mean velocity (n = 191, 53.3%) and peak velocity (n = 96, 26.8%) were the most frequently investigated in studies (Table S6 in S1 File). The relative loads were expressed as a percentage of 1RM measured by direct assessment, while absolute loads were expressed in kilograms, velocities were expressed in meters per second, and displacement was measured both in meters and centimeters.

## Discussion

This review highlights the considerable diversity among the included studies. First, we evaluated a wide spectrum of VBT devices—with 40 distinct devices assessed in validity studies and 33 in reliability studies, which demonstrated the rapid evolution of technology in this field. Second, the studies involved a varied array of exercise modalities, with common examples including the free-weight back squat and the Smith machine bench press. Finally, although a range of outcome metrics were employed, mean and peak velocity emerged as the most frequently measured parameters. This synthesis reveals the heterogenic nature of the evidence and emphasizes the importance of our comprehensive, multi-layered assessment framework.

Of the 66 studies investigating the validity of a VBT device, 24 used a gold standard criterion device to assess criterion validity. Only five studies [4,45–48] met the validity criteria proposed in this review. Specifically, Appleby, Banyard [45] assessed the validity of the GymAware LPT device to measure vertical barbell displacement. Weakley, Munteanu [47] assessed the validity of the Perch 3D motion camera device, and Weakley, Chalkley [4] assessed the validity of the Flex optical device. More recently, Lu, Zhang [46] assessed the validity of a novel full-waveform resistance training monitoring device (FWRTD) that is based on a linear position transducer. Olaya-Cuartero, Villalón-Gasch [48] was the only study identified in this review to successfully validate an IMU/Accelerometer device, the VmaxPro, according to our proposed criteria. These five studies were the only ones that were able to meet all the statistical and domain expert validity assessment guidelines (Table 1 and Fig 2). Among the remaining 19 studies that used a gold standard device, 15 failed to use the recommended statistics by the statistical experts, while four studies [74,83,92,93] used the appropriate statistics but failed to meet the validity thresholds set by the VBT domain experts (Fig 2).

As only five out of 66 studies met the validity criteria proposed in this systematic review, the quality of validity assessments in sport science studies needs more attention. It is crucial to acknowledge that validity assessments can be subjective and influenced by individual biases and perspectives [115]. Therefore, to ensure the assessments are universal and consistent, they should be carried out to the highest possible standard. When validity assessments are not robust, heterogeneity in interpretation can lead to inconsistent findings and make it challenging to draw clear conclusions from research [116]. More broadly, this can even result in ineffective interventions or treatments and impede progress in understanding athletic performance mechanisms. To address this issue, clear guidelines and standards should be established for validity assessments across sport science. Greater statistical transparency could be achieved by applying a range of contextually appropriate statistics and presenting the full set of results [117]. These results can then be accompanied with specific recommendations for their interpretation. This could potentially help improve the overall quality of sport science research and enhance our contextual understanding of the statistical data presented in studies.

In summary, this assessment of technological validity studies found that the GymAware LPT device can be a valid tool to measure vertical barbell displacement for the F/W back squat across a load range of 70–90% 1RM. The GymAware LPT device was investigated 10 times for validity, the limitation of this specific device’s reported utility within this review is a methodological one, as three of the ten investigations lacked a comparison to a gold standard device and thus automatically failed the first layer of the proposed criteria, as well as five studies failing to meet the statistical standards recommended, leaving one study employing appropriate statistics but failing to meet the proposed thresholds, while the study by Appleby, Banyard [45] met all the proposed criteria. Future studies with appropriate methodological quality should be conducted to provide greater certainty on the broader validity of the GymAware device. The Perch motion capture single camera system was shown to be a valid tool for measuring mean and peak velocity of the F/W back squat and F/W bench press across all loads ranging from 20–100% 1RM. The Flex optical device was also shown to be a valid tool for measuring mean velocity for the F/W back squat and F/W bench press across all loads ranging from 20–90% 1RM. The VmaxPro IMU/accelerometer device can be a valid tool to measure mean velocity and displacement for the F/W back squat at 75–95% 1RM. Additionally, the novel Full-Waveform Resistance Training Monitoring System (FRTMS) was valid for the S/M back squat across loads ranging from 30–90% 1RM for mean velocity, eccentric mean velocity, and the full waveform velocity metric proposed in the study [46].

To determine the true reliability of a technological device, it is critical that we are able to determine the intra-device and inter-device technological variability without the influence of biological variability from human involvement [47]. Intra-device reliability informs S&C coaches on how consistent a device is in measuring the same parameter over multiple times. Inter-device reliability is important because it informs S&C coaches how consistent different devices of the same model are in measuring the same parameter, for example a multiple device setup in team settings. Thus, to determine an accurate assessment of technological reliability both intra and inter-device technological variation needs to be assessed on a calibrated mechanical rig with pre-determined speeds. As this type of setup and study design can be expensive to resource, it is often not investigated or discussed. Researchers aiming to investigate the true reliability of a technological device should be aware of this limitation and aim to mitigate the amount of biological variability and influence through highly stable testing conditions.

In this review, out of the 56 reliability investigations identified, only two [4,47] met the bonus criteria concerning the differentiation between biological and technological reliability. This highlights the inherent challenges in isolating biological factors when assessing technological reliability, an issue that warrants further attention in future research. A total of 31 studies met our criteria for the application of contextually appropriate statistical methods, and 16 of these also met the required statistical thresholds for reliability (Table 5). This underscores the rigor of our multi-layered criterion and its utility in identifying VBT devices that not only meet but exceed arbitrarily standard reliability metrics. Given these complexities and challenges, device manufacturers have a pivotal role to play. They should be conducting highly controlled investigations aimed at reporting the technological error inherent in their devices. Such information is invaluable for S&C coaches in making informed decisions and determining meaningful changes during their VBT applications. Therefore, this review highlights the need for a multi-dimensional evaluation framework that considers both technological and biological factors when assessing the reliability of VBT devices. While we advocate for increased investment in rigorous research methods and recommend collaboration between academic researchers and device manufacturers to generate reliable data that can guide S&C coaches, we also recognize the practical challenges that may limit such partnerships. The fast-paced nature of the industry, bureaucratic hurdles like paperwork and institutional review board processes, and resource constraints—including time, budget, and product launch timelines—can impede collaboration. Despite these feasibility concerns, exploring innovative strategies to overcome these barriers is essential for fostering effective academia-industry partnerships.

The multi-layered criterion proposed in this review is a methodological and philosophical enhancement for evaluating the validity and reliability of velocity-monitoring devices in VBT. Developed through a consultative process involving statisticians and domain experts, this criterion framework offers a more critical approach to technological assessment. Statisticians provided a robust set of statistical methods for assessing validity and reliability, while domain experts contextualized these methods by setting specific thresholds based on their experience and expertise. This dual consultation ensures that the framework is both statistically rigorous and practically relevant, addressing the often-arbitrary nature of statistical thresholds in validity and reliability assessments. Designed for adaptability, the criterion-based framework is open to further refinement through ongoing consultation with experts, aligning it with the scientific principle of falsifiability. It offers a more robust and accurate method for identifying the most valid and reliable devices, thereby providing an improvement over existing practices for informed decision-making by S&C coaches and sport scientists. While the criterion is tailored to the specific context of VBT devices, its multi-layered framework could be adapted and applied to other contexts, addressing the inherent complexities and subjectivities in scientific inquiry. It’s important to note that while our criterion improves upon existing methods, it is not a claim to ultimate certainty. This approach represents a significant step toward a more accurate and reliable method of assessment, serving as a comprehensive attempt to navigate the complexities of scientific inquiry.

Although the current framework does not explicitly address sample size, the authors acknowledge that sample size is crucial in reliability assessments [118]. However, in the context of the reliability studies reported in this review—focusing on within-subject reliability of VBT devices—smaller sample sizes are common due to practical constraints in sport science, including limited participant availability, time, and resources. Because within-subject reliability emphasizes repeated measures within the same individuals, a robust methodology can still yield meaningful data even with fewer participants. While Bland and Altman recommend a minimum of 50 participants to obtain precise population-level Limits of Agreement [118], this threshold is less applicable to repeated-measures reliability at the individual level. Similarly, prescribing a strict cutoff—such as the 62 participants required for an ICC ≥ 0.95 at α = 0.05 and 80% power [119]—could exclude studies that otherwise meet the proposed statistical and methodological standards. As shown in Table 5, the sample sizes for studies deemed to have adequate reliability evidence based on the applied framework ranged from n = 9 to n = 31, meaning that even the largest sample did not reach the minimum recommended thresholds for population-specific estimations. All included studies’ sample sizes are reported in the relevant data tables.

Future research should carefully balance practical constraints with the statistical rigor necessary to ensure valid and reliable outcomes. The methodological approach and multi-layered framework proposed in this review have broad applicability for evaluating the validity and reliability of technological devices used in sports and resistance training. Researchers can readily adapt this framework to establish standardized protocols, ensuring consistent and comprehensive evaluation across diverse technologies. Through replication and ongoing refinement, this approach will foster more uniform standards within sport science, ultimately improving comparability among studies and facilitating informed decision-making by practitioners.

Importantly, merely replicating arbitrary statistical thresholds for simplicity does not enhance certainty regarding validity and reliability. In contrast, the proposed framework integrates both statistician-driven and domain expert-driven criteria, thus mitigating the arbitrary nature of previously established thresholds and addressing variability across studies. While this multi-layered framework constitutes a significant methodological advancement, it relies on established statistical measures and expert-defined criteria. As sports technology continues to evolve, novel statistical analyses capable of capturing additional aspects of error, bias, or reliability beyond current methodologies are likely to emerge. Therefore, future iterations of this framework should incorporate advanced analytical techniques to ensure ongoing robustness and comprehensiveness. By maintaining adaptability, this framework aims to preserve its relevance and strengthen confidence in the validity and reliability assessments of evolving sports technologies.

## Conclusions

This review systematically assessed the available literature to evaluate the validity and reliability of velocity-based training devices by applying our proposed comprehensive framework. Developed in collaboration with statisticians and domain experts, this framework was applied to all 75 studies identified through our systematic search. After application, only five studies met the validity criteria, and 16 met the reliability criteria. Despite the limited number of studies meeting these criteria, we included data from all relevant studies to fully demonstrate the framework’s application. The decision to include all relevant studies and extract the relevant data to then assess them using the framework was made to provide a thorough and transparent demonstration of how our framework operates across the entire landscape of validity and reliability studies (Tables S7–S16 in S1 File). Excluding studies that did not meet the highest standards as part of the exclusion criteria would have severely limited the scope and potentially introduced bias into our conclusions. By incorporating all studies, we ensure that our analysis captures a broad spectrum of literature, allowing for a more nuanced understanding of the framework’s utility and limitations. This inclusive approach allows us to present conclusions and practical applications for S&C coaches that are based on a comprehensive analysis of the available evidence. It demonstrates how the framework functions across varying levels of methodological quality, thereby enhancing the reliability and applicability of our findings.

The main conclusions of this review reported that the GymAware LPT device is a valid and reliable tool to measure vertical barbell displacement for the F/W back squat across a load range of 70–90% 1RM, with the majority of investigations failing to meet the methodological validity criteria. The Perch motion capture single camera system can be a valid and reliable tool for measuring mean and peak velocity of the F/W back squat and F/W bench press across all loads ranging from 20–100% 1RM. The Flex optical device can also be a valid and reliable tool for measuring mean velocity for the F/W back squat and F/W bench press across all loads ranging from 20–90% 1RM. The Flex and Perch VBT devices showed the most robust validity and reliability assessments and were the only devices to be assessed for true technological reliability on a mechanically calibrated rig setup. The VmaxPro was the only IMU/accelerometer device identified to be a valid and reliable tool to measure mean velocity and displacement for the F/W back squat at 75–95% 1RM. The novel Full-Waveform Resistance Training Monitoring System (FRTMS) was valid for the S/M back squat across loads ranging from 30–90% 1RM for mean velocity, eccentric mean velocity, and the full waveform velocity metric. This review emphasizes the need to establish standardized guidelines and consistent statistical practices for future validity and reliability assessments in sport science, along with clear recommendations for interpreting results within their specific contexts. Future investigations should aim to apply a gold-standard criterion in the form of 3D motion capture across a broader range of exercises and loading conditions, as well as differentiate between biological and technological reliability for greater device precision.

## Practical applications

**Device Selection:** S&C coaches could consider using the GymAware LPT device for vertical barbell displacement for the F/W back squat within a 70–90% 1RM load range. For a broader range of measures and exercises, the Perch motion capture single camera system and the Flex optical device are also recommended. The VmaxPro IMU device is a valid and reliable tool to measure mean velocity and displacement for the F/W back squat at 75–95% 1RM. Additionally, practitioners should avoid using VBT devices to measure force and power related metrics due to the inherent flaws in the methods of measurement that limit their validation to a gold standard force plate. **Load Range:** When using the Perch and Flex devices, coaches can confidently measure mean velocity for the F/W back squat and F/W bench press across all loads ranging from 20–100% 1RM (Perch) and 20–90% 1RM (Flex). For the VmaxPro device, loads ranging from 75–95% 1RM were valid and reliable. **Technological Reliability:** Given that the Flex and Perch devices were the only ones assessed for true technological reliability, these should be prioritized when true technological reliability is a critical factor. **Contextual Interpretation:** Coaches and researchers should be cautious when generalizing findings and should consider the specific context in which the device will be used. VBT devices should be used to evaluate barbell displacement and velocity, but not to approximate force or power as this would only permit estimations of force and power applied to the barbell and not the entire system.

## Supporting information

S1 File Supplemental Digital Content Tables S1–S17. (DOCX)

S2 File PRISMA Checklist PLOSONE- Systematic Review of VBT Devices. (DOCX)
