# Supplementary material for: A systematic review using a multi-layered criteria framework for assessing the validity and reliability of velocity monitoring devices in resistance training
Source: PLoS One. 2025 Sep 8;20(9):e0324606. doi: 10.1371/journal.pone.0324606 (PMC12416690; doi:10.1371/journal.pone.0324606)
Supplement: S1 File — (DOCX) [file pone.0324606.s001.docx]

S1 Table: Search terms and key words utilized in each database. Searches were combined using the BOOLEAN operator “AND”.

| **Database:** | **PubMed** | | |
| --- | --- | --- | --- |
| *Search 1* | *Search 2* | *Search 3* | |
| *Title/Abstract: "Linear position transducer" OR "Accelerometer" OR "High-speed camera" OR "Laser optics" OR "GymAware" OR "Push Band" OR "FLEX" OR "Tendo" OR "Beast sensor" OR "Trio-OptiTrack" OR "T-Force" OR "Chronojump" OR "Speed4Lift" OR "Velowin" OR "PowerLift" OR "WIMU" OR "iLOAD" OR “3D Motion Capture” OR “2D Motion Capture” OR "Perch" | *Title/Abstract: "Validity" OR "Reliability" OR "Agreement" OR "Accuracy" OR "Precision" OR "Consistency" OR "Test-retest" OR "Inter-device" OR "Intra-device" | *Title/Abstract: (Velocity OR Force OR Power) AND (Resistance Training OR Exercise OR Strength OR Speed OR Plyometric) | |
| **Database:** | **Scopus** | | |
| *Search 1* | *Search 2* | *Search 3* | |
| TITLE-ABS-KEY ("Linear position transducer" OR "Accelerometer" OR "High-speed camera" OR "Laser optics" OR "GymAware" OR "Push Band" OR "FLEX" OR "Tendo" OR "Beast sensor" OR "Trio-OptiTrack" OR "T-Force" OR "Chronojump" OR "Speed4Lift" OR "Velowin" OR "PowerLift" OR "WIMU" OR "iLOAD" OR "3D Motion Capture" OR "2D Motion Capture" OR "Perch") | TITLE-ABS-KEY ("Validity" OR "Reliability" OR "Agreement" OR "Accuracy" OR "Precision" OR "Consistency" OR "Test-retest" OR "Inter-device" OR "Intra-device") | TITLE-ABS-KEY (Velocity OR Force OR Power OR Resistance Training OR Exercise OR Strength OR Speed OR Plyometric) | |
| **Database:** | **SPORTDiscus** | | |
| *Search 1* | *Search 2* | *Search 3* | *Search 4* |
| (TI "Linear position transducer" OR TI "Accelerometer" OR TI "High-speed camera" OR TI "Laser optics" OR TI "GymAware" OR TI "Push Band" OR TI "FLEX" OR TI "Tendo" OR TI "Beast sensor" OR TI "Trio-OptiTrack" OR TI "T-Force" OR TI "Chronojump" OR TI "Speed4Lift" OR TI "Velowin" OR TI "PowerLift" OR TI "WIMU" OR TI "iLOAD" OR TI "3D Motion Capture" OR TI "2D Motion Capture" OR TI "Perch" OR AB "Linear position transducer" OR AB "Accelerometer" OR AB "High-speed camera" OR AB "Laser optics" OR AB "GymAware" OR AB "Push Band" OR AB "FLEX" OR AB "Tendo" OR AB "Beast sensor" OR AB "Trio-OptiTrack" OR AB "T-Force" OR AB "Chronojump" OR AB "Speed4Lift" OR AB "Velowin" OR AB "PowerLift" OR AB "WIMU" OR AB "iLOAD" OR AB "3D Motion Capture" OR AB "2D Motion Capture" OR AB "Perch") | (TI Validity OR TI Reliability OR TI Agreement OR TI Accuracy OR TI Precision OR TI Consistency OR TI "Test-retest" OR TI "Inter-device" OR TI "Intra-device" OR AB Validity OR AB Reliability OR AB Agreement OR AB Accuracy OR AB Precision OR AB Consistency OR AB "Test-retest" OR AB "Inter-device" OR AB "Intra-device") | (TI Velocity OR TI Force OR TI Power OR AB Velocity OR AB Force OR AB Power) | (TI "Resistance Training" OR TI Exercise OR TI Strength OR TI Speed OR TI Plyometric OR AB "Resistance Training" OR AB Exercise OR AB Strength OR AB Speed OR AB Plyometric) |

| S2 Table: Modified Downs and Black methodological reporting quality questions. | | |
| --- | --- | --- |
| **No.** | **Item** | **Score** |
| 1 | Is the hypothesis/aim/objective of the study clearly stated? | 0-1 |
| 2 | Are the outcome measures clearly stated? | 0-1 |
| 3 | Are details of the microtechnology (i.e. manufacturer, sampling frequency) stated? | 0-1 |
| 6 | Are the findings of the study clearly described? | 0-1 |
| 7 | Does the study provide estimates of the random variability in the data for the main outcomes? | 0-1 |
| 10 | Have actual statistical values been reported? | 0-1 |
| 16 | If any of the results were based on data dredging, was this made clear? | 0-1 |
| 18 | Were the statistical tests used appropriate? | 0-1 |
| 20 | Were the criterion measures used valid and reliable? | 0-1 |
|  | **Total** | **0-9** |

| S3 Table: Total number of validity investigations via Velocity Based Training device. | | |
| --- | --- | --- |
| **TYPE** | **DEVICE NAME** | **NUMBER OF VALIDITY INVESTIGATIONS** |
| Linear  Position  Transducer | GymAware | 10 |
|  | Rep One | 0 |
|  | Tendo | 6 |
|  | Vitruve | 0 |
|  | T-Force | 3 |
|  | SmartCoach | 1 |
|  | 1080Q | 2 |
|  | FitroDyne (fitronic) | 2 |
|  | Open Barbell System | 2 |
|  | Musclelab (Ergotest) | 0 |
|  | ChronoJump | 2 |
|  | Speed4Lift | 3 |
|  | Functional electromechanical dynamometer (FEMD) | 1 |
|  | Full-waveform resistance training monitoring system  (FRTMS) | 1 |
|  | Jueying (Beijing, China) | 1 |
|  | ADR Encoder | 3 |
| IMU/ Accelerometer | Push Band | 12 |
|  | Push Band 2.0 | 7 |
|  | MyoTest | 1 |
|  | VmaxPro | 6 |
|  | WIMU System | 3 |
|  | RehaGait | 1 |
|  | Beast Sensor | 4 |
|  | Bar Sensei | 3 |
|  | Output Sports Unit | 1 |
|  | Accelerometer mobile basic program (MBP) via Huawei G620S smartphone | 1 |
|  | Apple Sports Watch | 1 |
| 2D Motion  Analysis | Powerlift | 6 |
|  | Mylift | 1 |
|  | My Jump Lab v 3.0  with iPhone 12 Pro running iOS 15.5  (previously My Lift) | 0 |
|  | iLoad v1.0 | 3 |
|  | Iron Path (version 1.9) App Via iPhone 8 | 1 |
|  | Kinovea via Samsung S6 | 1 |
|  | Kinovea via Xiaomi A1 | 1 |
|  | Kinovea via Casio FH20 | 1 |
|  | Kinovea via iPhone X | 1 |
|  | Kinovea via  Smartphone (Redmi Note 8) | 1 |
|  | Kinovea via Digital Camera | 1 |
|  | Novel video system via  Pocophone F1 | 1 |
|  | Tracker 5.0.6 software via  Casio Exilim Pro EX-F1 | 1 |
| 3D Motion  Analysis/Advanced Camera Systems | Elite Form Training System (EFTS) | 0 |
|  | Perch | 1 |
| Optic Devices/ Laser | Flex | 2 |
|  | Velowin | 7 |
| **Total** | | **105** |

| S4 Table: Total number of reliability investigations via Velocity Based Training device. | | |
| --- | --- | --- |
| **TYPE** | **DEVICE NAME** | **NUMBER OF RELIABILITY INVESTIGATIONS** |
| Linear  Position  Transducer | GymAware | 11 |
|  | Rep One | 0 |
|  | Tendo | 5 |
|  | Vitruve | 0 |
|  | T-Force | 13 |
|  | SmartCoach | 1 |
|  | 1080Q | 1 |
|  | FitroDyne (fitronic) | 1 |
|  | Open Barbell System | 0 |
|  | Musclelab (Ergotest) | 1 |
|  | ChronoJump | 2 |
|  | Speed4Lift | 5 |
|  | Functional electromechanical dynamometer (FEMD) | 1 |
|  | Jueying (Beijing, China) | 1 |
|  | ADR Enconder | 2 |
| IMU/ Accelerometer | Push Band | 6 |
|  | Push Band 2.0 | 5 |
|  | MyoTest | 1 |
|  | VmaxPro | 4 |
|  | WIMU System | 3 |
|  | RehaGait | 1 |
|  | Beast Sensor | 3 |
|  | Bar Sensei | 3 |
|  | Output Sports Unit | 1 |
|  | Accelerometer mobile basic program (MBP) via Huawei G620S smartphone | 1 |
|  | Apple Watch Sport (1st generation) via Phone 6s with iOS 11.4.1 installed.  + StrengthControl App | 0 |
| 2D Motion  Analysis | Powerlift | 6 |
|  | Mylift | 1 |
|  | My Jump Lab v 3.0  with iPhone 12 Pro running iOS 15.5  (previously My Lift) | 1 |
|  | iLoad v1.0 | 1 |
|  | Iron Path (version 1.9) App Via iPhone 8 | 0 |
|  | Kinovea via Samsung S6 | 0 |
|  | "Kinovea via Xiaomi A1 | 0 |
|  | Kinovea via Casio FH20 | 0 |
|  | Kinovea via iPhone X | 0 |
|  | Kinovea via  Smartphone (Redmi Note 8) | 1 |
|  | Kinovea via Digital Camera | 0 |
|  | Novel video system via  Pocophone F1 | 1 |
|  | Tracker 5.0.6 software via  Casio Exilim Pro EX-F1 | 1 |
| 3D Motion  Analysis/Advanced Camera Systems | Elite Form Training System (EFTS) | 1 |
|  | Perch | 1 |
| Optic Devices/ Laser | Flex | 1 |
|  | Velowin | 7 |
| **Total** | | **94** |

S5 Table: List of exercises and their total times used per device type in both validity and reliability investigations.

| **Exercise Name** | **TOTAL** | **Validity Investigations** | | | | | **Reliability Investigations** | | | | |
| --- | --- | --- | --- | --- | --- | --- | --- | --- | --- | --- | --- |
|  |  | **LPT** | **IMU** | **2D Motion Capture** | **3D Motional Analysis/Advanced Camera System** | **Optic** | **LPT** | **IMU** | **2D Motion Capture** | **3D Motional Analysis/Advanced Camera System** | **Optic** |
| **Total Number of Exercises** | 31 | 17 | 22 | 10 | 2 | 11 | 20 | 14 | 8 | 5 | 9 |
| **Total Number of Measures** | 360 | 64 | 80 | 30 | 2 | 18 | 75 | 44 | 20 | 7 | 17 |
| F/W Back Squat | 94 | 18 | 25 | 5 | 1 | **4** | 17 | 16 | 3 | 2 | 3 |
| S/M Bench Press | 57 | 10 | 3 | 10 | 0 | 3 | 18 | 3 | 5 | 0 | 4 |
| S/M Back Squat | 36 | 5 | 3 | 5 | 0 | 2 | 10 | 3 | 5 | 0 | 2 |
| F/W Bench Press | 44 | 5 | 13 | 3 | 1 | 1 | 7 | 8 | 3 | 2 | 1 |
| F/W Deadlift | 15 | 5 | 6 | 1 | 0 | 0 | 1 | 0 | 1 | 1 | 0 |
| Power Clean | 10 | 1 | 4 | 0 | 0 | 0 | 1 | 3 | 0 | 1 | 0 |
| F/W Hip Thrust | 10 | 0 | 2 | 1 | 0 | 0 | 3 | 3 | 1 | 0 | 0 |
| CMJ | 9 | 3 | 2 | 0 | 0 | 2 | 0 | 1 | 0 | 0 | 1 |
| F/W Ballistic Squat | 8 | 3 | 1 | 0 | 0 | 0 | 3 | 1 | 0 | 0 | 0 |
| S/M Prone Bench Pull | 8 | 1 | 0 | 0 | 0 | 1 | 4 | 0 | 0 | 0 | 2 |
| Squat Jump | 9 | 2 | 4 | 0 | 0 | 1 | 1 | 0 | 0 | 0 | 0 |
| Hang Power Snatch | 5 | 2 | 2 | 0 | 0 | 1 | 0 | 0 | 0 | 0 | 0 |
| Power Snatch | 5 | 2 | 2 | 0 | 0 | 1 | 0 | 0 | 0 | 0 | 0 |
| S/M Half ROM  Back Squat | 5 | 0 | 0 | 2 | 0 | 1 | 0 | 0 | 1 | 0 | 1 |
| Leg Extension | 4 | 0 | 1 | 1 | 0 | 0 | 1 | 0 | 1 | 0 | 0 |
| F/W Standing  Bent Over Rows | 2 | 0 | 2 | 0 | 0 | 0 | 0 | 0 | 0 | 0 | 0 |
| Push-Up | 3 | 0 | 1 | 0 | 0 | 0 | 1 | 1 | 0 | 0 | 0 |
| S/M Concentric-Only  Half ROM Back Squat | 3 | 0 | 1 | 0 | 0 | 0 | 1 | 1 | 0 | 0 | 0 |
| Calibrated Rig | 2 | 0 | 0 | 0 | 0 | 0 | 0 | 0 | 0 | 0 | 2 |
| F/W Front Squat | 7 | 2 | 1 | 1 | 0 | 0 | 1 | 1 | 0 | 1 | 0 |
| F/W Loaded  Squat Jump | 2 | 1 | 1 | 0 | 0 | 0 | 0 | 0 | 0 | 0 | 0 |
| Loaded CMJ | 3 | 0 | 0 | 0 | 0 | 1 | 0 | 1 | 0 | 0 | 1 |
| S/M Bent Over Row | 2 | 1 | 0 | 0 | 0 | 0 | 1 | 0 | 0 | 0 | 0 |
| Bilateral & Unilateral  Ballistic Leg Extensions  on a Leg Press Machine | 1 | 0 | 0 | 1 | 0 | 0 | 0 | 0 | 0 | 0 | 0 |
| DB Biceps Curl | 1 | 0 | 1 | 0 | 0 | 0 | 0 | 0 | 0 | 0 | 0 |
| DB Shoulder Press | 1 | 0 | 1 | 0 | 0 | 0 | 0 | 0 | 0 | 0 | 0 |
| F/W Biceps Curls | 1 | 0 | 0 | 0 | 0 | 0 | 1 | 0 | 0 | 0 | 0 |
| Barbell Landmine Punch | 1 | 0 | 0 | 0 | 0 | 0 | 1 | 0 | 0 | 0 | 0 |
| Barbell Landmine  Punch Throw | 1 | 0 | 0 | 0 | 0 | 0 | 1 | 0 | 0 | 0 | 0 |
| Barbell Jump Shrug | 4 | 1 | 1 | 0 | 0 | 0 | 1 | 1 | 0 | 0 | 0 |
| Barbell Hang High Pull | 4 | 1 | 1 | 0 | 0 | 0 | 1 | 1 | 0 | 0 | 0 |

*Abbreviations: CMJ: Countermovement Jump; DB: Dumbbell; F/W: Free Weight; ROM: Range of Motion; S/M: Smith Machine.*

S6 Table: List of metrics and the total times used per device type in both validity and reliability investigations.

| **Exercise Name** | **TOTAL** | **Validity Investigations** | | | | | **Reliability Investigations** | | | | |
| --- | --- | --- | --- | --- | --- | --- | --- | --- | --- | --- | --- |
|  |  | **LPT** | **IMU** | **2D Motion Capture** | **3D Motional Analysis/Advanced Camera System** | **Optic** | **LPT** | **IMU** | **2D Motion Capture** | **3D Motional Analysis/Advanced Camera System** | **Optic** |
| **Total Number**  **of Metrics**  **Investigated** | 18 | **11** | 9 | **6** | **2** | 4 | **12** | 4 | **3** | 2 | 4 |
| **Total Number**  **of Investigations** | 358 | 67 | 70 | **32** | **2** | 16 | 85 | 47 | **15** | 4 | **20** |
| Mean Velocity | 191 | 30 | **40** | 18 | 1 | 8 | 41 | 29 | 12 | 2 | 11 |
| Peak Velocity | 96 | 19 | 23 | 3 | 1 | 5 | 20 | 16 | 2 | 2 | 5 |
| Time | 14 | 4 | 1 | 4 | 0 | 0 | 4 | 1 | 0 | 0 | 0 |
| Mean Propulsive Velocity | 11 | 1 | 0 | 0 | 0 | 2 | 5 | 0 | 0 | 0 | 3 |
| Maximum Velocity | 10 | 3 | 1 | 0 | 0 | 1 | 4 | 0 | 0 | 0 | 1 |
| Eccentric Mean Velocity | 3 | 0 | 1 | 0 | 0 | 0 | 1 | 1 | 0 | 0 | 0 |
| Time to Maximum Velocity | 7 | 3 | 1 | 0 | 0 | 0 | 3 | 0 | 0 | 0 | 0 |
| Distance | 4 | 0 | 0 | 4 | 0 | 0 | 0 | 0 | 0 | 0 | 0 |
| Displacement | 4 | 2 | 0 | 2 | 0 | 0 | 2 | 0 | 1 | 0 | 0 |
| Left And Right  Placement Conditions of LPT | 2 | 0 | 0 | 0 | 0 | 0 | 2 | 0 | 0 | 0 | 0 |
| Time Spent  at Isokinetic Velocity  (m/s) | 2 | 1 | 0 | 0 | 0 | 0 | 1 | 0 | 0 | 0 | 0 |
| Time to Reach Isokinetic Velocity (m/s) | 2 | 1 | 0 | 0 | 0 | 0 | 1 | 0 | 0 | 0 | 0 |
| Total Work | 2 | 0 | 0 | 1 | 0 | 0 | 1 | 0 | 0 | 0 | 0 |
| Barbell  Displacement  (Vertical) | 1 | 1 | 0 | 0 | 0 | 0 | 0 | 0 | 0 | 0 | 0 |
| Exercise  Recognition | 1 | 0 | 1 | 0 | 0 | 0 | 0 | 0 | 0 | 0 | 0 |
| Repetition Count | 1 | 0 | 1 | 0 | 0 | 0 | 0 | 0 | 0 | 0 | 0 |
| 1RM Prediction | 1 | 0 | 1 | 0 | 0 | 0 | 0 | 0 | 0 | 0 | 0 |
| Full Waveform Velocity | 1 | 1 | 0 | 0 | 0 | 0 | 0 | 0 | 0 | 0 | 0 |

*Abbreviations: 1RM: One Repetition Maximum; LPT: Linear Position Transducer; m: meters; m/s: Meters per Second.*

*S7 Table: Studies investigating the validity of a Linear Position Transducer device.*

| **Study** | **Device/s** | **Criterion** | **Exercise/s** | | **Intensity/Load** | **Variable/s Measured** | **Sample Size** | **Reported Statistics** | | **Validity Criteria** |
| --- | --- | --- | --- | --- | --- | --- | --- | --- | --- | --- |
| Appleby, Banyard [45] | GymAware | Vicon 3D Motion Analysis  Barbell Displacement Criterion: 7th cervical vertebrae reflective marker | F/W Back Squat | | 70-90% 1RM | Vertical Barbell Displacement | 12 | Typical Error as a CV: 2.5% (Confidence Limit: 2.2%-2.8%)  Pearson correlation: 0.97 (Confidence Limit: 0.96-0.98)  Overall mean bias: 1.3% (Confidence Limit: 0.9%-1.8%) | | Was a gold standard criterion used? **YES** / NO  Where the statistics/combination of used to validate the device appropriate? **YES** / NO  Did the original study claim the device was valid? **YES** / NO  Does this device validly measure what was measured? **YES** / NO |
| Askow, Stone [49] | GymAware | Qualysis Motion Capture System | F/W Back Squat | | 75-90% 1RM | Mean Velocity, Peak Velocity | 9 | Mean Velocity  Mean Bias: 0.03 m/s  ICC: 0.966  ES: 0.28  SEE: 0.04 m/s  Peak Velocity  Mean Bias: -0.12 m/s  ICC: 0.982  ES: -0.57  SEE: 0.05 m/s | | Was a gold standard criterion used? **YES** / NO  Where the statistics/combination of used to validate the device appropriate? YES / **NO**  Did the original study claim the device was valid? **YES** / NO  Does this device validly measure what was measured? YES / **NO** |
| Banyard, Nosaka [3] | GymAware | 4 x Celesco PT5A-250 LPT | F/W Back Squat | | 20%, 40%, 60%, 80%, 90%, 100% 1RM | Mean Velocity, Peak Velocity | 10 | 20% 1RM Mean Velocity r: 0.96; CV: 3.6%; ES: 0.17; SEE: 0.04 m/s Peak Velocity r: 0.94; CV: 4.1%; ES: - 0.03; SEE: 0.08 m/s Mean Force r: 0.99; CV: 2.2%; ES: 0.57; SEE: 22.57 N Peak Force r: 0.96; CV: 5.5%; ES: 0.61; SEE: 114.2 N Mean Power r: 0.98; CV: 4%; ES: 0.72; SEE: 49.7 W  40% 1RM Mean Velocity r: 0.97; CV: 3.2%; ES: 0.19; SEE: 0.03 m/s Peak Velocity r: 0.97; CV: 2.9%; ES: 0.02; SEE: 0.05 m/s Mean Force r: 0.99; CV: 1.8%; ES: 0.37; SEE: 25.75 N Peak Force r: 0.99; CV: 3.4%; ES: 0.38; SEE: 65.1 N Mean Power r: 0.97; CV: 4.3%; ES: 0.6; SEE: 55.39 W Peak Power r: 0.97; CV: 5.7%; ES: 0.67; SEE: 143.87 W  60% 1RM  Mean Velocity r: 0.95; CV: 3.1%; ES: 0.11; SEE: 0.03 m/s Peak Velocity r: 0.96; CV: 3.7%; ES: - 0.02; SEE: 0.05 m/s Mean Force r: 0.99; CV: 1.7%; ES: 0.29; SEE: 29.33 N Peak Force r: 0.99; CV: 3.3%; ES: 0.29; SEE: 74.63 N Mean Power r: 0.97; CV: 4.7%; ES: 0.34; SEE: 66.02 W Peak Power r: 0.97; CV: 5.7%; ES: 0.53; SEE: 152.84W  80% 1RM Mean Velocity r: 0.96; CV: 3%; ES: 0.14; SEE: 0.02 m/s Peak Velocity r: 0.97; CV: 5.4%; ES: 0.07; SEE: 0.06 m/s Mean Force r: 0.99; CV: 1.6%; ES: 0.16; SEE: 34.88 N Peak Force r: 0.99; CV: 3.5%; ES: 0.29; SEE: 79.78 N Mean Power r: 0.99; CV: 3.5%; ES: 0.27; SEE: 37.79 W Peak Power r: 0.98; CV: 6%; ES: 0.36; SEE: 145.79 W  90% 1RM Mean Velocity r: 0.96; CV: 2.8%; ES: 0.06; SEE: 0.02 m/s Peak Velocity r: 0.95; CV: 5.8%; ES: 0.12; SEE: 0.06 m/s Mean Force r: 1.00; CV: 1.1%; ES: 0.1; SEE: 23.83 N Peak Force r: 0.99; CV: 3.2%; ES: 0.29; SEE: 85.27 N Mean Power r: 0.99; CV: 4.7%; ES: 0.13; SEE: 44.9 W Peak Power r: 0.96; CV: 7.7%; ES: 0.33; SEE: 166.75 W  100% 1RM  Mean Velocity r: 0.99; CV: 2.5%; ES: 0.39; SEE: 0.01 m/s Peak Velocity r: 0.96; CV: 5.4%; ES: 0.04; SEE: 0.04 m/s Mean Force r: 1.00; CV: 0.7%; ES: 0.06; SEE: 16.9 N Peak Force r: 0.96; CV: 5.5%; ES: 0.13; SEE: 140.28 N Mean Power r: 0.99; CV: 5.3%; ES: 0.26; SEE: 23.89 W Peak Power r: 0.95; CV: 7.3%; ES: 0.19; SEE: 126.8 W | | Was a gold standard criterion used? YES / **NO**  Where the statistics/combination of used to validate the device appropriate? **YES** / NO  Did the original study claim the device was valid? **YES** / NO  Does this device validly measure what was measured? YES / ***NO*** |
| Bardella, Carrasquilla García [77] | (SmartCoach)  Power Encoder SPE-35 | N/A | Bench Press, CMJ, Squat Jump, Back Squat | | 10 kg barbell | Sampling Frequency (f) 100, 200, or 500 Hz; *the signal was artificially low pass filtered (limited) at Nyquist frequencies of 100, 50, and 25 Hz | 8 | The values of the sampling frequency required to acquire a signal up to 99 and 99.9% of its total energy.  Data are presented as the mean ± SD  Bench Press  f99(Hz): 6.077 ± 1.021 f99.99(Hz): 11.615 ± 2.680  CMJ f99(Hz): 4.491 ± 0.971 f99.99(Hz): 9.562 ± 0.951  Squat Jump f99(Hz): 5.133 ± 0.560 f99.99(Hz): 10.001 ± 1.754  Squat  f99(Hz): 5.171 ± 0.614B f99.99(Hz): 11.107 ± 3.461   The sampling frequency needed to reconstruct the signals with an error less than 0.1 % was f99.9 = 11.615 ± 2.680 Hz for the exercise exhibiting the largest bandwidth (BP), with the absolute highest individual value being 17.467 Hz.  A sampling rate of 25 Hz or above is more than adequate to record raw speed data and compute power during resistance training exercises, even under the most extreme circumstances during explosive exercises. Higher sampling frequencies provide no increase in the recording precision and may instead have adverse effects on the overall data quality. | | N/A |
| Boehringer and Whyte [79] | 1080Q | GymAware | S/M Bench Press | | 40-80% 1RM | Mean Velocity Peak Velocity | 27 | Mean Velocity  MD: 0.018 ± 0.024 m/s;  r:1.00   Peak Velocity  MD: 0.033 ± 0.026 m/s;  r:1.00 | | Was a gold standard criterion used? YES / **NO**  Where the statistics/combination of used to validate the device appropriate? YES / **NO**  Did the original study claim the device was valid? **YES** / NO  Does this device validly measure what was measured? YES / **NO** |
| Callaghan, Guy [83] | Speed4Lifts | Vicon (Vero 2.2) 3D Motion Capture | F/W Back Squat,  F/W Front Squat,  F/W Bench Press | | Loads 20%, 40%, 60%, 80% 1RM, Velocities Fast (>1.00 m/s), Moderate (0.65 to 1.00 m/s, Slow (<0.65 m/s) | Mean Propulsive Velocity | 20 | Back Squat Fast (>1.00 m/s) Cusum Linearity Test: 0.96 p-value: 0.12 Intercept (A): 0.04 95% CI of Intercept: ± 0.19 Slope (B): 0.89 95% CI of Slope: 0.83 ± 0.96 Fixed Bias: Yes Proportional Bias: Yes Agreement: No  Moderate (0.65 to 1.00 m/s) Cusum Linearity Test: 0.51 p-value: −0.01 Intercept (A): −0.06 95% CI of Intercept: ± 0.04 Slope (B): 1.06 95% CI of Slope: 0.99 ± 1.12 Fixed Bias: No Proportional Bias: No Agreement: Yes  Slow (<0.65 m/s) Cusum Linearity Test: 0.84 p-value: 0.03 Intercept (A): 0.00 95% CI of Intercept: ± 0.07 Slope (B): 0.96 95% CI of Slope: 0.89 ± 1.02 Fixed Bias: No Proportional Bias: No Agreement: Yes  Front Squat Fast (>1.00 m/s) Cusum Linearity Test: 0.38 p-value: 0.15 Intercept (A): 0.05 95% CI of Intercept: ± 0.24 Slope (B): 0.88 95% CI of Slope: 0.81 ± 0.97 Fixed Bias: Yes Proportional Bias: Yes Agreement: No  Moderate (0.65 to 1.00 m/s) Cusum Linearity Test: 0.98 p-value: −0.04 Intercept (A): −0.08 95% CI of Intercept: ± 0.00 Slope (B): 1.09 95% CI of Slope: 1.04 ± 1.14 Fixed Bias: No Proportional Bias: Yes Agreement: No  Slow (<0.65 m/s) Cusum Linearity Test: 0.30 p-value: −0.02 Intercept (A): −0.06 95% CI of Intercept: ± 0.03 Slope (B): 1.05 95% CI of Slope: 0.96 ± 1.12 Fixed Bias: No Proportional Bias: No Agreement: Yes  Bench Press Fast (>1.00 m/s) Cusum Linearity Test: 0.57 p-value: 0.17 Intercept (A): 0.12 95% CI of Intercept: ± 0.22 Slope (B): 0.87 95% CI of Slope: 0.83 ± 0.91 Fixed Bias: Yes Proportional Bias: Yes Agreement: No  Moderate (0.65 to 1.00 m/s) Cusum Linearity Test: 0.54 p-value: 0.04 Intercept (A): −0.01 95% CI of Intercept: ± 0.09 Slope (B): 0.99 95% CI of Slope: 0.94 ± 1.06 Fixed Bias: No Proportional Bias: No Agreement: Yes  Slow (<0.65 m/s) Cusum Linearity Test: 0.19 p-value: 0.03 Intercept (A): 0.01 95% CI of Intercept: ± 0.05 Slope (B): 0.98 95% CI of Slope: 0.94 ± 1.03 Fixed Bias: Yes Proportional Bias: No Agreement: Yes | | Was a gold standard criterion used? **YES** / NO  Where the statistics/combination of used to validate the device appropriate? **YES** / NO  Did the original study claim the device was valid? **Back Squat (low to moderate velocities): Yes** **Back Squat (high velocities): No** **Front Squat (low velocities): Yes** **Front Squat (moderate to high velocities): No** **Bench Press (moderate velocities): Yes** **Bench Press (low and high velocities): No**  Does this device validly measure what was measured? YES / **NO** |
| Chéry and Ruf [62] | Tendo | GymAware | F/W Deadlift | | 20%, 40%, 60%,80%, 90%, 100% 1RM, All Loads | Mean Velocity, Peak Velocity | 10 | 20% 1RM  Mean Velocity CV: 1.72%;  MD: 0.03 m/s  Peak Velocity CV: 1.03%;  MD: -0.16 m/s   40% 1RM  Mean Velocity  CV: 2.17%;  MD: 0.01 m/s  Peak Velocity CV: 1.47%;  MD: -0.11 m/s   60% 1RM  Mean Velocity  CV: 2.65%;  MD: 0.01 m/s  Peak Velocity CV: 1.08%;  MD: -0.09 m/s   80% 1RM  Mean Velocity  CV: 2.99%;  MD: 0.06 m/s  Peak Velocity CV: 1.37%;  MD: -0.06 m/s   90% 1RM  Mean Velocity  CV: 3.50%;  MD: 0.04 m/s  Peak Velocity CV: 1.36%;  MD:- 0.04 m/s   100% 1RM  Mean Velocity  CV: 4.07%;  MD: 0.03 m/s  Peak Velocity CV: 3.76%;  MD: -0.03 m/s   All Loads Mean Velocity  CV: 4.08%;  MD: 0.03 m/s  Peak Velocity CV: 1.56%;  MD: -0.08 m/s | | Was a gold standard criterion used? YES / **NO**  Where the statistics/combination of used to validate the device appropriate? YES / **NO**  Did the original study claim the device was valid? **YES** / NO  Does this device validly measure what was measured? YES / **NO** |
| Courel-Ibáñez, Martínez-Cava [68] | Chronojump | T-Force | S/M Bench Press, S/M Back Squat, S/M Prone Bench Pull | | 20 kg, 30 kg, 40 kg, 50 kg, 60 kg, 70 kg, 80 kg | Mean Velocity,  Mean Propulsive Velocity, Peak Velocity | 17 | S/M Bench Press Mean Velocity  SEM: 0.05 m/s;  CV: 6.1%;  ICC: 0.992   Mean Propulsive Velocity  SEM: 0.06 m/s;  CV: 6.8%;  ICC: 0.997   Peak Velocity SEM: 0.04 m/s;  CV: 2.8%;  ICC: 0.998    S/M Back Squat Mean Velocity  SEM: 0.04 m/s;  CV: 10.6%;  ICC: 0.984   Mean Propulsive Velocity  SEM: 0.04 m/s;  CV: 4.7%;  ICC: 0.986   Peak Velocity SEM: 0.05 m/s;  CV: 2.9%;  ICC: 0.989    S/M Prone Bench Pull Mean Velocity  SEM: 0.04 m/s;  CV: 3.9%;  ICC: 0.993   Mean Propulsive Velocity  SEM: 0.03 m/s;  CV: 3.2%;  ICC: 0.994 J.   Peak Velocity SEM: 0.05 m/s;  CV: 2.8%;  ICC: 0.997 | | Was a gold standard criterion used? YES / **NO**  Where the statistics/combination of used to validate the device appropriate? **YES** / NO  Did the original study claim the device was valid? **YES** / NO  Does this device validly measure what was measured? YES / **NO** |
| Dorrell, Moore [50] | GymAware | Rapture-E 3D cameras | F/W Back Squat, F/W Bench Press, F/W Deadlift | | 80% 1RM | Barbell Displacement, Peak Velocity, Mean Velocity | 13 | F/W Back Squat  80% 1RM  Bar displacement MD:  -0.009 ± 0.005 m Peak Velocity  MD: 0.005 ± 0.007 m/s Mean Velocity  MD: 0.029 ± 0.010 m/s  F/W Bench Press  80% 1RM  Bar displacement MD: -0.009 ± 0.009 m Peak Velocity  MD: 0.002 ± 0.007 m/s Mean Velocity  MD: 0.017 ± 0.016 m/s  F/W Deadlift 80% 1RM  Bar displacement MD: -0.016 ± 0.009 m Peak Velocity  MD: 0.004± 0.004 m/s Mean Velocity  MD: 0.100 ± 0.037 m/s | | Was a gold standard criterion used? **YES** / NO  Where the statistics/combination of used to validate the device appropriate? YES / **NO**  Did the original study claim the device was valid? **YES** / NO  Does this device validly measure what was measured? YES / **NO** |
| Fernandes, Lamb [80] | FitroDyne  (fitronic) | GymAware | S/M Bench Press, S/M Bent Over Row | | 20%, 30%, 40%, 50%, 60%, 70%, 80% 1RM | Peak Velocity,  Mean Velocity | 15 | S/M BENCH PRESS 20% 1RM Peak Velocity  LoA: 11.2 + 25.9 cm/s; r: 0.86  Mean Velocity LoA: -4.8 + 13.6 cm/s; r: 0.92   30% 1RM Peak Velocity  LoA: 10.7 + 22.9 cm/s; r: 0.79  Mean Velocity LoA: -2.0 + 13.1 cm/s; r: 0.88   40% 1RM Peak Velocity  LoA: 10.7 + 12.1 cm/s; r: 0.92  Mean Velocity LoA: -0.9 + 9.3 cm/s;  r: 0.89   50% 1RM Peak Velocity  LoA: 10.1 + 4.5 cm/s;  r: 0.98  Mean Velocity LoA: 0.1 + 9.5 cm/s;  r: 0.86   60% 1RM Peak Velocity  LoA: 7.7 + 3.8 cm/s; r: 0.99  Mean Velocity LoA: 0.0 + 11.6 cm/s;  r: 0.86   70% 1RM Peak Velocity  LoA: 7.5 + 9.6 cm/s;  r: 0.95  Mean Velocity LoA: 0.2 + 7.4 cm/s;  r: 0.93   80% 1RM Peak Velocity  LoA: 5.5 + 10.1 cm/s;  r: 0.96  Mean Velocity LoA: 1.0 + 6.8 cm/s;  r: 0.94   S/M BACK SQUAT 20% 1RM Peak Velocity  LoA: 12.0 + 8.8 cm/s;  r: 1.00  Mean Velocity LoA: 2.0 + 6.3 cm/s;  r: 0.98   30% 1RM Peak Velocity  LoA: 10.6 + 9.3 cm/s;  r: 0.99  Mean Velocity LoA: 0.6 + 7.5 cm/s;  r: 0.97   40% 1RM Peak Velocity  LoA: 10.4 + 9.1 cm/s;  r: 0.99  Mean Velocity LoA: 1.2 + 5.4 cm/s;  r: 0.98   50% 1RM Peak Velocity  LoA: 9.2 + 7.4 cm/s m/s; r: 0.99  Mean Velocity LoA: 2.3 + 3.3 cm/s;  r: 0.99  60% 1RM Peak Velocity  LoA: 8.5 + 5.8 cm/s;  r: 1.00  Mean Velocity LoA: 2.0 + 3.0 cm/s; r: 0.99  70% 1RM Peak Velocity  LoA: 8.3 + 5.4 cm/s;  r: 1.00  Mean Velocity LoA: 2.2 + 2.1 cm/s;  r: 1.00   80% 1RM Peak Velocity  LoA: 8.0 + 6.5 cm/s;  r: 1.00  Mean Velocity LoA: 1.1 + 6.0 cm/s;  r: 0.94  S/M BENT OVER ROW 20% 1RM Peak Velocity  LoA: 14.6 + 25.0 cm/s; r: 0.94 Mean Velocity LoA: -0.1 + 12.0 cm/s; r: 0.96 30% 1RM Peak Velocity  LoA: 14.6 + 18.9 cm/s; r: 0.93  Mean Velocity LoA: -0.8 + 7.3 cm/s;  r: 0.97   40% 1RM Peak Velocity  LoA: 13.6 + 12.0 cm/s; r: 0.97 Mean Velocity LoA: 0.4 + 11.6 cm/s;  r: 0.91   50% 1RM Peak Velocity  LoA: 10.6 + 6.8 cm/s;  r: 0.98  Mean Velocity LoA: 0.6 + 4.9 cm/s;  r: 0.98   60% 1RM Peak Velocity  LoA: 7.3 + 18.2 cm/s;  r: 0.92  Mean Velocity LoA: 0.3 + 6.0 cm/s;  r: 0.97   70% 1RM Peak Velocity  LoA: 7.7 + 15.3 cm/s;  r: 0.91 Mean Velocity LoA:- 0.2 + 4.2 cm/s;  r: 0.98   80% 1RM Peak Velocity  LoA: 7.3 + 14.9 cm/s;  r: 0.92  Mean Velocity  LoA: -0.2 + 3.9 cm/s;  r: 0.98 | | Was a gold standard criterion used? YES / **NO**  Where the statistics/combination of used to validate the device appropriate? YES / **NO**  Did the original study claim the device was valid? YES / **NO**  Does this device validly measure what was measured? YES / **NO** |
| Fritschi, Seiler [51] | GymAware | Vicon Vantage 5 3D Motion Capture | Hang Power Snatch, CMJ, Squat Jump, F/W Back Squat | | Hang power snatch 20 kg, CMJ 50% of the load determined for moderate back squat (mean ± standard deviation: 34 ± 10 kg), Squat Jump 50% of the load determined for moderate back squat (mean ±  standard deviation: 34 ±10 kg), F/W back squat (Moderate) = (65 ± 20 kg) to elicit Vmean in the range of 0.7-0.8 m/s (mean ± standard deviation of actual values: 0.75 ±  0.05 m/s), F/W back squat (Heavy) = Heavy back squat with the individual load determined during warm-up (90 ± 20 kg) to elicit Vmean of just under 0.5 m/s (actual values: 0.47 ± 0.05 m/s) | Mean Velocity, Peak Velocity | 14 | Mean Velocity  r: 0.99 (0.90-0.98)  SEE: 0.06 m/s (0.01-0.08)  SEE%: 5.4% (2.0-4.5)  Peak Velocity  r: 0.99 (0.95-1.00)  SEE: 0.08 m/s (0.03-0.06)  SEE%: 3.6% (1.0-4.0) | | Was a gold standard criterion used? **YES** / NO  Where the statistics/combination of used to validate the device appropriate? YES / **NO**  Did the original study claim the device was valid? **YES** / NO  Does this device validly measure what was measured? YES / **NO** |
|  | 1080Q | Vicon Vantage 5 3D Motion Capture | Hang Power Snatch, CMJ, Squat Jump, F/W Back Squat | | Hang power snatch 20 kg,  CMJ 50% of the load determined for moderate back squat (mean ± standard deviation: 34 ± 10 kg),  Squat Jump 50% of the load determined for moderate back squat (mean ±  standard deviation: 34 ±10 kg),  F/W back squat (Moderate) = (65 ± 20 kg) to elicit Vmean in the range of 0.7-0.8 m/s (mean ± standard deviation of actual values: 0.75 ±  0.05 m/s),  F/W back squat (Heavy) = Heavy back squat with the individual load determined during warm-up (90 ± 20 kg) to elicit Vmean of just under 0.5 m/s (actual values: 0.47 ± 0.05 m/s) | Mean Velocity,  Peak Velocity | 14 | Mean Velocity  r: 0.97 (0.88-0.98)  SEE: 0.13 m/s (0.01-0.18)  SEE%: 11.7% (1.6-9.8)  Peak Velocity  r: 1.00 (0.97-1.00)  SEE: 0.07 m/s (0.03-0.06)  SEE%: 3.2% (1.0-2.8) | | Was a gold standard criterion used? **YES** / NO  Where the statistics/combination of used to validate the device appropriate? YES / **NO**  Did the original study claim the device was valid? **YES** / NO  Does this device validly measure what was measured? YES / **NO** |
| Garnacho-Castaño, López-Lastra [60] | Tendo | T-Force | S/M Back Squat, S/M Bench Press | | 40-60 kg  (85% 1RM) | Mean Velocity,  Peak Velocity | 71 | S/M Back Squat 40-60 kg (85% 1RM)  Mean Velocity Bias: 0.02 ± 0.07 m/s; ICC: 0.985  Peak Velocity Bias: - 0.08 ± 0.13 m/s; ICC: 0.963     S/M Bench Press 40-60 kg (85% 1RM)  Mean Velocity Bias: 0.01 ± 0.06 m/s; ICC: 0.989   Peak Velocity Bias: -0.06 ± 0.10 m/s; ICC: 0.963 | | Was a gold standard criterion used? YES / **NO**  Where the statistics/combination of used to validate the device appropriate? YES / **NO**  Did the original study claim the device was valid? **YES** / NO  Does this device validly measure what was measured? YES / **NO** |
| Goldsmith, Trepeck [63] | Tendo | OptiTrak 3D Motion Capture | F/W Back Squat | | 70% 1RM | Mean Velocity, Peak Velocity | 25 | Mean Velocity  LoA (95% CI): - 0.006357 (- 0.06042 to 0.04771) m/s; ICC: 0.9364  Peak concentric velocity LoA (95% CI): 0.07569 (- 0.05406 to 0.2054) m/s; ICC: 0.9362 | | Was a gold standard criterion used? **YES** / NO  Where the statistics/combination of used to validate the device appropriate? YES / **NO**  Did the original study claim the device was valid? **YES** / NO  Does this device validly measure what was measured? YES / **NO** |
|  | Open Barbell System | OptiTrak 3D Motion Capture | F/W Back Squat | | 70% 1RM | Mean Velocity, Peak Velocity | 25 | Mean Velocity  LoA (95% CI): - 0.01163 (- 0.06855 to 0.04528) m/s; ICC: 0.8696  Peak concentric velocity LoA (95% CI): 0.03986 (- 0.1016 to 0.1813) m/s; ICC: 0.8351 | | Was a gold standard criterion used? **YES** / NO  Where the statistics/combination of used to validate the device appropriate? YES / **NO**  Did the original study claim the device was valid? **YES** / NO  Does this device validly measure what was measured? YES / **NO** |
| Gonzalez, Mangine [81] | Open Barbell System | Tendo | F/W Back Squat, F/W Front Squat | | 30%, 50%, 70%, 90% 1RM | Peak Velocity,  Mean Velocity | 11 | F/W Back Squat  Overall Peak Velocity MD: 0.11 + 0.01 m/s Mean Velocity  MD: 0.01 + 0.01 m/s  30% 1RM  Peak Velocity r: 0.95; Bias: 0.163 m/s Mean Velocity  r: 0.27; Bias: 0.012 m/s  50% 1RM  Peak Velocity  r: 0.73; Bias: 0.125 m/s Mean Velocity  r: 0.35; Bias: 0.007 m/s  70% 1RM  Peak Velocity r: 0.87; Bias: 0.086 m/s Mean Velocity  r: 0.03; Bias: 0.008 m/s  90% 1RM  Peak Velocity r: 0.58; Bias: 0.053 m/s Mean Velocity  r: 0.25; Bias: 0.009 m/s  F/W Front Squat   Overall Peak Velocity MD: 0.11 + 0.01 m/s Mean Velocity  MD: 0.01 + 0.01 m/s  30% 1RM  Peak Velocity r: 0.86; Bias: 0.158 m/s Mean Velocity  r: 0.44; Bias: 0.010 m/s  50% 1RM  Peak Velocity r: 0.76; Bias: 0.119 m/s Mean Velocity  r: -0.27; Bias: 0.012 m/s  70% 1RM  Peak Velocity r: 0.84; Bias: 0.091 m/s Mean Velocity  r: 0.29; Bias: 0.013 m/s  90% 1RM  Peak Velocity  r: 0.49; Bias: 0.055 m/s Mean Velocity  r: 0.60; Bias: 0.007 m/s | | Was a gold standard criterion used? YES / **NO**  Where the statistics/combination of used to validate the device appropriate? YES / **NO**  Did the original study claim the device was valid? **YES** / NO  Does this device validly measure what was measured? YES / **NO** |
| Janicijevic, García-Ramos [52] | GymAware | T-Force | F/W Back Squat | | 45% 1RM (Light), 65% 1RM (Medium), 85% 1RM (Heavy) | Mean Velocity,  Maximum Velocity | 20 | Mean Velocity r=0.995 P<0.001 Percent Differences = 4.42% Bias ± random error: -0.031 ± 0.019 m/s R2 (heteroscedasticity of errors): 0.097  Maximum Velocity r= 0.992 P<0.455 Percent Differences = 0.22% Bias ± random error: -0.003 ± 0.029 m/s R2 (heteroscedasticity of errors): 0.003 | | Was a gold standard criterion used? YES / **NO**  Where the statistics/combination of used to validate the device appropriate? YES / **NO**  Did the original study claim the device was valid? **YES** / NO  Does this device validly measure what was measured? YES / **NO** |
| Lopez-Torres, Fernandez-Elias [76] | ADR Encoder | T-Force and Speef4Lifts | S/M Bench Press | | 45%, 55%, 65%, 75%, 85% 1RM | Mean Propulsive Velocity | 17 | 45% 1RM T-Force r: 0.958, p<0.001 Speed4Lifts r: 0.927, p<0.001  55% 1RM T-Force r: 0.957, p<0.001 Speed4Lifts r:0.942, p<0.001  65% 1RM T-Force r: 0.879, p<0.001 Speed4Lifts r: 0.863, p<0.001  75% 1RM T-Force r: 0.892, p<0.001 Speed4Lifts r: 0.946, p<0.001  85% 1RM T-Force r: 0.905, p<0.001 Speed4Lifts r: 0.942, p<0.001  All Loads T-Force r: 0.992, p<0.001 Speed4Lifts r: 0.990, p<0.001 | | Was a gold standard criterion used? YES / **NO**  Where the statistics/combination of used to validate the device appropriate? YES / **NO**  Did the original study claim the device was valid? **YES** / NO  Does this device validly measure what was measured? YES / **NO** |
| Lorenzetti, Lamparter [10] | GymAware | Vicon 3D Motion Capture | F/W Back Squat, F/W Ballistic Squat | | F/W Back Squat: 70% 1RM, F/W Ballistic Squat: 25 kg | Mean Velocity,  Maximum Velocity,  Time to Maximum Velocity | 9 | F/W Back Squat70% 1RM  Mean Velocity RMSE: 0.064 m/s;  MD: 0.046 m/s  Maximum velocity RMSE: 0.163 m/s;  MD: 0.128 m/s  Time to maximum velocity RMSE: 0.042 s;  MD: 0.037 s  F/W Ballistic Squat 25 kg  Mean Velocity RMSE: 0.160 m/s;  MD: - 0.091 m/s  Maximum velocity RMSE: 0.304 m/s;  MD: 0.187 m/s  Time to maximum velocity RMSE: 0.046 s;  MD: 0.024 s | | Was a gold standard criterion used? **YES** / NO  Where the statistics/combination of used to validate the device appropriate? YES / **NO**  Did the original study claim the device was valid? **YES** / NO  Does this device validly measure what was measured? YES / **NO** |
|  | T-Force | Vicon 3D Motion Capture | F/W Back Squat, F/W Ballistic Squat | | F/W Back Squat: 70% 1RM, F/W Ballistic Squat: 25 kg | Mean Velocity, Maximum Velocity  Time to Maximum, Velocity | 9 | F/W Back Squat 70% 1RM  Mean Velocity RMSE: 0.070 m/s;  MD: 0.062 m/s  Maximum velocity RMSE: 0.151 m/s;  MD: 0.199 m/s  Time to maximum velocity RMSE: 0.026s;  MD: 0.010s  F/W Ballistic Squat 25 kg  Mean Velocity RMSE: 0.167 m/s;  MD: 0.102 m/s  Maximum velocity RMSE: 0.263 m/s;  MD: 0.150 m/s  Time to maximum Velocity RMSE: 0.045s;  MD: - 0.007s | | Was a gold standard criterion used? **YES** / NO  Where the statistics/combination of used to validate the device appropriate? YES / **NO**  Did the original study claim the device was valid? **YES** / NO  Does this device validly measure what was measured? YES / **NO** |
|  | Tendo | Vicon 3D Motion Capture | F/W Back Squat, F/W Ballistic Squat | | F/W Back Squat: 70% 1RM, F/W Ballistic Squat: 25 kg | Mean Velocity,  Maximum Velocity,  Time to Maximum Velocity | 9 | F/W Back Squat 70% 1RM  Mean Velocity RMSE: 0.046 m/s;  MD: 0.020 m/s  Maximum Velocity  RMSE: 0.194 m/s;  MD: 0.159 m/s  Time to maximum velocity RMSE: 0.041s;  MD: 0.031s  F/W Ballistic Squat 25 kg  Mean Velocity RMSE: 0.157 m/s;  MD: - 0.083 m/s  Maximum velocity RMSE: 0.315 m/s;  MD: 0.217 m/s  Time to maximum velocity RMSE: 0.064s;  MD: 0.046s | | Was a gold standard criterion used? **YES** / NO  Where the statistics/combination of used to validate the device appropriate? YES / **NO**  Did the original study claim the device was valid? **YES** / NO  Does this device validly measure what was measured? YES / **NO** |
| Lu, Zhang [46] | Full-Waveform Resistance Training Monitoring System  (FRTMS) | OptiTrack 3D  Motion Capture | S/M Back Squat | | 30%, 45%, 60%, 75%, 90% 1RM | Mean Velocity Eccentric Mean Velocity Full Waveform Velocity | 21 | Concentric Mean Velocity  30% 1RM r: 0.997 ICC (2,1): 0.999 RMSE: 0.009 m/s  45% 1RM r: 0.998 ICC (2,1): 0.999 RMSE: 0.010 m/s  60% 1RM r: 0.997 ICC (2,1): 0.998 RMSE: 0.010 m/s  75% 1RM r: 0.997 ICC (2,1): 0.999 RMSE: 0.005 m/s  90% 1RM r: 0.998 ICC (2,1): 1.000 RMSE: 0.005 m/s  All Loads Combined r: 0.998 ICC (2,1): 0.998  Eccentric Mean Velocity  30% 1RM r: 0.998 ICC (2,1): 0.999 RMSE: 0.007 m/s  45% 1RM r: 0.999 ICC (2,1): 0.999 RMSE: 0.007 m/s  60% 1RM r: 0.998 ICC (2,1): 0.999 RMSE: 0.008 m/s  75% 1RM r: 0.998 ICC (2,1): 0.999 RMSE: 0.007 m/s  90% 1RM r: 0.997 ICC (2,1): 1.000 RMSE: 0.005 m/s  All Loads Combined r: 0.997 ICC (2,1): 0.999 RMSE: 0.007  Full Waveform Velocity  30% 1RM r: 0.999 ICC (2,1): 1.000 RMSE: 0.021 m/s CMC: 1.000  45% 1RM r: 0.999 ICC (2,1): 0.999 RMSE: 0.023 m/s CMC: 0.999  60% 1RM r: 0.998 ICC (2,1): 0.999 RMSE: 0.028 m/s CMC: 0.999  75% 1RM r: 0.998 ICC (2,1): 0.999 RMSE: 0.024 m/s CMC: 0.999  90% 1RM r: 0.999 ICC (2,1): 0.999 RMSE: 0.017 m/s CMC: 0.999  All Loads Combined r: 0.999 ICC (2,1): 0.999 RMSE: 0.023 m/s CMC: 0.999 | | Was a gold standard criterion used? **YES** / NO  Where the statistics/combination of used to validate the device appropriate? **YES** / NO  Did the original study claim the device was valid? **YES** / NO  Does this device validly measure what was measured? **YES** / NO |
| Martínez-Cava, Hernández-Belmonte [70] | Speed4Lifts | T-Force | S/M Back Squat S/M Bench Press | | 25-95 kg | Peak Velocity, Mean Propulsive Velocity | 15 | S/M Back Squat 25-95 kg  Peak Velocity SEM: 0.02 m/s;  CV: 1.60%; ICC: 0.997  Mean Propulsive Velocity SEM: 0.03 m/s;  CV: 3.09%; ICC: 0.995 r: 0.9936;  SEE: 0.032 m/s   S/M Bench Press 25-95 kg  Peak Velocity SEM: 0.06 m/s; CV: 4.94%; ICC: 0.995   Mean Propulsive Velocity  SEM: 0.02 m/s;  CV: 2.72%; ICC: 0.999 r: 0.9985;  SEE: 0.024 m/s | | Was a gold standard criterion used? YES / **NO**  Where the statistics/combination of used to validate the device appropriate? **YES** / NO  Did the original study claim the device was valid? **YES** / NO  Does this device validly measure what was measured? YES / **NO** |
| McGrath, Flanagan [61] | Tendo | Eagle 3D Motion Capture Camera | F/W Bench Press | | 40%, 80% 1RM | Mean Velocity | 10 | 40% 1RM Mean Velocity Mean ± SD: 1.055±0.136 m/s CV: 12.95%  80% 1RM Mean Velocity Mean ± SD: 0.501±0.133 m/s CV: 26.55%  Combined Loads Mean Velocity Mean ± SD: 0.747±0.306 m/s CV: 40.90% R2: 0.985 ICC (95% CI): 0.977 (0.966-0.984) | | Was a gold standard criterion used? **YES** / NO  Where the statistics/combination of used to validate the device appropriate? YES / **NO**  Did the original study claim the device was valid? **YES** / NO  Does this device validly measure what was measured? YES / **NO** |
| Menrad and Edelmann-Nusser [53] | GymAware | Vicon (MX T20) 12 3D Motion Camera Setup | F/W Back Squat, F/W Standing Bent Over Row, F/W Deadlift | | 40%, 60%, 80% 1RM | Mean Velocity | 12 | F/W Back Squat  Mean Velocity R2: 0.9962 LoA: 0.050 m/s  F/W Barbell Row Mean Velocity R2: 0.9797 LoA: 0.133 m/s  F/W Deadlift  Mean Velocity R2: 0.9822 LoA: 0.113 m/s  All Exercises: Mean Velocity R2: 0.9825 LoA: 0.112 m/s | | Was a gold standard criterion used? **YES** / NO  Where the statistics/combination of used to validate the device appropriate? YES / **NO**  Did the original study claim the device was valid? **YES** / NO  Does this device validly measure what was measured? YES / **NO** |
| Mitter, Hölbling [54] | GymAware | Vicon 3D Motion Capture | F/W Back Squat, F/W Bench Press, F/W Deadlift | | 30-100% 1RM | Peak Velocity, Mean Velocity | 24 | F/W Back Squat  30-100% 1RM  Peak Velocity  SEE: 0.019 m/s  RMSE: 0.025 m/s Mean Velocity  SEE: 0.024 m/s RMSE: 0.035 m/s  F/W Bench Press  30-100% 1RM  Peak Velocity  SEE: 0.014 m/s;  RMSE: 0.017 m/s Mean Velocity  SEE: 0.030 m/s RMSE: 0.041 m/s  F/W Deadlift  30-100% 1RM  Peak Velocity  SEE: 0.017 m/s RMSE: 0.021 m/s Mean Velocity  SEE: 0.029 m/s RMSE: 0.029 m/s | | Was a gold standard criterion used? **YES** / NO  Where the statistics/combination of used to validate the device appropriate? YES / **NO**  Did the original study claim the device was valid? **YES** / NO  Does this device validly measure what was measured? YES / **NO** |
|  | FitroDyne  (fitronic) | Vicon 3D Motion Capture | F/W Back Squat, F/W Bench Press, F/W Deadlift | | 30-100% 1RM | Peak Velocity, Mean Velocity | 24 | F/W Back Squat  30-100% 1RM  Peak Velocity  SEE: 0.022 m/s  RMSE: 0.043 m/s Mean Velocity  SEE: 0.073 m/s  RMSE: 0.104 m/s  F/W Bench Press  30-100% 1RM  Peak Velocity  SEE: 0.019 m/s  RMSE: 0.022 m/s Mean Velocity  SEE: 0.100 m/s RMSE: 0.162 m/s  F/W Deadlift  30-100% 1RM  Peak Velocity  SEE: 0.018 m/s  RMSE: 0.035 m/s Mean Velocity  SEE: 0.084 m/s  RMSE: 0.182 m/s | | Was a gold standard criterion used? **YES** / NO  Where the statistics/combination of used to validate the device appropriate? YES / **NO**  Did the original study claim the device was valid? **YES** / NO  Does this device validly measure what was measured? YES / **NO** |
| Moreno-Villanueva, Rico-González [87] | ADR Encoder | T-Force | S/M Bench Press | | 5% to 100% 1RM | Mean Propulsive Velocity | 11 | With Pause Protocol  Zone 1 ADR 1 – T-Force 1 t: 22.534, p < 0.05 SEM: 0.0204 m/s, SDC: 0.057 m/s Bias ± SD: -0.0519 ± 0.01626, 95% Bias: -0.0928 to -0.01  Zone 1 ADR 1 – T-Force 2 t: 22.514, p < 0.05 SEM: 0.0186 m/s, SDC: 0.052 m/s Bias ± SD: -0.0467 ± 0.01474, 95% Bias: -0.0838 to -0.0095  Zone 2 ADR 1 – T-Force 1 t: 25.384, p < 0.001 SEM: 0.0090 m/s, SDC: 0.025 m/s Bias ± SD: -0.0487 ± 0.00557, 95% Bias: -0.0670 to -0.0303  Zone 3 ADR 1 – T-Force 1 t: 29.291, p < 0.001 SEM: 0.0031 m/s, SDC: 0.009 m/s Bias ± SD: -0.0291 ± 0.00203, 95% Bias: -0.0354 to -0.0228  Zone 4 ADR 1 – T-Force 1 t: 28.104, p < 0.001 SEM: 0.0024 m/s, SDC: 0.007 m/s Bias ± SD: -0.0196 ± 0.00179, 95% Bias: -0.0244 to -0.0147  No Pause Protocol  Zone 1 ADR 1 – T-Force 1 t: 26.901, p < 0.001 SEM: 0.0288 m/s, SDC: 0.080 m/s Bias ± SD: -0.1988 ± 0.02512, 95% Bias: -0.2562 to -0.1414  Zone 1 ADR 1 – T-Force 2 t: 13.633, p < 0.001 SEM: 0.0166 m/s, SDC: 0.046 m/s Bias ± SD: -0.2264 ± 0.01448, 95% Bias: -0.2595 to -0.1934  Zone 2 ADR 1 – T-Force 1 t: 28.908, p < 0.001 SEM: 0.0173 m/s, SDC: 0.048 m/s Bias ± SD: -0.1544 ± 0.01082, 95% Bias: -0.1894 to -0.1193  Zone 3 ADR 1 – T-Force 1 No p or t values provided for comparison in Zone 3 under no pause protocol.  Zone 4 ADR 1 – T-Force 1 No p or t values provided for comparison in Zone 4 under no pause protocol. | | Was a gold standard criterion used? YES / **NO**  Where the statistics/combination of used to validate the device appropriate? **YES** / NO  Did the original study claim the device was valid? YES / **NO**  Does this device validly measure what was measured? YES / **NO** |
| Pérez-Castilla, Piepoli [66] | T-Force | OptiTrack 3D Motion Capture | S/M Bench Press | | 45% to 85% 1RM | Mean Velocity | 14 | Bias: - 0.01 ± 0.03 m/s r=0.995 | | Was a gold standard criterion used? **YES** / NO  Where the statistics/combination of used to validate the device appropriate? YES / **NO**  Did the original study claim the device was valid? **YES** / NO  Does this device validly measure what was measured? YES / **NO** |
|  | Chronojump | OptiTrack 3D Motion Capture | S/M Bench Press | | 45% to 85% 1RM | Mean Velocity | 14 | Bias: - 0.03 ± 0.03 m/s r=0.991 | | Was a gold standard criterion used? **YES** / NO  Where the statistics/combination of used to validate the device appropriate? YES / **NO**  Did the original study claim the device was valid? **YES** / NO  Does this device validly measure what was measured? YES / **NO** |
|  | Speed4Lifts | OptiTrack 3D Motion Capture | S/M Bench Press | | 45% to 85% 1RM | Mean Velocity | 14 | Bias: - 0.04 ± 0.02 m/s r=0.994 | | Was a gold standard criterion used? **YES** / NO  Where the statistics/combination of used to validate the device appropriate? YES / **NO**  Did the original study claim the device was valid? **YES** / NO  Does this device validly measure what was measured? YES / **NO** |
| Pérez-Castilla, Miras-Moreno [73] | ADR Encoder | T-Force | S/M Bench Press | | 40%, 60%, 80% 1RM | Mean Velocity | 28 | No fixed or proportional bias was observed for any relative load.  40% 1RM Slope (95% CI): 0.895 (0.640, 1.150) Intercept (95% CI): 0.102 (-0.165, 0.369) R=0.808  60% 1RM Slope (95% CI): 1.017 (0.891, 1.142) Intercept (95% CI): -0.002 (-0.093, 0.090) R=0.956  80% 1RM Slope (95% CI): 0.996 (0.882, 1.110) Intercept (95% CI): 0.017 (-0.033, 0.067) R=0.964  All Loads Systematic Bias ± Random Error = 0.01 ± 0.03 m/s  R2= 0.103 | | Was a gold standard criterion used? YES / **NO**  Where the statistics/combination of used to validate the device appropriate? YES / **NO**  Did the original study claim the device was valid? **YES** / NO  Does this device validly measure what was measured? YES / **NO** |
| Rodriguez-Perea, Jerez-Mayorga [85] | Functional Electromechanical  Dynamometer (FEMD) | T-Force | N/A  Study was done over a 40 cm range of movement | | 0.4 m/s, 0.6 m/s, 0.8 m/s, 1.0 m/s, 1.2 m/s | Mean Velocity, Time to Reach Isokinetic Velocity (TRIV m/s), Time spent at Isokinetic Velocity (TSIV m/s) | 150 Trials | Mean Velocity   0.4 m/s Systematic Bias ± Random Error: 0.042 ± 0.000 m/s R2: 0.503  0.6 m/s Systematic Bias ± Random Error: 0.067 ± 0.001 m/s R2: 0.208  0.8 m/s Systematic Bias ± Random Error: 0.091 ± 0.006 m/s R2: 0.231  1.0 m/s Systematic Bias ± Random Error: 0.121 ± 0.006 m/s R2: 0.044  1.2 m/s Systematic Bias ± Random Error: 0.161 ± 0.060 m/s R2: 0.931 | | Was a gold standard criterion used? YES / **NO**  Where the statistics/combination of used to validate the device appropriate? YES / **NO**  Did the original study claim the device was valid? **YES** / NO  Does this device validly measure what was measured? YES / **NO** |
| Qu, Qian [86] | Jueying (Beijing, China) | GymAware | S/M Back Squat | | 30%, 45%, 60%, 75% 1RM | Mean Velocity, Peak Velocity | 12 | V_peak (m/s) 30% 1RM: Mean difference = 0.002, r = 0.999, ES = 0.004 45% 1RM: Mean difference = 0.006, r = 0.998, ES = 0.014 60% 1RM: Mean difference = 0.010, r = 0.991, ES = 0.033 75% 1RM: Mean difference = 0.012, r = 0.989, ES = 0.042  V_mean (m/s) 30% 1RM: Mean difference = -0.009, r = 0.986, ES = 0.045 45% 1RM: Mean difference = -0.018, r = 0.974, ES = 0.090 60% 1RM: Mean difference = -0.021, r = 0.896, ES = 0.189 75% 1RM: Mean difference = -0.019, r = 0.925, ES = 0.174  Linear Regression Analysis for Fixed and Proportional Bias V_peak (m/s): Fixed bias = 0.008 ± 0.005, Proportional bias = 1.047 (1.024–1.070) V_mean (m/s): Fixed bias = 0.017 ± 0.005, Proportional bias = 0.945 (0.900–0.991) | | Was a gold standard criterion used? YES / **NO**  Where the statistics/combination of used to validate the device appropriate? **YES** / NO  Did the original study claim the device was valid? **YES** / NO  Does this device validly measure what was measured? YES / **NO** |
| Suchomel, Techmanski [59] | Tendo | GymAware | Barbell Jump Shrug, Barbell Hang High Pull | 20%, 40%, 60%, 80%, 100% 1RM | Mean Velocity, Peak Velocity | 15 | Jump Shrug 20% Load R²: MV = 0.89, PV = 0.94 Slope: MV = 0.882 (0.767–0.998), PV = 1.007 (0.866–1.148) Intercept: MV = 0.074 (−0.124–0.272), PV = −0.128 (−0.550–0.293)  40% Load R²: MV = 0.90, PBV = 0.87 Slope: MV = 0.850 (0.674–1.027), PV = 1.024 (0.804–1.244) Intercept: MV = 0.123 (−0.153–0.398), PV = −0.181 (−0.759–0.396)  60% Load R²: MV = 0.88, PV = 0.91 Slope: MV = 0.977 (0.724–1.231), PV = 1.073 (0.929–1.218) Intercept: MV = −0.068 (−0.413–0.277), PV = −0.246 (−0.592–0.100)  80% Load R²: MV = 0.98, PV = 0.98 Slope: MV = 0.959 (0.877–1.040), PV = 0.988 (0.887–1.090) Intercept: MV = −0.016 (−0.108–0.076), PV = −0.010 (−0.220–0.201)  100% Load R²: MV = 0.95, PV = 0.96 Slope: MV = 0.920 (0.795–1.045), PV = 0.970 (0.855–1.086) Intercept: MV = 0.011 (−0.123–0.145), PV = 0.011 (−0.196–0.217)  Hang High Pull 20% Load R²: MV = 0.93, PV = 0.93 Slope: MV = 0.921 (0.755–1.087), PV = 1.215 (0.982–1.449) Intercept: MV = 0.033 (−0.283–0.350), PV = −0.785 (−1.522–−0.048) ‡  40% Load R²: MV = 0.93, PV = 0.79 Slope: MV = 0.982 (0.888–1.076), PV = 1.214 (0.959–1.470) Intercept: MV = −0.052 (−0.208–0.104), PV = −0.693 (−1.398–0.012)  60% Load R²: MV = 0.95, PV = 0.83 Slope: MV = 0.973 (0.819–1.127), PV = 1.064 (0.797–1.331) Intercept: MV = −0.013 (−0.253–0.227), PV = −0.217 (−0.849–0.415)  80% Load R²: MV = 0.96, PV = 0.94 Slope: MV = 0.978 (0.890–1.066), PV = 1.094 (0.898–1.290) Intercept: MV = −0.017 (−0.122–0.088), PV = −0.220 (−0.630–0.189)  100% Load R²: MV = 0.96, PV = 0.91 Slope: MV = 0.886 (0.791–0.981), PV = 1.029 (0.842–1.216) Intercept: MV = 0.082 (−0.022–0.187), PV = −0.092 (−0.457–0.273) | | Was a gold standard criterion used? YES / **NO**  Where the statistics/combination of used to validate the device appropriate? YES / **NO**  Did the original study claim the device was valid? **YES** / NO  Does this device validly measure what was measured? YES / **NO** | |
| Thompson, Rogerson [55] | GymAware | Raptor  3D Motion Capture 12 Camera System | F/W Back Squat Power Clean | 40%, 50%, 60%, 70%, 80%, 90%, 100% 1RM | Mean Velocity, Peak Velocity | 11 | F/W Back Squat   40% 1RM  Mean Velocity  R2: 0.95; LoA: - 0.02 ± 0.05 m/s Peak Velocity  R2: 0.97; LoA: 0.01 ± 0.07 m/s  50% 1RM  Mean Velocity  R2: 0.95; LoA: - 0.01 ± 0.04 m/s Peak Velocity  R2: 0.98; LoA: 0.01 ± 0.06 m/s  60% 1RM  Mean Velocity  R2: 0.98; LoA: - 0.02 ± 0.02 m/s Peak Velocity  R2: 0.99; LoA: 0.01 ± 0.04 m/s  70% 1RM  Mean Velocity  R2: 0.97; LoA: - 0.01 ± 0.03 m/s Peak Velocity  R2: 0.99; LoA: 0.01 ± 0.05 m/s  80% 1RM  Mean Velocity R2: 0.99; LoA: - 0.01 ± 0.02 m/s Peak Velocity  R2: 0.99; LoA: 0.02 ± 0.05 m/s  90% 1RM  Mean Velocity  R2: 0.99; LoA: - 0.01 ± 0.01 m/s Peak Velocity  R2: 0.96; LoA: 0.02 ± 0.10 m/s  100% 1RM  Mean Velocity  R2: 0.97; LoA: - 0.02 ± 0.04 m/s Peak Velocity  R2: 0.97; LoA: 0.01 ± 0.08 m/s  Full  Mean Velocity  R2: 0.99; LoA: - 0.01 ± 0.03 m/s Peak Velocity  R2: 0.99; LoA: 0.01 ± 0.06 m/s  Power Clean   40% 1RM  Mean Velocity  R2: 0.93; LoA: - 0.03 ± 0.08 m/s Peak Velocity  R2: 0.91; LoA: - 0.01 ± 0.14 m/s  50% 1RM  Mean Velocity R2: 0.95; LoA: - 0.03 ± 0.05 m/s Peak Velocity  R2: 0.93; LoA: - 0.01 ± 0.12 m/s  60% 1RM  Mean Velocity  R2: 0.95; LoA: - 0.03 ± 0.05 m/s Peak Velocity  R2: 0.95; LoA: - 0.01 ± 0.10 m/s  70% 1RM  Mean Velocity  R2: 0.78; LoA: - 0.02 ± 0.07 m/s Peak Velocity  R2: 0.95; LoA: 0.00 ± 0.08 m/s  80% 1RM  Mean Velocity R2: 0.86; LoA: - 0.02 ± 0.05 m/s Peak Velocity  R2: 0.91; LoA: 0.00 ± 0.10 m/s  90% 1RM  Mean Velocity  R2: 0.42; LoA: 0.00 ± 0.11 m/s Peak Velocity  R2: 0.86; LoA: 0.01 ± 0.11 m/s  100% 1RM  Mean Velocity  R2: 0.64; LoA: - 0.01 ± 0.07 m/s Peak Velocity  R2: 0.86; LoA: 0.08 ± 0.11 m/s  Full Mean Velocity  R2: 0.94; LoA: - 0.02 ± 0.07 m/s Peak Velocity  R2: 0.96; LoA: 0.00 ± 0.11 m/s | | Was a gold standard criterion used? **YES** / NO  Where the statistics/combination of used to validate the device appropriate? YES / **NO**  Did the original study claim the device was valid? **YES** / NO  Does this device validly measure what was measured? YES / **NO** | |

*Abbreviations: 1RM: One Repetition Maximum, CMC: Coefficient of Multiple Correlation, CI: Confidence Interval, CV%: Coefficient of Variation Percent, ES: Effect Size, F/W: Free Weight, Hz: Hertz, ICC: Intraclass Correlation Coefficient, kg: Kilograms, LoA: Limits of Agreement, m/s: Meters per Second, MD: Mean Difference, N: Newton, P: Probability Value, R2: Coefficient of Determination, r: Pearson correlation coefficient, RMSE: Root Mean Square Error, S/M: Smith Machine, SDC: Smallest Detectable Change, SD: Standard Deviation, SEE: Standard Error of Estimate, SEM: Standard Error of Measurement, t: t-test statistic, W: Watt..*

S8 Table: Studies investigating the validity of an IMU/Accelerometer device.

| **Study** | **Device/s** | **Criterion** | **Exercise/s** | **Intensity/Load** | **Variable/s Measured** | **Sample Size** | **Reported Statistics** | **Validity Criteria** |
| --- | --- | --- | --- | --- | --- | --- | --- | --- |
| Abbott et al. (1) | Bar Sensei | Vicon (nexus 1.8.5) 3D Motion Capture | F/W Back Squat | 20%, 30%, 40%, 50%, 60%, 70%, 80%, 90%, 100% 1RM | Peak Velocity, Mean Velocity, Mean Propulsive Velocity | 16 | 20% 1RM  Peak Velocity  SEE: 0.06 m/s Mean Velocity  SEE: 0.05 m/s Mean Propulsive Velocity  SEE: 0.11 m/s  30% 1RM  Peak Velocity  SEE: 0.05 m/s Mean Velocity  SEE: 0.04 m/s Mean Propulsive Velocity  SEE: 0.08 m/s  40% 1RM  Peak Velocity  SEE: 0.05 m/s Mean Velocity  SEE: 0.05 m/s Mean Propulsive Velocity  SEE: 0.07 m/s  50% 1RM  Peak Velocity  SEE: 0.06 m/s Mean Velocity  SEE: 0.05 m/s Mean Propulsive Velocity  SEE: 0.10 m/s  60% 1RM  Peak Velocity  SEE: 0.09 m/s Mean Velocity  SEE: 0.04 m/s Mean Propulsive Velocity  SEE: 0.06 m/s  70% 1RM  Peak Velocity  SEE: 0.14 m/s Mean Velocity  SEE: 0.04 m/s Mean Propulsive Velocity  SEE: 0.07 m/s  80% 1RM  Peak Velocity  SEE: 0.17 m/s Mean Velocity  SEE: 0.05 m/s Mean Propulsive Velocity  SEE: 0.05 m/s  90% 1RM  Peak Velocity  SEE: 0.19 m/s Mean Velocity  SEE: 0.04 m/s Mean Propulsive Velocity  SEE: 0.04 m/s  100% 1RM  Peak Velocity  SEE: 0.16 m/s Mean Velocity  SEE: 0.04 m/s Mean Propulsive Velocity  SEE: 0.03 m/s | Was a gold standard criterion used? **YES** / NO  Where the statistics/combination of used to validate the device appropriate? YES / **NO**  Did the original study claim the device was valid? YES / **NO**  Does this device validly measure what was measured? YES / **NO** |
| Balsalobre-Fernández, Kuzdub [89] | Push Band | T-Force | S/M Back Squat | 20-70 kg | Peak Velocity, Mean Velocity | 10 | Peak Velocity  r: 0.91; SEE: 0.1 m/s; MD: - 0.07 ± 0.1 m/s  Mean Velocity r: 0.86; SEE: 0.08 m/s; MD: 0.11 ± 0.1 m/s | Was a gold standard criterion used? YES / **NO**  Where the statistics/combination of used to validate the device appropriate? **YES** / NO  Did the original study claim the device was valid? **YES** / NO  Does this device validly measure what was measured? YES / **NO** |
| Balsalobre-Fernández, Marchante [78] | Beast Sensor (wrist) & Beast Sensor (barbell) | SmartCoach Power Encoder | F/W Back Squat, F/W Bench Press, F/W Hip Thrust | 50-95% 1RM | Mean Velocity | 10 | Beast Sensor (wrist):  F/W Back Squat  50-95% 1RM  Mean Velocity  Bias: 0.03 ± 0.06 m/s;  SEE: 0.06 m/s   F/W Bench Press  50-95% 1RM Mean Velocity  Bias: 0.009 ± 0.04 m/s; SEE: 0.04 m/s   F/W Hip-Thrust  50-95% 1RM  Mean Velocity  Bias: 0.06 ± 0.07 m/s; SEE: 0.07 m/s   Beast Sensor (barbell):  F/W Back Squat  50-95% 1RM  Mean Velocity  Bias: - 0.003 ± 0.05 m/s; SEE: 0.05 m/s   F/W Bench Press  50-95% 1RM  Mean Velocity  Bias: 0.04 ± 0.05 m/s; SEE: 0.05 m/s   F/W Hip-Thrust  50-95% 1RM  Mean Velocity  Bias: 0.03 ± 0.05 m/s; SEE: 0.04 m/s | Was a gold standard criterion used? YES / **NO**  Where the statistics/combination of used to validate the device appropriate? YES / **NO**  Did the original study claim the device was valid? **YES** / NO  Does this device validly measure what was measured? YES / **NO** |
| Banyard, Nosaka [3] | Push Band | 4 x Celesco PT5A-250 LPT | F/W Back Squat | 20%, 40%, 60%, 70%, 80%, 90%, 100% 1RM | Mean Velocity, Peak Velocity | 10 | 20% 1RM  Mean Velocity r: 0.86; CV: 6.9%; ES: 0.24; SEE: 0.08 m/s Peak Velocity r: 0.80; CV: 8.1%; ES: 0.03; SEE: 0.16 m/s  40% 1RM  Mean Velocity r: 0.80; CV: 8.2%; ES: 0.18; SEE: 0.08 m/s Peak Velocity r: 0.76; CV: 9.6%; ES: - 0.16; SEE: 0.15 m/s  60% 1RM  Mean Velocity r: 0.76; CV: 8.4%; ES: 0.49; SEE: 0.06 m/s Peak Velocity r: 0.74; CV: 10%; ES: - 0.22; SEE: 0.13 m/s Mean Force r: 0.96; CV: 5%; ES: 1.2; SEE: 87.53N Peak Force r: 0.97; CV: 5%; ES: 0.09; SEE: 117.37N  80% 1RM  Mean Velocity r: 0.73; CV: 10.7%; ES: 0.54; SEE: 0.05 m/s Peak Velocity r: 0.68; CV: 13.2%; ES: - 0.29; SEE: 0.14 m/s  90% 1RM  Mean Velocity r: 0.65; CV: 11.8%; ES: 0.69; SEE: 0.04 m/s Peak Velocity r: 0.65; CV: 12.9%; ES: - 0.52; SEE: 0.12 m/s  100% 1RM  Mean Velocity r: 0.33; CV: 27.2%; ES: 1.62; SEE: 0.05 m/s Peak Velocity r: 0.49; CV: 18.4%; ES: - 0.13; SEE: 0.13 m/s | Was a gold standard criterion used? YES / **NO**  Where the statistics/combination of used to validate the device appropriate? **YES** / NO  Did the original study claim the device was valid? YES / **NO**  Does this device validly measure what was measured? YES / **NO** |
| Pelaez Barrajon and San Juan [99] | Accelerometer mobile basic program (MBP) via Huawei G620S smartphone | Speed4Lifts | F/W Bench Press | 70%, 90%, 100% 1RM | Mean Velocity, | 10 | r = 0.54,  p < 0.001 SEE = 0.13 m/s,  OP = 0.83 | Was a gold standard criterion used? YES / **NO**  Where the statistics/combination of used to validate the device appropriate? **YES** / NO  Did the original study claim the device was valid? YES / **NO**  Does this device validly measure what was measured? YES / **NO** |
| Beckham, Layne [56] | Bar Sensei | GymAware | F/W Back Squat | 45%, 60%, 75% 1RM | Mean Velocity, Peak Velocity | 16 | 45% 1RM  Mean Velocity  ICC: 0.482 MD: - 0.106 m/s Peak Velocity  ICC: 0.555 MD: 0.009 m/s  60% 1RM  Mean Velocity  ICC: 0.303 MD: - 0.094 m/s Peak Velocity  ICC: 0.362 MD: - 0.037 m/s  75% 1RM  Mean Velocity  ICC: 0.329 MD: - 0.081 m/s Peak Velocity  ICC: 0.361 MD: - 0.099 m/s | Was a gold standard criterion used? YES / **NO**  Where the statistics/combination of used to validate the device appropriate? YES / **NO**  Did the original study claim the device was valid? YES / **NO**  Does this device validly measure what was measured? YES / **NO** |
| Callaghan, Guy [83] | Push Band 2.0 | Vicon (Vero 2.2) 3D Motion Capture | F/W Back Squat, F/W Front Squat,  F/W Bench Press | Loads  20% ,40%, 60%, 80% 1RM  Velocities Fast (>1.00 m/s), Moderate (0.65 to 1.00 m/s), Slow (<0.65 m/s) | Mean Velocity | 20 | F/W Back Squat Fast (>1.00 m/s)  Cusum Linearity Test: 0.73 p-value: 0.19 Intercept (A): 0.03 95% CI of Intercept: ± 0.35 Slope (B): 0.84 95% CI of Slope: 0.70 ± 0.99 Fixed Bias: Yes Proportional Bias: Yes Agreement: No Moderate (0.65 to 1.00 m/s)  Cusum Linearity Test: 0.33 p-value: 0.07 Intercept (A): 0.00 95% CI of Intercept: ± 0.15 Slope (B): 0.94 95% CI of Slope: 0.84 ± 1.03 Fixed Bias: No Proportional Bias: No Agreement: Yes Slow (<0.65 m/s)  Cusum Linearity Test: 0.39 p-value: −0.01 Intercept (A): −0.14 95% CI of Intercept: ± 0.09 Slope (B): 1.03 95% CI of Slope: 0.84 ± 1.29 Fixed Bias: No Proportional Bias: No Agreement: Yes F/W Front Squat Fast (>1.00 m/s)  Cusum Linearity Test: 0.21 p-value: 0.17 Intercept (A): 0.06 95% CI of Intercept: ± 0.30 Slope (B): 0.84 95% CI of Slope: 0.73 ± 0.94 Fixed Bias: Yes Proportional Bias: Yes Agreement: No Moderate (0.65 to 1.00 m/s)  Cusum Linearity Test: 0.86 p-value: 0.08 Intercept (A): 0.04 95% CI of Intercept: ± 0.12 Slope (B): 0.91 95% CI of Slope: 0.86 ± 0.96 Fixed Bias: Yes Proportional Bias: Yes Agreement: No Slow (<0.65 m/s)  Cusum Linearity Test: 0.26 p-value: 0.03 Intercept (A): −0.12 95% CI of Intercept: ± 0.12 Slope (B): 0.95 95% CI of Slope: 0.78 ± 1.20 Fixed Bias: No Proportional Bias: No Agreement: Yes F/W Bench Press Fast (>1.00 m/s)  Cusum Linearity Test: 0.55 p-value: 0.11 Intercept (A): 0.02 95% CI of Intercept: ± 0.20 Slope (B): 0.91 95% CI of Slope: 0.83 ± 0.98 Fixed Bias: Yes Proportional Bias: Yes Agreement: No Moderate (0.65 to 1.00 m/s)  Cusum Linearity Test: 0.01 p-value: (not provided) Intercept (A): (not provided) 95% CI of Intercept: (not provided) Slope (B): (not provided) 95% CI of Slope: (not provided) Fixed Bias: (not provided) Proportional Bias: No Agreement: No Slow (<0.65 m/s)  Cusum Linearity Test: 0.63 p-value: 0.01 Intercept (A): −0.03 95% CI of Intercept: ± 0.04 Slope (B): 0.96 95% CI of Slope: 0.90 ± 1.03 Fixed Bias: No Proportional Bias: No Agreement: Yes | Was a gold standard criterion used? **YES** / NO  Where the statistics/combination of used to validate the device appropriate? **YES** / NO  Did the original study claim the device was valid? **Back Squat (slow to moderate velocities): Yes Back Squat (fast velocities): No Bench Press (slow velocities): Yes Bench Press (moderate to fast velocities): No Front Squat (slow velocities): Yes Front Squat (moderate to fast velocities): No**  Does this device validly measure what was measured? YES / **NO** |
| Chéry and Ruf [62] | Push Band | GymAware | F/W Deadlift | 20%, 40%, 60%, 80%, 90%, 100%, All Loads | Mean Velocity, Peak Velocity | 10 | 20% 1RM  Mean Velocity CV: 11.21%;  MD: 0.05 m/s Peak Velocity CV: 10.63%;  MD: 0.12 m/s   40% 1RM  Mean Velocity CV: 17.51%;  MD:- 0.10 m/s  Peak Velocity CV: 15.23%;  MD:- 0.16 m/s  60% 1RM  Mean Velocity CV: 9.51%;  MD: 0.02 m/s  Peak Velocity CV: 10.34%;  MD: 0.02 m/s   80% 1RM  Mean Velocity CV: 16.02%;  MD: 0.13 m/s Peak Velocity CV: 11.76%;  MD: 0.18 m/s  90% 1RM  Mean Velocity CV: 14.23%;  MD: 0.10 m/s  Peak Velocity  CV: 8.15%;  MD: 0.12 m/s   100% 1RM  Mean Velocity CV: 35.00%;  MD: 0.08 m/s  Peak Velocity CV: 33.34%;  MD: 0.13 m/s   All Loads Mean Velocity CV: 22.69%;  MD: 0.05 m/s  Peak Velocity CV: 20.35%;  MD: 0.07 m/s | Was a gold standard criterion used? YES / **NO**  Where the statistics/combination of used to validate the device appropriate? YES / **NO**  Did the original study claim the device was valid? YES / **NO**  Does this device validly measure what was measured? YES / **NO** |
| Courel-Ibáñez, Martínez-Cava [68] | Push Band | T-Force | S/M Bench Press, S/M Back Squat | 20 kg, 30 kg, 40 kg, 50 kg, 60 kg,70 kg, 80 kg | Mean Velocity,  Peak Velocity | 17 | S/M Bench Press Mean Velocity  SEM: 0.13 m/s;  CV: 18.3%;  ICC: 0.928   PEAK velocity SEM: 0.23 m/s;  CV: 17.1%;  ICC: 0.937   S/M Back Squat Mean Velocity  SEM: 0.07 m/s;  CV: 8.8%;  ICC: 0.941   PEAK velocity SEM: 0.10 m/s;  CV: 6.4%;  ICC: 0.952 | Was a gold standard criterion used? YES / **NO**  Where the statistics/combination of used to validate the device appropriate? **YES** / NO  Did the original study claim the device was valid? **YES** / NO  Does this device validly measure what was measured? YES / **NO** |
| Dragutinovic, Jacobs [93] | VmaxPro | Vicon 3D Motion Capture  (MoCap) | F/W Bench Press, F/W Back Squat | 30%, 50%, 70%, 90% 1RM | Mean Velocity | 17 | F/W Bench Press  L0 ( kg): VmaxPro: 102.98 ± 17.38 MoCap: 101.58 ± 17.18 Correlation (r): 0.925 (p ≤ 0.001) R2: 0.856 (p ≤ 0.001) MAPE (%): 4.46 ± 4.40  v0 (m/s): VmaxPro: 1.66 ± 0.22 MoCap: 1.65 ± 0.23 Correlation (r): 0.808 (p ≤ 0.001) R2: 0.652 (p ≤ 0.001) MAPE (%): 6.01 ± 5.07  Aline (m/s· kg): VmaxPro: 52.32 ± 8.66 MoCap: 51.61 ± 8.56 Correlation (r): 0.927 (p ≤ 0.001) R2: 0.859 (p ≤ 0.001) MAPE (%): 4.30 ± 4.30  1RM ( kg): VmaxPro: 95.12 ± 17.58 MoCap: 93.60 ± 16.90 Correlation (r): 0.942 (p ≤ 0.001) R2: 0.887 (p ≤ 0.001) MAPE (%): 4.06 ± 4.31  F/W Back Squat  L0 ( kg): VmaxPro: 182.76 ± 70.71 MoCap: 165.87 ± 31.69 Correlation (r): 0.740 (p ≤ 0.001) R2: 0.548 (p ≤ 0.001) MAPE (%): 10.65 ± 16.18  v0 (m/s): VmaxPro: 1.32 ± 0.20 MoCap: 1.35 ± 0.14 Correlation (r): 0.615 (p ≤ 0.001) R2: 0.378 (p ≤ 0.001) MAPE (%): 7.00 ± 7.18  Aline (m/s· kg): VmaxPro: 92.04 ± 35.30 MoCap: 83.61 ± 15.84 Correlation (r): 0.741 (p ≤ 0.001) R2: 0.549 (p ≤ 0.001) MAPE (%): 10.51 ± 15.86  1RM ( kg): VmaxPro: 142.96 ± 42.59 MoCap: 133.09 ± 26.25 Correlation (r): 0.627 (p ≤ 0.001) R2: 0.393 (p ≤ 0.001) MAPE (%): 9.48 ± 14.30​​. | Was a gold standard criterion used? **YES** / NO  Where the statistics/combination of used to validate the device appropriate? **YES** / NO  Did the original study claim the device was valid? **YES** / NO  Does this device validly measure what was measured? YES / **NO** |
| Feuerbacher, Jacobs [74] | VmaxPro | Vicon 3D Motion Capture (MoCap) and T-Force | F/W Back Squat | 30%, 50%, 70%,  90% 1RM | Mean Velocity | 19 | 30%RM Difference (VmaxPro - Vicon): 0.07 ± 0.13 Difference (VmaxPro - T-force): 0.07 ± 0.08 MARD (VmaxPro vs. Vicon): 7.6 ± 14.4 MARD (VmaxPro vs. T-force): 5.6 ± 11.5 Correlation (VmaxPro vs. Vicon): 0.401 (p < 0.001) Correlation (VmaxPro vs. T-force): 0.407 (p < 0.001)  50%RM Difference (VmaxPro - Vicon): 0.06 ± 0.06 Difference (VmaxPro - T-force): 0.06 ± 0.06 MARD (VmaxPro vs. Vicon): 7.1 ± 7.4 MARD (VmaxPro vs. T-force): 7.4 ± 7.6 Correlation (VmaxPro vs. Vicon): 0.743 (p < 0.001) Correlation (VmaxPro vs. T-force): 0.744 (p < 0.001)  70%RM Difference (VmaxPro - Vicon): 0.04 ± 0.07 Difference (VmaxPro - T-force): 0.04 ± 0.05 MARD (VmaxPro vs. Vicon): 7.0 ± 12.3 MARD (VmaxPro vs. T-force): 7.1 ± 10.6 Correlation (VmaxPro vs. Vicon): 0.559 (p < 0.001) Correlation (VmaxPro vs. T-force): 0.720 (p < 0.001)  90%RM Difference (VmaxPro - Vicon): 0.03 ± 0.07 Difference (VmaxPro - T-force): 0.04 ± 0.04 MARD (VmaxPro vs. Vicon): 6.0 ± 10.7 MARD (VmaxPro vs. T-force): 6.0 ± 14.4 Correlation (VmaxPro vs. Vicon): 0.827 (p < 0.001) Correlation (VmaxPro vs. T-force): 0.828 (p < 0.001)  MARD = mean absolute relative difference. | Was a gold standard criterion used? **YES** / NO  Where the statistics/combination of used to validate the device appropriate? **YES** / NO  Did the original study claim the device was valid? **YES** / NO  Does this device validly measure what was measured? YES / **NO** |
| Fritschi, Seiler [51] | VmaxPro | Vantage 5, Vicon 3D Motion Capture | Hang Power Snatch,  CMJ, Squat Jump,  F/W Back Squat | Hang power snatch 20 kg,  CMJ 50% of the load determined for moderate back squat (mean ± standard deviation: 34 ± 10 kg),  Squat Jump 50% of the load determined for moderate back squat (mean ±  standard deviation: 34 ±10 kg),  F/W back squat (Moderate) = (65 ± 20 kg) to elicit Vmean in the range of 0.7-0.8 m/s (mean ± standard deviation of actual values: 0.75 ±  0.05 m/s),  F/W back squat (Heavy) = Heavy back squat with the individual load determined during warm-up (90 ± 20 kg) to elicit Vmean of just under 0.5 m/s (actual values: 0.47 ± 0.05 m/s) | Mean Velocity,  Peak Velocity | 14 | Mean Velocity r: 0.99 (0.94-0.96)  SEE (m/s): 0.08 (0.02-0.13)  SEEpct (%): 7.0 (2.4-6.8)  Peak Velocity r: 0.99 (0.92-0.99) SEE (m/s): 0.11 (0.07-0.08)  SEEpct (%): 5.2 (2.4-6.6) | Was a gold standard criterion used? **YES** / NO  Where the statistics/combination of used to validate the device appropriate? YES / **NO**  Did the original study claim the device was valid? **YES** / NO  Does this device validly measure what was measured? YES / **NO** |
|  | Push Band | Vantage 5, Vicon 3D Motion Capture | Hang Power Snatch, CMJ, Squat Jump, F/W Back Squat | Hang power snatch 20 kg,  CMJ 50% of the load determined for moderate back squat (mean ± standard deviation: 34 ± 10 kg),  Squat Jump 50% of the load determined for moderate back squat (mean ±  standard deviation: 34 ±10 kg),  F/W back squat (Moderate) = (65 ± 20 kg) to elicit Vmean in the range of 0.7-0.8 m/s (mean ± standard deviation of actual values: 0.75 ±  0.05 m/s),  F/W back squat (Heavy) = Heavy back squat with the individual load determined during warm-up (90 ± 20 kg) to elicit Vmean of just under 0.5 m/s (actual values: 0.47 ± 0.05 m/s) | Mean Velocity, Peak Velocity | 14 | Mean Velocity r: 0.97 (0.69-0.90)  SEE (m/s): 0.12 (0.03-0.17) SEEpct (%): 11.0 (5.0-9.1)  Peak Velocity r: 0.98 (0.79-0.96) SEE (m/s): 0.15 (0.12-0.16) SEEpct (%): 7.1 (4.7-11.0) | Was a gold standard criterion used? **YES** / NO  Where the statistics/combination of used to validate the device appropriate? YES / **NO**  Did the original study claim the device was valid? **YES** / NO  Does this device validly measure what was measured? YES / **NO** |
| García-Pinillos, Latorre-Román [69] | WIMU System | T-Force | S/M Concentric-Only Half ROM Back Squat | 10-100% 1RM | Mean Velocity, Mean Propulsive Velocity | 19 | 10-100% 1RM  Mean Velocity Bias: 0.02 ± 0.06 m/s Mean propulsive velocity Bias: 0.06 ± 0.07 m/s Maximum velocity Bias: 0.16 ± 0.16 m/s  10% 1RM  Mean Velocity r: 0.865 Mean propulsive velocity r: 0.898 Maximum velocity r: 0.971  20% 1RM  Mean Velocity  r: 0.520 Mean propulsive velocity r: 0.398 Maximum velocity  r: 0.773  30% 1RM  Mean Velocity r: 0.696 Mean propulsive velocity r: 0.813 Maximum velocity  r: 0.196  40% 1RM  Mean Velocity  r: 0.877 Mean propulsive velocity r: 0.882 Maximum velocity  r: 0.842  50% 1RM  Mean Velocity  r: 0.760 Mean propulsive velocity r: 0.823 Maximum velocity  r: 0.908  60% 1RM  Mean Velocity  r: 0.646 Mean propulsive velocity r: 0.645 Maximum velocity  r: 0.729  70% 1RM  Mean Velocity  r: 0.419 Mean Propulsive Velocity  r: 0.628 Maximum velocity  r: 0.819  80% 1RM  Mean Velocity  r: 0.662 Mean Propulsive Velocity  r: 0.632 Maximum velocity  r: 0.498  90% 1RM  Mean Velocity  r: 0.739 Mean Propulsive Velocity  r: 0.717 Maximum velocity  r: 0.742  100% 1RM  Mean Velocity  r: 0.687 Mean Propulsive Velocity  r: 0.685 Maximum velocity  r: 0.861 | Was a gold standard criterion used? YES / **NO**  Where the statistics/combination of used to validate the device appropriate? YES / **NO**  Did the original study claim the device was valid? YES / **NO**  Does this device validly measure what was measured? YES / **NO** |
| Gilic, Gabrilo [90] | Push Band 2.0 | Broad Jump Test | Hex Bar Deadlift | 45%, 55%, 75% 1RM | Mean Velocity, Peak Velocity | 16 | Mean Velocity at 45% 1RM  r: 0.73  p: 0.001 Peak Velocity at 45% 1RM  r: 0.76  p: 0.001 | Was a gold standard criterion used? YES / **NO**  Where the statistics/combination of used to validate the device appropriate? YES / **NO**  Did the original study claim the device was valid? **YES** / **NO**  Does this device validly measure what was measured? YES / **NO** |
| Gomez-Piriz, Sanchez [75] | Myotest | T-Force | F/W Bench Press | 25 kg | Maximum Velocity | 3 | Subject 1: Max. Strength (D-W: 0.700, Interval: 0.810–1.580, b1: 0.010, b2: 0.140, t: 0.450, df: 10, p: 0.663), Max. Velocity (D-W: 1.250, b1: -20.010, b2: -20.040, t: -20.120, df: 10, p: 0.904).   Subject 2: Max. Strength (D-W: 1.490, Interval: 0.810–1.580, b1: -20.140, b2: -20.320, t: -21.080, df: 10, p: 0.304), Max. Velocity (D-W: 2.060, b1: -20.040, b2: -20.310, t: -21.020, df: 10, p: 0.333).   Subject 3: Max. Strength (D-W: 1.580, Interval: 0.980–1.540, b1: 0.020, b2: 0.010, t: 0.037, df: 14, p: 0.971), Max. Velocity (D-W: 2.130, b1: 0.320, b2: 0.670, t: 3.350, df: 14, p: 0.005). | Was a gold standard criterion used? YES / **NO**  Where the statistics/combination of used to validate the device appropriate? YES / **NO**  Did the original study claim the device was valid? YES / **NO**  Does this device validly measure what was measured? YES / **NO** |
| Held, Rappelt [84] | VmaxPro | Speed4Lifts | F/W Back Squat, F/W Hip Thrust | 75% 1RM | Mean Velocity, Barbell Displacement | 19 | F/W back squat  Mean Concentric Velocity (m/s) TE%; 5.6 CV%; 7.6 ICC (95% CI); 0.94 (0.92-0.95 CC (95% CI); 0.96 (0.95-0.96)  LoA; 0.1  SEM; 0.01  Barbell Displacement (cm) TE%; 5.9 CV%; 5.9 ICC (95% CI); 0.88 (0.43-0.95 CC (95% CI); 0.84 (0.83-0.85)  LoA; 10.69 SEM; 1.18  F/W hip thrust   Mean Concentric Velocity (m/s) TE%; 9.2 CV %; 11.2 ICC (95% CI); 0.85 (0.70-0.92 CC (95% CI); 0.91 (0.91-0.92)  LoA; 0.12 SEM; 0.02  Barbell Displacement (cm) TE%; 7.2 CV%; 10.2 ICC (95% CI); 0.76 (0.73-0.78) CC (95% CI); 0.76 (0.74-0.78)  LoA; 7.78 SEM; 1.64 | Was a gold standard criterion used? YES / **NO**  Where the statistics/combination of used to validate the device appropriate? **YES** / NO  Did the original study claim the device was valid? **YES** / NO  Does this device validly measure what was measured? YES / **NO** |
| Jovanovic and Jukic [57] | Push Band 2.0 | GymAware | Hex Bar Deadlift | 40%, 60%, 80%, 90%, 100% 1RM | Peak Velocity, Mean Velocity | 12 | High correlations observed between PUSH2 and GYM (r = 0.915–0.948). Fixed bias: Unit 1 MV intercept = 0.045 m/s (95% CI (0.025–0.066)), PV intercept = 0.179 m/s (95% CI (0.151–0.207)); Unit 2 MV intercept = -0.044 m/s (95% CI (-0.061 to -0.026)), PV intercept = 0.048 m/s (95% CI (0.023–0.070)). Proportional bias: Unit 1 MV slope = 0.850 (95% CI (0.806–0.887)), PV slope = 0.813 (95% CI (0.784–0.84)); Unit 2 MV slope = 0.943 (95% CI (0.913–0.973)), PV slope = 0.871 (95% CI (0.849–0.894)). SDC = 0.167–0.264 m/s exceeds SESOI limits. SDC% 1RM = 11.780–11.892%, indicating poor between-units agreement with GYM as criterion. | Was a gold standard criterion used? YES / **NO**  Where the statistics/combination of used to validate the device appropriate? **YES** / NO  Did the original study claim the device was valid? **YES** / NO  Does this device validly measure what was measured? YES / **NO** |
| Lake, Augustus [91] | Push Band 2.0 | Vicon T40S 3D Motion Capture | F/W Bench Press | 60%, 90% 1RM | Peak Velocity, Mean Velocity | 14 | 60% 1RM  Peak Velocity  MD: - 0.039 m/s LPR: 0.907 Mean Velocity  MD: - 0.065 m/s  LPR: 0.797  90% 1RM  Peak Velocity  MD: - 0.063 m/s LPR: 1.110 Mean Velocity  MD: - 0.038 m/s  LPR: 0.816 | Was a gold standard criterion used? **YES** / NO  Where the statistics/combination of used to validate the device appropriate? YES / **NO**  Did the original study claim the device was valid? **YES (PV)** / **NO (MV)**  Does this device validly measure what was measured? YES / **NO** |
| Lorenzetti, Lamparter [10] | Myotest | Vicon 3D Motion Capture | F/W Back Squat, F/W Ballistic Squat | F/W Back Squat:  70% 1RM, F/W Ballistic Squat: 25 kg | Mean Velocity, Maximum Velocity,  Time to Maximum Velocity | 9 | F/W Ballistic Squat  Mean Velocity RMSE: 0.233 m/s;  MD: 0.149 m/s  Maximum velocity  RMSE: 0.418 m/s; MD: 0.278 m/s  Time to maximum velocity RMSE: 0.054 s;  MD: -0.034 s | Was a gold standard criterion used? **YES** / NO  Where the statistics/combination of used to validate the device appropriate? YES / **NO**  Did the original study claim the device was valid? **YES (Ballistic Squat)** /  **NO Back Squat**  Does this device validly measure what was measured? YES / **NO** |
| Mateo [96] | RehaGait sensor (Bar Attachment)   RehaGait sensor (Lower Back Attachment) | MyLift App via  iPhone 6 with  an iOS 9.3.2. version. | F/W Back Squat | <1 kg | Mean Velocity | 6 | RehaGait sensor (Barbell Attachment)  Mean Concentric Velocity t = 6.518,  p = 0.001,  mean difference (±SD) = 0.364 (0.069)  RehaGait sensor (Lower Back Attachment)  Mean Concentric Velocity t = 3.373,  p = 0.020,  mean difference (±SD) = 0.318 (0.08) | Was a gold standard criterion used? YES / **NO**  Where the statistics/combination of used to validate the device appropriate? YES / **NO**  Did the original study claim the device was valid? YES / **NO**  Does this device validly measure what was measured? YES / **NO** |
| McGrath, Flanagan [61] | Push Band | Eagle 3D Motion Capture Camera | F/W Bench Press | 40%, 80% 1RM | Mean Velocity | 10 | 40% 1RM Mean Velocity Mean ± SD: 0.746 ± 0.124 m/s;  CV: 16.62%  80% 1RM Mean Velocity Mean ± SD: 0.322 ± 0.124 m/s;  CV: 38.50%   Combined Load  Mean Velocity Mean ± SD: 0.510 ± 0.244 m/s;  CV: 47.83%;  ICC (95% CI): 0.923 (0.889-0.946) | Was a gold standard criterion used? **YES** / NO  Where the statistics/combination of used to validate the device appropriate? YES / **NO**  Did the original study claim the device was valid? YES / **NO**  Does this device validly measure what was measured? YES / **NO** |
| Menrad and Edelmann-Nusser [53] | VmaxPro | Vicon (MX T20) 3D Motion Capture 12 Camera Setup | F/W Back Squats, F/W Standing Bent Over Rows, F/W Deadlift | 40%,60%, 80% 1RM | Mean Velocity | 12 | F/W Back Squat  Mean Velocity R2: 0.9848 LoA (m/s): 0.084  F/W Barbell Row Mean Velocity R2: 0.9759 LoA (m/s): 0.093  F/W Deadlift  Mean Velocity R2: 0.9854 LoA (m/s): 0.076  All Exercises: Mean Velocity R2: 0.9835 LoA (m/s): 0.085 | Was a gold standard criterion used? **YES** / NO  Where the statistics/combination of used to validate the device appropriate? YES / **NO**  Did the original study claim the device was valid? **YES** / NO  Does this device validly measure what was measured? YES / **NO** |
|  | Push Band 2.0 | Vicon (MX T20) 3D Motion Capture 12 Camera Setup | F/W Back Squats, F/W Standing Bent over Rows, F/W Deadlift | 40%,60%, 80% 1RM | Mean Velocity | 12 | F/W Back Squat  Mean Velocity R2: 0.9583 LoA (m/s): 0.143  F/W Barbell Row Mean Velocity R2: 0.8857 LoA (m/s): 0.284  F/W Deadlift  Mean Velocity R2: 0.831 LoA (m/s): 0.335  All Exercises: Mean Velocity R2: 0.8758 LoA (m/s): 0.272 | Was a gold standard criterion used? **YES** / NO  Where the statistics/combination of used to validate the device appropriate? YES / **NO**  Did the original study claim the device was valid? **YES** / NO  Does this device validly measure what was measured? YES / **NO** |
| Merrigan and Martin [98] | Output  Sports Unit | Four PCTs (PT5A-150 Celesco; Measurement Specialties, Chats-worth, CA | F/W Back Squat, F/W Bench Press | 35%, 45%, 55%, 65%, 75%, 85% 1RM | Mean Velocity, Peak Velocity | 11 | Bench Press  All Loads MV Systematic Bias (95% CI): -0.032†  (-0.060 to -0.005 PV Systematic Bias (95% CI): -0.138†  (-0.188 to -0.089)  35% MV Systematic Bias (95% CI): -0.141†  (-0.209 to -0.073)  PV: Systematic Bias (95% CI): -0.026 (-0.183 to 0.131)  45% MV Systematic Bias (95% CI): -0.023 (-0.047 to 0.001) PV: Systematic Bias (95% CI): 0.013 (-0.066 to 0.092)  55% MV Systematic Bias (95% CI): -0.022 (-0.129 to 0.084) PV: Systematic Bias (95% CI): -0.295†  (-0.399 to -0.192)  65% MV Systematic Bias (95% CI): -0.074†  (-0.118 to -0.030) PV: Systematic Bias (95% CI): -0.237†  (-0.370 to -0.105)  75% MV Systematic Bias (95% CI): .008 (20.048 to 0.065) PV: Systematic Bias (95% CI): -0.140 (-0.289 to 0.008)  85% MV Systematic Bias (95% CI): 0.015 (-0.082 to 0.111) PV: Systematic Bias (95% CI): -0.211†  (-0.359 to -0.063)  Back Squat  All Loads MV Systematic Bias (95% CI): 045†  (0.017 to 0.073) PV: Systematic Bias (95% CI): 0.306†  (0.261 to 0.352)  35% MV Systematic Bias (95% CI): 0.232†  (0.145 to 0.319) PV: Systematic Bias (95% CI): -0.034 (-0.160 to 0.092)  45% MV Systematic Bias (95% CI): 0.164†  (0.049 to 0.280) PV: Systematic Bias (95% CI): -0.015 (-0.191 to 0.160)  55% MV Systematic Bias (95% CI): 0.185†  (0.072 to 0.297) PV: Systematic Bias (95% CI): -0.129 (-0.348 to 0.089)  65% MV Systematic Bias (95% CI): 0.238†  (0.169 to 0.307) PV: Systematic Bias (95% CI): 0.226†  (0.070 to 0.382)  75% MV Systematic Bias (95% CI): 0.127†  (0.037 to 0.218) PV: Systematic Bias (95% CI): 0.251†  (0.125 to 0.376)  85% MV Systematic Bias (95% CI): 0.089†  (0.012 to 0.165) PV: Systematic Bias (95% CI): 0.264†  (0.139 to 0.388)  † = Systematic bias (intercept CI95% does not cross 0) and proportional bias (slope CI95% does not cross 1) | Was a gold standard criterion used? YES / **NO**  Where the statistics/combination of used to validate the device appropriate? YES / **NO**  Did the original study claim the device was valid? YES / **NO**  Does this device validly measure what was measured? YES / **NO** |
| Mitter, Hölbling [54] | Push Band | Vicon 3D Motion Capture | F/W Back Squat, F/W Bench Press, F/W Deadlift | 30-100% 1RM | Peak Velocity, Mean Velocity | 24 | F/W Back Squat  30-100% 1RM  Peak Velocity  SEE: 0.137 m/s  RMSE: 0.229 m/s Mean Velocity  SEE: 0.078 m/s RMSE: 0.147 m/s  F/W Bench Press  30-100% 1RM  Peak Velocity  SEE: 0.113 m/s  RMSE: 0.121 m/s Mean Velocity  SEE: 0.065 m/s  RMSE: 0.101 m/s  F/W Deadlift  30-100% 1RM  Peak Velocity  SEE: 0.183 m/s RMSE 0.235 m/s Mean Velocity  SEE: 0.105 m/s  RMSE: 0.136 m/s | Was a gold standard criterion used? **YES** / NO  Where the statistics/combination of used to validate the device appropriate? YES / **NO**  Did the original study claim the device was valid? **YES** / NO  Does this device validly measure what was measured? YES / **NO** |
|  | Beast Sensor | Vicon 3D Motion Capture | F/W Back Squat F/W Bench Press F/W Deadlift | 30-100% 1RM | Peak Velocity Mean Velocity | 24 | F/W Back Squat  30-100% 1RM  Peak Velocity  SEE: 0.176 m/s  RMSE: 0.320 m/s Mean Velocity SEE: 0.116 m/s RMSE: 0.177 m/s   F/W Bench Press  30-100% 1RM  Peak Velocity  SEE: 0.113 m/s  RMSE: 0.134 m/s Mean Velocity  SEE: 0.084 m/s  RMSE: 0.098 m/s  F/W Deadlift  30-100% 1RM  Peak Velocity  SEE: 0.147 m/s;  RMSE: 0.361 m/s Mean Velocity  SEE: 0.105 m/s RMSE: 0.200 m/s | Was a gold standard criterion used? **YES** / NO  Where the statistics/combination of used to validate the device appropriate? YES / **NO**  Did the original study claim the device was valid? **YES** / NO  Does this device validly measure what was measured? YES / **NO** |
| Muyor, Granero-Gil [15] | WIMU System | T-Force | S/M Back Squat | 40%, 80% 1RM | Mean Velocity, Eccentric Mean Velocity | 23 | 40% 1RM  Mean Velocity (con)  ES: 0.34d SEM: 0.003 m/s ICC: 0.970 Mean Velocity (ecc)  ES: 0.21d SEM: 0.007 m/s ICC: 0.971  80% 1RM  Mean Velocity (con)  ES: 0.25d SEM: 0.003 m/s ICC: 0.976 Mean Velocity (ecc)  ES: 0.44d  SEM: 0.005 m/s  ICC: 0.953 | Was a gold standard criterion used? YES / **NO**  Where the statistics/combination of used to validate the device appropriate? YES / **NO**  Did the original study claim the device was valid? **YES** / NO  Does this device validly measure what was measured? YES / **NO** |
| Oberhofer et al. (81) | Apple Watch Sport (1st generation) via Phone 6s with iOS 11.4.1 installed.  + StrengthControl App | LPT | F/W Bench Press, F/W Back Squat, F/W Deadlift | 60-80% 1RM | Exercise Recognition, Repetition Count, 1RM prediction | 30 | Exercise Recognition  BB bench press Number of Sets:119 True: 115 False: 3 NILL: 1 % True: 96.5%  BB back squat Number of Sets: 121 True: 98 False: 10 NILL: 13 % True: 76.5%  BB deadlift Number of Sets: 124 True: 115 False: 4 NILL: 5 % True: 92.2%  Total Number of Sets: 363 True: 327 False: 17 NILL: 19 % True: 88.4%  Repetition Count  BB bench press Number of Sets: 115 TRmean: 8.84 ± 2.01  RRmean: 9.44 ± 3.15 RMSE: 1.36 ± 2.16 p-Value: 0.01 Pearson: 0.61  BB back squat Number of Sets: 98 TRmean: 8.84 ± 2.01 RRmean: 9.44 ± 3.15 RMSE: 9.44 ± 3.15 p-Value: 0.68 Pearson: 0.61  BB deadlift Number of Sets: 115 TRmean: 9.41 ± 1.54 RRmean: 9.97 ± 3.80 RMSE: 2.57 ± 2.47 p-Value: 0.09 Pearson: 0.37  Total Number of Sets: 327 TRmean: 9.23 ± 1.59 RRmean: 9.58 ± 3.67 RMSE: 2.14 ± 2.56 p-Value: 0.06 Pearson: 0.40  1RM Predictions  BB bench press Number of subjects: 30 Attempts: 30 Predicted: 2 % Success: 6.7%  BB back squat Number of subjects: 30 Attempts: 30 Predicted: 1 % Success: 3.3%  BB deadlift Number of subjects: 30 Attempts:30 Predicted: 3 % Success: 10%  Total Number of subjects: 30 Attempts: 90 Predicted: 6 % Success: 8.9% | Was a gold standard criterion used? YES / **NO**  Where the statistics/combination of used to validate the device appropriate? YES / **NO**  Did the original study claim the device was valid? YES / **NO**  Does this device validly measure what was measured? YES / **NO** |
| Olaya-Cuartero, Villalón-Gasch [48] | VmaxPro | Optitrack 3D Motion Capture | F/W Back Squat | 75%, 85%, 90%, 95% 1RM | Mean Velocity, Displacement | 20 | Mean Velocity (m/s) Mean Difference: -0.021 (-0.025 to -0.016)  Systematic Error: -0.021 Random Error: 0.053 r (95% CI): 0.992 (0.990 to 0.994)  Displacement (cm) Mean Difference: 1.935 (1.796 to 2.090) Systematic Error: 1.935 Random Error: 1.901 r (95% CI): 0.957 (0.938 to 0.971) | Was a gold standard criterion used? **YES** / NO  Where the statistics/combination of used to validate the device appropriate? **YES** / NO  Did the original study claim the device was valid? **YES** / NO  Does this device validly measure what was measured? **YES /** NO |
| Orange, Metcalfe [11] | Push Band | GymAware | F/W Back Squat  F/W Bench Press | 20%, 40%, 60%, 80%, 90% 1RM | Mean Velocity, Peak Velocity | 29 | F/W Back Squat   20% 1RM  Mean Velocity Standardized mean bias: 0.61 m/s r:0.80 Peak Velocity Standardized mean bias: 0.53 m/s  r:0.80  40% 1RM  Mean Velocity Standardized mean bias: 1.17 m/s  r: 0.72 Peak Velocity Standardized mean bias: 1.20 m/s  r:0.70  60% 1RM  Mean Velocity Standardized mean bias: 1.41 m/s  r: 0.78 Peak Velocity Standardized mean bias: 1.58 m/s r:0.68  80% 1RM  Mean Velocity Standardized mean bias: 2.23 m/s  r: 0.79 Peak Velocity Standardized mean bias: 2.23 m/s  r:0.84  90% 1RM  Mean Velocity Standardized mean bias: 2.61 m/s r:0.66 Peak Velocity Standardized mean bias: 2.74 m/s r:0.41   F/W Bench Press   20% 1RM  Mean Velocity  Standardized mean bias: 1.06 m/s  r: 0.30 Peak Velocity  Standardized mean bias: 0.55 m/s r:0.44  40% 1RM  Mean Velocity  Standardized mean bias: 0.68 m/s r: 0.85 Peak Velocity  Standardized mean bias: 0.37 m/s r:0.73  60% 1RM Mean Velocity  Standardized mean bias: 0.55 m/s r: 0.59 Peak Velocity  Standardized mean bias: 0.18 m/s r:0.52  80% 1RM  Mean Velocity  Standardized mean bias: 1.03 m/s; r: 0.73 Peak Velocity  Standardized mean bias: 0.00 m/s  r: 0.50  90% 1RM  Mean Velocity  Standardized mean bias: 1.12 m/s r: 0.44 Peak Velocity  Standardized mean bias: 0.10 m/s r: 0.45 | Was a gold standard criterion used? YES / **NO**  Where the statistics/combination of used to validate the device appropriate? YES / **NO**  Did the original study claim the device was valid? YES / **NO**  Does this device validly measure what was measured? YES / **NO** |
| Orser, Agar-Newman [92] | Push Band 2.0 | Dual Force Plates  (AMTI OR6-7, Massachusetts, USA)  recording at 1000 Hz | Squat Jump | B/W, 15 kg, 30 kg, 45 kg, 60 kg | Mean Velocity, Peak Velocity | 15 | Mean Velocity R2: 0.876 Bias: 0.078 m/s or 7.40% LOA's: Range = 0.59% to 12.8%  Peak Velocity R2: 0.852 Bias: 0.293 m/s or 13.9% | Was a gold standard criterion used? YES / **NO**  Where the statistics/combination of used to validate the device appropriate? **YES** / NO  Did the original study claim the device was valid? YES / **NO**  Does this device validly measure what was measured? YES / **NO** |
| Pérez-Castilla, Piepoli [66] | Push Band | Optitrack 3D motion Capture | S/M Bench Press | 45-85% 1RM | Mean Velocity | 14 | Bias: 0.10 ± 0.06 m/s r = 0.947 | Was a gold standard criterion used? **YES** / NO  Where the statistics/combination of used to validate the device appropriate? YES / **NO**  Did the original study claim the device was valid? **YES** / NO  Does this device validly measure what was measured? YES / **NO** |
|  | Beast Sensor | Optitrack 3D motion Capture | S/M Bench Press | 45-85% 1RM | Mean Velocity | 14 | Bias: 0.05 ± 0.21 m/s r=0.765 | Was a gold standard criterion used? **YES** / NO  Where the statistics/combination of used to validate the device appropriate? YES / **NO**  Did the original study claim the device was valid? **YES** / NO  Does this device validly measure what was measured? YES / **NO** |
| Pino-Ortega, Bastida-Castillo [94] | WIMU System | GymAware | Leg Extension | 30-90 kg | Mean Velocity | 10 | R2: 0.9995  Bias: 0.011 ± 0.006 m/s;  LoA: - 0.024 to 0.01 m/s | Was a gold standard criterion used? YES / **NO**  Where the statistics/combination of used to validate the device appropriate? YES / **NO**  Did the original study claim the device was valid? **YES** / NO  Does this device validly measure what was measured? YES / **NO** |
| Sato, K. Beckham [88] | Push Band | Vicon (Nexus 1.8.5) 3D Motion Capture | DB Biceps Curl, DB Shoulder Press | 4.54 to 6.82 kg | Mean Velocity, Peak Velocity | 5 | DB Biceps Curl  Mean Velocity TE: 0.060 m/s;  RTE: 7.2%; r: 0.883  Peak Velocity TE: 0.105 m/s;  RTE: 6.5%; r: 0.923  DB Shoulder Press  Mean Velocity TE: 0.090 m/s;  RTE: 12.6%; r: 0.864  Peak Velocity TE: 0.163 m/s;  RTE: 14.0%; r: 0.801 | Was a gold standard criterion used? **YES** / NO  Where the statistics/combination of used to validate the device appropriate? YES / **NO**  Did the original study claim the device was valid? **YES** / NO  Does this device validly measure what was measured? **YES** / NO |
| Suchomel, Techmanski [59] | Push Band 2.0 | GymAware | Barbell Jump Shrug, Barbell Hang High Pull | 20%, 40%, 60%, 80%, 100% 1RM | Mean Velocity, Peak Velocity | 15 | JUMP SHRUG 20% Load R²: MV = 0.84, PV = 0.78 Slope: MV = 1.009 (0.837–1.181), PV = 1.117 (0.693–1.541) Intercept: MV = -0.042 (−0.309–0.226), PV = -0.213 (−1.334–0.908)  40% Load R²: MV = 0.64, PV = 0.66 Slope: MV = 1.006 (0.610–1.402), PV = 0.994 (0.691–1.297) Intercept: MV = -0.005 (−0.575–0.565), PV = 0.051 (−0.690–0.791)  60% Load R²: MV = 0.75, PV = 0.89 Slope: MV = 0.949 (0.697–1.202), PV = 1.042 (0.845–1.238) Intercept: MV = 0.100 (−0.194–0.394), PV = -0.018 (−0.432–0.395)  80% Load R²: MV = 0.86, PV = 0.89 Slope: MV = 1.064 (0.901–1.227), PV = 1.054 (0.900–1.207) Intercept: MV = -0.016 (−0.184–0.152), PV = 0.008 (−0.283–0.300)  100% Load R²: MV = 0.47, PV = 0.58 Slope: MV = 1.152 (0.926–1.378), PV = 1.248 (0.992–1.504) Intercept: MV = -0.114 (−0.378–0.150), PV = -0.340 (−0.790–0.109)  HANG HIGH PULL 20% Load R²: MV = 0.77, PV = 0.65 Slope: MV = 0.681 (0.442–0.921), PV = 1.059 (0.617–1.501) Intercept: MV = 0.592 (0.149–1.034) ‡, PV = -0.008 (−1.321–1.305)  40% Load R²: MV = 0.89, PV = 0.65 Slope: MV = 0.779 (0.604–0.954), PV = 0.948 (0.661–1.235) Intercept: MV = 0.446 (0.185–0.708) ‡, PV = 0.226 (−0.501–0.953)  60% Load R²: MV = 0.96, PV = 0.62 Slope: MV = 0.905 (0.825–0.985), PV = 1.009 (0.689–1.328) Intercept: MV = 0.247 (0.141–0.353) ‡, PV = 0.121 (−0.534–0.777)  80% Load R²: MV = 0.91, PV = 0.87 Slope: MV = 0.953 (0.752–1.155), PV = 0.971 (0.838–1.104) Intercept: MV = 0.155 (−0.062–0.372), PV = 0.223 (−0.011–0.458)  100% Load R²: MV = 0.91, PV = 0.92 Slope: MV = 1.010 (0.798–1.222), PV = 0.966 (0.832–1.100) Intercept: MV = 0.101 (−0.111–0.312), PV = 0.230 (0.012–0.448) | Was a gold standard criterion used? YES / **NO**  Where the statistics/combination of used to validate the device appropriate? YES / **NO**  Did the original study claim the device was valid? YES / **NO**  Does this device validly measure what was measured? YES / **NO** |
| Thompson, Rogerson [55] | Push Band (body) | 12 Camera Raptor  3D Motion Capture | F/W Back Squat, Power Clean | 40%, 50%, 60%, 70%, 80%, 90%, 100% 1RM | Mean Velocity, Peak Velocity | 11 | F/W BACK SQUAT  40% 1RM  Mean Velocity  R2: 0.92; LoA: 0.00 ± 0.06 m/s Peak Velocity  R2: 0.94; LoA: 0.09 ± 0.10 m/s  50% 1RM  Mean Velocity  R2: 0.96; LoA: 0.00 ± 0.04 m/s Peak Velocity  R2: 0.76; LoA: 0.09 ± 0.22 m/s  60% 1RM  Mean Velocity  R2: 0.95; LoA: 0.01 ± 0.05 m/s Peak Velocity  R2: 0.45; LoA: 0.12 ± 0.33 m/s  70% 1RM  Mean Velocity R2: 0.88; LoA: 0.02 ± 0.07 m/s Peak Velocity  R2: 0.60; LoA: 0.11 ± 0.29 m/s  80% 1RM  Mean Velocity  R2: 0.92; LoA: 0.00 ± 0.08 m/s Peak Velocity  R2: 0.37; LoA: 0.13 ± 0.44 m/s  90% 1RM  Mean Velocity  R2: 0.79; LoA: 0.00 ± 0.10 m/s Peak Velocity  R2: 0.53; LoA: 0.15 ± 0.30 m/s  100% 1RM  Mean Velocity R2: 0.58; LoA: - 0.07 ± 0.10 m/s Peak Velocity  R2: 0.48; LoA: 0.12 ± 0.35 m/s  Full Mean Velocity  R2: 0.97; LoA: - 0.01 ± 0.09 m/s Peak Velocity  R2: 0.80; LoA: 0.12 ± 0.30 m/s  POWER CLEAN  40% 1RM  Mean Velocity  R2: 0.38; LoA: 0.07 ± 0.24 m/s Peak Velocity  R2: 0.27; LoA: 0.61 ± 0.40 m/s  50% 1RM  Mean Velocity  R2: 0.50; LoA: 0.07 ± 0.23 m/s Peak Velocity  R2: 0.43; LoA: 0.60 ± 0.38 m/s  60% 1RM  Mean Velocity  R2: 0.50; LoA: 0.07 ± 0.18 m/s Peak Velocity  R2: 0.24; LoA: 0.56 ± 0.42 m/s  70% 1RM  Mean Velocity  R2: 0.66; LoA: 0.07 ± 0.18 m/s Peak Velocity  R2: 0.43; LoA: 0.59 ± 0.32 m/s  80% 1RM  Mean Velocity  R2: 0.54; LoA: 0.07 ± 0.22 m/s Peak Velocity  R2: 0.27; LoA: 0.58 ± 0.36 m/s  90% 1RM  Mean Velocity  R2: 0.61; LoA: 0.10 ± 0.19 m/s Peak Velocity  R2: 0.60; LoA: 0.62 ± 0.24 m/s  100% 1RM  Mean Velocity  R2: 0.34; LoA: 0.09 ± 0.16 m/s Peak Velocity  R2: 0.66; LoA: 0.59 ± 0.17 m/s  Full Mean Velocity  R2: 0.72; LoA: 0.08 ± 0.19 m/s Peak Velocity  R2: 0.65; LoA: 0.59 ± 0.32 m/s | Was a gold standard criterion used? **YES** / NO  Where the statistics/combination of used to validate the device appropriate? YES / **NO**  Did the original study claim the device was valid? YES / **NO**  Does this device validly measure what was measured? YES / **NO** |
|  | Push Band (bar) | 12 Camera Raptor  3D Motion Capture | F/W Back Squat, Power Clean | 40%, 50%, 60%, 70%, 80%, 90%, 100% 1RM | Mean Velocity, Peak Velocity | 11 | F/W Back Squat   40% 1RM  Mean Velocity  R2: 0.69; LoA: - 0.08 ± 0.09 m/s Peak Velocity  R2: 0.91; LoA: 0.02 ± 0.14 m/s  50% 1RM  Mean Velocity  R2: 0.95; LoA: - 0.06 ± 0.05 m/s Peak Velocity  R2: 0.89; LoA: 0.07 ± 0.14 m/s  60% 1RM  Mean Velocity  R2: 0.83; LoA: - 0.07 ± 0.08 m/s Peak Velocity  R2: 0.84; LoA: 0.06 ± 0.17 m/s  70% 1RM  Mean Velocity  R2: 0.84; LoA: - 0.07 ± 0.07 m/s Peak Velocity  R2: 0.80; LoA: 0.09 ± 0.18 m/s  80% 1RM  Mean Velocity  R2: 0.87; LoA: - 0.07 ± 0.09 m/s Peak Velocity R2: 0.60; LoA: 0.08 ± 0.28 m/s  90% 1RM  Mean Velocity  R2: 0.92; LoA: - 0.07 ± 0.05 m/s Peak Velocity  R2: 0.56; LoA: 0.08 ± 0.29 m/s  100% 1RM  Mean Velocity  R2: 0.39; LoA: 0.10 ± 0.13 m/s Peak Velocity  R2: 0.41; LoA: 0.01± 0.36 m/s  Full  Mean Velocity  R2: 0.97; LoA: - 0.07 ± 0.08 m/s Peak Velocity  R2: 0.86; LoA: 0.06 ± 0.23 m/s  Power clean   40% 1RM  Mean Velocity  R2: 0.62; LoA: 0.22 ± 0.18 m/s Peak Velocity  R2: 0.59; LoA: 0.35 ± 0.43 m/s  50% 1RM  Mean Velocity  R2: 0.54; LoA: 0.22 ± 0.22 m/s Peak Velocity  R2: 0.11; LoA: 0.36 ± 0.62 m/s  60% 1RM  Mean Velocity  R2: 0.50; LoA: 0.22 ± 0.15 m/s Peak Velocity  R2: 0.68; LoA: 0.35 ± 0.26 m/s  70% 1RM  Mean Velocity  R2: 0.24; LoA: 0.16 ± 0.26 m/s Peak Velocity  R2: 0.08; LoA: 0.31 ± 0.74 m/s  80% 1RM  Mean Velocity  R2: 0.35; LoA: 0.14 ± 0.22 m/s Peak Velocity  R2: 0.06; LoA: 0.30 ± 0.64 m/s  90% 1RM  Mean Velocity  R2: 0.41; LoA: 0.19 ± 0.15 m/s Peak Velocity  R2: 0.26; LoA: 0.46 ± 0.35 m/s  100% 1RM  Mean Velocity  R2: 0.23; LoA: 0.14 ± 0.16 m/s Peak Velocity  R2: 0.60; LoA: 0.35 ± 0.23 m/s  Full  Mean Velocity  R2: 0.62; LoA: 0.18 ± 0.20 m/s Peak Velocity  R2: 0.48; LoA: 0.35 ± 0.49 m/s | Was a gold standard criterion used? **YES** / NO  Where the statistics/combination of used to validate the device appropriate? YES / **NO**  Did the original study claim the device was valid? YES / **NO**  Does this device validly measure what was measured? YES / **NO** |
|  | Bar Sensei | 12 Camera Raptor  3D Motion Capture | F/W Back Squat, Power Clean | 40%, 50%, 60%, 70%, 80%, 90%, 100% 1RM | Mean Velocity, Peak Velocity | 11 | F/W Back Squat   40% 1RM  Mean Velocity R2: 0.82; LoA: 0.07 ± 0.12 m/s Peak Velocity  R2: 0.96; LoA: 0.03 ± 0.12 m/s  50% 1RM  Mean Velocity  R2: 0.75; LoA: 0.05 ± 0.16 m/s Peak Velocity  R2: 0.93; LoA: 0.07 ± 0.16 m/s  60% 1RM  Mean Velocity  R2: 0.67; LoA: 0.04 ± 0.12 m/s Peak Velocity  R2: 0.82; LoA: 0.12 ± 0.17 m/s  70% 1RM  Mean Velocity  R2: 0.86; LoA: 0.04 ± 0.08 m/s Peak Velocity  R2: 0.66; LoA: 0.19 ± 0.25 m/s  80% 1RM  Mean Velocity  R2: 0.66; LoA: 0.05 ± 0.11 m/s Peak Velocity  R2: 0.52; LoA: 0.31 ± 0.33 m/s  90% 1RM  Mean Velocity  R2: 0.23; LoA: 0.01 ± 0.21 m/s Peak Velocity R2: 0.10; LoA: 0.31 ± 0.33 m/s  100% 1RM  Mean Velocity  R2: 0.01; LoA: - 0.05 ± 0.18 m/s Peak Velocity  R2: 0.02; LoA: 0.24 ± 0.49 m/s  Full  Mean Velocity  R2: 0.87; LoA: 0.03 ± 0.16 m/s Peak Velocity  R2: 0.80; LoA: 0.18 ± 0.37 m/s  Power Clean  40% 1RM  Mean Velocity  R2: 0.82; LoA: - 0.13 ± 0.14 m/s Peak Velocity  R2: 0.47; LoA: - 0.36 ± 0.38 m/s  50% 1RM  Mean Velocity  R2: 0.82; LoA: - 0.08 ± 0.15 m/s Peak Velocity  R2: 0.22; LoA: - 0.25 ± 0.49 m/s  60% 1RM  Mean Velocity  R2: 0.73; LoA: - 0.10 ± 0.18 m/s Peak Velocity  R2: 0.61; LoA: - 0.20 ± 0.35 m/s  70% 1RM  Mean Velocity  R2: 0.04; LoA: - 0.02 ± 0.34 m/s Peak Velocity  R2: 0.50; LoA: - 0.15 ± 0.38 m/s  80% 1RM  Mean Velocity  R2: 0.07; LoA: 0.01 ± 0.27 m/s peak velocity  R2: 0.69; LoA: - 0.12 ± 0.27 m/s  90% 1RM  Mean Velocity  R2: 0.18; LoA: 0.02 ± 0.22 m/s Peak Velocity  R2: 0.84; LoA: - 0.04 ± 0.18 m/s  100% 1RM  Mean Velocity  R2: 0.02; LoA: 0.01 ± 0.27 m/s Peak Velocity  R2: 0.57; LoA: - 0.05 ± 0.27 m/s  Full  Mean Velocity  R2: 0.73; LoA: - 0.04 ± 0.25 m/s Peak Velocity  R2: 0.74; LoA: - 0.17 ± 0.39 m/s | Was a gold standard criterion used? **YES** / NO  Where the statistics/combination of used to validate the device appropriate? YES / **NO**  Did the original study claim the device was valid? YES / **NO**  Does this device validly measure what was measured? YES / **NO** |
|  | Beast Sensor | 12 Camera Raptor  3D Motion Capture | F/W Back Squat, Power Clean | 40%, 50%, 60%, 70%, 80%, 90%, 100% 1RM | Mean Velocity, Peak Velocity | 11 | F/W Back Squat  40% 1RM  Mean Velocity  R2: 0.64; LoA: - 0.01 ± 0.16 m/s Peak Velocity  R2: 0.10; LoA: - 0.05 ± 0.50 m/s  50% 1RM  Mean Velocity  R2: 0.71; LoA: 0.04 ± 0.13 m/s Peak Velocity  R2: 0.12; LoA: - 0.04 ± 0.43 m/s  60% 1RM  Mean Velocity  R2: 0.49; LoA: 0.08 ± 0.21 m/s Peak Velocity  R2: 0.00; LoA: 0.02 ± 0.53 m/s  70% 1RM  Mean Velocity  R2: 0.46; LoA: 0.11 ± 0.18 m/s Peak Velocity  R2: 0.00; LoA: 0.01 ± 0.56 m/s  80% 1RM  Mean Velocity  R2: 0.58; LoA: 0.16 ± 0.18 m/s Peak Velocity  R2: 0.02; LoA: 0.04 ± 0.44 m/s  90% 1RM  Mean Velocity  R2: 0.12; LoA: 0.10 ± 0.22 m/s Peak Velocity  R2: 0.58; LoA: 0.22 ± 0.28 m/s  100% 1RM  Mean Velocity  R2: 0.20; LoA: - 0.09 ± 0.26 m/s Peak Velocity  R2: 0.15; LoA: 0.25 ± 0.52 m/s  Full  Mean Velocity  R2: 0.80; LoA: 0.06 ± 0.24 m/s Peak Velocity  R2: 0.57; LoA: 0.06 ± 0.51 m/s | Was a gold standard criterion used? **YES** / NO  Where the statistics/combination of used to validate the device appropriate? YES / **NO**  Did the original study claim the device was valid? YES / **NO**  Does this device validly measure what was measured? YES / **NO** |
| Van Den Tillaar and Ball [82] | Push Band | ET-Enc-02 Ergotest Linear Encoder | F/W Bench Press, Push-Up | F/W Bench Press: 50% 1RM, + 10 to 30 kg, Push Up:  Body Weight, 10-30 kg Weight vests | Mean Velocity, Mean peak velocity | 20 | F/W Bench Press   50% 1RM, + 10 to 30 kg  Mean Velocity  Bias: 0.11 m/s;  SEE: 0.17 m/s r:0.62 Mean Peak Velocity  Bias: 0.22 m/s;  SEE: 0.33 m/s r:0.49  Push-Up   Body Weight, 10-30 kg Weight vests Mean Velocity  Bias: 0.12 m/s;  SEE: 0.16 m/s r: 0.70 Mean Peak Velocity  Bias: 0.15 m/s;  SEE: 0.34 m/s r: 0.46 | Was a gold standard criterion used? YES / **NO**  Where the statistics/combination of used to validate the device appropriate? YES / **NO**  Did the original study claim the device was valid? YES / **NO**  Does this device validly measure what was measured? YES / **NO** |

*Abbreviations: 1RM: One Repetition Maximum, Bias: Difference between the mean outcome of the device and the criterion measure, CC: Concordance Correlation, CV%: Coefficient of Variation Percent, ES: Effect Size, F/W: Free Weight, ICC: Intraclass Correlation Coefficient, kg: Kilograms, LoA: Limits of Agreement, MARD: Mean Absolute Relative Difference, MD: Mean Difference, m/s: Meters per Second, R2: Coefficient of Determination, RMSE: Root Mean Square Error, r: Pearson correlation coefficient, RTE: Relative Typical Error, SEE: Standard Error of Estimate, SEM: Standard Error of Measurement, S/M: Smith Machine, TE: Typical Error.*

S9 Table: Studies investigating the validity of a 2D Motion Analysis device.

| **Study** | **Device/s** | **Criterion** | **Exercise/s** | **Intensity/Load** | **Variable/s Measured** | **Sample Size** | **Reported Statistics** | **Validity Criteria** |
| --- | --- | --- | --- | --- | --- | --- | --- | --- |
| Balsalobre-Fernández, Marchante [78] | PowerLift  (v4.0 iOS) | SmartCoach  Power Encoder | F/W Back Squat, F/W Bench Press, F/W Hip Thrust | 50-95% 1RM | Mean Velocity | 10 | F/W Back Squat  50-95% 1RM  Mean Velocity  Bias: - 0.005 ± 0.04 m/s SEE: 0.04 m/s  F/W Bench Press  50-95% 1RM  Mean Velocity  Bias: - 0.01 ± 0.05 m/s SEE: 0.05 m/s  F/W Hip-Thrust  50-95% 1RM Mean Velocity  Bias: 0.02 ± 0.04 m/s SEE: 0.03 m/s | Was a gold standard criterion used? YES / **NO**  Where the statistics/combination of used to validate the device appropriate? YES / **NO**  Did the original study claim the device was valid? **YES** / NO  Does this device validly measure what was measured? YES / **NO** |
| Balsalobre-Fernández, Marchante [101] | PowerLift  (v2.8iOS) | SmartCoach  Power Encoder | F/W Bench Press | 75-100% 1RM | Mean Velocity | 10 | r: 0.94; SEE: 0.028 m/s; ICC: 0.965  MD: 0.008±0.03 m/s | Was a gold standard criterion used? YES / **NO**  Where the statistics/combination of used to validate the device appropriate? **YES** / NO  Did the original study claim the device was valid? **YES** / NO  Does this device validly measure what was measured? YES / **NO** |
| Balsalobre-Fernández, Xu [103] | My Jump Lab v 3.0  with iPhone 12 Pro running iOS 15.5 (previously My Lift) | GymAware | F/W Bench Press | 50%, 75% 1RM | Mean Velocity | 27 | 50% 1RM ES: Trivial  r: 0.9 (0.82-0.97) Mean Difference: -0.010 m/s (95% CI: -0.024 to 0.003 m/s)  75% 1RM ES: Trivial  r: 0.92 (0.86-0.98) Mean Difference: -0.026 m/s (95% CI: -0.038 to -0.014 m/s) | Was a gold standard criterion used? YES / **NO**  Where the statistics/combination of used to validate the device appropriate? **YES** / NO  Did the original study claim the device was valid? **YES** / NO  Does this device validly measure what was measured? YES / **NO** |
| Cetin and Isik [102] | My Lift app (v.9.1.3) iPad Pro 11. (Apple, USA) | Traditional  1RM Assessment | F/W Back Squat, F/W Deadlift | 75%, 85% 1RM | Mean Velocity | 10 | Deadlift:  Estimated 1RM ± S.D. = 129.10 ± 35.70 kg;  Actual 1RM ±S.D.= 128.48 ± 35.28 kg; p=0.248 R2= 0.99  Squat: Estimated 1RM ± S.D. = 137.26 ± 42.80 kg;  Actual 1RM ± S.D.= 137.90 ± 41.94 kg; p=0.201 R2= 0.99 | Was a gold standard criterion used? YES / **NO**  Where the statistics/combination of used to validate the device appropriate? YES / **NO**  Did the original study claim the device was valid? **YES** / NO  Does this device validly measure what was measured? YES / **NO** |
| Courel-Ibáñez, Martínez-Cava [68] | PowerLift (v4.0iOS) | T-Force | S/M Bench Press, S/M Back Squat | 20 kg, 30 kg. 40 kg, 50 kg, 60 kg, 70 kg, 80 kg | Mean Velocity | 17 | S/M Bench Press Mean Velocity  SEM:0.09 m/s; CV:11.7%; ICC: 0.966  S/M Back Squat Mean Velocity  SEM:0.06 m/s; CV:7.6%; ICC: 0.955 | Was a gold standard criterion used? YES / **NO**  Where the statistics/combination of used to validate the device appropriate? **YES** / NO  Did the original study claim the device was valid? YES / **NO**  Does this device validly measure what was measured? YES / **NO** |
| De Sá, Medeiros [104] | iLoad v1.0 | Chronojump | S/M Half ROM Back Squat | 10RM | Mean Velocity, Total Work | 16 | 10RM  Mean Velocity  Bias ± random error: - 0.022 ± 0.034 m/s ES: - 0.21 (- 0.39, - 0.04) r: 0.948  10RM  Total work  Bias ± random error: 0.706 ± 3.391 kJ ES: 0.04 (- 0.07, 0.16) r: 0.977 | Was a gold standard criterion used? YES / **NO**  Where the statistics/combination of used to validate the device appropriate? YES / **NO**  Did the original study claim the device was valid? **YES** / NO  Does this device validly measure what was measured? YES / **NO** |
| Jiménez-Olmedo, Penichet-Tomás [109] | Kinovea (v.0.9.1) via  Smartphone (Redmi Note 8, Xiaomi, Beijing, China) | Chronojump | S/M Half ROM Back Squat | <40% 1RM, 40 to 70% 1RM, >70% 1RM, 20 kg and 50 kg | Mean Velocity, Maximum Velocity | 15 | Mean Velocity  Bias ± random error: 0.03 ± 0.33 m/s  R2= 0.990  Maximum Velocity Bias ± random error: 0.55 ± 0.42 m/s  R2= 0.996 | Was a gold standard criterion used? YES / **NO**  Where the statistics/combination of used to validate the device appropriate? YES / **NO**  Did the original study claim the device was valid? **YES** / NO  Does this device validly measure what was measured? YES / **NO** |
| Kasovic, Martin [107] | Iron Path (version 1.9) App Via Iphone 8 | Open Barbell System | F/W Back Squat, F/W Front Squat, F/W Conventional Deadlift, F/W Sumo Deadlift | 30-100% 1RM | Mean Velocity, Range of Motion (Linear Displacement) | 27 | Front Squat  MV (m/s): OBS = 0.44 ± 0.18, IP = 0.41 ± 0.14, p = 0.041 ROM (m): OBS = 0.515 ± 0.067, IP = 0.507 ± 0.064, p = 0.216 ICC (ACV): 0.789 (95% CI (0.648 to 0.876)), SEM = 0.07 ICC (ROM): 0.231 (95% CI (-0.053 to 0.480)), SEM = 0.057  Back Squat  MV: OBS = 0.44 ± 0.15 m/s, IP = 0.43 ± 0.23 m/s, p < 0.001 ROM: OBS = 0.526 ± 0.073 m, IP = 0.502 ± 0.098 m, p = 0.004 ICC (ACV): 0.825 (95% CI (0.698 to 0.897)), SEM = 0.06 m/s  ICC (ROM): 0.384 (95% CI (0.156 to 0.574)), SEM = 0.061 m  Conventional Deadlift  MV: OBS = 0.44 ± 0.18 m/s, IP = 0.42 ± 0.15 m/s, p = 0.153 ROM: OBS = 0.548 ± 0.048 m, IP = 0.552 ± 0.071 m, p = 0.184 ICC (ACV): 0.897 (95% CI (0.849 to 0.930)), SEM = 0.05 m/s ICC (ROM): 0.502 (95% CI (0.337 to 0.636)), SEM = 0.042 m  Sumo Deadlift  MV: OBS = 0.39 ± 0.18 m/s, IP = 0.39 ± 0.17 m/s, p = 0.654 ROM: OBS = 0.489 ± 0.073 m, IP = 0.498 ± 0.079 m, p = 0.037 ICC (ACV): 0.866 (95% CI (0.805 to 0.909)), SEM = 0.02 m/s ICC (ROM): 0.643 (95% CI (0.545 to 0.770)), SEM = 0.027 m  **OBS= Open Barbell System / IP = Iron Path App | Was a gold standard criterion used? YES / **NO**  Where the statistics/combination of used to validate the device appropriate? YES / **NO**  Did the original study claim the device was valid? YES / **NO**  Does this device validly measure what was measured? YES / **NO** |
| Martínez-Cava, Hernández-Belmonte [70] | PowerLift (My Lift (v8.1 iOS) | T-Force | S/M Back Squat, S/M Bench Press | 25-95 kg | Peak Velocity | 15 | S/M Back Squat 25-95 kg  Peak Velocity ES: 0.04 (- 0.07, 0.16);  r: 0.977 SEM: 0.12 m/s;  CV: 7.59%;  ICC: 0.937  S/M Bench Press 25-95 kg  Peak Velocity SEM: 0.10 m/s;  CV: 7.04%;  ICC: 0.99 | Was a gold standard criterion used? YES / **NO**  Where the statistics/combination of used to validate the device appropriate? **YES** / NO  Did the original study claim the device was valid? YES / **NO**  Does this device validly measure what was measured? YES / **NO** |
| Martinopoulou, Tsoukos [65] | Tracker 5.0.6 software via  Casio Exilim Pro EX-F1) with sampling frequency 300 fps | Tendo | Bilateral and Unilateral  Ballistic Leg Extensions  on a Leg Press Machine | 35-106 kg  (0.34-1.53m/s) | Mean Velocity, Peak Velocity | 10 | Mean Velocity LoA: (-0.14 to 0.03 m/s) Systematic Bias:  (-0.06 ± 0.04 m/s,  CI: - 0.06 to -0.05 m/s, p<0.05) R2: 0.0015  Peak Velocity LoA: (-0.07 to 0.04 m/s) Systematic Bias:  (-0.01 ± 0.03 m/s,  CI: -0.02 to -0.01 m/s,  p < 0.05)  R2: 0.0702 | Was a gold standard criterion used? YES / **NO**  Where the statistics/combination of used to validate the device appropriate? **YES** / NO  Did the original study claim the device was valid? **YES** / NO  Does this device validly measure what was measured? YES / **NO** |
| Pérez-Castilla, Piepoli [66] | PowerLift  (v.6.0.1 iOS) | OptiTrack 3D Motion Capture | S/M Bench Press | 45-85% 1RM | Mean Velocity | 14 | Bias: - 0.01 ± 0.05 m/s r = 0.970 | Was a gold standard criterion used? **YES** / NO  Where the statistics/combination of used to validate the device appropriate? YES / **NO**  Did the original study claim the device was valid? **YES** / NO  Does this device validly measure what was measured? YES / **NO** |
| Pérez-Castilla, Boullosa [105] | iLOAD App via  two iPhone 8 Plus running iOS 12.4.5 (iPhone; Apple, Inc., Cupertino, CA). | T-Force | S/M Back Squat, S/M Bench Press | 25%, 40%, 55%, 70% 1RM | Mean Velocity | 20 | S/M Back Squat  25% 1RM Reps 1-5 p: 0.003 ES (95%CI): 0.96 (0.30 to 1.61)  r (95%CI): 0.83 (0.62-0.93)  SEE (95% CI): 0.05 m/s (0.04-0.08 m/s)  CV (95%CI): 5.57% (4.18-8.35%)  Reps 1-10 p: 0.442  ES (95%CI): 0.26 (-0.36 to 0.88 r (95%CI): 0.83 (0.62-0.93) SEE (95% CI): 0.05 m/s (0.04-0.08 m/s)  CV (95%CI): 5.62% (4.22-8.42%)  40% 1RM Reps 1-5 p: 0.16  ES (95%CI): 0.80 (0.16 to 1.45)  r (95%CI): 0.72 (0.41-0.88)  SEE (95% CI): 0.07 m/s (0.06-0.11 m/s)  CV (95%CI): 8.43% (6.31-12.7%)  Reps 1-10 p: 0.184  ES (95%CI): 0.43 (20.19 to 1.06)  r (95%CI): 0.81 (0.57-0.92)  SEE (95% CI): 0.05 m/s (0.04-0.08)  CV (95%CI): 6.40% (4.80-9.61%)  55% 1RM Reps 1-5 p: 0.009  ES (95%CI): 0.84 (0.19 to 1.49)  r (95%CI): 0.70 (0.38-0.87) SEE (95% CI): 0.06 m/s (0.05-0.10 m/s) CV (95%CI): 8.59% (6.42-13.0%)  Reps 1-10 p: 0.308 ES (95%CI): 0.31 (-0.32 to 0.93)  r (95%CI): 0.72 (0.42-0.88)  SEE (95% CI): 0.05 m/s (0.04-0.08)  CV (95%CI): 7.55% (5.65-11.4%)  70% 1RM Reps 1-5 p: 0.468  ES (95%CI): 0.22 (-0.41 to 0.84)  r (95%CI): 0.79 (0.54-0.91)  SEE (95% CI): 0.06 m/s (0.05-0.09 m/s)  CV (95%CI): 10.1% (7.57-15.4%)  Reps 1-10 p: 0.511 ES (95%CI): -0.20 (-0.82 to 0.43)  r (95%CI): 0.79 (0.53-0.91)  SEE (95% CI): 0.07 m/s (0.05-0.10 m/s) CV (95%CI): 13.2% (9.81-20.1%)  S/M Bench Press  25% 1RM  Reps 1-5  p: 0.271  ES (95%CI): 0.42 (-0.20 to 1.05)  r (95%CI): 0.74 (0.51-0.88)  SEE (95% CI): 0.09 m/s (0.07-0.13 m/s)  CV (95%CI): 8.04% (6.02-12.1%)  Reps 1-10  p: 0.914  ES (95%CI): 0.24 (-0.38 to 0.87)  r (95%CI): 0.80 (0.60-0.90)  SEE (95% CI): 0.09 m/s (0.07-0.13 m/s)  CV (95%CI): 7.97% (5.97-12.0%)  40% 1RM  Reps 1-5  p: 0.008  ES (95%CI): 0.71 (0.08 to 1.35)  SEE (95% CI): 0.07 m/s (0.05-0.11 m/s)  r (95%CI): 0.84 (0.64-0.94)  CV (95%CI): 7.32% (5.48-11.0%)  Reps 1-10  p: 0.060  ES (95%CI): 0.56 (-0.07 to 1.19)  r (95%CI): 0.90 (0.75-0.96)  SEE (95% CI): 0.06 m/s (0.04-0.08 m/s)  CV (95%CI): 5.82% (4.37-8.73%)  55% 1RM  Reps 1-5 p: <0.001  ES (95%CI): 0.92 (0.27 to 1.57)  SEE (95% CI): 0.04 m/s (0.03-0.06 m/s)  r (95%CI): 0.90 (0.76-0.96)  CV (95%CI): 4.79% (3.60-7.16%)  Reps 1-10  p: 0.001  ES (95%CI): 0.83 (0.19 to 1.48)  r (95%CI): 0.86 (0.67-0.94)  SEE (95% CI): 0.05 m/s (0.03-0.07 m/s)  CV (95%CI): 5.38% (4.04-8.06%)  70% 1RM  Reps 1-5  p: 0.001  ES (95%CI): 1.04 (0.38 to 1.70)  r (95%CI): 0.83 (0.62-0.93)  SEE (95% CI): 0.06 m/s (0.05-0.09 m/s)  CV (95%CI): 13.0% (9.66-19.8%)  Reps 1-10  p: 0.009  ES (95%CI): 0.88 (0.23 to 1.53)  r (95%CI): 0.89 (0.74-0.96)  SEE (95% CI): 0.05 m/s (0.04-0.08 m/s)  CV (95%CI): 14.1% (10.5-21.6%) | Was a gold standard criterion used? YES / **NO**  Where the statistics/combination of used to validate the device appropriate? **YES** / NO  Did the original study claim the device was valid? **YES** / NO  Does this device validly measure what was measured? YES / **NO** |
| Pérez-Castilla, Boullosa [106] | LOAD® App via iPhone 8Plus running iOS 12.4.5  (iPhone; Apple, Inc, Cupertino, CA). | T-Force | S/M Back Squat, S/M Bench Press | 25%, 70% 1RM | Mean Velocity | 20 | BACK SQUAT  Power Group r: 0.88 SEE: 7.2%  Strength Group: r: 0.85 SEE: 8.7%  All Subjects: r: 0.87 SEE: 7.8% bias ± random error (R2): -1.6±7.2% (0.18)   BENCH PRESS  Power Group r: 0.87 SEE: 8.5%  Strength Group: r: 0.93 SEE: 5.5%  All Subjects: r: 0.86 SEE: 5.5% bias ± random error (R2): -2.4±10.1% (0.48) | Was a gold standard criterion used? YES / **NO**  Where the statistics/combination of used to validate the device appropriate? YES / **NO**  Did the original study claim the device was valid? **YES** / NO  Does this device validly measure what was measured? YES / **NO** |
| Pueo, Lopez [111] | Novel video system via  Pocophone F1, Xiaomi, Pekin, China) | Chronojump | S/M Back Squat | 75%, 85%, 90%, 95% 1RM | Mean Velocity, Range (Displacement) | 20 | Mean Velocity p: <0.01 ICC: 0.988  Range (Displacement): p: <0.01 ICC: 0.996 | Was a gold standard criterion used? YES / **NO**  Where the statistics/combination of used to validate the device appropriate? YES / **NO**  Did the original study claim the device was valid? **YES** / NO  Does this device validly measure what was measured? YES / **NO** |
| Sánchez-Pay, Courel-Ibáñez [108] | Kinovea (v0.8.15)  via Samsung S6 | T-Force | S/M Bench Press | All loads, High Loads  (MV <0.80 m/s), Low Loads  (MV >0.80 m/s) | Mean Velocity,  Distance, Time | 11 | All loads  Mean Velocity  Bias: 0.10 ± 0.06 m/s;  r: 0.997 Distance  Bias:  1.07 ± 0.65 cm;  r: 0.996 Time  Bias:  - 61.6 ± 36.1 ms;  r: 0.998  High Loads  (MV <0.80 m/s) Mean Velocity  Bias: 0.06 ± 0.05 m/s;  r: 0.986 Distance  Bias: - 0.95 ± 0.69 cm;  r: 0.992 Time  Bias: - 76.6 ± 41.3 ms;  r: 0.997  Low Loads  (MV > 0.80m/s) Mean Velocity  Bias: 0.14 ± 0.06 m/s;  r: 0.985 Distance  Bias: 1.21 ± 0.59 cm;  r: 0.996 Time  Bias: - 44.2 ± 17.2 ms;  r: 0.978 | Was a gold standard criterion used? YES / **NO**  Where the statistics/combination of used to validate the device appropriate? YES / **NO**  Did the original study claim the device was valid? YES / **NO**  Does this device validly measure what was measured? YES / **NO** |
|  | Kinovea (v0.8.15)  via Xiaomi A1 | T-Force | S/M Bench Press | All loads, High Loads  (MV <0.80 m/s), Low Loads  (MV >0.80 m/s) | Mean Velocity,  Distance, Time | 11 | All Loads  Mean Velocity  Bias: 0.09 ± 0.06 m/s;  r: 0.996 Distance  Bias: 1.24 ± 0.45 cm; r: 0.998 Time  Bias: - 57.4 ± 33.4 ms; r: 0.998  High Loads  (MV <0.80 m/s) Mean Velocity  Bias: 0.06 ± 0.03 m/s; r: 0.994 Distance  Bias: 1.21 ± 0.47 cm; r: 0.995 Time  Bias: - 74.3 ± 34.5 ms; r: 0.998  Low Loads  (MV >0.80 m/s) Mean Velocity Bias: 0.13 ± 0.06 ms -1 ; r: 0.981 Distance Bias: 1.38 ± 0.39 cm; r: 0.997 Time  Bias: - 37.6 ± 18.0 ms; r: 0.975 | Was a gold standard criterion used? YES / **NO**  Where the statistics/combination of used to validate the device appropriate? YES / **NO**  Did the original study claim the device was valid? YES / **NO**  Does this device validly measure what was measured? YES / **NO** |
|  | Kinovea (v0.8.15)  via iPhone X | T-Force | S/M Bench Press | All loads, High Loads  (MV <0.80 m/s), Low Loads  (MV >0.80 m/s) | Mean Velocity,  Distance, Time | 11 | All Loads  Mean Velocity  Bias: 0.11 ± 0.08 m/s ; r: 0.994 Distance  Bias: 1.34 ± 0.75 cm; r: 0.993 Time  Bias: - 69.5 ± 34.8 ms; r: 0.998  High Loads  (MV <0.80 m/s) Mean Velocity  Bias: 0.07 ± 0.04 m/s ; r: 0.995 Distance  Bias: 1.29 ± 0.81 cm; r: 0.986 Time  Bias: - 85.6 ± 41.3 ms; r: 0.998  Low Loads  (MV > 0.80 m/s) Mean Velocity  Bias: 0.17 ± 0.09 m/s ; r: 0.977 Distance  Bias: 1.40 ± 0.68 cm;  r: 0.993 Time  Bias: - 50.4 ± 22.0 ms;  r: 0.961 | Was a gold standard criterion used? YES / **NO**  Where the statistics/combination of used to validate the device appropriate? YES / **NO**  Did the original study claim the device was valid? YES / **NO**  Does this device validly measure what was measured? YES / **NO** |
|  | Kinovea (v0.8.15)  via Casio FH20 | T-Force | S/M Bench Press | All loads, High Loads  (MV <0.80 m/s), Low Loads  (MV >0.80 m/s) | Mean Velocity, Distance, Time | 11 | All Loads  Mean Velocity  Bias: 0.14± 0.09 m/s ; r: 0.992 Distance  Bias: 2.48 ± 0.87 cm; r: 0.990 Time  Bias: - 69.5 ± 40.7 ms; r: 0.997  High Loads  (MV <0.80 m/s) Mean Velocity  Bias: 0.08 ± 0.05 m/s ; r: 0.990 Distance  Bias: 2.36 ± 0.91 cm; r: 0.984 Time  Bias: - 83.4 ± 44.3 ms; r: 0.996  Low Loads  (MV >0.80m/s)  Mean Velocity  Bias: 0.20 ± 0.09 m/s; r: 0.963 Distance  Bias: 2.62 ± 0.81 cm;  r: 0.989 Time  Bias: - 53.2 ± 28.9 ms;  r: 0.932 | Was a gold standard criterion used? YES / **NO**  Where the statistics/combination of used to validate the device appropriate? YES / **NO**  Did the original study claim the device was valid? YES / **NO**  Does this device validly measure what was measured? YES / **NO** |
| Sañudo, Rueda [110] | Kinovea (v0.8.15) via Digital Video Camera (50Hz) | T-Force | S/M Bench Press | 20 kg, 30 kg, 40 kg, 50 kg, 60 kg, 70 kg, 80 kg | Mean Propulsive Velocity, Maximal Velocity | 21 | S/M Bench Press  20 kg Mean Propulsive Velocity Bias: - 0.43 m/s Maximal Velocity Bias: - 0.57 m/s  30 kg Mean Propulsive Velocity Bias: - 0.41 m/s Maximal Velocity Bias: - 0.59 m/s   40 kg Mean Propulsive Velocity Bias: - 0.30 m/s Maximal Velocity Bias: - 0.42 m/s   50 kg Mean Propulsive Velocity Bias: - 0.23 m/s Maximal Velocity Bias: - 0.36 m/s   60 kg Mean Propulsive Velocity Bias: - 0.16 m/s Maximal Velocity Bias: - 0.28 m/s  70 kg Mean Propulsive Velocity Bias: - 0.14 m/s Maximal Velocity Bias: - 0.28 m/s  80 kg Mean Propulsive Velocity Bias: - 0.16 m/s Maximal Velocity Bias: - 0.23 m/s | Was a gold standard criterion used? YES / **NO**  Where the statistics/combination of used to validate the device appropriate? YES / **NO**  Did the original study claim the device was valid? **YES** / NO  Does this device validly measure what was measured? YES / **NO** |
| Thompson, Rogerson [55] | MyLift (PowerLift at the time of data collection) | Raptor  3D Motion Capture  (12 Cameras) | F/W Back Squat | 40%, 50%, 60%, 70%, 80%, 90%, 100% 1RM | Mean Velocity, Peak Velocity | 11 | 40% 1RM  Mean Velocity R2: 0.96;  LoA: 0.02 ± 0.06 m/s  50% 1RM  Mean Velocity  R2: 0.94;  LoA: 0.01 ± 0.05 m/s  60% 1RM  Mean Velocity  R2: 0.88;  LoA: 0.01 ± 0.07 m/s  70% 1RM  Mean Velocity  R2: 0.95;  LoA: 0.01 ± 0.04 m/s  80% 1RM  Mean Velocity  R2: 0.93;  LoA: 0.00 ± 0.05 m/s  90% 1RM  Mean Velocity  R2: 0.92;  LoA: 0.00 ± 0.04 m/s  100% 1RM  Mean Velocity  R2: 0.85;  LoA: 0.00 ± 0.06 m/s  Full  Mean Velocity  R2: 0.99;  LoA: 0.01 ± 0.05 m/s | Was a gold standard criterion used? **YES** / NO  Where the statistics/combination of used to validate the device appropriate? YES / **NO**  Did the original study claim the device was valid? **YES** / NO  Does this device validly measure what was measured? YES / **NO** |

*Abbreviations: 1RM: One Repetition Maximum, Bias: Difference between the mean outcome of the device and the criterion measure, CC: Concordance Correlation, CV%: Coefficient of Variation Percent, ES: Effect Size, F/W: Free Weight, ICC: Intraclass Correlation Coefficient, kg: Kilograms, LoA: Limits of Agreement, MARD: Mean Absolute Relative Difference, MD: Mean Difference, m/s: Meters per Second, ms: millisecond, R2: Coefficient of Determination, RMSE: Root Mean Square Error, r: Pearson correlation coefficient, RTE: Relative Typical Error, SEE: Standard Error of Estimate, SEM: Standard Error of Measurement, S/M: Smith Machine, TE: Typical Error.*

S10 Table: Studies investigating the validity of a 3D motion capture analysis or advanced camera system device.

| **Study** | **Device/s** | **Criterion** | **Exercise/s** | **Intensity/Load** | **Variable/s Measured** | **Sample Size** | **Reported Statistics** | **Validity Criteria** |
| --- | --- | --- | --- | --- | --- | --- | --- | --- |
| Weakley, Munteanu [47] | Perch | Vicon 3D  Motion Capture | F/W Back Squat, F/W Bench Press | 20%, 40%, 60%, 80%, 90-100% 1RM | Mean Velocity, Peak Velocity | 16 | F/W Back Squat   All Loads Mean Velocity: R2; 0.96 RMSE; 0.05 Peak Velocity:  R2; 0.97 RMSE; 0.04  20% 1RM Mean Velocity: Mean Bias (95% CL): 0.01 m/s (-0.01 to 0.00 m/s) Peak Velocity: Mean Bias (95% CL): -0.08 m/s (-0.09 to -0.07 m/s)  40% 1RM Mean Velocity: Mean Bias (95% CL): -0.01 m/s (-0.03 to 0.00 m/s) Peak Velocity: Mean Bias (95% CL): -0.09 m/s (-0.10 to -0.08 m/s)  60% 1RM Mean Velocity: Mean Bias (95% CL): -0.01 m/s (-0.02 to 0.01 m/s) Peak Velocity: Mean Bias (95% CL): -0.11 m/s (-0.12 to -0.10 m/s)  80% 1RM Mean Velocity: Mean Bias (95% CL): -0.02 m/s (-0.03 to -0.01 m/s) Peak Velocity: Mean Bias (95% CL): -0.12 m/s (-0.13 to -0.11 m/s)  90-100% 1RM Mean Velocity: Mean Bias (95% CL): -0.01m/s (-0.02 to 0.01 m/s) Peak Velocity: Mean Bias (95% CL): -0.10 m/s (-0.11 to -0.09 m/s)  F/W Bench Press  All Loads Mean Velocity: R2; 0.96 RMSE; 0.05 Peak Velocity:  R2; 0.97 RMSE; 0.04  20% 1RM Mean Velocity: Mean Bias (95% CL):  0.01 m/s (0.01 to 0.02 m/s) Peak Velocity: Mean Bias (95% CL): -0.01 m/s (-0.02 to 0.01 m/s)  40% 1RM Mean Velocity: Mean Bias (95% CL): 0.01 m/s (0.01 to 0.02 m/s) Peak Velocity: Mean Bias (95% CL): -0.02 m/s (-0.03 to -0.01 m/s)  60% 1RM Mean Velocity: Mean Bias (95% CL): 0.01 m/s (0.00 to 0.01 m/s) Peak Velocity: Mean Bias (95% CL): -0.02 m/s (-0.03 to -0.01 m/s)  80% 1RM Mean Velocity: Mean Bias (95% CL): 0.01 m/s (0.01 to 0.02 m/s) Peak Velocity: Mean Bias (95% CL): -0.02 m/s (-0.03 to -0.01 m/s)  90-100% 1RM Mean Velocity: Mean Bias (95% CL): 0.01 m/s (0.00 to 0.01 m/s) Peak Velocity: Mean Bias (95% CL): -0.02 m/s (-0.03 to -0.01 m/s) | Was a gold standard criterion used? **YES** / NO  Where the statistics/combination of used to validate the device appropriate? **YES** / NO  Did the original study claim the device was valid? **YES** / NO  Does this device validly measure what was measured? **YES** / NO |

*Abbreviations: 1RM: One Repetition Maximum, Bias: Difference between the mean outcome of the device and the criterion measure, CC: Concordance Correlation, CL: Confidence Limits, CV%: Coefficient of Variation Percent, ES: Effect Size, F/W: Free Weight, ICC: Intraclass Correlation Coefficient, kg: Kilograms, LoA: Limits of Agreement, MARD: Mean Absolute Relative Difference, MD: Mean Difference, m/s: Meters per Second, R2: Coefficient of Determination, RMSE: Root Mean Square Error, r: Pearson correlation coefficient, RTE: Relative Typical Error, SEE: Standard Error of Estimate, SEM: Standard Error of Measurement, S/M: Smith Machine, TE: Typical Error.*

S11 Table: Studies investigating the validity of an Optic/Laser device.

| **Study** | **Device/s** | **Criterion** | **Exercise/s** | **Intensity/Load** | **Variable/s Measured** | **Sample Size** | **Reported Statistics** | **Validity Criteria** |
| --- | --- | --- | --- | --- | --- | --- | --- | --- |
| Courel-Ibáñez, Martínez-Cava [68] | Velowin | T-Force | S/M Bench Press, S/M Back Squat, S/M Prone Bench Pull | 20 kg, 30 kg, 40 kg, 50 kg, 60 kg, 70 kg, 80 kg | Mean Velocity,  Mean Propulsive Velocity  Peak Velocity | 17 | S/M Bench Press Mean Velocity  SEM:0.02 m/s; CV:3.1%; ICC:0.998  Mean Propulsive Velocity  SEM: 0.03 m/s; CV: 3.5%; ICC: 0.997  Peak Velocity SEM: 0.02 m/s; CV: 1.7%; ICC: 0.999   S/M Back Squat Mean Velocity  SEM: 0.03 m/s; CV: 4.4%; ICC: 0.992   Mean Propulsive Velocity  SEM: 0.03 m/s; CV: 3.6%; ICC: 0.992   Peak Velocity SEM: 0.04 m/s; CV: 2.3%; ICC: 0.993   S/M Prone Bench Pull Mean Velocity  SEM: 0.09 m/s; CV: 8.1%; ICC: 0.967  Mean Propulsive Velocity  SEM: 0.25 m/s; CV: 8.1%; ICC: 0.967   Peak Velocity SEM:0.03 m/s; CV:1.9%; ICC:0.999 | Was a gold standard criterion used? YES / **NO**  Where the statistics/combination of used to validate the device appropriate? **YES** / NO  Did the original study claim the device was valid? **YES** / NO  Does this device validly measure what was measured? YES / **NO** |
| Fritschi, Seiler [51] | Flex | Vantage 5, Vicon 3D Motion Capture | Hang Power Snatch,  CMJ, Squat Jump,  F/W Back Squat | Hang power snatch 20 kg,  CMJ 50% of the load determined for moderate back squat (mean ± standard deviation: 34 ± 10 kg),  Squat Jump 50% of the load determined for moderate back squat (mean ±  standard deviation: 34 ±10 kg),  F/W back squat (Moderate) = (65 ± 20 kg) to elicit Vmean in the range of 0.7-0.8 m/s (mean ± standard deviation of actual values: 0.75 ±  0.05 m/s),  F/W back squat (Heavy) = Heavy back squat with the individual load determined during warm-up (90 ± 20 kg) to elicit Vmean of just under 0.5 m/s (actual values: 0.47 ± 0.05 m/s) | Mean Velocity, Peak Velocity | 14 | Mean Velocity r: 0.96 (0.81-0.94)  SEE: 0.12 m/s (0.02-0.19 m/s) SEEpct: 11.2% (3.6-10.1%)  Peak Velocity r: 0.96 (0.60-0.90) SEE: 0.18 m/s (0.12-0.19 m/s)  SEEpct: 8.6% (5.7-10.5%) | Was a gold standard criterion used? **YES** / NO  Where the statistics/combination of used to validate the device appropriate? YES / **NO**  Did the original study claim the device was valid? **YES** / NO  Does this device validly measure what was measured? YES / **NO** |
| García-Ramos, Pérez-Castilla [67] | Velowin | T-Force | F/W Back Squat | 20 kg, 40 kg, 50 kg, 60 kg, 70 kg | Mean Velocity, Mean Propulsive Velocity,  Maximum Velocity | 31 | Mean Velocity  Bias: 0.02 ± 0.05 m/s; SEE: 0.040 m/s   Mean Propulsive Velocity  Bias: 0.02 ± 0.06 m/s; SEE: 0.055 m/s   Maximum velocity  Bias: -0.09 ± 0.06 m/s; SEE:0.057 m/s | Was a gold standard criterion used? YES / **NO**  Where the statistics/combination of used to validate the device appropriate? YES / **NO**  Did the original study claim the device was valid? **YES** / NO  Does this device validly measure what was measured? YES / **NO** |
| Laza-Cagigas, Goss-Sampson [113] | Velowin | Oqus Infrared cameras and Kistler multi-component force platform | F/W Back Squat | <30-90% 1RM | Barbell Displacement, Mean Velocity, Peak Velocity | 11 | Displacement RMSE:3.73cm; CV:6.6%; ICC:0.84   Mean Velocity RMSE:0.06 m/s; CV:7.3%; ICC:0.97   Peak Velocity RMSE:0.09 m/s; CV:6.5%; ICC:0.96 | Was a gold standard criterion used? **YES** / NO  Where the statistics/combination of used to validate the device appropriate? YES / **NO**  Did the original study claim the device was valid? **YES** / NO  Does this device validly measure what was measured? YES / **NO** |
| Muniz-Pardos, Lozano-Berges [71] | Velowin | T-Force | S/M Half ROM Back Squat, S/M Bench Press | 40%, 60%, 80% 1RM | Mean Propulsive Velocity | 22 | S/M Bench Press MPV 40% 1RM Fixed Bias (95% CI): 0.065 m/s (0.03 to 0.10 m/s) Proportional Bias (95% CI): 0.954 m/s (0.91 to 1.00 m/s)  60% 1RM Fixed Bias (95% CI): 0.010 m/s (-0.01 to 0.03 m/s) Proportional Bias (95% CI): 1.023 m/s (0.99 to 1.05 m/s)  80% 1RM Fixed Bias (95% CI): 0.005 m/s (0.00-0.01 m/s) Proportional Bias (95% CI): 1.028 m/s (1.01 to 1.05 m/s)  S/M Half Squat MPV 40% 1RM Fixed Bias (95% CI): -0.040 m/s (-0.07 to -0.01 m/s) Proportional Bias (95% CI): 1.062 (1.02 to 1.10 m/s)  60% 1RM Fixed Bias (95% CI): -0.013 m/s (-0.04 to 0.01 m/s) Proportional Bias (95% CI): 1.032 m/s (0.99 to 1.07 m/s)  80% 1RM Fixed Bias (95% CI): 0.023 m/s (0.01-0.04 m/s)  Proportional Bias (95% CI): 0.981 m/s (0.95 to 1.01 m/s) | Was a gold standard criterion used? YES / **NO**  Where the statistics/combination of used to validate the device appropriate? YES / **NO**  Did the original study claim the device was valid? **YES** / NO  Does this device validly measure what was measured? YES / **NO** |
| Peña García-Orea, Belando-Pedreño [114] | Velowin | T-Force | S/M Back Squat | 20-70 kg | Mean Velocity, Mean Propulsive Velocity, Peak Velocity | 26 | No significant differences were found between the variances of the two devices | Was a gold standard criterion used? YES / **NO**  Where the statistics/combination of used to validate the device appropriate? YES / **NO**  Did the original study claim the device was valid? **YES** / NO  Does this device validly measure what was measured? YES / **NO** |
| Peña García-Orea, Belando-Pedreño [72] | Velowin | T-Force | Loaded CMJ | 3.5-43.5 kg | Mean Velocity, Peak Velocity | 21 | No significant differences were found between the variances of the two devices | Was a gold standard criterion used? YES / **NO**  Where the statistics/combination of used to validate the device appropriate? YES / **NO**  Did the original study claim the device was valid? **YES** / NO  Does this device validly measure what was measured? YES / **NO** |
| Pérez-Castilla, Piepoli [66] | Velowin | OptiTrack  3D Motion Capture | S/M Bench Press | 45-85% 1RM | Mean Velocity | 14 | Bias: - 0.05± 0.03 m/s r = 0.993 | Was a gold standard criterion used? **YES** / NO  Where the statistics/combination of used to validate the device appropriate? YES / **NO**  Did the original study claim the device was valid? **YES** / NO  Does this device validly measure what was measured? YES / **NO** |
| Weakley, Chalkley [4] | FLEX | Vicon | F/W Back Squat, F/W Bench Press | 20%, 40%, 60%, 80%, > or equal to 90% 1RM | Mean Velocity | 18 | F/W Back Squat  20% 1RM  Mean Velocity  Bias: 0.00± 0.01m/s;  TEE: 0.06 ± 0.02 m/s r: 0.97  40% 1RM  Mean Velocity  Bias: 0.00 ± 0.00 m/s TEE: 0.02 ± 0.008 m/s r: 0.99 ± 0.01  60% 1RM  Mean Velocity  Bias: 0.00 ± 0.00 m/s;  TEE: 0.02 ± 0.008 m/s;  r: 0.97 ± 0.03  80% 1RM  Mean Velocity  Bias: 0.00 ± 0.00m/s;  TEE: 0.02 ± 0.004 m/s;  r: 0.95 ± 0.05  90% 1RM  Mean Velocity  Bias: 0.00 ± 0.00 m/s;  TEE: 0.02 ± 0.004 m/s;  r: 0.99 ± 0.01  Overall  Mean Velocity  Bias: 0.00 ± 0.00 m/s;  TEE: 0.03 ± 0.004 m/s;  r:0.99 ± 0.00 ± 0.03  F/W Bench Press  20% 1RM  Mean Velocity  Bias: - 0.01 ± 0.03 m/s;  TEE: 0.08 ± 0.04 m/s;  r: 0.97 ± 0.04  40% 1RM  Mean Velocity  Bias: - 0.02 ± 0.06 m/s;  TEE: 0.04 ± 0.02 m/s;  r: 0.99 ± 0.02  60% 1RM  Mean Velocity  Bias: 0.00 ± 0.01 m/s;  TEE: 0.02 ± 0.04 m/s;  r: 0.98 ± 0.02  80% 1RM  Mean Velocity  Bias: 0.00 ± 0.00 m/s;  TEE: 0.01 ± 0.005 m/s; r: 0.99 ± 0.01  90% 1RM  Mean Velocity  Bias: 0.00 ± 0.00 m/s;  TEE: 0.02 ± 0.005 m/s;  r: 0.98 ± 0.01  Overall  Mean Velocity  Bias: - 0.01 ± 0.01 m/s;  TEE: 0.04 ± 0.005 m/s; r: 0.99 ± 0.00 | Was a gold standard criterion used? **YES** / NO  Where the statistics/combination of used to validate the device appropriate? **YES** / NO  Did the original study claim the device was valid? **YES** / NO  Does this device validly measure what was measured? **YES** / NO |

*Abbreviations: CL: Confidence Level, CMJ: Countermovement Jump, CV: Coefficient of Variation, F/W: Free Weight, ICC: Intraclass Correlation Coefficient, kg: Kilograms, m/s: Meters per Second, MPV: Mean Propulsive Velocity, RMSE: Root Mean Square Error, r: Pearson correlation coefficient, SEE: Standard Error of Estimate, SEEpct: Standard Error of Estimate Percentage, SEM: Standard Error of Measurement, S/M: Smith Machine, TEE: Typical Error of Estimate.*

S12 Table: Studies investigating the reliability of a Linear Position Transducer device.

| **Study** | **Device/s** | **Type of Reliability** | **Exercise/s** | **Intensity/Load** | **Variable/s Measured** | **Sample Size** | **Reported Statistics** | **Reliability Criteria** |
| --- | --- | --- | --- | --- | --- | --- | --- | --- |
| Appleby, Banyard [45] | GymAware | Inter-device  vs  criterion marker | F/W Back Squat | 70-90% 1RM | Barbell Displacement | 12 | mean range (mm) + (90% confidence limit):   529 (56)   CV + (CL): 6.6% (5.5-8.4)  ICC + (CL): 0.67 (0.46-0.82) | Was both biological and technological reliability reported? YES / **NO**  Where the statistics/combination of used to validate the device appropriate? **YES** / NO  Did the original study claim the device was reliable? **YES** / NO  Does this device reliably measure what was measured? YES / **NO** |
| Askow, Stone [49] | GymAware | Intra-device | F/W Back Squat | 75-90% 1RM | Mean Velocity, Peak Velocity | 9 | Mean Velocity Mean Bias: 0.03 m/s ICC: 0.966 ES: 0.28 SEE: 0.04 m/s  Peak Velocity  Mean Bias: -0.12 m/s ICC: 0.982;  ES: - 0.57 SEE: 0.05 m/s | Was both biological and technological reliability reported? YES / **NO**  Where the statistics/combination of used to validate the device appropriate? **YES** / NO  Did the original study claim the device was reliable? **YES** / NO  Does this device reliably measure what was measured? **YES** / NO |
| Balsalobre-Fernández, Marchante [78] | SmartCoach | Intra-device | F/W Back Squat, F/W Bench Press, F/W Hip Thrust | 50-95% 1RM | Mean Velocity | 10 | F/W Back Squat  50-95% 1RM  Mean Velocity  ICC: 0.981  F/W Bench Press  50-95% 1RM  Mean Velocity  ICC: 0.981  F/W Hip Thrust  50-95% 1RM  Mean Velocity  ICC: 0.966 | Was both biological and technological reliability reported? YES / **NO**  Where the statistics/combination of used to validate the device appropriate? YES / **NO**  Did the original study claim the device was reliable? **YES** / NO  Does this device reliably measure what was measured? YES / **NO** |
| Beckham, Layne [56] | GymAware | Intra-device | F/W Back Squat | 45%, 60%, 75% 1RM | Mean Velocity, Peak Velocity | 16 | 45% 1RM  Mean Velocity  ICC: 0.774 Peak Velocity ICC: 0.793  60% 1RM  Mean Velocity  ICC: 0.752 Peak Velocity ICC: 0.775  75% 1RM  Mean Velocity ICC: 0.651 Peak Velocity ICC: 0.761 | Was both biological and technological reliability reported? YES / **NO**  Where the statistics/combination of used to validate the device appropriate? YES / **NO**  Did the original study claim the device was reliable? **YES** / NO  Does this device reliably measure what was measured? YES / **NO** |
| Boehringer and Whyte [79] | 1080Q | Intra-Device | S/M Bench Press | 30-80% 1RM | Mean Velocity,  Peak Velocity | 27 | Mean Velocity 30-80% 1RM MD: 0.004 m/s; CV: 7.0%; ICC: 0.97   30% 1RM MD: -0.003 m/s; CV: 4.5%; ICC: 0.64   40% 1RM MD: 0.001 m/s; CV: 3.7% ICC: 0.82   50% 1RM MD: 0.010 m/s; CV: 5.7% ICC: 0.53   60% 1RM MD: 0.017 m/s; CV: 6.5 ICC: 0.45   70% 1RM MD: 0.000 m/s; CV: 7.6% ICC: 0.63   80% 1RM MD: -0.003 m/s; CV: 11.0% ICC: 0.69   Mean Force 30-80% 1RM MD: -0.3 N; CV: 1.7%; ICC: 1.00   30% 1RM MD:-4.1N; CV:2.0%; ICC:0.93   40% 1RM MD: -0.9 N; CV: 1.9%; ICC: 0.98   50% 1RM MD: 3.4N; CV: 2.3%; ICC: 0.96   60% 1RM MD: 3.2N; CV: 1.7%; ICC: 0.98   70% 1RM MD: -4.8N; CV: 1.1%; ICC: 0.99   80% 1RM MD: -0.9N; CV: 0.9%; ICC: 0.99    Peak Velocity  30-80% 1RM MD: 0.002 m/s; CV: 6.3%; ICC: 0.97   30% 1RM MD: 0.002 m/s; CV: 4.1%; ICC: 0.61   40% 1RM MD: 0.001 m/s; CV: 3.6%; ICC: 0.84   50% 1RM MD: 0.008 m/s; CV: 5.0%; ICC: 0.68   60% 1RM MD: 0.000 m/s; CV: 5.0%; ICC: 0.75   70% 1RM MD: 0.004 m/s; CV: 8.2%; ICC: 0.58   80% 1RM MD: -0.001 m/s; CV: 9.5%; ICC: 0.80 | Was both biological and technological reliability reported? YES / **NO**  Where the statistics/combination of used to validate the device appropriate? **YES** / NO  Did the original study claim the device was reliable? **YES** / NO  Does this device reliably measure what was measured? YES / **NO** |
| Callaghan, Guy [83] | Speed4Lifts | Test-Retest | F/W Back Squat, F/W Front Squat, F/W Bench Press | 20%, 40%, 60%, 80% 1RM | Mean Velocity | 20 | Back Squat Load: 20% 1RM ICC: 0.75 ICC Interpretation: High ICC 95% CI: 0.37 to 0.90 SEM: 0.04 m/s MDC: 0.11 m/s CV: 3.51% CV Interpretation: Good CV 95% CI: 1.76 to 5.26%  Load: 40% 1RM ICC: 0.79 ICC Interpretation: High ICC 95% CI: 0.46 to 0.91 SEM: 0.03 m/s MDC: 0.08 m/s CV: 3.28% CV Interpretation: Good CV 95% CI: 1.57 to 4.98%  Load: 60% 1RM ICC: 0.88 ICC Interpretation: High ICC 95% CI: 0.62 to 0.96 SEM: 0.01 m/s MDC: 0.04 m/s CV: 2.80% CV Interpretation: Good CV 95% CI: 1.50 to 4.10%  Load: 80% 1RM ICC: 0.88 ICC Interpretation: High ICC 95% CI: 0.70 to 0.95 SEM: 0.01 m/s MDC: 0.04 m/s CV: 3.6% CV Interpretation: Good CV 95% CI: 1.74 to 5.47%  Front Squat Load: 20% 1RM ICC: 0.70 ICC Interpretation: High ICC 95% CI: 0.27 to 0.88 SEM: 0.05 m/s MDC: 0.13 m/s CV: 4.26% CV Interpretation: Good CV 95% CI: 3.07 to 5.45%  Load: 40% 1RM ICC: 0.74 ICC Interpretation: High ICC 95% CI: 0.34 to 0.90 SEM: 0.03 m/s MDC: 0.09 m/s CV: 3.47% CV Interpretation: Good CV 95% CI: 2.05 to 4.90%  Load: 60% 1RM ICC: 0.72 ICC Interpretation: High ICC 95% CI: 0.33 to 0.89 SEM: 0.03 m/s MDC: 0.08 m/s CV: 3.07% CV Interpretation: Good CV 95% CI: 1.55 to 4.59%  Load: 80% 1RM ICC: 0.91 ICC Interpretation: Very High ICC 95% CI: 0.77 to 0.96 SEM: 0.01 m/s MDC: 0.03 m/s CV: 2.57% CV Interpretation: Good CV 95% CI: 1.33 to 3.82%  Bench Press Load: 20% 1RM ICC: 0.59 ICC Interpretation: Moderate ICC 95% CI: 0.00 to 0.84 SEM: 0.08 m/s MDC: 0.22 m/s CV: 4.59% CV Interpretation: Good CV 95% CI: 2.58 to 6.60%  Load: 40% 1RM ICC: 0.86 ICC Interpretation: High ICC 95% CI: 0.64 to 0.94 SEM: 0.03 m/s MDC: 0.08 m/s CV: 3.61% CV Interpretation: Good CV 95% CI: 2.19 to 5.02%  Load: 60% 1RM ICC: 0.92 ICC Interpretation: Very High ICC 95% CI: 0.81 to 0.97 SEM: 0.01 m/s MDC: 0.03 m/s CV: 2.71% CV Interpretation: Good CV 95% CI (%): 1.47 to 3.96  Load: 80% 1RM ICC: 0.96 ICC Interpretation: Very High ICC 95% CI: 0.91 to 0.99 SEM: 0.01 m/s MDC: 0.02 m/s CV: 3.33% CV Interpretation: Good CV 95% CI: 2.08 to 4.57% | Was both biological and technological reliability reported? YES / **NO**  Where the statistics/combination of used to validate the device appropriate? **YES** / NO  Did the original study claim the device was reliable? **YES** / NO (Except for 20%RM BP)  Does this device reliably measure what was measured? YES / **NO** (Except for BP at 80%RM) |
| Courel-Ibáñez, Martínez-Cava [68] | T-Force | Inter-device | S/M Bench Press, S/M Back Squat, S/M Prone Bench Pull | 20 kg, 30 kg, 40 kg, 50 kg, 60 kg, 70 kg, 80 kg | Mean Velocity,  Mean Propulsive Velocity,  Peak Velocity | 17 | S/M Bench Press Mean Velocity  SEM: 0.01 m/s;  CV: 1.4%;  ICC: 1.00   Mean Propulsive Velocity  SEM: 0.01 m/s;  CV: 1.3%;  ICC: 1.00   Peak Velocity SEM: 0.01 m/s;  CV: 0.6%;  ICC: 1.00   S/M Back Squat Mean Velocity  SEM: 0.01 m/s;  CV: 1.0%;  ICC: 0.999   Mean Propulsive Velocity  SEM: 0.01 m/s;  CV: 1.1%;  ICC: 0.999   Peak Velocity SEM: 0.01 m/s;  CV: 0.8%;  ICC: 0.999   S/M Prone Bench Pull Mean Velocity  SEM: 0.02 m/s;  CV: 2.1%;  ICC: 0.998   Mean Propulsive Velocity  SEM: 0.02 m/s;  CV: 1.9%;  ICC: 0.998    Peak Velocity SEM: 0.01 m/s;  CV: 0.8%;  ICC: 1.000 | Was both biological and technological reliability reported? YES / **NO**  Where the statistics/combination of used to validate the device appropriate? **YES** / NO  Did the original study claim the device was reliable? **YES** / NO  Does this device reliably measure what was measured? **YES** / NO |
|  | Chronojump | Inter-device | S/M Bench Press, S/M Back Squat, S/M Prone Bench Pull | 20 kg, 30 kg, 40 kg, 50 kg, 60 kg, 70 kg, 80 kg | Mean Velocity, Mean Propulsive Velocity,  Peak Velocity | 17 | S/M Bench Press Mean Velocity  SEM: 0.04 m/s;  CV: 4.7%;  ICC: 0.995   Mean Propulsive Velocity  SEM: 0.04 m/s;  CV: 5.2%;  ICC: 0.995   Peak Velocity SEM: 0.02 m/s;  CV: 1.4%;  ICC: 1.000  S/M Back Squat Mean Velocity  SEM: 0.03 m/s;  CV: 3.6%;  ICC: 0.991   Mean Propulsive Velocity  SEM: 0.03 m/s;  CV: 3.9%;  ICC: 0.991   Peak Velocity SEM: 0.03 m/s;  CV: 1.8%; ICC: 0.996  S/M Prone Bench Pull Mean Velocity  SEM: 0.04 m/s;  CV: 3.3%;  ICC: 0.995   Mean Propulsive Velocity  SEM: 0.04 m/s;  CV: 3.4%;  ICC: 0.995    Peak Velocity SEM: 0.04 m/s;  CV: 2.4%;  ICC: 1.00 | Was both biological and technological reliability reported? YES / **NO**  Where the statistics/combination of used to validate the device appropriate? **YES** / NO  Did the original study claim the device was reliable? **YES** / NO  Does this device reliably measure what was measured? **YES** / NO |
|  | T-Force | Intra-Device | S/M Bench Press, S/M Back Squat, S/M Prone Bench Pull | 20 kg, 30 kg, 40 kg, 50 kg, 60 kg, 70 kg, 80 kg | Mean Velocity,  Mean Propulsive Velocity, Peak Velocity | 17 | S/M Bench Press Mean Velocity  SEM: 0.02 m/s;  CV: 1.9%;  ICC: 0.999   Mean Propulsive Velocity  SEM: 0.02 m/s;  CV: 1.8%;  ICC: 0.999   Peak Velocity SEM: 0.03 m/s;  CV: 2.0%;  ICC: 0.999  S/M Back Squat Mean Velocity  SEM: 0.03 m/s;  CV: 2.5%;  ICC: 0.995   Mean Propulsive Velocity  SEM: 0.02 m/s;  CV: 2.6%;  ICC: 0.996   Peak Velocity SEM: 0.05 m/s;  CV: 2.9%;  ICC: 0.989  S/M Prone Bench Pull Mean Velocity  SEM: 0.04 m/s;  CV: 3.0%;  ICC: 0.995   Mean Propulsive Velocity  SEM: 0.03 m/s;  CV: 3.0%;  ICC: 0.995    Peak Velocity SEM: 0.03 m/s;  CV: 1.8%;  ICC: 0.999 | Was both biological and technological reliability reported? YES / **NO**  Where the statistics/combination of used to validate the device appropriate? **YES** / NO  Did the original study claim the device was reliable? **YES** / NO  Does this device reliably measure what was measured? **YES** / NO |
|  | Chronojump | Intra-Device | S/M Bench Press, S/M Back Squat, S/M Prone Bench Pull | 20 kg, 30 kg, 40 kg, 50 kg, 60 kg, 70 kg, 80 kg | Mean Velocity,  Mean Propulsive Velocity, Peak Velocity | 17 | S/M Bench Press Mean Velocity  SEM: 0.04 m/s;  CV: 4.3%;  ICC: 0.997   Mean Propulsive Velocity  SEM: 0.03 m/s;  CV: 3.2%;  ICC: 0.998   Peak Velocity SEM: 0.04 m/s;  CV: 2.4%;  ICC: 0.999  S/M Back Squat Mean Velocity  SEM: 0.04 m/s;  CV: 3.9%;  ICC: 0.990   Mean Propulsive Velocity  SEM: 0.04 m/s;  CV: 3.8%;  ICC: 0.991    Peak Velocity SEM: 0.06 m/s;  CV: 3.4%;  ICC: 0.985  S/M Prone Bench Pull Mean Velocity  SEM: 0.07 m/s;  CV: 5.2%;  ICC: 0.990   Mean Propulsive Velocity  SEM: 0.07 m/s;  CV: 5.4%;  ICC: 0.987    Peak Velocity SEM: 0.04 m/s;  CV: 2.3%;  ICC: 0.998 | Was both biological and technological reliability reported? YES / **NO**  Where the statistics/combination of used to validate the device appropriate? **YES** / NO  Did the original study claim the device was reliable? **YES** / NO  Does this device reliably measure what was measured? **YES** / NO |
| Dorrell, Moore [50] | GymAware | Intra-device | F/W Back Squat,  F/W Bench Press,  F/W Deadlift | 80% 1RM | Barbell Displacement,  Peak Velocity, Mean Velocity | 13 | F/W Back Squat  80% 1RM  Bar displacement  Mean TE: 3.8% (3.0-5.3%) Peak Velocity  Mean TE: 8.1% (6.4-11.5%) Mean Velocity  Mean TE: 7.0% (5.6-10.0%)  F/W Bench Press  80% 1RM  Bar displacement  Mean TE: 3.0% (2.3-4.1) Peak Velocity  Mean TE: 6.2% (4.9-8.7) Mean Velocity  Mean TE: 7.4% (5.8-10.5%)  F/W Deadlift  80% 1RM  Bar displacement  Mean TE: 2.0% (1.6-2.7%) Peak Velocity  Mean TE: 8.8% (7.0-12.5%) Mean Velocity  Mean TE: 7.0% (5.5-9.8%) | Was both biological and technological reliability reported? YES / **NO**  Where the statistics/combination of used to validate the device appropriate? YES / **NO**  Did the original study claim the device was reliable? **YES** / NO  Does this device reliably measure what was measured? YES / **NO** |
| Fernandes, Lamb [80] | FitroDyne  (fitronic) | Intra-device (intraday) | S/M Bench Press, S/M Bench Press, S/M Bent Over Row | 20%, 30%, 40%, 50%, 60%, 70%, 80% 1RM | Peak Velocity,  Mean Velocity | 15 | S/M BENCH PRESS 20% 1RM Peak Velocity  TE: 6.9 cm/s; CV: 3.3%  Mean Velocity  TE: 3.4 cm/s; CV: 2.9%   30% 1RM Peak Velocity  TE: 3.1 cm/s; CV: 1.7% Mean Velocity TE: 2.9 cm/s; CV: 2.7%    40% 1RM Peak Velocity  TE: 2.8 cm/s; CV: 1.8% Mean Velocity TE: 2.9 cm/s; CV: 3.1%   50% 1RM Peak Velocity  TE: 3.6 cm/s; CV: 2.8%  Mean Velocity TE: 2.2 cm/s; CV: 2.8%   60% 1RM Peak Velocity  TE: 3.8 cm/s; CV: 3.9% Mean Velocity TE: 2.8 cm/s; CV: 4.4%  70% 1RM Peak Velocity  TE: 2.6 cm/s; CV: 3.3%  Mean Velocity TE: 2.2 cm/s; CV: 4.5%  80% 1RM Peak Velocity  TE: 5.3 cm/s; CV: 9.7% Mean Velocity TE: 4.3 cm/s; CV: 13.4%  S/M BACK SQUAT 20% 1RM Peak Velocity  TE: 7.0 cm/s; CV: 4.1%  Mean Velocity TE: 3.9 cm/s; CV: 4.0%  30% 1RM Peak Velocity  TE: 7.4 cm/s; CV: 4.5%  Mean Velocity TE: 4.2 cm/s; CV: 4.6%  40% 1RM Peak Velocity  TE: 6.1 cm/s; CV: 4.0%  Mean Velocity TE: 4.8 cm/s; CV: 5.7%  50% 1RM Peak Velocity  TE: 3.9 cm/s; CV: 2.7%  Mean Velocity TE: 2.4 cm/s; CV: 3.2%   60% 1RM Peak Velocity  TE: 4.5 cm/s; CV: 3.3% Mean Velocity TE: 3.0 cm/s; CV: 4.3%  70% 1RM Peak Velocity  TE: 6.4 cm/s; CV: 5.2%  Mean Velocity TE: 3.8 cm/s; CV: 6.3%  80% 1RM Peak Velocity  TE: 5.1 cm/s; CV: 4.4%  Mean Velocity TE: 3.1 cm/s; CV: 6.4%  S/M BENT OVER ROW 20% 1RM Peak Velocity  TE: 8.8 cm/s; CV: 4.4% Mean Velocity TE: 6.6 cm/s; CV: 5.6%   30% 1RM Peak Velocity  TE: 7.1 cm/s; CV: 3.8%  Mean Velocity TE: 8.7 cm/s; CV: 7.9%  40% 1RM Peak Velocity  TE: 6.1 cm/s; CV: 3.7%  Mean Velocity TE: 4.8 cm/s; CV: 4.7%   50% 1RM Peak Velocity  TE: 4.0 cm/s; CV: 2.6%  Mean Velocity TE: 6.0 cm/s; CV: 6.4%  60% 1RM Peak Velocity  TE: 5.7 cm/s; CV: 4.0%  Mean Velocity TE: 6.4 cm/s; CV: 7.5%   70% 1RM Peak Velocity  TE: 10.5 cm/s; CV: 8.5%  Mean Velocity TE: 6.7 cm/s; CV: 9.0%   80% 1RM Peak Velocity  TE: 8.8 cm/s; CV: 8.3%  Mean Velocity TE: 5.4 cm/s; CV: 8.5% | Was both biological and technological reliability reported? YES / **NO**  Where the statistics/combination of used to validate the device appropriate? YES / **NO**  Did the original study claim the device was reliable? YES / **NO**  Does this device reliably measure what was measured? YES / **NO** |
| Feuerbacher, Jacobs [74] | T-Force | Intra-day Inter-day | F/W Back Squat | 30%, 50%, 70%, 90% 1RM | Mean Velocity | 19 | Intra-day Reliability  30%RM  ICC(p): 00.832–0.937 (<0.001) 50%RM  ICC(p): 0.864–0.934 (<0.001) 70%RM  ICC(p): 0.874–0.955 (<0.001) 90%RM  ICC(p): 0.868–0.956 (<0.001)  Inte-rday Reliability 30%RM  ICC 0.889 (<0.001) 95% CI: 0.737 to 0.960 50%RM  ICC 0.820 (<0.001) 95% CI: 0.571 to 0.934 70%RM  ICC 0.792 (<0.001) 95% CI: 0.506 to 0.924 90%RM  ICC 0.901 (<0.001) 95% CI: 0.764 to 0.964 | Was both biological and technological reliability reported? YES / **NO**  Where the statistics/combination of used to validate the device appropriate? **YES** / NO  Did the original study claim the device was reliable? **YES** / NO  Does this device reliably measure what was measured? YES / **NO** |
| García-Ramos, Pérez-Castilla [67] | T-Force | Intra-device | F/W Back Squat | 20 kg, 40 kg, 50 kg, 60 kg, 70 kg | Mean Velocity, Mean Propulsive Velocity, Maximum Velocity | 31 | 20 kg Load  Mean Velocity SEM: 0.052 m/s;  CV: 4.65%; ICC: 0.90  Mean Propulsive Velocity SEM: 0.059 m/s;  CV: 4.87%; ICC: 0.91  Maximum velocity SEM: 0.075 m/s;  CV: 4.17%; ICC: 0.93  40 kg Load  Mean Velocity SEM: 0.040 m/s;  CV: 4.19%; ICC: 0.93  Mean Propulsive Velocity SEM: 0.046 m/s;  CV: 4.46%; ICC: 0.92  Maximum velocity SEM: 0.058 m/s;  CV: 3.59%; ICC: 0.95  50 kg Load Mean Velocity SEM: 0.038 m/s;  CV: 4.25%; ICC: 0.90  Mean Propulsive Velocity SEM: 0.047 m/s;  CV: 4.83%; ICC: 0.87   Maximum velocity SEM: 0.044 m/s;  CV: 2.84%; ICC: 0.95  60 kg Load Mean Velocity SEM: 0.031 m/s;  CV: 3.75%; ICC: 0.94   Mean Propulsive Velocity SEM: 0.033 m/s;  CV: 3.73%; ICC: 0.95   Maximum velocity SEM: 0.049 m/s;  CV: 3.35%; ICC: 0.94  70 kg Load Mean Velocity SEM: 0.036 m/s;  CV: 4.84%; ICC: 0.92   Mean Propulsive Velocity SEM: 0.036 m/s;  CV: 4.49%; ICC: 0.93 | Was both biological and technological reliability reported? YES / **NO**  Where the statistics/combination of used to validate the device appropriate? **YES** / NO  Did the original study claim the device was reliable? **YES** / NO  Does this device reliably measure what was measured? **YES** / NO |
| García-Pinillos, Latorre-Román [69] | T-Force | Intra-device | S/M Concentric-Only Half ROM Back Squat | 10-100% 1RM | Mean Velocity, Mean Propulsive Velocity | 19 | 10% 1RM  Mean Velocity  CV: 8.60%; SEM: 0.03 m/s Mean propulsive velocity CV: 11.28%; SEM: 0.04 m/s Maximum velocity  CV: 9.18%; SEM: 0.06 m/s  20% 1RM  Mean Velocity  CV: 6.76%; SEM: 0.02 m/s Mean propulsive velocity CV: 8.29%; SEM: 0.03 m/s Maximum velocity  CV: 8.17%; SEM: 0.05 m/s  30% 1RM  Mean Velocity  CV: 11.86%; SEM: 0.11 m/s Mean propulsive velocity CV: 14.39%; SEM: 0.04 m/s Maximum velocity  CV: 7.42%; SEM: 0.04 m/s  40% 1RM  Mean Velocity  CV: 9.95%; SEM: 0.09 m/s Mean propulsive velocity CV: 10.87%; SEM: 0.03 m/s Maximum velocity  CV: 6.64%; SEM: 0.03 m/s  50% 1RM  Mean Velocity CV: 9.06%; SEM: 0.07 m/s Mean propulsive velocity CV: 11.21%; SEM: 0.02 m/s Maximum velocity  CV: 6.49%; SEM: 0.03 m/s  60% 1RM  Mean Velocity CV: 9.27%; SEM: 0.02 m/s Mean propulsive velocity CV: 13.81%; SEM: 0.03 m/s Maximum velocity  CV: 9.91%; SEM: 0.04 m/s  70% 1RM  Mean Velocity CV: 9.50%; SEM: 0.02 m/s Mean propulsive velocity CV: 12.27%; SEM: 0.02 m/s Maximum velocity  CV: 8.68%; SEM: 0.03 m/s  80% 1RM  Mean Velocity CV: 9.09%; SEM: 0.02 m/s Mean propulsive velocity CV: 9.07%; SEM: 0.02 m/s Maximum velocity CV: 5.67%; SEM: 0.02 m/s  90% 1RM  Mean Velocity CV: 11.02%; SEM: 0.05 m/s Mean propulsive velocity CV: 11.00%; SEM: 0.02 m/s Maximum velocity  CV: 7.73%; SEM: 0.02 m/s  100% 1RM  Mean Velocity  CV: 16.77%; SEM: 0.02 m/s Mean propulsive velocity CV: 17.26%; SEM: 0.02 m/s Maximum velocity  CV: 9.79%; SEM: 0.02 m/s | Was both biological and technological reliability reported? YES / **NO**  Where the statistics/combination of used to validate the device appropriate? YES / **NO**  Did the original study claim the device was reliable? **YES** / NO  Does this device reliably measure what was measured? YES / **NO** |
| Garnacho-Castaño, López-Lastra [60] | Tendo | Intra-device | S/M Back Squat, S/M Bench press | 40-60 kg  (30-90% 1RM) | Mean Velocity,  Peak Velocity, | 32 | S/M Back Squat 40-60 kg  Mean Velocity Bias: -0.02 ± 0.07 m/s; CV: 8.5%  Peak Velocity Bias: -0.05 ± 0.13 m/s; CV: 9.6%   S/M Bench Press 40-60 kg  Mean Velocity  Bias: 0.001 ± 0.08 m/s; CV: 9.6%  Peak Velocity Bias: -0.004 ± 0.08 m/s; CV: 9.0% | Was both biological and technological reliability reported? YES / **NO**  Where the statistics/combination of used to validate the device appropriate? YES / **NO**  Did the original study claim the device was reliable? **YES** / NO  Does this device reliably measure what was measured? YES / **NO** |
| Gomez-Piriz, Sanchez [75] | T-Force | Inter-device | F/W Bench Press | 25 kg | Maximum Velocity | 3 | Statistics Reported Unclear | Was both biological and technological reliability reported? YES / **NO**  Where the statistics/combination of used to validate the device appropriate? YES / **NO**  Did the original study claim the device was reliable? YES / **NO**  Does this device reliably measure what was measured? YES / **NO** |
| Jovanovic and Jukic [57] | GymAware | Inter-device  (Left and Right) | Hex Bar Deadlift | 40%, 60%, 80%, 90%,  100% 1RM | Peak Velocity, Mean Velocity | 12 | MV fixed bias: Intercept = -0.009 m/s, 95% CI (-0.013 to -0.005) PV fixed bias: Intercept = -0.009 m/s, 95% CI (-0.014 to -0.004)  MV SESOI: ±0.031 m/s, 95% CI (0.029–0.033) PV SESOI: ±0.045 m/s, 95% CI (0.042–0.048)  MV slope: 1.003, 95% CI (0.996–1.011) PV slope: 1.002, 95% CI (0.997–1.008)  MV SDC: 0.037 m/s, 95% CI (0.035–0.041) PV SDC: 0.040 m/s, 95% CI (0.038–0.044)  MV SDC% 1RM: 2.679%, 95% CI (2.451–2.964) PV SDC% 1RM: 1.795% 1RM, 95% CI (1.642–1.979) | Was both biological and technological reliability reported? YES / **NO**  Where the statistics/combination of used to validate the device appropriate? **YES** / NO  Did the original study claim the device was reliable? **YES** / NO  Does this device reliably measure what was measured? YES / **NO** |
| Held, Rappelt [84] | Speed4Lifts | Within-day | F/W Back Squat, F/W Hip Thrust | 75% 1RM | Mean Velocity, Barbell Displacement | 19 | F/W Back Squat  MCV TE (%); 3.3 CV(%); 4.4 ICC (95% CI); 0.91 (0.78-0.97) CC (95% CI); 0.92 (0.84-0.98) LoA; 0.04 m/s SEM; 0.01 m/s  Barbell Displacement TE (%); 3.8 CV(%); 5.5 ICC (95% CI); 0.94 (0.85-0.98) CC (95% CI); 0.94 (0.84-0.98 LoA; 6.58 cm SEM; 0.82 cm  F/W Hip Thrust  MCV TE (%); 3.3 CV(%); 4.5 ICC (95% CI); 0.93 (0.83-0.97) CC (95% CI); 0.93 (0.83-0.97) LoA; 5.07 m/s SEM; 1.4 m/s  Barbell Displacement TE (%); 4.8 CV(%); 6.9 ICC (95% CI); 0.71 (0.39-0.88) CC (95% CI); 0.71 (0.37-0.88) LoA; 5.07 cm SEM; 1.4 cm | Was both biological and technological reliability reported? YES / **NO**  Where the statistics/combination of used to validate the device appropriate? **YES** / NO  Did the original study claim the device was reliable? **YES** / NO  Does this device reliably measure what was measured? **YES** / NO |
|  | Speed4Lifts | Between- day | F/W Back Squat, F/W Hip Thrust | 75% 1RM | Mean Velocity, Barbell Displacement | 19 | F/W Back Squat  MCV TE (%); 8.6 CV(%); 12.3 ICC (95% CI); 0.75 (0.56-0.87) CC (95% CI); 0.75 (0.85-0.87) LoA; 0.21 m/s SEM; 0.05 m/s  Barbell Displacement  TE (%); 6.7 CV(%); 9.3 ICC (95% CI); 0.78 (0.61-0.89) CC (95% CI); 0.93 (0.83-0.88) LoA; 5.07 cm SEM; 1.4 cm  F/W Hip Thrust  MCV  TE (%); 12 CV(%); 15.9 ICC (95% CI); 0.56 (0.22-0.74) CC (95% CI); 0.56 (0.26-0.76) LoA; 0.2 m/s SEM; 0.72 m/s  Barbell Displacement TE (%); 10.3  CV(%); 13.9 ICC (95% CI); 0.49 (0.19-0.71) CC (95% CI); 0.52 (0.208-0.735) LoA; 10.68 cm SEM; 3.87 cm | Was both biological and technological reliability reported? YES / **NO**  Where the statistics/combination of used to validate the device appropriate? **YES** / NO  Did the original study claim the device was reliable? **YES** / NO  Does this device reliably measure what was measured? YES / **NO** |
| Janicijevic, García-Ramos [52] | GymAware | Intra-device | F/W Back Squat | 45% 1RM (Light), 65% 1RM (Medium), 85% 1RM (Heavy) | Mean Velocity,  Maximum Velocity, Left and Right Placement conditions of LPT | 20 | Mean Velocity  45% 1RM (Light)  Placement: LEFT  Block 1: 0.96 ± 0.08 m/s  Block 2: 0.95 ± 0.07 m/s  CV: 3.84% (95% CI: 2.92-5.61%)  ICC: 0.78 (95% CI: 0.52-0.91)  Placement: RIGHT  Block 1: 0.93 ± 0.09 m/s  Block 2: 0.93 ± 0.05 m/s  CV: 3.49% (95% CI: 2.66-5.10%)  ICC: 0.69 (95% CI: 0.36-0.86)  65% 1RM  (Medium)  Placement: LEFT  Block 1: 0.74 ± 0.08 m/s  Block 2: 0.73 ± 0.07 m/s  CV: 4.89% (95% CI: 3.72, 7.15)  ICC: 0.77 (95% CI: 0.51, 0.90)  Placement: RIGHT  Block 1: 0.73 ± 0.07 m/s  Block 2: 0.71 ± 0.10 m/s  CV: 5.05% (95% CI: 3.84, 7.37)  ICC: 0.82 (95% CI: 0.61, 0.93)  85% 1RM (Heavy)  Placement: LEFT  Block 1: 0.53 ± 0.09 m/s  Block 2: 0.53 ± 0.10 m/s  CV: 9.57% (95% CI: 7.23, 14.16)  ICC: 0.76 (95% CI: 0.48, 0.90)  Placement: RIGHT  Block 1: 0.54 ± 0.09 m/s  Block 2: 0.51 ± 0.11 m/s  CV: 9.71% (95% CI: 7.38, 14.18)  ICC: 0.76 (95% CI: 0.48, 0.90)  MAX Velocity  45% 1RM (Light)  Placement: LEFT  Block 1: 1.52 ± 0.12 m/s  Block 2: 1.54 ± 0.12 m/s  CV: 4.03% (95% CI: 3.07, 5.89)  ICC: 0.75 (95% CI: 0.48, 0.89)  Placement: RIGHT  Block 1: 1.51 ± 0.13 m/s  Block 2: 1.50 ± 0.09 m/s  CV: 4.95% (95% CI: 3.77, 7.23)  ICC: 0.58 (95% CI: 0.20, 0.81)  65% 1RM (Medium)  Placement: LEFT  Block 1: 1.31 ± 0.11 m/s  Block 2: 1.29 ± 0.11 m/s  CV: 3.91% (95% CI: 2.97, 5.71)  ICC: 0.80 (95% CI: 0.56, 0.92)  Placement: RIGHT  Block 1: 1.25 ± 0.11 m/s  Block 2: 1.24 ± 0.15 m/s  CV: 4.87% (95% CI: 3.71, 7.12)  ICC: 0.81 (95% CI: 0.58, 0.92)  85% 1RM (Heavy)  Placement: LEFT  Block 1: 1.04 ± 0.14 m/s  Block 2: 1.06 ± 0.12 m/s  CV: 8.50% (95% CI: 6.42, 12.57)  ICC: 0.54 (95% CI: 0.12, 0.79)  Placement: RIGHT  Block 1: 1.04 ± 0.13 m/s  Block 2: 1.03 ± 0.15 m/s  CV: 6.61% (95% CI: 5.03, 9.66)  ICC: 0.79 (95% CI: 0.53, 0.91) | Was both biological and technological reliability reported? YES / **NO**  Where the statistics/combination of used to validate the device appropriate? **YES** / NO  Did the original study claim the device was reliable? **YES** / NO  Does this device reliably measure what was measured? YES / **NO** |
|  | T-Force | Intra-device | F/W Back Squat | 45%, 65%, 85% 1RM | Mean Velocity, Maximum Velocity, Left and Right Placement conditions of LPT | 20 | Mean Velocity  45% 1RM (Light)  Placement: LEFT  Block 1: 0.91 ± 0.06 m/s  Block 2: 0.90 ± 0.06 m/s  CV: 3.46% (95% CI: 2.63, 5.05)  ICC: 0.77 (95% CI: 0.50, 0.90)  Placement: RIGHT  Block 1: 0.91 ± 0.08 m/s  Block 2: 0.90 ± 0.07 m/s  CV: 3.37% (95% CI: 2.56, 4.92)  ICC: 0.84 (95% CI: 0.63, 0.93)  65% 1RM (Medium)  Placement: LEFT  Block 1: 0.71 ± 0.06 m/s  Block 2: 0.67 ± 0.07 m/s  CV: 4.53% (95% CI: 3.44, 6.61)  ICC: 0.82 (95% CI: 0.59, 0.92)  Placement: RIGHT  Block 1: 0.71 ± 0.07 m/s  Block 2: 0.69 ± 0.07 m/s  CV: 4.79% (95% CI: 3.65, 7.00)  ICC: 0.78 (95% CI: 0.52, 0.91)  85% 1RM (Heavy)  Placement: LEFT  Block 1: 0.51 ± 0.08 m/s  Block 2: 0.49 ± 0.10 m/s  CV: 9.66% (95% CI: 7.35, 14.11)  ICC: 0.73 (95% CI: 0.43, 0.88)  Placement: RIGHT  Block 1: 0.50 ± 0.09 m/s  Block 2: 0.50 ± 0.09 m/s  CV: 9.45% (95% CI: 7.14, 13.97)  ICC: 0.74 (95% CI: 0.44, 0.89)  MAX Velocity  45% 1RM (Light)  Placement: LEFT  Block 1: 1.52 ± 0.12 m/s  Block 2: 1.51 ± 0.10 m/s  CV: 3.26% (95% CI: 2.48, 4.77)  ICC: 0.81 (95% CI: 0.57, 0.92)  Placement: RIGHT  Block 1: 1.52 ± 0.11 m/s  Block 2: 1.52 ± 0.11 m/s  CV: 3.08% (95% CI: 2.34, 4.50)  ICC: 0.83 (95% CI: 0.63, 0.93)  65% 1RM (Medium)  Placement: LEFT  Block 1: 1.26 ± 0.11 m/s  Block 2: 1.23 ± 0.14 m/s  CV: 4.11% (95% CI: 3.12, 6.00)  ICC: 0.84 (95% CI: 0.64, 0.93)  Placement: RIGHT  Block 1: 1.29 ± 0.11 m/s  Block 2: 1.28 ± 0.10 m/s  CV: 3.81% (95% CI: 2.90, 5.56)  ICC: 0.79 (95% CI: 0.55, 0.91)  85% 1RM (Heavy)  Placement: LEFT  Block 1: 1.03 ± 0.13 m/s  Block 2: 1.02 ± 0.15 m/s  CV: 7.94% (95% CI: 6.04, 11.59)  ICC: 0.70 (95% CI: 0.38, 0.87)  Placement: RIGHT  Block 1: 1.04 ± 0.11 m/s  Block 2: 1.05 ± 0.12 m/s  CV: 5.84% (95% CI: 4.42, 8.64)  ICC: 0.73 (95% CI: 0.43, 0.89) | Was both biological and technological reliability reported? YES / **NO**  Where the statistics/combination of used to validate the device appropriate? **YES** / NO  Did the original study claim the device was reliable? **YES** / NO  Does this device reliably measure what was measured? YES / **NO** |
| Lopez-Torres, Fernandez-Elias [76] | ADR Encoder | Intra-device | S/M Bench Press | 45%, 55%, 65%, 75%, 85% 1RM | Mean Propulsive Velocity | 17 | 45% 1RM  STE: 0.24 m/s (95% CI: 0.20–0.32)  ICC: 0.95 (95% CI: 0.90–0.98)  CV: 9.93% (95% CI: 7.93–11.93%)  55% 1RM  STE: 0.22 m/s (95% CI: 0.18–0.30)  ICC: 0.96 (95% CI: 0.91–0.98)  CV: 11.25% (95% CI: 9.25–13.25%)  65% 1RM  STE: 0.52 m/s (95% CI: 0.43–0.71)  ICC: 0.75 (95% CI: 0.55–0.88)  CV: 6.78% (95% CI: 4.78–8.78%)  75% 1RM  STE: 0.32 m/s (95% CI: 0.26–0.42)  ICC: 0.91 (95% CI: 0.83–0.93)  CV: 10.95% (95% CI: 8.95–12.95%)  85% 1RM  STE: 0.41 m/s (95% CI: 0.34–0.55)  ICC: 0.85 (95% CI: 0.72–0.93)  CV: 14.40% (95% CI: 12.40–16.40%) | Was both biological and technological reliability reported? YES / **NO**  Where the statistics/combination of used to validate the device appropriate? **YES** / NO  Did the original study claim the device was reliable? **YES** / NO  Does this device reliably measure what was measured? YES / **NO** |
|  | T-Force |  |  |  |  |  | 45% 1RM  STE: 0.29 m/s (95% CI: 0.24–0.39)  ICC: 0.93 (95% CI: 0.84–0.976)  CV: 11.72% (95% CI: 10.50–12.94)  55% 1RM  STE: 0.25 m/s (95% CI: 0.21–0.33)  ICC: 0.95 (95% CI: 0.89–0.98)  CV: 13.15% (95% CI: 11.15–15.15)  65% 1RM  STE: 0.34 m/s (95% CI: 0.28–0.46)  ICC: 0.90 (95% CI: 0.80–0.95)  CV: 9.06% (95% CI: 7.06–11.06)  75% 1RM  STE: 0.23 m/s (95% CI: 0.19–0.31)  ICC: 0.95 (95% CI: 0.91–0.98)  CV: 11.93% (95% CI: 9.93–13.93)  85% 1RM  STE: 0.30 m/s (95% CI: 0.25–0.41)  ICC: 0.92 (95% CI: 0.84–0.96)  CV: 16.20% (95% CI: 14.20–18.20) | Was both biological and technological reliability reported? YES / **NO**  Where the statistics/combination of used to validate the device appropriate? **YES** / NO  Did the original study claim the device was reliable? **YES** / NO  Does this device reliably measure what was measured? YES / **NO** |
|  | Speed4Lifts |  |  |  |  |  | 45% 1RM  STE: 0.38 m/s (95% CI: 0.31–0.50)  ICC: 0.87 (95% CI: 0.76–0.94)  CV: 11.92% (95% CI: 9.92–13.92)  55% 1RM  STE: 0.19 m/s (95% CI: 0.16–0.26)  ICC: 0.97 (95% CI: 0.93–0.99)  CV: 14.48% (95% CI: 12.48–16.48)  65% 1RM  STE: 0.39 m/s (95% CI: 0.32–0.53)  ICC: 0.86 (95% CI: 0.74–0.94)  CV: 7.52% (95% CI: 5.52–9.52)  75% 1RM  STE: 0.43 m/s (95% CI: 0.36–0.58)  ICC: 0.83 (95% CI: 0.68–0.92)  CV: 12.30% (95% CI: 10.30–14.30)  85% 1RM  STE: 0.32 m/s (95% CI: 0.26–0.42)  ICC: 0.91 (95% CI: 0.83–0.96)  CV: 14.29% (95% CI: 12.29–16.29) | Was both biological and technological reliability reported? YES / **NO**  Where the statistics/combination of used to validate the device appropriate? **YES** / NO  Did the original study claim the device was reliable? **YES** / NO  Does this device reliably measure what was measured? YES / **NO** |
| Lorenzetti, Lamparter [10] | GymAware | Intra-device | F/W Back Squat, F/W Ballistic Squat | F/W Back Squat 70% 1RM, F/W Ballistic Squat 25 kg | Mean Velocity, Maximum Velocity,  Time to Maximum Velocity | 9 | F/W Back Squat 70% 1RM  Mean Velocity r: 0.958;  RMSE: 0.064 m/s  Maximum velocity r: 0.957;  RMSE: 0.163 m/s  Time to peak velocity r: 0.990;  RMSE: 0.042s  F/W Ballistic Squat 25 kg  Mean Velocity r: 0.783;  RMSE: 0.160 m/s  Maximum velocity r: 0.852;  RMSE: 0.304 m/s  Time to peak velocity r: 0.701;  RMSE: 0.046s | Was both biological and technological reliability reported? YES / **NO**  Where the statistics/combination of used to validate the device appropriate? YES / **NO**  Did the original study claim the device was reliable? **YES** / NO  Does this device reliably measure what was measured? YES / **NO** |
|  | T-Force | Intra-device | F/W Back Squat, F/W Ballistic Squat | F/W Back Squat, 70% 1RM F/W Ballistic Squat 25 kg | Mean Velocity,  Maximum Velocity,  Time to Maximum Velocity | 9 | F/W Back Squat 70% 1RM  Mean Velocity r: 0.970;  RMSE: 0.070 m/s  Maximum velocity r: 0.933;  RMSE: 0.151 m/s m/s  Time to peak velocity: 0.985;  RMSE: 0.026s  F/W Ballistic Squat 25 kg  Mean Velocity r: 0.724;  RMSE: 0.167 m/s  Maximum velocity: 0.810;  RMSE: 0.263 m/s  Time to peak velocity: 0.655;  RMSE: 0.045s | Was both biological and technological reliability reported? YES / **NO**  Where the statistics/combination of used to validate the device appropriate? YES / **NO**  Did the original study claim the device was reliable? **YES** / NO  Does this device reliably measure what was measured? YES / **NO** |
|  | Tendo | Intra-device | F/W Back Squat, F/W Ballistic Squat | F/W Back Squat 70% 1RM, F/W Ballistic Squat 25 kg | Mean Velocity, Maximum Velocity,  Time to Maximum Velocity | 9 | F/W Back Squat 70% 1RM  Mean Velocity r: 0.963;  RMSE: 0.046 m/s  Maximum velocity r: 0.932;  RMSE: 0.194 m/s  Time to peak velocity r: 0.985;  RMSE: 0.041s  F/W Ballistic Squat 25 kg  Mean Velocity r: 0.770;  RMSE: 0.157 m/s  Maximum velocity r: 0.860; RMSE: 0.135 m/s  Time to peak velocity r: 0.604;  RMSE: 0.064s | Was both biological and technological reliability reported? YES / **NO**  Where the statistics/combination of used to validate the device appropriate? YES / **NO**  Did the original study claim the device was reliable? **YES** / NO  Does this device reliably measure what was measured? YES / **NO** |
| Martínez-Cava, Hernández-Belmonte [70] | T-Force | Inter-device | S/M Back Squat, S/M Bench Press | 25-95 kg | Peak Velocity, Mean Propulsive Velocity, Mean Velocity | 15 | S/M Back Squat 25-95 kg  Peak Velocity SEM: 0.01 m/s;  CV: 0.46%;  ICC: 1.000;  r: 0.9997  Mean Propulsive Velocity SEM: 0.01 m/s;  CV: 0.58%;  ICC: 1.000;  r: 0.9998  Mean Velocity SEM: 0.01 m/s;  CV: 0.44%;  ICC: 1.000;  r: 0.9998  S/M Bench Press 25-95 kg  Peak Velocity SEM: 0.01 m/s;  CV: 0.45%;  ICC: 1.000;  r: 0.9998   Mean Propulsive Velocity SEM: 0.01 m/s;  CV: 0.62%;  ICC: 1.000;  r: 0.9999   Mean Velocity SEM: 0.01 m/s;  CV: 0.55%;  ICC: 1.000;  r: 0.9999 | Was both biological and technological reliability reported? YES / **NO**  Where the statistics/combination of used to validate the device appropriate? **YES** / NO  Did the original study claim the device was reliable? **YES** / NO  Does this device reliably measure what was measured? **YES** / NO |
|  | Speed4Lifts | Inter-device | S/M Back Squat, S/M Bench Press | 25-95 kg | Peak Velocity, Mean Propulsive Velocity, Mean Velocity | 15 | S/M Back Squat 25-95 kg  Peak Velocity SEM: 0.01 m/s;  CV: 0.86%;  ICC: 0.999  Mean Propulsive Velocity SEM: 0.01 m/s;  CV: 1.24%;  ICC: 0.999  S/M Bench Press 25-95 kg  Peak Velocity SEM: 0.02 m/s;  CV: 1.54%;  ICC: 1.000   Mean Propulsive Velocity SEM: 0.02 m/s;  CV: 1.80%;  ICC: 0.999 | Was both biological and technological reliability reported? YES / **NO**  Where the statistics/combination of used to validate the device appropriate? **YES** / NO  Did the original study claim the device was reliable? **YES** / NO  Does this device reliably measure what was measured? **YES** / NO |
| Martinopoulou, Tsoukos [65] | Tendo | Intra-device | Bilateral and Unilateral Ballistic Leg Extensions on a Leg Press machine | 35-106 kg  (0.34-1.53m/s) | Mean Velocity  Peak Velocity | 10 | Mean Velocity ICC: 0.990 SEM: 0.02 m/s SEM: 2.5%  Peak Velocity ICC: 0.988 SEM: 0.035 m/s SEM: 3.6% | Was both biological and technological reliability reported? YES / **NO**  Where the statistics/combination of used to validate the device appropriate? YES / **NO**  Did the original study claim the device was reliable? **YES** / NO  Does this device reliably measure what was measured? YES / NO |
| Moreno-Villanueva, Rico-González [87] | ADR Encoder | Inter-device | S/M Bench Press | 5 to 100% 1RM | Mean Propulsive Velocity | 11 | Pause Protocol  Zone 1  ICC: 0.940  r: 0.899  Confidence Interval (95% CI): 0.901 to 0.964  CV: 3.43%  SEM: 0.0153 m/s  SDC: 0.042 m/s  Zone 2  ICC: 0.968  r: 0.946  Confidence Interval (95% CI): 0.938 to 0.983  CV: 4.05%  SEM: 0.0111 m/s  SDC: 0.031 m/s  Zone 3  ICC: 0.998  r: 0.997  Confidence Interval (95% CI): 0.997 to 0.999  CV: 1.65%  SEM: 0.0028 m/s  SDC: 0.008 m/s  Zone 4  ICC: 0.999  r: 0.997  Confidence Interval (95% CI): 0.998 to 0.999  CV: 1.56%  SEM: 0.0022 m/s  SDC: 0.006 m/s  Non-Pause Protocol  Zone 1  ICC: 0.963  r: 0.946  Confidence Interval (95% CI): 0.942 to 0.977  CV: 3.01%  SEM: 0.0099 m/s  SDC: 0.027 m/s  Zone 2  ICC: 0.997  r: 0.994  Confidence Interval (95% CI): 0.994 to 0.998  CV: 1.52%  SEM: 0.0029 m/s  SDC: 0.008 m/s  Zone 3  ICC: 0.999  r: 0.998  Confidence Interval (95% CI): 0.998 to 0.999  CV: 0.96%  SEM: 0.0015 m/s  SDC: 0.004 m/s  Zone 4  ICC: 0.999  r: 0.997  Confidence Interval (95% CI): 0.998 to 0.999  CV: 0.70%  SEM: 0.0012 m/s  SDC: 0.003 m/s | Was both biological and technological reliability reported? YES / **NO**  Where the statistics/combination of used to validate the device appropriate? **YES** / NO  Did the original study claim the device was reliable? **YES** / NO  Does this device reliably measure what was measured? **YES** / NO |
| Muniz-Pardos, Lozano-Berges [71] | T-Force | Intra-device | S/M Half Squat, S/M Bench Press | 40%, 60%, 80% 1RM | Mean Propulsive Velocity | 22 | S/M Bench Press - MPV  40% 1RM  Fixed Bias (95% CI): 0.101 m/s (-0.01 to 0.21 m/s)  Proportional Bias (95% CI): 0.858 (0.72 to 1.00)  60% 1RM  Fixed Bias (95% CI): 0.082 m/s (0.02 to 0.14 m/s)  Proportional Bias (95% CI): 0.859 (0.76 to 0.95)  80% 1RM  Fixed Bias (95% CI): 0.081 m/s (0.04 to 0.12 m/s)  Proportional Bias (95% CI): 0.812 (0.72 to 0.91)  S/M Half Squat - MPV  40% 1RM  Fixed Bias (95% CI): -0.011 m/s (-0.12 to 0.10 m/s)  Proportional Bias (95% CI): 1.033 (0.88 to 1.18)  60% 1RM  Fixed Bias (95% CI): 0.004 m/s (-0.09 to 0.10 m/s)  Proportional Bias (95% CI): 1.008 (0.86 to 1.15)  80% 1RM  Fixed Bias (95% CI): -0.031 m/s (-0.09 to 0.03 m/s)  Proportional Bias (95% CI): 1.082 (0.96 to 1.20) | Was both biological and technological reliability reported? YES / **NO**  Where the statistics/combination of used to validate the device appropriate? YES / **NO**  Did the original study claim the device was reliable? **YES** / NO  Does this device reliably measure what was measured? YES / **NO** |
| Muyor, Granero-Gil [15] | Tendo | Intra-device | S/M Back Squat | 60%, 80% 1RM | Mean Velocity, Eccentric Mean Velocity | 23 | 40% 1RM  Mean Velocity (con)  ES: 0.08 SEM: 0.007 m/s CV: 2.00% ICC: 0.979 Mean Velocity (ecc)  ES: 0.15 SEM: 0.009 m/s CV: 3.65% ICC: 0.970  80% 1RM Mean Velocity (con)  ES: 0.24 SEM: 0.013 m/s CV: 4.28% ICC: 0.855 Mean Velocity (ecc)  ES: 0.10 SEM: 0.011 m/s CV: 4.55% ICC: 0.924 | Was both biological and technological reliability reported? YES / **NO**  Where the statistics/combination of used to validate the device appropriate? **YES** / NO  Did the original study claim the device was reliable? **YES** / NO  Does this device reliably measure what was measured? **YES** / NO |
| Oleksy, Kuchciak [58] | GymAware | Intra-device  (within-day and between-day) | Barbell Landmine Punch, Barbell Landmine Punch Throw | 20 kg, 25 kg, 30 kg, 35 kg | Height (m) Mean Velocity, Peak Velocity | 25 | Within-Day Lowest Reliability: Barbell height (ICC < 0.5), weak to moderate correlations. Good to Excellent: Mean velocity. Higher Loads (30, 35 kg): Moderate ICC, lower/non-significant correlations. Lower Loads (20, 25 kg): Higher reliability, stronger correlations. Without Barbell Throw (NT) vs. With Throw (T): Better reliability for NT. CV & SEM: Low, indicating good data consistency.  Between-Day Test-Retest vs. Intra-Rater: Similar reliability levels. Load Effect: Decreasing reliability with increasing barbell load. Barbell Throw Impact: Higher reliability without throw compared to with throw. CV & SEM: Low, indicating good data consistency. | Was both biological and technological reliability reported? YES / **NO**  Where the statistics/combination of used to validate the device appropriate? **YES** / NO  Did the original study claim the device was reliable? **YES** / NO  Does this device reliably measure what was measured? YES / **NO** |
| Orange, Metcalfe [5] | GymAware | Intra-device | F/W Back Squat, F/W Bench Press | 20%, 40%, 60%, 80%, 90% 1RM | Mean Velocity,  Peak Velocity | 29 | F/W Back Squat  20% 1RM  Mean Velocity Standardized  mean bias: 0.21 SEM: 0.05 m/s ICC: 0.72 Peak Velocity Standardized mean bias: 0.08 SEM: 0.09 m/s ICC: 0.77  40% 1RM Mean Velocity Standardized mean bias: 0.22 SEM: 0.04 m/s ICC: 0.77 Peak Velocity Standardized mean bias: 0.08  SEM: 0.07 m/s ICC: 0.78  60% 1RM Mean Velocity Standardized mean bias: 0.06 SEM: 0.04 m/s ICC: 0.83 Peak Velocity Standardized mean bias: 0.13 SEM: 0.06 m/s ICC: 0.79  80% 1RM Mean Velocity Standardized mean bias: 0.22  SEM: 0.03 m/s ICC: 0.83 Peak Velocity Standardized mean bias: 0.33 SEM: 0.06 m/s ICC: 0.68  90% 1RM  Mean Velocity Standardized mean bias: 0.11 SEM: 0.04 m/s ICC:0.79 Peak Velocity Standardized mean bias: 0.42 SEM: 0.06 m/s ICC: 0.65  F/W Bench Press  20% 1RM  Mean Velocity Standardized mean bias: 0.56  SEM: 0.09 m/s ICC: 0.64 Peak Velocity Standardized mean bias: 0.27 SEM: 0.13 m/s ICC: 0.70  40% 1RM  Mean Velocity Standardized mean bias: 0.27 SEM: 0.05 m/s ICC: 0.71 Peak Velocity Standardized mean bias: 0.21 SEM: 0.06 m/s; ICC: 0.82  60% 1RM  Mean Velocity Standardized mean bias: 0.09 SEM: 0.04 m/s ICC: 0.70 Peak Velocity Standardized mean bias: 0.12 SEM: 0.05 m/s ICC: 0.81  80% 1RM  Mean Velocity Standardized mean bias: 0.00  SEM: 0.04 m/s; ICC: 0.78 Peak Velocity Standardized mean bias: 0.03 SEM: 0.06 m/s; ICC: 0.77  90% 1RM Mean Velocity Standardized mean bias: 0.00 SEM: 0.03 m/s ICC: 0.87 Peak Velocity Standardized mean bias: 0.03 SEM: 0.07 m/s ICC: 0.68 | Was both biological and technological reliability reported? YES / **NO**  Where the statistics/combination of used to validate the device appropriate? **YES** / NO  Did the original study claim the device was reliable? **YES** / NO  Does this device reliably measure what was measured? YES / **NO** |
| Pérez-Castilla, Piepoli [66] | T-Force | Intra-device | S/M Bench Press | 45-85% 1RM | Mean Velocity | 14 | 45% 1RM  Mean Velocity  CV: 2.48%; ICC: 0.90  55% 1RM  Mean Velocity CV: 1.82%; ICC: 0.95  65% 1RM  Mean Velocity  CV: 4.35%; ICC: 0.78  75% 1RM  Mean Velocity  CV: 4.78%; ICC: 0.77  85% 1RM  Mean Velocity  CV: 4.90%; ICC: 0.87 | Was both biological and technological reliability reported? YES / **NO**  Where the statistics/combination of used to validate the device appropriate? YES / **NO**  Did the original study claim the device was reliable? **YES** / NO  Does this device reliably measure what was measured? YES / **NO** |
|  | Chronojump | Intra-device | S/M Bench Press | 45-85% 1RM | Mean Velocity | 14 | 45% 1RM  Mean Velocity  CV: 2.31%; ICC: 0.87  55% 1RM  Mean Velocity  CV: 2.09%; ICC: 0.90  65% 1RM  Mean Velocity  CV: 6.24%; ICC: 0.72  75% 1RM  Mean Velocity  CV: 4.53%; ICC: 0.85  85% 1RM  Mean Velocity  CV: 5.65%; ICC: 0.86 | Was both biological and technological reliability reported? YES / **NO**  Where the statistics/combination of used to validate the device appropriate? YES / **NO**  Did the original study claim the device was reliable? **YES** / NO  Does this device reliably measure what was measured? YES / **NO** |
|  | Speed4Lifts | Intra-device | S/M Bench Press | 45-85% 1RM | Mean Velocity | 14 | 45% 1RM  Mean Velocity  CV: 2.61%; ICC: 0.87  55% 1RM  Mean Velocity  CV: 2.39%; ICC: 0.84  65% 1RM  Mean Velocity  CV: 2.42%; ICC: 0.93  75% 1RM  Mean Velocity  CV: 3.92%; ICC: 0.81  85% 1RM  Mean Velocity  CV: 3.41%; ICC: 0.94 | Was both biological and technological reliability reported? YES / **NO**  Where the statistics/combination of used to validate the device appropriate? YES / **NO**  Did the original study claim the device was reliable? **YES** / NO  Does this device reliably measure what was measured? YES / **NO** |
| Pérez-Castilla, Boullosa [105] | T-Force | Intra-device (Between-session) | S/M Back Squat, S/M Bench Press | 25%, 40%, 55%, 70% 1RM | Mean Velocity | 20 | S/M Back Squat  25% 1RM  Reps 1-5  ES (95%CI): 0.00 (-0.62 to 0.62)  SEM: 0.07 m/s (95% CI: 0.05-0.10)  CV: 7.02% (95%CI: 5.34-10.3)  Reps 1-10  ES (95%CI): 0.00 (-0.62 to 0.62)  SEM: 0.08 m/s (95% CI: 0.06-0.12)  CV: 8.18% (95%CI: 6.22-11.9)  40% 1RM  Reps 1-5  ES (95%CI): 0.15 (-0.47 to 0.77)  SEM: 0.04 m/s (95% CI: 0.03-0.06)  CV: 4.94% (95%CI: 3.76-7.21)  Reps 1-10  ES (95%CI): 0.15 (-0.47 to 0.77)  SEM: 0.05 m/s (95% CI: 0.04-0.08)  CV: 6.23% (95%CI: 4.74-9.11)  55% 1RM  Reps 1-5  ES (95%CI): -0.16 (-0.78 to 0.46)  SEM: 0.04 m/s (95% CI: 0.03-0.06)  CV: 5.64% (95%CI: 4.29-8.24)  Reps 1-10  ES (95%CI): -0.16 (-0.78 to 0.46)  SEM: 0.05 m/s (95% CI: 0.04-0.07)  CV: 6.69% (95%CI: 5.09-9.77)  70% 1RM  Reps 1-5  ES (95%CI): -0.28 (-0.90 to 0.35)  SEM: 0.07 m/s (95% CI: 0.05-0.10)  CV: 11.3% (95%CI: 8.59-16.5)  Reps 1-10  ES (95%CI): -0.40 (-1.03 to 0.22)  SEM: 0.07 m/s (95% CI: 0.05-0.10)  CV: 12.8% (95%CI: 9.70-18.6)  S/M Bench Press  25% 1RM  Reps 1-5  ES (95%CI): -0.24 (-0.87 to 0.38)  SEM: 0.09 m/s (95% CI: 0.07-0.13)  CV: 7.02% (95%CI: 5.34-10.3)  Reps 1-10  ES (95%CI): -0.16 (-0.78 to 0.46)  SEM: 0.09 m/s (95% CI: 0.07-0.13)  CV: 6.98% (95%CI: 6.31-10.2)  40% 1RM  Reps 1-5  ES (95%CI): -0.12 (-0.74 to 0.51)  SEM: 0.05 m/s (95% CI: 0.04-0.09)  CV: 4.55% (95%CI: 3.46-6.65)  Reps 1-10  ES (95%CI): -0.11 (-0.73 to 0.51)  SEM: 0.05 m/s (95% CI: 0.04-0.07)  CV: 4.67% (95%CI: 3.55-6.82)  55% 1RM  Reps 1-5  ES (95%CI): -0.26 (-0.88 to 0.36)  SEM: 0.04 m/s (95% CI: 0.03-0.05)  CV: 4.51% (95%CI: 3.43-6.58)  Reps 1-10  ES (95%CI): -0.12 (-0.74 to 0.50)  SEM: 0.04 m/s (95% CI: 0.03-0.05)  CV: 4.76% (95%CI: 3.62-6.95)  70% 1RM  Reps 1-5  ES (95%CI): 0.00 (-0.62 to 0.62)  SEM: 0.03 m/s (95% CI: 0.03-0.05)  CV: 6.37% (95%CI: 4.84-9.30)  Reps 1-10  ES (95%CI): -0.14 (-0.76 to 0.48)  SEM: 0.04 m/s (95% CI: 0.03-0.06)  CV: 8.26% (95%CI: 6.28-12.1) | Was both biological and technological reliability reported? YES / **NO**  Where the statistics/combination of used to validate the device appropriate? YES / **NO**  Did the original study claim the device was reliable? **YES** / NO  Does this device reliably measure what was measured? YES / **NO** |
| Pérez-Castilla, Miras-Moreno [73] | ADR Encoder | Intra-device (intra-day) | S/M Bench Press | 40%, 60%, 80% 1RM | Mean Velocity | 28 | 40% 1RM  SEM: 0.03 m/s (95% CI: 0.02, 0.04)  CV: 2.80% (95% CI: 2.22, 3.81)  ICC: 0.82 (95% CI: 0.64, 0.91)  60% 1RM  SEM: 0.03 m/s (95% CI: 0.02, 0.04)  CV: 3.91% (95% CI: 3.09, 5.32)  ICC: 0.78 (95% CI: 0.58, 0.89)  80% 1RM  SEM: 0.03 m/s (95% CI: 0.02, 0.04)  CV: 6.40% (95% CI: 5.04, 8.77)  ICC: 0.78 (95% CI: 0.57, 0.89) | Was both biological and technological reliability reported? YES / **NO**  Where the statistics/combination of used to validate the device appropriate? **YES** / NO  Did the original study claim the device was reliable? **YES** / NO  Does this device reliably measure what was measured? YES / **NO** |
|  | T-Force | Intra-device (intra-day) | S/M Bench Press | 40%, 60%, 80% 1RM | Mean Velocity | 28 | 40% 1RM  SEM: 0.03 m/s (95%CI: 0.03, 0.05)  CV: 3.27% (95%CI: 2.58, 4.45)  ICC: 0.81 (95%CI: 0.64, 0.91)  60% 1RM  SEM: 0.03 m/s (95%CI: 0.02, 0.04)  CV: 4.24% (95%CI: 3.36, 5.78)  ICC: 0.78 (95%CI: 0.58, 0.89)  80% 1RM  SEM: 0.03 m/s (95%CI: 0.02, 0.04)  CV: 6.62% (95%CI: 5.21, 9.07)  ICC: 0.77 (95%CI: 0.56, 0.89) | Was both biological and technological reliability reported? YES / **NO**  Where the statistics/combination of used to validate the device appropriate? **YES** / NO  Did the original study claim the device was reliable? **YES** / NO  Does this device reliably measure what was measured? YES / **NO** |
| Rodriguez-Perea, Jerez-Mayorga [85] | Functional Electromechanical Dynamometer  (FEMD) | Intra-device (between-session) | N/A  Study was done over a 40cm range of movement | 0.4 m/s, 0.6 m/s, 0.8 m/s, 1.0 m/s, 1.2 m/s | Mean Velocity, Time to Reach, Isokinetic Velocity (TRIV m/s), Time spent at Isokinetic Velocity (TSIV m/s) | 15 trials | Mean Velocity  0.4 m/s  SEM: 0.000 m/s (95% CI: 0.00, 0.00)  CV: 0.08% (95% CI: 0.06, 0.13)  0.6 m/s  SEM: 0.000 m/s (95% CI: 0.00, 0.00)  CV: 0.11% (95% CI: 0.08, 0.17)  0.8 m/s  SEM: 0.000 m/s (95% CI: 0.00, 0.00)  CV: 0.24% (95% CI: 0.17, 0.37)  1.0 m/s  SEM: 0.000 m/s (95% CI: 0.00, 0.00)  CV: 0.21% (95% CI: 0.15, 0.33)  1.2 m/s  SEM: 0.000 m/s (95% CI: 0.00, 0.00)  CV: 0.22% (95% CI: 0.16, 0.35)  TRIV  0.4 m/s  SEM: 3.14 (95% CI: 1.78, 11.69)  CV: 1.89% (95% CI: 1.07, 7.04)  0.6 m/s  SEM: 5.69 (95% CI: 3.22, 21.20)  CV: 2.83% (95% CI: 1.60, 10.53)  0.8 m/s  SEM: 3.61 (95% CI: 2.04,13.44)  CV: 1.68% (95% CI: 0.95, 6.25)  1.0 m/s  SEM: 4.67 (95% CI: 2.64, 17.41)  CV: 2.01% (95% CI: 1.14, 7.48)  1.2 m/s  SEM: 23.86 (95% CI: 13.52, 88.98)  CV: 9.70% (95% CI: 5.49, 36.15)  TSIV  0.4 m/s  SEM: 5.18 (95% CI: 2.93, 19.31)  CV: 0.53% (95% CI: 0.30, 1.98)  0.6 m/s  SEM: 13.57 (95% CI: 7.69, 50.59)  CV: 2.26% (95% CI: 1.28, 8.41)  0.8 m/s  SEM: 6.04 (95% CI: 3.42, 22.51)  CV: 1.44% (95% CI: 0.82, 5.37)  1.0 m/s  SEM: 24.84 (95% CI: 14.05, 92.62)  CV: 8.33% (95% CI: 4.72, 31.04)  1.2 m/s  SEM: 21.32 (95% CI: 12.07, 79.47)  CV: 8.94% (95% CI: 5.06, 33.32) | Was both biological and technological reliability reported? YES / **NO**  Where the statistics/combination of used to validate the device appropriate? YES / **NO**  Did the original study claim the device was reliable? **YES** / NO  Does this device reliably measure what was measured? YES / **NO** |
| Stock, Beck [64] | Tendo | Intra-device | F/W Bench Press | 10%, 20%, 30%, 40%, 50%, 60%, 70%, 80%, 90% 1RM | Mean Velocity | 21 | F/W Bench Press  Mean Velocity  10% 1RM MD: 0.35 m/s;  SEM: 4.2%;  ICC: 0.717  20% 1RM MD: 0.33 m/s;  SEM: 5.0%;  ICC: 0.572  30% 1RM MD: 0.17 m/s;  SEM: 3.1%;  ICC: 0.805  40% 1RM MD: 0.21 m/s;  SEM: 4.7%;  ICC: 0.669  50% 1RM MD: 0.17 m/s;  SEM: 4.6%;  ICC: 0.790  60% 1RM MD: 0.15 m/s;  SEM: 4.8%;  ICC: 0.785  70% 1RM MD: 0.14 m/s;  SEM: 5.8%;  ICC: 0.811  80% 1RM MD: 0.19 m/s;  SEM: 10.3%;  ICC: 0.714  90% 1RM MD: 0.18 m/s;  SEM: 12.6%;  ICC: 0.564 | Was both biological and technological reliability reported? YES / **NO**  Where the statistics/combination of used to validate the device appropriate? **YES** / NO  Did the original study claim the device was reliable? **YES** / NO  Does this device reliably measure what was measured? YES / **NO** |
| Suchomel, Techmanski [59] | GymAware | Test-retest | Barbell Jump Shrug, Barbell Hang High Pull | 20%, 40%, 60%, 80%, 100% 1RM | Mean Velocity, Peak Velocity | 15 | Jump Shrug  20% Load  MV: ICC = 0.91 (0.78–0.97), CV = 7.1% (5.2–11.5)  PV: ICC = 0.97 (0.93–0.99), CV = 3.4% (2.5–5.4)  40% Load  MV: ICC = 0.96 (0.91–0.99), CV = 4.1% (3.0–6.5)  PV: ICC = 0.97 (0.93–0.99), CV = 3.0% (2.2–4.7)  60% Load  MV: ICC = 0.96 (0.89–0.98), CV = 3.6% (2.6–5.7)  PV: ICC = 0.97 (0.92–0.99), CV = 2.1% (1.5–3.3)  80% Load  MV: ICC = 0.95 (0.87–0.98), CV = 4.9% (3.6–7.8)  PV: ICC = 0.96 (0.90–0.99), CV = 3.4% (2.5–5.5)  100% Load  MV: ICC = 0.97 (0.93–0.99), CV = 3.8% (2.8–6.0)  PV: ICC = 0.96 (0.89–0.98), CV = 3.3% (2.4–5.3)  Hang High Pull  20% Load  MV: ICC = 0.95 (0.89–0.98), CV = 5.8% (4.2–9.2)  PV: ICC = 0.96 (0.92–0.99), CV = 4.6% (3.4–7.4)  40% Load  MV: ICC = 0.97 (0.93–0.99), CV = 3.1% (2.3–5.0)  PV: ICC = 0.95 (0.87–0.98), CV = 3.1% (2.3–5.0)  60% Load  MV: ICC = 0.96 (0.90–0.98), CV = 4.6% (3.3–7.3)  PV: ICC = 0.95 (0.89–0.98), CV = 3.0% (2.2–4.8)  80% Load  MV: ICC = 0.98 (0.94–0.99), CV = 3.2% (2.3–5.1)  PV: ICC = 0.98 (0.96–0.99), CV = 2.5% (1.8–3.9)  100% Load  MV: ICC = 0.98 (0.95–0.99), CV = 3.2% (2.3–5.1)  PV: ICC = 0.98 (0.95–0.99), CV = 2.2% (1.6–3.5) | Was both biological and technological reliability reported? YES / **NO**  Where the statistics/combination of used to validate the device appropriate? **YES** / NO  Did the original study claim the device was reliable? **YES** / NO  Does this device reliably measure what was measured? **YES** / NO **(except for MV at 20% 1RM for both exercises)** |
|  | Tendo | Test-retest | Barbell Jump Shrug,  Barbell Hang High Pull | 20%, 40%, 60%, 80%,  100% 1RM | Mean Velocity,  Peak Velocity | 15 | Jump Shrug  20% Load  MV: ICC = 0.94, CV = 5.9%  PV: ICC = 0.98, CV = 2.5%  40% Load  MV: ICC = 0.95, CV = 5.0%  PV: ICC = 0.97, CV = 2.5%  60% Load  MV: ICC = 0.96, CV = 3.4%  PV: ICC = 0.93, CV = 2.9%  80% Load  MV: ICC = 0.97, CV = 3.8%  PV: ICC = 0.96, CV = 3.6%  100% Load  MV: ICC = 0.98, CV = 2.7%  PV: ICC = 0.95, CV = 3.6%  Hang High Pull  20% Load  MV: ICC = 0.97, CV = 5.5%  PV: ICC = 0.92, CV = 6.0%  40% Load  MV: ICC = 0.97, CV = 3.1%  PV: ICC = 0.93, CV = 2.9%  60% Load  MV: ICC = 0.96, CV = 3.9%  PV: ICC = 0.94, CV = 3.3%  80% Load  MV: ICC = 0.96, CV = 4.0%  PV: ICC = 0.97, CV = 2.9%  100% Load  MV: ICC = 0.97, CV = 4.2%  PV: ICC = 0.96, CV = 3.1% | Was both biological and technological reliability reported? YES / **NO**  Where the statistics/combination of used to validate the device appropriate? **YES** / NO  Did the original study claim the device was reliable? **YES** / NO  Does this device reliably measure what was measured? YES / **NO** **(except for Hang High Pull at 80% 1RM)** |
| Thompson, Rogerson [55] | GymAware | Intra-device | F/W Back Squat, Power Clean | 40%, 50%, 60%, 70%, 80%, 90%, 100% 1RM | Mean Velocity, Peak Velocity | 11 | F/W Back Squat  40% 1RM Mean Velocity  TE: 0.04 m/s;  CV: 4.5% Peak Velocity  TE: 0.08 m/s;  CV: 5.6%  50% 1RM  Mean Velocity  TE: 0.03 m/s;  CV: 3.4% Peak Velocity  TE: 0.07 m/s;  CV: 4.9%  60% 1RM  Mean Velocity  TE: 0.02 m/s;  CV: 2.9% Peak Velocity  TE: 0.08 m/s;  CV: 6.0%  70% 1RM  Mean Velocity  TE: 0.03 m/s;  CV: 4.5% Peak Velocity  TE: 0.10 m/s;  CV: 8.3%  80% 1RM  Mean Velocity  TE: 0.04 m/s;  CV: 7.0% Peak Velocity  TE: 0.09 m/s;  CV: 8.6%  90% 1RM  Mean Velocity  TE: 0.04 m/s;  CV: 9.5% Peak Velocity  TE: 0.09 m/s;  CV: 12.6%  100% 1RM  Mean Velocity  TE: 0.03m/s; CV: 13.6% Peak Velocity  TE: 0.15 m/s;  CV: 22.0%  Full  Mean Velocity  TE: 0.04 m/s;  CV: 9.8% Peak Velocity  TE: 0.10 m/s;  CV: 11.3%  Power clean   40% 1RM  Mean Velocity  TE: 0.05 m/s;  CV: 3.6% Peak Velocity  TE: 0.09 m/s;  CV: 3.7%  50% 1RM  Mean Velocity  TE: 0.03 m/s;  CV: 2.2% Peak Velocity  TE: 0.08 m/s;  CV: 3.7%  60% 1RM  Mean Velocity  TE: 0.03 m/s;  CV: 2.4% Peak Velocity  TE: 0.07 m/s;  CV: 3.1%  70% 1RM  Mean Velocity  TE: 0.04 m/s;  CV: 3.2% Peak Velocity  TE: 0.05 m/s;  CV: 2.5%  80% 1RM  Mean Velocity  TE: 0.04 m/s;  CV: 3.3% Peak Velocity  TE: 0.08 m/s;  CV: 3.8%  90% 1RM  Mean Velocity  TE: 0.08 m/s;  CV: 8.9% Peak Velocity  TE: 0.07 m/s;  CV: 3.9%  100% 1RM  Mean Velocity  TE: 0.04 m/s;  CV: 4.3% Peak Velocity  TE: 0.06 m/s;  CV: 4.0%  Full Mean Velocity  TE: 0.05 m/s;  CV: 4.9% Peak Velocity  TE: 0.07 m/s;  CV: 3.3% | Was both biological and technological reliability reported? YES / **NO**  Where the statistics/combination of used to validate the device appropriate? YES / **NO**  Did the original study claim the device was reliable? **YES** / NO  Does this device reliably measure what was measured? YES / **NO** |
| Qu, Qian [86] | Jueying (Beijing, China) | Test-retest | S/M Back Squat | 30%, 45%, 60%, 75% 1RM | Mean Velocity, Peak Velocity | 12 | Peak Velocity (m/s)  30% 1RM: Mean difference = 0.06 m/s, ICC = 0.89  45% 1RM: Mean difference = 0.01 m/s, ICC = 0.95  60% 1RM: Mean difference = 0.01 m/s, ICC = 0.91  75% 1RM: Mean difference = 0.01 m/s, ICC = 0.79  Mean Velocity (m/s)  30% 1RM: Mean difference = 0.02 m/s, ICC = 0.83  45% 1RM: Mean difference = 0.02 m/s, ICC = 0.89  60% 1RM: Mean difference = 0.01 m/s, ICC = 0.82  75% 1RM: Mean difference = 0.01 m/s, ICC = 0.88 | Was both biological and technological reliability reported? YES / **NO**  Where the statistics/combination of used to validate the device appropriate? **YES** / NO  Did the original study claim the device was reliable? **YES** / NO  Does this device reliably measure what was measured? YES / **NO** |
| Van Den Tillaar and Ball [82] | Musclelab (Ergotest) | Intra-device | F/W Bench Press, Push-Up | F/W Bench Press: 10-30 kg (50% 1RM)  Push Up:  Body Weight, 10-30 kg Weight Vests | Mean Velocity, Mean Peak Velocity | 20 | F/W Bench Press   50% 1RM + 10 kg up to 30 kg Mean Velocity  ICC: 0.98;  CV: 6.6 ± 2.4%;  r: 0.96 Peak Velocity  ICC: 0.98;  CV: 6.9 ± 2.0%;  r: 0.96  F/W push-up Body weight, 10-20-30 kg Weight Vest mean velocity  ICC: 0.98;  CV: 5.9 ± 1.7%;  r: 0.95 peak velocity  ICC: 0.98;  CV: 7.3 ± 3.0%;  r: 0.95 | Was both biological and technological reliability reported? YES / **NO**  Where the statistics/combination of used to validate the device appropriate? YES / **NO**  Did the original study claim the device was reliable? **YES** / NO  Does this device reliably measure what was measured? YES / **NO** |

*Abbreviations: CC: Concordance Correlation, CI: Confidence Interval, CL: Confidence Limit, CV: Coefficient of Variation, ES: Effect Size, F/W: Free Weight, ICC: Intraclass Correlation Coefficient, kg: Kilograms, LoA: Limits of Agreement, MCV: Mean Contraction Velocity, MDC: Minimal Detectable Change, MD: Mean Difference, m/s: Meters per Second, %SEM: Percentage of Standard Error of Measurement, Reps: Repetitions, r: Pearson correlation coefficient, RMSE: Root Mean Square Error, SDC: Smallest Detectable Change, SDC% 1RM: Smallest Detectable Change as a Percentage of 1 Repetition Maximum, SEE: Standard Error of Estimate, SEM: Standard Error of Measurement, SESOI: Smallest Effect Size of Interest, S/M: Smith Machine, STE: Standardized Typical Error, TE: Typical Error.*

S13 Table: Studies investigating the reliability of an IMU/Accelerometer device.

| **Study** | **Device/s** | **Type of Reliability** | **Exercise/s** | **Intensity/Load** | **Variable/s Measured** | **Sample Size** | **Reported Statistics** | **Reliability Criteria** |
| --- | --- | --- | --- | --- | --- | --- | --- | --- |
| Abbott et al. (1) | Bar Sensei,  Assess2Perform | Intra-device | F/W Back Squat | 20%, 30%, 40%, 50%, 60%, 70%, 80%, 90%, 100% 1RM | Peak Velocity, Mean Velocity, Mean Propulsive Velocity | 16 | 20% 1RM  Peak Velocity CV: 14.17% Mean Velocity CV: 18.97% Mean propulsive velocity CV: 15.42%  30% 1RM  Peak Velocity CV: 12.44% Mean Velocity CV: 15.79% Mean propulsive velocity CV: 15.73%  40% 1RM  Peak Velocity CV: 13.39% Mean Velocity CV: 17.58% Mean propulsive velocity CV: 16.13%  50% 1RM  Peak Velocity CV: 15.38% Mean Velocity CV: 20.89% Mean propulsive velocity CV: 17.98%  60% 1RM  Peak Velocity CV: 17.86% Mean Velocity CV: 19.24% Mean propulsive velocity CV: 20.2%  70% 1RM  Peak Velocity CV: 23.97% Mean Velocity CV: 18.82% Mean propulsive velocity CV: 19.21%  80% 1RM  Peak Velocity CV: 31.43% Mean Velocity CV: 25.51% Mean propulsive velocity CV: 25.71%  90% 1RM  Peak Velocity CV: 33.36% Mean Velocity CV: 29.94% Mean propulsive velocity CV: 25.58%  100% 1RM  Peak Velocity CV: 43.77% Mean Velocity CV: 43.02% Mean propulsive velocity CV: 34.59% | Was both biological and technological reliability reported? YES / **NO**  Where the statistics/combination of used to validate the device appropriate? YES / **NO**  Did the original study claim the device was reliable? YES / **NO**  Does this device reliably measure what was measured? YES / **NO** |
| Balsalobre-Fernández, Kuzdub [89] | Push Band | Intra-device | S/M Back Squat | 20-70 kg | Peak Velocity,  Mean Velocity | 10 | Peak Velocity  CV: 6.0 ± 3.9% ICC: 0.981  r: 0.952  Mean Velocity CV: 5.0 ± 4.1% ICC: 0.978 r: 0.956 | Was both biological and technological reliability reported? YES / **NO**  Where the statistics/combination of used to validate the device appropriate? YES / **NO**  Did the original study claim the device was reliable? **YES** / NO  Does this device reliably measure what was measured? YES / **NO** |
| Balsalobre-Fernández, Marchante [78] | Beast Sensor (Wrist) & Beast Sensor (Barbell) | Intra-device | F/W Back Squat, F/W Bench Press, F/W Hip Thrust | 50-95% 1RM | Mean Velocity | 10 | Beast Sensor (wrist):  F/W Back Squat  50-95% 1RM  Mean Velocity  ICC: 0.975  F/W Bench Press  50-95% 1RM  Mean Velocity  ICC: 0.977  F/W Hip Thrust  50-95% 1RM  Mean Velocity  ICC: 0.952  Beast Sensor (Barbell):  F/W Back Squat  50-95% 1RM  Mean Velocity  ICC: 0.979  F/W Bench Press  50-95% 1RM  Mean Velocity  ICC: 0.981  F/W Hip Thrust  50-95% 1RM  Mean Velocity  ICC: 0.958 | Was both biological and technological reliability reported? YES / **NO**  Where the statistics/combination of used to validate the device appropriate? YES / **NO**  Did the original study claim the device was reliable? **YES** / NO  Does this device reliably measure what was measured? YES / **NO** |
| Beckham, Layne [56] | Bar Sensei | Intra-device | F/W Back Squat | 45%, 60%, 75% 1RM | Mean Velocity, Peak Velocity | 16 | 45% 1RM  Mean Velocity  ICC: 0.419 Peak Velocity  ICC: 0.451  60% 1RM  Mean Velocity  ICC: 0.171 Peak Velocity  ICC: 0.273  75% 1RM  Mean Velocity  ICC: 0.295 Peak Velocity  ICC: 0.349 | Was both biological and technological reliability reported? YES / **NO**  Where the statistics/combination of used to validate the device appropriate? YES / **NO**  Did the original study claim the device was reliable? YES / **NO**  Does this device reliably measure what was measured? YES / **NO** |
| Callaghan, Guy [83] | Push Band 2.0 | Test-retest | F/W Back Squat, F/W Front Squat, F/W Bench Press | 20%, 40%, 60%, 80% 1RM | Mean Velocity | 20 | Back Squat  20% 1RM  ICC: 0.80 (High), 95% CI: 0.52 to 0.92  SEM: 0.02 m/s, MDC: 0.07 m/s  CV: 2.63%, Good, 95% CI: 1.12 to 4.13%  40% 1RM  ICC: 0.79 (High), 95% CI: 0.48 to 0.92  SEM: 0.03 m/s, MDC: 0.07 m/s  CV: 3.40%, Good, 95% CI: 1.98 to 4.82%  60% 1RM  ICC: 0.90 (Very High), 95% CI: 0.70 to 0.96  SEM: 0.01 m/s, MDC: 0.03 m/s  CV: 2.85%, Good, 95% CI: 1.75 to 3.95%  80% 1RM  ICC: 0.92 (Very High), 95% CI: 0.80 to 0.97  SEM: 0.01 m/s, MDC: 0.03 m/s  CV: 3.70%, Good, 95% CI: 2.25 to 5.15%  Front Squat  20% 1RM  ICC: 0.64 (Moderate), 95% CI: 0.12 to 0.86  SEM: 0.04 m/s, MDC: 0.11 m/s  CV: 3.62%, Good, 95% CI: 2.45 to 4.78%  40% 1RM  ICC: 0.83 (High), 95% CI: 0.56 to 0.93  SEM: 0.02 m/s, MDC: 0.06 m/s  CV: 2.68%, Good, 95% CI: 1.43 to 3.92%  60% 1RM  ICC: 0.81 (High), 95% CI: 0.53 to 0.93  SEM: 0.02 m/s, MDC: 0.05 m/s  CV: 2.72%, Good, 95% CI: 1.36 to 4.08%  80% 1RM  ICC: 0.87 (High), 95% CI: 0.66 to 0.95  SEM: 0.01 m/s, MDC: 0.04 m/s  CV: 3.13%, Good, 95% CI: 1.75 to 4.51%  Bench Press  20% 1RM  ICC: 0.49 (Low), 95% CI: -0.20 to 0.79  SEM: 0.08 m/s, MDC: 0.22 m/s  CV: 4.67%, Good, 95% CI: 2.70 to 6.64%  40% 1RM  ICC: 0.79 (High), 95% CI: 0.47 to 0.92  SEM: 0.03 m/s, MDC: 0.09 m/s  CV: 4.14%, Good, 95% CI: 2.91 to 5.37%  60% 1RM  ICC: 0.81 (High), 95% CI: 0.52 to 0.92  SEM: 0.03 m/s, MDC: 0.07 m/s  CV: 4.22%, Good, 95% CI: 2.54 to 5.90%  80% 1RM  ICC: 0.82 (High), 95% CI: 0.54 to 0.93  SEM: 0.03 m/s, MDC: 0.08 m/s  CV: 6.89%, Moderate, 95% CI: 3.52 to 10.27% | Was both biological and technological reliability reported? YES / **NO**  Where the statistics/combination of used to validate the device appropriate? **YES** / NO  Did the original study claim the device was reliable? YES / NO (Except for 20%RM for FS and BP)  Does this device reliably measure what was measured? YES / **NO** |
| Courel-Ibáñez, Martínez-Cava [68] | Push Band | Intra-device | S/M Bench Press, S/M Back Squat | 20 kg, 30 kg, 40 kg, 50 kg, 60 kg, 70 kg, 80 kg | Mean Velocity,  Peak Velocity | 17 | S/M Bench Press Mean Velocity  SEM: 0.08 m/s;  CV: 12.2%;  ICC: 0.974   Peak velocity SEM: 0.18 m/s;  CV: 13.7%;  ICC: 0.96  S/M Back Squat Mean Velocity  SEM: 0.06 m/s;  CV: 5.6%;  ICC: 0.979   Peak velocity SEM: 0.09 m/s;  CV: 5.9%;  ICC: 0.944 | Was both biological and technological reliability reported? YES / **NO**  Where the statistics/combination of used to validate the device appropriate? **YES** / NO  Did the original study claim the device was reliable? YES / **NO**  Does this device reliably measure what was measured? YES / **NO** |
| Dragutinovic, Jacobs [93] | VmaxPro | Intra-day, Inter-day | F/W Bench Press, F/W Back Squat | 30%, 50%, 70%, 90% 1RM | Mean Velocity | 17 | Intra-day Reliability: F/W Bench Press CVs ranged from 2.4% to 9.7%.  F/W Back Squat  CVs ranged from 3.7% to 8.6%.  Inter-day Reliability: F/W Bench Press  CVs ranged from 3.5% to 5.9%. F/W Back Squat CVs ranged from 3.2% to 6.7%​​. | Was both biological and technological reliability reported? YES / **NO**  Where the statistics/combination of used to validate the device appropriate? YES / **NO**  Did the original study claim the device was reliable? **YES** / NO  Does this device reliably measure what was measured? YES / **NO** |
| Ferro, Floría [95] | WIMU | Inter-device | S/M Loaded CMJ | 20 kg, 25 kg, 30 kg, 35 kg, 40 kg, 45 kg | Maximum Velocity | 9 | ICC: 0.815 (95% CI: 0.748 to 0.882) Typical Error (TE): 4.88% TE (absolute value): 0.09 m/s Smallest Worthwhile Change (SWC): 0.13 m/s | Was both biological and technological reliability reported? YES / **NO**  Where the statistics/combination of used to validate the device appropriate? **YES** / NO  Did the original study claim the device was reliable? **YES** / NO  Does this device reliably measure what was measured? YES / **NO** |
| Feuerbacher, Jacobs [74] | VmaxPro | Intra-day, Inter-day | F/W Back Squat | 30%, 50%, 70%, 90% 1RM | Mean Velocity | 19 | Intra-day Reliability  30%RM  ICC(p): 0.729–0.901 (<0.001)  50%RM  ICC(p): 0.708–0.911 (<0.001)  70%RM  ICC(p): 0.662–0.859 (<0.001)  90%RM  ICC(p): 0.704–0.938 (<0.001)  Inter-day Reliability  30%RM  ICC 0.623 (0.017)  95% CI: 0.075 to 0.869  50%RM  ICC 0.623 (0.016)  95% CI: 0.077 to 0.869  70%RM  ICC 0.568 (0.033)  95% CI: -0.058 to 0.850  90%RM  ICC 0.837 (,0.001)  95% CI: 0.586 to 0.946 | Was both biological and technological reliability reported? YES / **NO**  Where the statistics/combination of used to validate the device appropriate? **YES** / NO  Did the original study claim the device was reliable? **YES** / NO  Does this device reliably measure what was measured? YES / **NO** |
| García-Pinillos, Latorre-Román [69] | WIMU | Intra-device | S/M Concentric-Only Half ROM Back Squat | 10-100% 1RM | Mean Velocity, Mean Propulsive Velocity | 19 | 10% 1RM  Mean Velocity CV: 9.02%; SEM: 0.03 m/s Mean propulsive velocity CV: 11.69%; SEM: 0.04 m/s Maximum velocity CV: 11.76%; SEM: 0.07 m/s  20% 1RM  Mean Velocity  CV: 6.19%; SEM: 0.02 m/s Mean propulsive velocity CV: 8.14%; SEM: 0.03 m/s Maximum velocity  CV: 8.45%; SEM: 0.05 m/s  30% 1RM  Mean Velocity  CV: 11.77%; SEM: 0.11 m/s Mean propulsive velocity CV: 12.44%; SEM: 0.03 m/s Maximum velocity  CV: 14.44%; SEM: 0.06 m/s  40% 1RM  Mean Velocity  CV: 7.90%; SEM: 0.06 m/s Mean propulsive velocity CV: 8.32%; SEM: 0.02 m/s Maximum velocity  CV: 7.48%; SEM: 0.03 m/s  50% 1RM  Mean Velocity  CV: 7.86%; SEM: 0.06 m/s Mean propulsive velocity CV: 8.99%; SEM: 0.02 m/s Maximum velocity  CV: 7.52%; SEM: 0.03 m/s  60% 1RM  Mean Velocity  CV: 10.41%; SEM: 0.02 m/s Mean propulsive velocity CV: 11.23%; SEM: 0.02 m/s Maximum velocity CV: 13.62%; SEM: 0.05 m/s  70% 1RM  Mean Velocity  CV: 13.82%; SEM: 0.02 m/s Mean propulsive velocity CV: 16.75%; SEM: 0.03 m/s Maximum velocity  CV: 16.80%; SEM: 0.05 m/s  80% 1RM  Mean Velocity  CV: 12.04%; SEM: 0.02 m/s Mean propulsive velocity CV: 13.23%; SEM: 0.02 m/s Maximum velocity  CV: 7.18%; SEM: 0.03 m/s  90% 1RM  Mean Velocity  CV: 12.62%; SEM: 0.06 m/s Mean propulsive velocity CV: 16.47%; SEM: 0.02 m/s Maximum velocity  CV: 11.77%; SEM: 0.03 m/s  100% 1RM  Mean Velocity  CV: 13.27%; SEM: 0.01 m/s Mean propulsive velocity CV: 14.21%; SEM: 0.01 m/s Maximum velocity  CV: 15.66%; SEM: 0.03 m/s 30%, 60%, 90% 1RM | Was both biological and technological reliability reported? YES / **NO**  Where the statistics/combination of used to validate the device appropriate? YES / **NO**  Did the original study claim the device was reliable? **YES** / NO  Does this device reliably measure what was measured? YES / **NO** |
| Gilic, Gabrilo [90] | Push Band 2.0 | Test-retest | Hex Bar Deadlift | 45%, 55%, 65%, 75%, 85%, 95% 1RM | Mean Velocity, Peak Velocity | 16 | 45% 1RM Mean Velocity R: 0.83  Peak Velocity R: 0.81 55% 1RM Mean Velocity R: 0.39  Peak Velocity R: 0.61  65% 1RM Mean Velocity R: 0.38  Peak Velocity R: 0.24  75% 1RM Mean Velocity R: 0.69  Peak Velocity R: 0.66  85% 1RM Mean Velocity R: 0.51  Peak Velocity R: 0.51  95% 1RM Mean Velocity R: 0.01  Peak Velocity R: 0.3 | Was both biological and technological reliability reported? YES / **NO**  Where the statistics/combination of used to validate the device appropriate? YES / **NO**  Did the original study claim the device was reliable? **YES** / NO  Does this device reliably measure what was measured? YES / **NO** |
| Jovanovic and Jukic [57] | Push Band 2.0 | Inter-device  (Left and Right) | Hex Bar Deadlift | 40%, 60%, 80%, 90%, 100% 1RM | Peak Velocity, Mean Velocity | 12 | MV fixed bias: Intercept = -0.118 m/s, 95% CI (-0.155 to -0.086) PV fixed bias: Intercept = -0.174 m/s, 95% CI (-0.223 to -0.125)  MV SESOI: ±0.032 m/s, 95% CI (0.029–0.035) PV SESOI: ±0.048 m/s, 95% CI (0.043–0.054)  MV slope: 1.116, 95% CI (1.059–1.185) PV slope: 1.075, 95% CI (1.029–1.122)  MV SDC: 0.260 m/s, 95% CI (0.227–0.320) PV SDC: 0.431 m/s, 95% CI (0.376–0.509)  MV SDC% 1RM: 14.558%, 95% CI (12.698–17.964) PV SDC% 1RM: 14.113% 1RM, 95% CI (12.155–16.930) | Was both biological and technological reliability reported? YES / **NO**  Where the statistics/combination of used to validate the device appropriate? **YES** / NO  Did the original study claim the device was reliable? YES / **NO**  Does this device reliably measure what was measured? YES / **NO** |
| Held, Rappelt [84] | VmaxPro | Within-day | F/W Back Squat, F/W Hip Thrust | 75% 1RM | Mean Velocity, Barbell Displacement | 19 | F/W Back Squat  MCV (m/s)  TE: 3%  CV: 4.4%  ICC: 0.88 (95% CI: 0.71-0.95)  CC: 0.87 (95% CI: 0.69-0.95)  LoA: 0.05 m/s  SEM: 0.09 m/s  Barbell Displacement (cm)  TE: 4.5%  CV: 6.5%  ICC: 0.91 (95% CI: 0.79-0.97)  CC: 0.91 (95% CI: 0.78-0.96)  LoA: 7.29 cm  SEM: 1.11 cm  F/W Hip Thrust  MCV (m/s)  TE: 5.3%  CV: 7.4%  ICC: 0.8 (95% CI: 0.56-0.92)  CC: 0.81 (95% CI: 0.57-0.93)  LoA: 0.09 m/s  SEM: 0.02 m/s  Barbell Displacement (cm)  TE: 6%  CV: 8.7%  ICC: 0.58 (95% CI: 0.25-0.75)  CC: 0.57 (95% CI: 0.16-0.82)  LoA: 0.21 cm  SEM: 0.07 cm | Was both biological and technological reliability reported? YES / **NO**  Where the statistics/combination of used to validate the device appropriate? **YES** / NO  Did the original study claim the device was reliable? **YES** / NO  Does this device reliably measure what was measured? YES / **NO** |
|  | VmaxPro | Between Day | F/W Back Squat, F/W Hip Thrust | 75% 1RM | Mean Velocity, Barbell Displacement | 19 | F/W Back Squat  MCV (m/s)  TE: 6.9%  CV: 9.9%  ICC: 0.82 (95% CI: 0.67-0.90)  CC: 0.81 (95% CI: 0.66-0.90)  LoA: 0.16 m/s  SEM: 0.04 m/s  Barbell Displacement (cm)  TE: 5.6%  CV: 8%  ICC: 0.83 (95% CI: 0.69-0.91)  CC: 0.83 (95% CI: 0.69-0.91)  LoA: 9.43 cm  SEM: 1.98 cm  F/W Hip Thrust  MCV (m/s)  TE: 10.4%  CV: 14.9%  ICC: 0.55 (95% CI: 0.25-0.75)  CC: 0.55 (95% CI: 0.24-0.75)  LoA: 0.21 m/s  SEM: 0.07 m/s  Barbell Displacement (cm)  TE: 9.3%  CV: 13.3%  ICC: 0.41 (95% CI: 0.07-0.66)  CC: 0.41 (95% CI: 0.07-0.66)  LoA: 10.75 cm  SEM: 4.23 cm | Was both biological and technological reliability reported? YES / **NO**  Where the statistics/combination of used to validate the device appropriate? **YES** / NO  Did the original study claim the device was reliable? **YES** / NO  Does this device reliably measure what was measured? YES / **NO** |
| Lake, Augustus [91] | PUSH Band 2.0 (bar) | Intra-device | F/W Bench Press | 60%, 90% 1RM | Peak Velocity, Mean Velocity | 14 | 60% 1RM  Peak Velocity  ICC: 0.947 CV: 4.2% Mean Velocity  ICC: 0.937 CV: 5.8%  90% 1RM Peak Velocity ICC: 0.957 CV: 4.7% Mean Velocity  ICC: 0.973 CV: 7.2% | Was both biological and technological reliability reported? YES / **NO**  Where the statistics/combination of used to validate the device appropriate? YES / **NO**  Did the original study claim the device was reliable? **YES** / NO  Does this device reliably measure what was measured? YES / **NO** |
| Lorenzetti, Lamparter [10] | Myotest | Intra-device | F/W Ballistic Squat | 25 kg | Mean Velocity, Maximum Velocity, Time to Peak Velocity | 9 | F/W Ballistic Squat 25 kg  Mean Velocity r: 0.610  Maximum velocity  r: 0.552  Time to peak velocity r: 0.700 | Was both biological and technological reliability reported? YES / **NO**  Where the statistics/combination of used to validate the device appropriate? YES / **NO**  Did the original study claim the device was reliable? YES / **NO**  Does this device reliably measure what was measured? YES / **NO** |
| Mateo [96] | RehaGait sensor | Intra-device  (Bar Attachment  vs Lower Back Attachment) | F/W Back Squat | <1 kg | Mean Velocity | 6 | F/W Back Squat mean concentric velocity t = 0.385,  p = 0.716,  mean difference ± SD = 0.046 ± 0.052 | Was both biological and technological reliability reported? YES / **NO**  Where the statistics/combination of used to validate the device appropriate? YES / **NO**  Did the original study claim the device was reliable? YES / **NO**  Does this device reliably measure what was measured? YES / **NO** |
| Merrigan and Martin [98] | Output Sports Unit | Intra-device | F/W Back Squat, F/W Bench Press | 35%, 45%, 55%, 65%, 75% 85% 1RM | Mean Velocity, Peak Velocity | 11 | Bench Press 35% 1RM MV ICC: 0.9 CV: 0.06%  PV ICC: 0.9 CV: 0.06%  45% 1RM MV ICC: 0.9 CV: 0.03%  PV ICC: 0.9 CV: 0.03%  55% 1RM MV ICC: 0.9 CV: 0.05%  PV ICC: 0.9 CV: 0.05%  65% 1RM MV ICC: 0.8 CV: 0.04%  PV ICC: 0.8 CV: 0.05%  75% 1RM MV ICC: 0.8 CV: 0.05%  PV ICC: 0.9 CV: 0.05%   85% 1RM MV ICC: 0.65 CV: 0.08%  PV ICC: 0.75 CV: 0.07%   Back Squat 35% 1RM MV ICC: 0.75 CV: 0.09%  PV ICC: 0.7 CV: 0.08%  45% 1RM MV ICC: 0.95 CV: 0.05%  PV ICC: 0.99 CV: 0.05%  55% 1RM MV ICC: 0.95 CV: 0.07%  PV ICC: 0.9 CV: 0.05%  65% 1RM MV ICC: 0.9 CV: 0.04%  PV ICC: 0.9 CV: 0.07%  75% 1RM MV ICC: 0.85 CV: 0.06%  PV ICC: 0.9 CV: 0.08%   85% 1RM MV ICC: 0.6 CV: 0.10%  PV ICC: 0.85 CV: 0.12% | Was both biological and technological reliability reported? YES / **NO**  Where the statistics/combination of used to validate the device appropriate? YES / **NO**  Did the original study claim the device was reliable? **YES** / NO  Does this device reliably measure what was measured? YES / **NO** |
| Muyor, Granero-Gil [15] | WIMU | Intra-device | S/M Back Squat | 40%, 80% 1RM | Mean Velocity, Eccentric Mean Velocity | 23 | 40% 1RM  Mean Velocity (con)  ES: 0.00 SEM: 0.007 m/s  CV: 2.60%  ICC: 0.976 Mean Velocity (ecc)  ES: 0.06 SEM: 0.013 m/s  CV: 3.79% ICC: 0.955  80% 1RM  Mean Velocity (con)  ES: 0.00  SEM: 0.011 m/s  CV: 3.53%  ICC: 0.905 Mean Velocity (ecc)  ES: 0.11 SEM: 0.010 m/s  CV: 4.51% ICC: 0.924 | Was both biological and technological reliability reported? YES / **NO**  Where the statistics/combination of used to validate the device appropriate? **YES** / NO  Did the original study claim the device was reliable? **YES** / NO  Does this device reliably measure what was measured? **YES** / NO |
| Olaya-Cuartero, Villalón-Gasch [48] | VmaxPro | Within-day | F/W Back Squat | 75%, 85%, 90%, 95% 1RM | Mean Velocity, Displacement | 20 | Mean Velocity (m/s) Systematic Error: -0.021 m/s Random Error: 0.053 m/s ICC (95% CI): 0.986 (0.944 to 0.994) Cronbach’s α: 0.995  Displacement (cm) Systematic Error: 1.935 cm Random Error: 1.901 cm ICC (95% CI):  0.812 (-0.048 to 0.948) Cronbach’s α : 0.977 | Was both biological and technological reliability reported? YES / **NO**  Where the statistics/combination of used to validate the device appropriate? **YES** / NO  Did the original study claim the device was reliable? **YES** / NO  Does this device reliably measure what was measured? **YES** / NO |
| Orange, Metcalfe [11] | Push Band (arm) | Intra-device | F/W Back Squat,  F/W Bench Press | 20%, 40%, 60%, 80%, 90% 1RM | Mean Velocity, Peak Velocity | 29 | F/W Back Squat   20% 1RM  Mean Velocity  SEM: 0.08 m/s ICC: 0.68 Peak Velocity  SEM: 0.12 m/s ICC: 0.71  40% 1RM  Mean Velocity  SEM: 0.07 m/s ICC:0.62 Peak Velocity  SEM: 0.18 m/s ICC: 0.25  60% 1RM  Mean Velocity  SEM: 0.06 m/s ICC: 0.64 Peak Velocity  SEM: 0.11 m/s ICC: 0.55  80% 1RM  Mean Velocity  SEM: 0.06 m/s;  ICC: 0.60 Peak Velocity  SEM: 0.11 m/s  ICC: 0.44  90% 1RM  Mean Velocity  SEM: 0.06 m/s  ICC: 0.36 Peak Velocity  SEM: 0.12 m/s ICC: 0.66  F/W Bench Press   20% 1RM  Mean Velocity  SEM: 0.11 m/s ICC: 0.28 Peak Velocity  SEM: 0.21 m/s ICC: 0.27  40% 1RM  Mean Velocity  SEM: 0.08 m/s ICC: 0.60 Peak Velocity  SEM: 0.11 m/s ICC: 0.66  60% 1RM  Mean Velocity  SEM: 0.08 m/s ICC: 0.58 Peak Velocity SEM: 0.12 m/s;  ICC:58  80% 1RM  Mean Velocity  SEM: 0.06 m/s ICC: 0.51 Peak Velocity  SEM: 0.08 m/s ICC: 0.47  90% 1RM  Mean Velocity  SEM: 0.05m/s ICC: 0.37 Peak Velocity  SEM: 0.10 m/s ICC: 0.40 | Was both biological and technological reliability reported? YES / **NO**  Where the statistics/combination of used to validate the device appropriate? YES / **NO**  Did the original study claim the device was reliable? YES / **NO**  Does this device reliably measure what was measured? YES / **NO** |
| Pelaez Barrajon and San Juan [99] | Accelerometer Mobile Basic Program (MBP) via Huawei G620S Smartphone | Intra-device | F/W Bench Press | 70%, 90%, 100% 1RM | Mean Velocity | 10 | ICC = 0.634 CI = 0.308–0.794 Cronbach’s αlpha = 0.698 | Was both biological and technological reliability reported? YES / **NO**  Where the statistics/combination of used to validate the device appropriate? YES / **NO**  Did the original study claim the device was reliable? YES / **NO**  Does this device reliably measure what was measured? YES / **NO** |
| Pérez-Castilla, Piepoli [66] | Push band (arm) | Intra-device | S/M Bench press | 45-85% 1RM | Mean Velocity | 14 | 45% 1RM  Mean Velocity  CV: 5.02%; ICC: 0.69  55% 1RM  Mean Velocity  CV: 7.84%; ICC: 0.46  65% 1RM  Mean Velocity  CV: 9.34%; ICC: 0.78  75% 1RM  Mean Velocity  CV: 14.6%; ICC: 0.50  85% 1RM  Mean Velocity  CV: 19.1%; ICC: 0.47 | Was both biological and technological reliability reported? YES / **NO**  Where the statistics/combination of used to validate the device appropriate? YES / **NO**  Did the original study claim the device was reliable? YES / **NO**  Does this device reliably measure what was measured? YES / **NO** |
|  | Beast Sensor | Intra-device | S/M Bench press | 45-85% 1RM | Mean Velocity | 14 | 45% 1RM  Mean Velocity  CV: 33.4%;  ICC: 0.29  55% 1RM  Mean Velocity  CV: 24.2%;  ICC: 0.64  65% 1RM  Mean Velocity  CV: 35.0%;  ICC: 0.30  75% 1RM  Mean Velocity  CV: 40.2%;  ICC: 0.31  85% 1RM  Mean Velocity  CV: 54.9%;  ICC: 0.27 | Was both biological and technological reliability reported? YES / **NO**  Where the statistics/combination of used to validate the device appropriate? YES / **NO**  Did the original study claim the device was reliable? YES / **NO**  Does this device reliably measure what was measured? YES / **NO** |
| Suchomel, Techmanski [59] | Push Band 2.0 | Test-retest | Barbell Jump Shrug, Barbell Hang High Pull | 20% 1RM 40% 1RM 60% 1RM 80% 1RM 100% 1RM | Mean Velocity, Peak Velocity | 15 | Jump Shrug  20% Load  MV: ICC = 0.91 (0.77–0.97), CV = 5.8% (4.2–9.3)  PV: ICC = 0.96 (0.91–0.99), CV = 3.0% (2.2–4.8)  40% Load  MV: ICC = 0.90 (0.77–0.96), CV = 6.1% (4.4–9.8)  PV: ICC = 0.96 (0.91–0.99), CV = 3.4% (2.5–5.4)  60% Load  MV: ICC = 0.89 (0.73–0.96), CV = 5.5% (4.0–8.9)  PV: ICC = 0.89 (0.75–0.96), CV = 4.0% (2.9–6.4)  80% Load  MV: ICC = 0.94 (0.86–0.98), CV = 5.3% (3.8–8.5)  PV: ICC = 0.96 (0.91–0.99), CV = 3.4% (2.4–5.3)  100% Load  MV: ICC = 0.79 (0.49–0.92), CV = 13.2% (9.5–21.5)  PV: ICC = 0.69 (0.26–0.89), CV = 8.6% (6.3–14.0)  Hang High Pull  20% Load  MV: ICC = 0.90 (0.75–0.96), CV = 5.0% (3.6–8.0)  PV: ICC = 0.65 (0.14–0.88), CV = 5.1% (3.7–8.2)  40% Load  MV: ICC = 0.88 (0.73–0.96), CV = 11.2% (8.1–18.3)  PV: ICC = 0.38 (−0.47–0.78), CV = 12.0% (8.6–19.7)  60% Load  MV: ICC = 0.94 (0.85–0.98), CV = 6.3% (4.6–10.1)  PV: ICC = 0.90 (0.76–0.96), CV = 5.5% (4.0–8.8)  80% Load  MV: ICC = 0.94 (0.84–0.98), CV = 6.2% (4.5–9.9)  PV: ICC = 0.88 (0.70–0.96), CV = 6.3% (4.6–10.3)  100% Load  MV: ICC = 0.94 (0.86–0.98), CV = 5.3% (3.9–8.5)  PV: ICC = 0.92 (0.81–0.97), CV = 4.7% (3.4–7.5) | Was both biological and technological reliability reported? YES / **NO**  Where the statistics/combination of used to validate the device appropriate? **YES** / NO  Did the original study claim the device was reliable? YES / **NO**  Does this device reliably measure what was measured? YES / **NO** |
| Thompson, Rogerson [55] | PUSH Band (body) | Intra-device | F/W Back Squat, Power Clean | 40%, 50%, 60%, 70%, 80%, 90%, 100% 1RM | Mean Velocity, Peak Velocity | 11 | F/W Back Squat   40% 1RM  Mean Velocity  TE: 0.04 m/s -1; CV: 3.5% Peak Velocity  TE: 0.08 m/s -1; CV: 6.0%  50% 1RM  Mean Velocity  TE: 0.03 m/s -1; CV: 4.1% Peak Velocity  TE: 0.07 m/s -1; CV: 9.9%  60% 1RM  Mean Velocity  TE: 0.02 m/s -1; CV: 5.4% Peak Velocity  TE: 0.08 m/s -1; CV: 9.1%  70% 1RM  Mean Velocity  TE: 0.03 m/s -1; CV: 5.0% Peak Velocity  TE: 0.10 m/s -1; CV: 8.9%  80% 1RM  Mean Velocity  TE: 0.04 m/s -1; CV: 5.2% Peak Velocity  TE: 0.09 m/s -1; CV: 6.8%  90% 1RM  Mean Velocity  TE: 0.04 m/s -1; CV: 15.6% Peak Velocity  TE: 0.09 m/s -1; CV: 11.0%  100% 1RM  Mean Velocity  TE: 0.03 m/s -1; CV: 14.9% Peak Velocity  TE: 0.15 m/s -1; CV: 11.4%  Full  Mean Velocity  TE: 0.04 m/s -1; CV: 10.6% Peak Velocity  TE: 0.10 m/s -1; CV: 11.3%  Power Clean   40% 1RM  Mean Velocity  TE: 0.06 m/s -1; CV: 4.9% Peak Velocity  TE: 0.08 m//s -1; CV: 4.9%  50% 1RM  Mean Velocity  TE: 0.06 m/s -1; CV: 5.2% Peak Velocity  TE: 0.08 m/s -1; CV: 5.2%  60% 1RM  Mean Velocity  TE: 0.05 m//s -1; CV: 4.5% Peak Velocity  TE: 0.08 m/s -1; CV: 4.5%  70% 1RM  Mean Velocity  TE: 0.09 m/s;  CV: 7.7% Peak Velocity  TE: 0.12 m/s;  CV: 7.7%  80% 1RM  Mean Velocity  TE: 0.10 m/s;  CV: 10.2% Peak Velocity  TE: 0.14 m/s;  CV: 10.2%  90% 1RM  Mean Velocity  TE: 0.09 m/s;  CV: 11.3% Peak Velocity  TE: 0.13 m/s;  CV: 11.3%  100% 1RM  Mean Velocity  TE: 0.09 m/s;  CV: 11.4% Peak Velocity  TE: 0.12 m/s;  CV: 11.4%  Full  Mean Velocity  TE: 0.08 m/s;  CV: 8.3% Peak Velocity  TE: 0.11 m/s;  CV: 8.3% | Was both biological and technological reliability reported? YES / **NO**  Where the statistics/combination of used to validate the device appropriate? YES / **NO**  Did the original study claim the device was reliable? **YES** / NO  Does this device reliably measure what was measured? YES / **NO** |
|  | PUSH Band (bar) | Intra-device | F/W Back Squat, Power Clean | 40%, 50%, 60%, 70%, 80%, 90%,  100% 1RM | Mean Velocity, Peak Velocity | 11 | F/W Back Squat   40% 1RM  Mean Velocity  TE: 0.06 m/s;  CV: 5.2% Peak Velocity  TE: 0.09 m/s;  CV: 5.7%  50% 1RM  Mean Velocity  TE: 0.08 m/s;  CV: 9.2% Peak Velocity  TE: 0.10 m/s;  CV: 7.5%  60% 1RM  Mean Velocity  TE: 0.05 m/s;  CV: 5.1% Peak Velocity  TE: 0.12 m/s;  CV: 9.4%  70% 1RM  Mean Velocity  TE: 0.04 m/s;  CV: 5.9% Peak Velocity  TE: 0.09 m/s;  CV: 8.3%  80% 1RM  Mean Velocity  TE: 0.09 m/s;  CV: 14.3% Peak Velocity  TE: 0.09 m/s;  CV: 8.8%  90% 1RM  Mean Velocity  TE: 0.09 m/s;  CV: 20.3% Peak Velocity  TE: 0.12 m/s;  CV: 14.2%  100% 1RM  Mean Velocity  TE: 0.06 m/s;  CV: 15.4% Peak Velocity  TE: 0.09 m/s;  CV: 11.6%  Full Mean Velocity  TE: 0.07 m/s;  CV: 14.5% Peak Velocity  TE: 0.11 m/s;  CV: 11.0%  Power Clean   40% 1RM  Mean Velocity  TE: 0.20 m/s;  CV: 21.5% Peak Velocity  TE: 0.36 m/s;  CV: 21.5%  50% 1RM  Mean Velocity  TE: 0.18 m/s;  CV: 19.0% Peak Velocity  TE: 0.33 m/s;  CV: 17.9%  60% 1RM  Mean Velocity  TE: 0.17 m/s;  CV: 18.9% Peak Velocity  TE: 0.42 m/s;  CV: 25.4%  70% 1RM  Mean Velocity  TE: 0.13 m/s;  CV: 14.6% Peak Velocity  TE: 0.22 m/s;  CV: 13.4%  80% 1RM  Mean Velocity  TE: 0.14 m/s;  CV: 16.3% Peak Velocity  TE: 0.25 m/s;  CV: 15.6%  90% 1RM  Mean Velocity  TE: 0.15 m/s;  CV: 18.1% Peak Velocity  TE: 0.31 m/s;  CV: 22.2%  100% 1RM  Mean Velocity  TE: 0.10 m/s;  CV: 13.3% Peak Velocity  TE: 0.23 m/s;  CV: 17.5%  Full  Mean Velocity  TE: 0.21 m/s;  CV: 18.6% Peak Velocity TE: 0.32 m/s;  CV: 20.5% | Was both biological and technological reliability reported? YES / **NO**  Where the statistics/combination of used to validate the device appropriate? YES / **NO**  Did the original study claim the device was reliable? **YES** / NO  Does this device reliably measure what was measured? YES / NO |
|  | Bar Sensei | Intra-device | F/W Back Squat, Power Clean | 40%, 50%, 60%, 70%, 80%, 90%,  100% 1RM | Mean Velocity, Peak Velocity | 11 | F/W Back Squat   40% 1RM  Mean Velocity  TE: 0.08 m/s;  CV: 9.1% Peak Velocity  TE: 0.14 m/s;  CV: 9.4%  50% 1RM  Mean Velocity  TE: 0.09 m/s;  CV: 13.5% Peak Velocity  TE: 0.10 m/s;  CV: 7.6%  60% 1RM  Mean Velocity  TE: 0.07 m/s;  CV: 8.8% Peak Velocity  TE: 0.08 m/s;  CV: 8.0%  70% 1RM  Mean Velocity  TE: 0.07 m/s;  CV: 10.7%  TE: 0.07 m/s;  CV:10.7% Peak Velocity  TE: 0.09 m/s;  CV: 10.2%  80% 1RM  Mean Velocity  TE: 0.08 m/s;  CV: 18.3% Peak Velocity  TE: 0.24 m/s;  CV: 35.8%  90% 1RM  Mean Velocity  TE: 0.08 m/s;  CV: 19.1% Peak Velocity  TE: 0.12 m/s;  CV: 18.0%  100% 1RM  Mean Velocity  TE: 0.13 m/s;  CV: 60.5% Peak Velocity  TE: 0.12 m/s;  CV: 28.5%  Full Mean Velocity  TE: 0.09 m/s;  CV: 22.1% Peak Velocity  TE: 0.13 m/s;  CV: 18.7%  Power Clean   40% 1RM  Mean Velocity  TE: 0.23 m/s;  CV: 20.4% Peak Velocity  TE: 0.20 m/s;  CV: 7.7%  50% 1RM  Mean Velocity  TE: 0.16 m/s;  CV: 13.8% Peak Velocity  TE: 0.15 m/s;  CV: 6.5%  60% 1RM  Mean Velocity  TE: 0.13 m/s;  CV: 12.1% Peak Velocity  TE: 0.13 m/s;  CV: 5.8%  70% 1RM  Mean Velocity  TE: 0.13 m/s; CV: 11.8% Peak Velocity  TE: 0.19 m/s;  CV: 8.8%  TE: 0.19 m/s;  CV:8.8%  80% 1RM  Mean Velocity  TE: 0.13 m/s;  CV: 14.9% Peak Velocity  TE: 0.13 m/s; CV: 6.1%  90% 1RM  Mean Velocity  TE: 0.15 m/s;  CV: 17.7% Peak Velocity  TE: 0.14 m/s;  CV: 7.9%  100% 1RM  Mean Velocity  TE: 0.14 m/s;  CV: 18.4% Peak Velocity  TE: 0.15 m/s;  CV: 8.5%  Full  Mean Velocity  TE: 0.16 m/s;  CV: 15.9% Peak Velocity  TE: 0.17 m/s; CV: 8.7% | Was both biological and technological reliability reported? YES / **NO**  Where the statistics/combination of used to validate the device appropriate? YES / **NO**  Did the original study claim the device was reliable? **YES** / NO  Does this device reliably measure what was measured? YES / NO |
|  | Beast Sensor | Intra-device | F/W Back Squat | 40%, 50%, 60%, 70%, 80%, 90%,  100% 1RM | Mean Velocity, Peak Velocity | 11 | F/W Back Squat  40% 1RM  Mean Velocity  TE: 0.05 m/s; CV: 20.4% Peak Velocity  TE: 0.10 m/s; CV: 7.7%  50% 1RM  Mean Velocity  TE: 0.06 m/s; CV: 13.8% Peak Velocity  TE: 0.11 m/s; CV: 6.5%  60% 1RM  Mean Velocity  TE: 0.08 m/s; CV: 12.1% Peak Velocity  TE: 0.15 m/s; CV: 5.8%  70% 1RM  Mean Velocity  TE: 0.12 m/s; CV: 11.8% Peak Velocity  TE: 0.27 m/s; CV: 8.8%  80% 1RM  Mean Velocity  TE: 0.22 m/s; CV: 14.9% Peak Velocity  TE: 0.33 m/s; CV: 6.1%  90% 1RM  Mean Velocity  TE: 0.21 m/s; CV: 17.7% Peak Velocity  TE: 0.48 m/s; CV: 7.9%  100% 1RM  Mean Velocity  TE: 0.15 m/s; CV: 18.4% Peak Velocity  TE: 0.29 m/s; CV: 8.5%  Full  Mean Velocity  TE: 0.14 m/s; CV: 15.9% | Was both biological and technological reliability reported? YES / **NO**  Where the statistics/combination of used to validate the device appropriate? YES / **NO**  Did the original study claim the device was reliable? **YES** / NO  Does this device reliably measure what was measured? YES / NO |
| Van Den Tillaar and Ball [82] | PUSH Band (arm) | Intra-device | F/W Bench Press Push-Up | F/W Bench Press: 10-30 kg  (50% 1RM)  Push Up:  Body Weight, 10-30 kg Weight Vests | Mean Velocity, Mean Peak Velocity | 20 | F/W Bench Press  50% 1RM  Mean Velocity  ICC: 0.95;  CV: 12.8 ± 2.4%;  r: 0.87 Peak Velocity  ICC: 0.92;  CV: 13.3 ± 2.3%;  r: 0.81  Push-up Body Weight  10-20-30 kg Weight Vest Mean Velocity  ICC: 0.98;  CV: 6.6 ± 1.3%;  r: 0.95 Peak Velocity  ICC: 0.98;  CV: 6.6 ± 1.3%;  r: 0.94 | Was both biological and technological reliability reported? YES / **NO**  Where the statistics/combination of used to validate the device appropriate? YES / **NO**  Did the original study claim the device was reliable? YES / **NO**  Does this device reliably measure what was measured? YES / **NO** |

*Abbreviations: 1RM: One Repetition Maximum, CI: Confidence Interval, con: Concentric, CV: Coefficient of Variation, ecc: Eccentric, ES: Effect Size, F/W: Free Weights, ICC: Intraclass Correlation Coefficient, kg: Kilograms, LoA: Limits of Agreement, MDC: Minimal Detectable Change, m/s: Meters per Second, r: Pearson Correlation Coefficient, SDC: Smallest Detectable Change, SEM: Standard Error of Measurement, SESOI: Smallest Effect Size of Interest, S/M: Smith Machine, SWC: Smallest Worthwhile Change, TE: Typical Error.*

S14 Table: Studies investigating the reliability of a 2D Motion Analysis device.

| **Study** | **Device/s** | **Type of Reliability** | **Exercise/s** | **Intensity/Load** | **Variable/s Measured** | **Sample Size** | **Reported Statistics** | **Reliability Criteria** |
| --- | --- | --- | --- | --- | --- | --- | --- | --- |
| Balsalobre-Fernández, Marchante [78] | PowerLift  (v4.0) | Intra-device | F/W Back Squat,  F/W Bench Press, F/W Hip Thrust | 50-95% 1RM | Mean Velocity | 10 | F/W Back Squat  50-95% 1RM  Mean Velocity  ICC: 0.981  F/W Bench Press  50-95% 1RM  Mean Velocity  ICC: 0.974  F/W Hip Thrust  50-95% 1RM  Mean Velocity  ICC: 0.961 | Was both biological and technological reliability reported? YES / **NO**  Where the statistics/combination of used to validate the device appropriate? YES / **NO**  Did the original study claim the device was reliable? **YES** / NO  Does this device reliably measure what was measured? YES / NO |
| Balsalobre-Fernández, Marchante [101] | PowerLift  (v2.8iOS) | Intra-observer | F/W Bench Press | 75–100% 1RM | Mean Velocity | 10) | MD: -0.0007 ± 0.02 m/s ICC = 0.941,  90% CI = 0.922-0.955 | Was both biological and technological reliability reported? YES / **NO**  Where the statistics/combination of used to validate the device appropriate? **YES** / NO  Did the original study claim the device was reliable? **YES** / NO  Does this device reliably measure what was measured? YES / **NO** |
| Balsalobre-Fernández, Xu [103] | My Jump Lab v 3.0  with iPhone 12 Pro running iOS 15.5 (previously My Lift) | Intra-device (within-session) | F/W Bench Press | 50%, 75% 1RM | Mean Velocity | 27 | 50% 1RM  CV: 8.17% (95% CI: 5.99-10.34)  ICC: 0.97 (95% CI: 0.94-0.98)  75% 1RM  CV: 13.55% (95% CI: 9.93-17.16)  ICC: 0.96 (95% CI: 0.92-0.98) | Was both biological and technological reliability reported? YES / **NO**  Where the statistics/combination of used to validate the device appropriate? **YES** / NO  Did the original study claim the device was reliable? **YES** / NO  Does this device reliably measure what was measured? YES / **NO** |
| Cetin and Isik [102] | My Lift (v.9.1.3) iPad Pro 11. (Apple, USA) | Intra-device | F/W Back Squat, F/W Deadlift | 75%, 85% 1RM | Mean Velocity | 10 | ICC: 0.99 for both exercises | Was both biological and technological reliability reported? YES / **NO**  Where the statistics/combination of used to validate the device appropriate? YES / **NO**  Did the original study claim the device was reliable? **YES** / NO  Does this device reliably measure what was measured? YES / **NO** |
| Courel-Ibáñez, Martínez-Cava [68] | PowerLift  (v4.0) | Inter-device | S/M Bench Press,  S/M Back Squat | 20 kg, 30 kg, 40 kg, 50 kg, 60 kg, 70 kg, 80 kg | Mean Velocity | 17 | S/M Bench Press Mean Velocity  SEM: 0.08 m/s; CV: 10.4%; ICC: 0.973   S/M Back Squat Mean Velocity  SEM: 0.08 m/s; CV: 10.4%; ICC: 0.973 | Was both biological and technological reliability reported? YES / **NO**  Where the statistics/combination of used to validate the device appropriate? **YES** / NO  Did the original study claim the device was reliable? YES / **NO**  Does this device reliably measure what was measured? YES / **NO** |
|  | PowerLift  (v4.0) | Intra-device | S/M Bench Press,  S/M Back Squat | 20 kg, 30 kg, 40 kg, 50 kg, 60 kg, 70 kg, 80 kg | Mean Velocity | 17 | S/M Bench Press Mean Velocity  SEM:0.05 m/s; CV: 6.7%; ICC: 0.988  S/M Back Squat Mean Velocity  SEM:0.04 m/s; CV: 4.6%; ICC: 0.986 | Was both biological and technological reliability reported? YES / **NO**  Where the statistics/combination of used to validate the device appropriate? **YES** / NO  Did the original study claim the device was reliable? YES / **NO**  Does this device reliably measure what was measured? YES / **NO** |
| Jiménez-Olmedo, Penichet-Tomás [109] | Kinovea (v.0.9.1) via  Smartphone (Redmi Note 8, Xiaomi, Beijing, China) | Intra-device | S/M Half ROM Back Squat | <40% 1RM, 40 to 70% 1RM, >70% 1RM, 20 kg and 50 kg | Mean Velocity, Maximum Velocity | 15 | Mean Velocity  Consistency >  ICC (95%CI): 0.995 (0.989 ˆ’ 0.998) Agreement >  ICC (95%CI): 0.978 (0.315 ˆ’ 0.995) Cronbach's a: 0.997 Mean Difference (95% CI): 0.03 (0.03 ˆ’ 0.04)  Maximum Velocity  Consistency >  ICC (95%CI): 0.997 (0.994 ˆ’ 0.999) Agreement >  ICC (95%CI): 0.980 (0.173 ˆ’ 0.996) Cronbach's a: 0.998 Mean Difference (95% CI): 0.05 (0.05 ˆ’ 0.06) | Was both biological and technological reliability reported? YES / **NO**  Where the statistics/combination of used to validate the device appropriate? **YES** / NO  Did the original study claim the device was reliable? **YES** / NO  Does this device reliably measure what was measured? **YES** / NO |
| Martínez-Cava, Hernández-Belmonte [70] | PowerLift My Lift (v8.1 iOS) | Inter-device | S/M Back Squat, S/M Bench Press | 25-95 kg | Peak Velocity | 15 | S/M Back Squat  Peak Velocity SEM: 0.08 m/s;  CV: 5.79%;  ICC: 0.993  S/M Bench Press  Peak Velocity SEM: 0.08 m/s;  CV: 5.02%;  ICC: 0.972 | Was both biological and technological reliability reported? YES / **NO**  Where the statistics/combination of used to validate the device appropriate? **YES** / NO  Did the original study claim the device was reliable? YES / **NO**  Does this device reliably measure what was measured? YES / **NO** |
| Martinopoulou, Tsoukos [65] | Tracker 5.0.6 software via  Casio Exilim Pro EX-F1) with sampling frequency 300 fps | Intra-device | Bilateral and Unilateral Ballistic Leg Extensions on a Leg Press machine | 35-106 kg  (0.34-1.53m/s) | Mean Velocity, Peak Velocity | 10 | Mean Velocity ICC: 0.987 SEM: 0.02 %SEM: 2.7%  Peak Velocity ICC: 0.992 SEM: 0.03 %SEM: 2.0% | Was both biological and technological reliability reported? YES / **NO**  Where the statistics/combination of used to validate the device appropriate? YES / **NO**  Did the original study claim the device was reliable? **YES** / NO  Does this device reliably measure what was measured? YES / **NO** |
| Pérez-Castilla, Piepoli [66] | PowerLift  (v6.0.1) | Intra-device | S/M Bench Press | 45-85% 1RM | Mean Velocity | 14 | 45% 1RM  Mean Velocity  CV: 2.85%; ICC: 0.84  55% 1RM  Mean Velocity  CV: 3.97%; ICC: 0.85  65% 1RM  Mean Velocity  CV: 4.91%; ICC: 0.74  75% 1RM  Mean Velocity  CV: 3.69%; ICC: 0.87  85% 1RM  Mean Velocity  CV: 4.97%; ICC: 0.85 | Was both biological and technological reliability reported? YES / **NO**  Where the statistics/combination of used to validate the device appropriate? YES / **NO**  Did the original study claim the device was reliable? **YES** / NO  Does this device reliably measure what was measured? YES / **NO** |
| Pérez-Castilla, Boullosa [105] | iLOADApp via  two iPhone 8 Plus running iOS 12.4.5 (iPhone; Apple, Inc., Cupertino, CA). | Intra-device (Between-session) | S/M Back Squat S/M Bench Press | 25%, 40%, 55%, 70% 1RM | Mean Velocity | 20 | S/M Back Squat  25% 1RM  Reps 1-5  ES (95%CI): -0.25 (-0.88 to 0.37)  SEM: 0.10 m/s (95% CI: 0.08-0.15)  CV: 9.71% (95%CI: 7.38-14.2)  Reps 1-10  ES (95%CI): -0.25 (-0.87 to 0.37)  SEM: 0.10 m/s (95% CI: 0.08-0.15)  CV: 9.79% (95%CI: 7.45-14.3)  40% 1RM  Reps 1-5  ES (95%CI): 0.00 (-0.62 to 0.62)  SEM: 0.06 m/s (95% CI: 0.05-0.09)  CV: 6.72% (95%CI: 5.11-9.82)  Reps 1-10  ES (95%CI): -0.11 (-0.73 to 0.51)  SEM: 0.05 m/s (95% CI: 0.04-0.07)  CV: 5.61% (95%CI: 4.27-8.20)  55% 1RM  Reps 1-5  ES (95%CI): -0.29 (-0.92 to 0.33)  SEM: 0.07 m/s (95% CI: 0.06-0.11)  CV: 9.45% (95%CI: 7.19-13.8)  Reps 1-10  ES (95%CI): -0.22 (-0.85 to 0.39)  SEM: 0.06 m/s (95% CI: 0.05-0.09)  CV: 8.63% (95%CI: 6.56-12.6)  70% 1RM  Reps 1-5  ES (95%CI): -0.37 (-1.00 to 0.25)  SEM: 0.09 m/s (95% CI: 0.07-0.14)  CV: 16.1% (95%CI: 12.2-23.5)  Reps 1-10  ES (95%CI): -0.39 (-1.02 to 0.24)  SEM: 0.09 m/s (95% CI: 0.07-0.13)  CV: 17.4% (95%CI: 13.2-25.3)  S/M Bench Press  25% 1RM  Reps 1-5  ES (95%CI): -0.25 (-0.87 to 0.37)  SEM: 0.11 m/s (95% CI: 0.09-0.17)  CV: 9.05% (95%CI: 6.88-13.2)  Reps 1-10  ES (95%CI): -0.25 (-0.88 to 0.37)  SEM: 0.11 m/s (95% CI: 0.08-0.16)  CV: 8.66% (95%CI: 6.59-12.7)  40% 1RM  Reps 1-5  ES (95%CI): -0.08 (-0.70 to 0.54)  SEM: 0.09 m/s (95% CI: 0.07-0.13)  CV: 8.17% (95%CI: 6.22-11.9)  Reps 1-10  ES (95%CI): -0.09 (-0.71 to 0.53)  SEM: 0.08 m/s (95% CI: 0.06-0.11)  CV: 7.10% (95%CI: 5.40-10.4)  55% 1RM  Reps 1-5  ES (95%CI): -0.09 (-0.71 to 0.53)  SEM: 0.07 m/s (95% CI: 0.05-0.10)  CV: 7.18% (95%CI: 5.46-10.5)  Reps 1-10  ES (95%CI): 0.00 (-0.62 to 0.62)  SEM: 0.08 m/s (95% CI: 0.06-0.11)  CV: 9.20% (95%CI: 7.00-13.4)  70% 1RM  Reps 1-5  ES (95%CI): 0.10 (-0.52 to 0.72)  SEM: 0.08 m/s (95% CI: 0.06-0.12)  CV: 12.1% (95%CI: 9.23-17.7)  Reps 1-10  ES (95%CI): -0.10 (-0.72 to 0.52)  SEM: 0.07 m/s (95% CI: 0.05-0.10)  CV: 11.4% (95%CI: 8.66-16.6) | Was both biological and technological reliability reported? YES / **NO**  Where the statistics/combination of used to validate the device appropriate? YES / **NO**  Did the original study claim the device was reliable? **YES** / NO  Does this device reliably measure what was measured? YES / **NO** |
| Pueo, Lopez [111] | Novel video system via  Pocophone F1, Xiaomi, Pekin, China) | Intra-device | S/M Back Squat | 75%, 85%, 90%, 95% 1RM | Mean Velocity, Range (Displacement) | 20 | All Loads  Range: ICC (95% CI): 0.996 (0.881-0.999)  Cronbach’s a: 0.999  Mean Difference (95% CI): -0.35 (-0.39- -0.31) cm SWC (95% CI): 0.97 (0.87-1.09) cm SEM: 0.31 cm  SWC/SEM Ratio: 3.16  SEE (95% CI): 0.21 (0.19-0.23) cm  Standardized SEE (95% CI): 0.04 (0.04-0.05)  SEE Effect Size: Trivial  Mean Velocity ICC: 0.988 (0.542-0.997) Cronbach’s a: 0.999 Mean Difference (95% CI): -0.016 (0.018- -0.015) m/s SWC (95% CI): 0.02 (0.02-0.03) m/s SEM: 0.010 m/s SWC/SEM Ratio: 2.00 SEE (95% CI): 0.01 (0.01-0.01) m/s Standardized SEE (95% CI): 0.08 (0.07-0.09) SEE Effect Size: Trivial | Was both biological and technological reliability reported? YES / **NO**  Where the statistics/combination of used to validate the device appropriate? **YES** / NO  Did the original study claim the device was reliable? **YES** / NO  Does this device reliably measure what was measured? **YES** / NO |
| Thompson, Rogerson [55] | MyLift  (PowerLift at time of data collection) | Intra-device | F/W Back Squat | 40%, 50%, 60%, 70%, 80%, 90%, 100% 1RM | Mean Velocity | 11 | F/W Back Squat  40% 1RM  Mean Velocity  TE: 0.04 m/s;  CV: 4.2%  50% 1RM  Mean Velocity  TE: 0.03 m/s;  CV: 3.7%  60% 1RM  Mean Velocity  TE: 0.04 m/s;  CV: 5.5%  70% 1RM  Mean Velocity  TE: 0.03 m/s;  CV: 4.9%  80% 1RM  Mean Velocity  TE: 0.04 m/s;  CV: 6.8%  90% 1RM  Mean Velocity  TE: 0.05 m/s;  CV: 12.6%  100% 1RM  Mean Velocity  TE: 0.03 m/s;  CV: 13.8%  Full Mean Velocity  TE: 0.05 m/s;  CV: 9.7% | Was both biological and technological reliability reported? YES / **NO**  Where the statistics/combination of used to validate the device appropriate? YES / **NO**  Did the original study claim the device was reliable? **YES** / NO  Does this device reliably measure what was measured? YES / **NO** |

*Abbreviations: 1RM: One Repetition Maximum, CI: Confidence Interval, CV: Coefficient of Variation, ES: Effect Size, F/W: Free Weights, ICC: Intraclass Correlation Coefficient, kg: Kilograms, m/s: Meters per Second, MD: Mean Difference, SEE: Standard Error of the Estimate, SEM: Standard Error of Measurement, S/M: Smith Machine, SWC: Smallest Worthwhile Change, TE: Typical Error.*

S15 Table: Studies investigating the reliability of a 3D motion capture analysis or advanced camera system device.

| **Study** | **Device/s** | **Type of Reliability** | **Exercise/s** | **Intensity/Load** | **Variable/s Measured** | **Sample Size** | **Reported Statistics** | **Reliability Criteria** |
| --- | --- | --- | --- | --- | --- | --- | --- | --- |
| Tomasevicz, Hasenkamp [112] | Elite Form Training System (EFTS) | Intra-device (between-session) | Power Clean, F/W Deadlift, F/W Bench Press, F/W Back Squat, F/W Front Squat, Jump Squat | 15%, 35%, 55%, 75%, 85% 1RM  and  Jump Squat 10% 1RM (of back Squat weight)  20% 1RM (of back Squat weight)  30% 1RM (of back Squat weight)  40% 1RM (of back Squat weight)  50% 1RM (of back Squat weight) | Peak Velocity, Mean Velocity | 6 | Between EFTS session1 and EFTS session 2 (n=1800)  ALL EXERCISES  peak Velocity  r: 0.961  Average Velocity  r: 0.935  EFTS Capture Errors All Exercises  Missed errors (error %): 72 (2.0%)  Multiple Errors (error %): 74 (2.1%) | Was both biological and technological reliability reported? YES / **NO**  Where the statistics/combination of used to validate the device appropriate? YES / **NO**  Did the original study claim the device was reliable? **YES** / NO  Does this device reliably measure what was measured? YES / **NO** |
| Weakley, Munteanu [47] | Perch | Intra-device  (technological & biological variability) | F/W Back Squat,  F/W Bench Press | 20%, 40%, 60%, 80%, 90%, 100% 1RM | Mean Velocity,  Peak Velocity | 16 | F/W Back Squat  20% 1RM Mean Velocity: SEM: 0.06 m/s MDC: 0.17 m/s Peak Velocity: SEM: 0.06 m/s MDC: 0.15 m/s  40% 1RM Mean Velocity: SEM: 0.04 m/s MDC: 0.12 m/s Peak Velocity: SEM: 0.04 m/s MDC: 0.12 m/s  60% 1RM Mean Velocity: SEM: 0.04 m/s MDC: 0.11 m/s Peak Velocity: SEM: 0.05 m/s MDC: 0.13 m/s  80% 1RM Mean Velocity: SEM: 0.04 m/s MDC: 0.12 m/s Peak Velocity: SEM: 0.06 m/s MDC: 0.16 m/s  90-100% 1RM Mean Velocity: SEM: 0.05 m/s MDC: 0.15 m/s Peak Velocity: SEM: 0.05 m/s MDC: 0.14 m/s  F/W Bench Press  20% 1RM Mean Velocity: SEM: 0.05 m/s MDC: 0.14 m/s Peak Velocity: SEM: 0.08 m/s MDC: 0.21 m/s  40% 1RM Mean Velocity: SEM: 0.05 m/s MDC: 0.13 m/s Peak Velocity: SEM: 0.08 m/s MDC: 0.23 m/s  60% 1RM Mean Velocity: SEM: 0.04 m/s MDC: 0.10 m/s Peak Velocity: SEM: 0.06 m/s MDC: 0.17 m/s  80% 1RM Mean Velocity: SEM: 0.07 m/s MDC: 0.21 m/s Peak Velocity: SEM: 0.07 m/s MDC: 0.19 m/s  90-100% 1RM Mean Velocity: SEM: 0.07 m/s MDC: 0.18 m/s Peak Velocity: SEM: 0.06 m/s MDC: 0.17 m/s | Was both biological and technological reliability reported? **YES** / NO  Where the statistics/combination of used to validate the device appropriate? **YES** / NO  Did the original study claim the device was reliable? **YES** / NO  Does this device reliably measure what was measured? **YES** / NO |

*Abbreviations: 1RM: One Repetition Maximum, CI: Confidence Interval, CV: Coefficient of Variation, ES: Effect Size, F/W: Free Weights, ICC: Intraclass Correlation Coefficient, MDC: Minimal Detectable Change, MD: Mean Difference, m/s: Meters per Second, r: Pearson correlation coefficient, SEE: Standard Error of the Estimate, SEM: Standard Error of Measurement, S/M: Smith Machine, SWC: Smallest Worthwhile Change, TE: Typical Error.*

S16 Table: Studies investigating the reliability of an Optic/Laser device.

| **Study** | **Device/s** | **Type of Reliability** | **Exercise/s** | **Intensity/Load** | **Variable/s Measured** | **Sample Size** | **Reported Statistics** | **Reliability Criteria** |
| --- | --- | --- | --- | --- | --- | --- | --- | --- |
| Courel-Ibáñez, Martínez-Cava [68] | Velowin | Inter-device | S/M Bench Press, S/M Prone Bench Pull | 20 kg, 30 kg, 40 kg, 50 kg, 60 kg,70 kg, 80 kg | Mean Velocity, Mean Propulsive Velocity,  Peak Velocity | 17 | S/M Bench Press Mean Velocity  SEM: 0.03 m/s;  CV: 3.5%;  ICC: 0.997   Mean Propulsive Velocity  SEM: 0.03 m/s;  CV: 3.4%;  ICC: 0.997  Peak velocity SEM: 0.03 m/s;  CV: 2.1%;  ICC: 0.999  S/M Prone Bench Pull Mean Velocity  SEM: 0.04 m/s;  CV: 3.6%;  ICC: 0.995  Mean Propulsive Velocity  SEM: 0.04 m/s;  CV: 3.5%;  ICC: 0.995   Peak velocity SEM: 0.06 m/s;  CV: 3.2%; ICC: 0.998 | Was both biological and technological reliability reported? YES / **NO**  Where the statistics/combination of used to validate the device appropriate? **YES** / NO  Did the original study claim the device was reliable? **YES** / NO  Does this device reliably measure what was measured? **YES** / NO |
|  | Velowin | Intra-device | S/M Bench Press, S/M Back Squat S/M Prone Bench Pull | 20 kg, 30 kg, 40 kg, 50 kg, 60 kg,70 kg, 80 kg | Mean Velocity,  Mean Propulsive Velocity,  Peak Velocity | 17 | S/M Bench Press Mean Velocity  SEM: 0.04 m/s;  CV: 4.0%;  ICC: 0.997   Mean Propulsive Velocity  SEM: 0.03 m/s;  CV: 3.2%;  ICC: 0.998   Peak velocity SEM: 0.04 m/s;  CV: 2.6%;  ICC: 0.998   S/M Back Squat Mean Velocity SEM: 0.04 m/s;  CV: 3.7%;  ICC: 0.988   Mean Propulsive Velocity  SEM: 0.06 m/s;  CV: 4.6%;  ICC: 0.987   PEAK velocity SEM: 0.07 m/s;  CV: 3.5%;  ICC: 0.983   S/M Prone Bench Pull Mean Velocity SEM: 0.06 m/s;  CV: 3.9%;  ICC: 0.994   Mean Propulsive Velocity  SEM: 0.06 m/s;  CV: 3.9%;  ICC: 0.994    Peak velocity SEM: 0.06 m/s;  CV: 2.6%;  ICC: 0.998 | Was both biological and technological reliability reported? YES / **NO**  Where the statistics/combination of used to validate the device appropriate? **YES** / NO  Did the original study claim the device was reliable? **YES** / NO  Does this device reliably measure what was measured? **YES** / NO |
| García-Ramos, Pérez-Castilla [67] | Velowin | Intra-device | F/W Back Squat | 20 kg, 40 kg, 50 kg, 60 kg, 70 kg | Mean Velocity, Mean Propulsive Velocity,  Maximum Velocity | 31 | 20 kg  Mean Velocity SEM: 0.045 m/s;  CV: 4.29%; ICC: 0.91  Mean propulsive SEM: 0.054 m/s;  CV: 4.61%; ICC: 0.90 velocity  Maximum velocity SEM: 0.088 m/s;  CV: 4.77%; ICC: 0.92   40 kg Mean Velocity SEM: 0.041 m/s;  CV: 4.34%; ICC: 0.92   Mean Propulsive Velocity SEM: 0.047 m/s;  CV: 4.60%; ICC: 0.91   Maximum velocity SEM: 0.085 m/s; CV: 5.01%; ICC: 0.91   50 kg Mean Velocity  SEM: 0.033 m/s;  CV: 3.74%; ICC: 0.90   Mean Propulsive Velocity SEM: 0.043 m/s;  CV: 4.50%; ICC: 0.88   Maximum velocity SEM: 0.050 m/s;  CV: 3.04%; ICC: 0.95   60 kg Mean Velocity  SEM: 0.039 m/s;  CV: 4.75%; ICC: 0.89   Mean Propulsive Velocity SEM: 0.037 m/s;  CV: 4.20%; ICC: 0.92   Maximum velocity SEM: 0.069 m/s; CV: 4.44%; ICC: 0.91   70 kg Mean Velocity  SEM: 0.031 m/s;  CV: 4.12%; ICC: 0.93   Mean Propulsive Velocity SEM: 0.041 m/s;  CV: 5.15%; ICC: 0.90   Maximum velocity SEM: 0.053 m/s;  CV: 3.57%; ICC: 0.95 | Was both biological and technological reliability reported? YES / **NO**  Where the statistics/combination of used to validate the device appropriate? **YES** / NO  Did the original study claim the device was reliable? **YES** / NO  Does this device reliably measure what was measured? **YES** / NO |
| Laza-Cagigas, Goss-Sampson [113] | Velowin | Intra-device | F/W Back Squat | <30-90% 1RM | Barbell Displacement, Mean Velocity, Peak Velocity | 11 | Displacement RMSE: 3.73 cm; CV: 6.6%; ICC: 0.84   Mean Velocity RMSE: 0.06 m/s; CV:7.3%; ICC: 0.97   Peak Velocity RMSE: 0.09 m/s; CV: 6.5%; ICC: 0.96 | Was both biological and technological reliability reported? YES / **NO**  Where the statistics/combination of used to validate the device appropriate? **YES** / NO  Did the original study claim the device was reliable? **YES** / NO  Does this device reliably measure what was measured? **YES** / NO |
| Muniz-Pardos, Lozano-Berges [71] | Velowin | Intra-device | S/M Half ROM Back Squat, S/M Bench Press | 40%, 60%, 80% 1RM | Mean Propulsive Velocity | 22 | S/M Bench Press - MPV  40% 1RM  Fixed Bias: 0.063 m/s (95% CI: -0.06 to 0.19)  Proportional Bias: 0.904 (95% CI: 0.75 to 1.06)  60% 1RM  Fixed Bias: 0.015 m/s (95% CI: -0.06 to 0.09)  Proportional Bias: 0.976 (95% CI: 0.86 to 1.09)  80% 1RM  Fixed Bias: 0.058 m/s (95% CI: 0.02 to 0.10)  Proportional Bias: 0.867 (95% CI: 0.77 to 0.96)  S/M Half Squat - MPV  40% 1RM  Fixed Bias: -0.048 m/s (95% CI: -0.17 to 0.08)  Proportional Bias: 1.091 (95% CI: 0.92 to 1.26)  60% 1RM  Fixed Bias: -0.065 m/s (95% CI: -0.17 to 0.04)  Proportional Bias: 1.128 (95% CI: 0.96 to 1.29)  80% 1RM  Fixed Bias: -0.092 m/s (95% CI: -0.17 to -0.02)  Proportional Bias: 1.206 (95% CI: 1.07 to 1.35) | Was both biological and technological reliability reported? YES / **NO**  Where the statistics/combination of used to validate the device appropriate? YES / **NO**  Did the original study claim the device was reliable? **YES** / NO  Does this device reliably measure what was measured? YES / **NO** |
| Peña García-Orea, Belando-Pedreño [114] | Velowin (v.1.7.232) | Intra-device | S/M Back Squat | 20 kg, 30 kg, 40 kg, 50 kg, 60 kg,70 kg | Mean Velocity, Mean Propulsive Velocity, Peak Velocity | 26 | 20 kg  Mean Velocity  ICC: 0.95; CV: 3.35%; SEM: 0.225 m/s Mean propulsive velocity ICC: 0.96; CV: 3.29%; SEM: 0.287 m/s Peak Velocity  ICC: 0.95; CV: 2.89%; SEM: 0.399 m/s  30 kg  Mean Velocity  ICC: 0.97; CV: 2.20%; SEM: 0.227 m/s Mean propulsive velocity ICC: 0.97; CV: 2.45%; SEM: 0.284 m/s Peak Velocity  ICC: 0.97; CV: 2.46%; SEM: 0.373 m/s  40 kg  Mean Velocity  ICC: 0.99; CV: 2.13%; SEM: 0.248 m/s Mean propulsive velocity ICC: 0.99; CV: 2.30%; SEM: 0.306 m/s Peak Velocity  ICC: 0.98; CV: 2.29%; SEM: 0.363 m/s  50 kg  Mean Velocity  ICC: 0.98; CV: 2.82%; SEM: 0.276 m/s Mean propulsive velocity ICC: 0.99; CV: 2.98%; SEM: 0.337 m/s Peak Velocity  ICC: 0.98; CV: 2.56%; SEM: 0.362 m/s  60 kg  Mean Velocity  ICC: 0.99; CV: 2.46%; SEM: 0.272 m/s Mean propulsive velocity ICC: 0.99; CV: 2.62%; SEM: 0.316 m/s Peak Velocity ICC: 0.98; CV: 2.39%; SEM: 0.313 m/s  70 kg  Mean Velocity  ICC: 0.99; CV: 2.55%; SEM: 0.243 m/s Mean propulsive velocity ICC: 0.99; CV: 2.79%; SEM: 0.280 m/s Peak Velocity  ICC: 0.98; CV: 2.30%; SEM: 0.269 m/s | Was both biological and technological reliability reported? YES / **NO**  Where the statistics/combination of used to validate the device appropriate? **YES** / NO  Did the original study claim the device was reliable? **YES** / NO  Does this device reliably measure what was measured? **YES** / NO |
| Peña García-Orea, Belando-Pedreño [72] | Velowin (v1.7.232) | Intra-device | Loaded CMJ | 3.5-43.5 kg | Mean Velocity, Peak Velocity | 21 | 3.5 kg  Mean Velocity  ICC: 0.98; CV: 2.41%; SEM: 0.0025 m/s Peak Velocity  ICC: 0.98; CV: 1.77%; SEM: 0.0021 m/s  13.5 kg  Mean Velocity  ICC: 0.97; CV: 1.70%; SEM: 0.0021 m/s Peak Velocity  ICC: 0.99; CV: 1.68%; SEM: 0.0014 m/s  23.5 kg  Mean Velocity  ICC: 0.95; CV: 2.56%; SEM: 0.0033 m/s Peak Velocity  ICC: 0.97; CV: 2.38%; SEM: 0.0023 m/s  33.5 kg  Mean Velocity  ICC: 0.98; CV: 1.87%; SEM: 0.0022 m/s Peak Velocity  ICC: 0.99; CV: 1.60%; SEM: 0.0018 m/s  43.5 kg  Mean Velocity  ICC: 0.99; CV: 2.03%; SEM: 0.0040 m/s Peak Velocity  ICC: 0.99; CV: 1.57%; SEM: 0.0027 m/s | Was both biological and technological reliability reported? YES / **NO**  Where the statistics/combination of used to validate the device appropriate? **YES** / NO  Did the original study claim the device was reliable? **YES** / NO  Does this device reliably measure what was measured? **YES** / NO |
| Pérez-Castilla, Piepoli [66] | Velowin | Intra-device | S/M Bench press | 45-85% 1RM | Mean Velocity | 14 | 45% 1RM  Mean Velocity  CV: 2.89%; ICC: 0.83  55% 1RM  Mean Velocity  CV: 3.27%; ICC: 0.79  65% 1RM  Mean Velocity  CV: 3.99%; ICC: 0.83  75% 1RM  Mean Velocity  CV: 6.01%; ICC: 0.68  85% 1RM  Mean Velocity  CV: 7.64%; ICC: 0.69 | Was both biological and technological reliability reported? YES / **NO**  Where the statistics/combination of used to validate the device appropriate? YES / **NO**  Did the original study claim the device was reliable? **YES** / NO  Does this device reliably measure what was measured? YES / **NO** |
| Weakley, Chalkley [4] | FLEX (technological and biological error) | Inter-device | F/W Back Squat | 20-100% 1RM | Mean Velocity | 18 | MD: 0.00 m/s;  TE: 0.070 m/s;  CV: 9.82% | Was both biological and technological reliability reported? **YES** / NO  Where the statistics/combination of used to validate the device appropriate? **YES** / NO  Did the original study claim the device was reliable? **YES** / NO  Does this device reliably measure what was measured? **YES** / NO |
|  | FLEX (technological error) | Inter-device | F/W Bench Press | 20-100% 1RM | Mean Velocity | 18 | MD: 0.01 m/s;  TE: 0.064 m/s;  CV: 9.83% | Was both biological and technological reliability reported? **YES** / NO  Where the statistics/combination of used to validate the device appropriate? **YES** / NO  Did the original study claim the device was reliable? **YES** / NO  Does this device reliably measure what was measured? **YES** / NO |
|  | FLEX | Inter-device | Calibrated Rig | 0.53 ± 0.27 m/s 0.99 ± 0.00 m/s  0.84 ± 0.00 m/s  0.78 ± 0.00 m/s  0.71 ± 0.00 m/s 0.60 ± 0.00 m/s  0.54 ± 0.00 m/s 0.47 ± 0.00 m/s  0.38 ± 0.00 m/s  0.28 ± 0.00 m/s 0.17 ± 0.00 m/s  0.09 ± 0.00 m/s | Mean Velocity | 18 | 0.53 ± 0.27 m/s Mean Velocity  MD: 0.00 m/s;  TE: 0.017 m/s;  CV: 3.96%  0.99 ± 0.00 m/s Mean Velocity  MD: 0.01 m/s m/s; TE: 0.041 m/s;  CV: 4.17%  0.84 ± 0.00 m/s Mean Velocity  MD: -0.01 m/s;  TE: 0.06 m/s;  CV: 7.10%  0.78 ± 0.00 m/s Mean Velocity  MD: 0.00 m/s;  TE: 0.019 m/s;  CV: 2.41%  0.71 ± 0.00 m/s Mean Velocity  MD: 0.00 m/s;  TE: 0.016 m/s;  CV: 2.28%  0.60 ± 0.00 m/s Mean Velocity  MD: -0.01 m/s;  TE: 0.02 m/s;  CV: 3.37%  0.54 ± 0.00 m/s Mean Velocity  MD: 0.00 m/s;  TE: 0.016 m/s;  CV: 2.99%  0.47 ± 0.00 m/s Mean Velocity  MD: 0.00 m/s;  TE: 0.013 m/s;  CV: 2.71%  0.38 ± 0.00 m/s Mean Velocity  MD: 0.00 m/s;  TE: 0.013 m/s;  CV: 3.49%  0.28 ± 0.00 m/s Mean Velocity  MD: 0.00 m/s;  TE: 0.016 m/s;  CV: 5.73%  0.17 ± 0.00 m/s Mean Velocity  MD: 0.00 m/s;  TE: 0.006 m/s;  CV: 3.81%  0.09 ± 0.00 m/s Mean Velocity  MD: 0.00 m/s;  TE: 0.002 m/s;  CV: 2.43% | Was both biological and technological reliability reported? **YES** / NO  Where the statistics/combination of used to validate the device appropriate? **YES** / NO  Did the original study claim the device was reliable? **YES** / NO  Does this device reliably measure what was measured? **YES** / NO |
|  | FLEX | Intra-Device | Calibrated Rig | 0.53 ± 0.27 m/s  0.99 ± 0.00 m/s  0.84 ± 0.00 m/s  0.78 ± 0.00 m/s  0.71 ± 0.00 m/s  0.60 ± 0.00 m/s  0.54 ± 0.00 m/s  0.47 ± 0.00 m/s  0.38 ± 0.00 m/s  0.28 ± 0.00 m/s  0.17 ± 0.00 m/s  0.09 ± 0.00 m/s | Mean Velocity | 18 | 0.53 ± 0.27 m/s Mean Velocity  MD: 0.00 m/s;  TE: 0.016 m/s;  CV: 3.77%  0.99 ± 0.00 m/s Mean Velocity  MD: 0.00 m/s;  TE: 0.032 m/s;  CV: 3.28%  0.84 ± 0.00 m/s Mean Velocity  MD: 0.01 m/s;  TE: 0.043 m/s;  CV: 5.11%  0.78 ± 0.00 m/s Mean Velocity  MD: 0.00 m/s;  TE: 0.021 m/s;  CV: 2.71%  0.71 ± 0.00 m/s Mean Velocity  MD: -0.01 m/s;  TE: 0.020 m/s;  CV: 2.81%  0.60 ± 0.00 m/s Mean Velocity  MD: 0.00 m/s;  TE: 0.023 m/s;  CV: 3.82%  0.54 ± 0.00 m/s Mean Velocity  MD: 0.00 m/s;  TE: 0.017 m/s;  CV: 3.19%  0.47 ± 0.00 m/s Mean Velocity  MD: 0.00 m/s;  TE: 0.014 m/s;  CV: 3.01%  0.38 ± 0.00 m/s Mean Velocity  MD: 0.00 m/s;  TE: 0.013 m/s;  CV: 3.42%  0.28 ± 0.00 m/s Mean Velocity  MD: 0.00 m/s;  TE: 0.016 m/s;  CV: 5.93%  0.17 ± 0.00 m/s Mean Velocity  MD: 0.00 m/s;  TE: 0.006 m/s;  CV: 3.64%  0.09 ± 0.00 m/s Mean Velocity  MD: 0.00 m/s;  TE: 0.003 m/s;  CV: 2.89% | Was both biological and technological reliability reported? **YES** / NO  Where the statistics/combination of used to validate the device appropriate? **YES** / NO  Did the original study claim the device was reliable? **YES** / NO  Does this device reliably measure what was measured? **YES** / NO |

*Abbreviations: CMJ: Countermovement Jump, CV: Coefficient of Variation, F/W: Free Weights, ICC: Intraclass Correlation Coefficient, kg: Kilograms, m/s: Meters per Second, MD: Mean Difference, MPV: Mean Propulsive Velocity, PEAK: Peak, RMSE: Root Mean Square Error, S/M: Smith Machine, SEM: Standard Error of Measurement, TE: Typical Error.*

*S17 Table: List of all included & excluded full text studies identified and reviewed from the literature search.*

| **Number** | **Study** | **Status** | **Reason** |
| --- | --- | --- | --- |
|  | Abbott 2020 | Included | Meets inclusion criteria |
|  | Alcazar 2021 | Excluded | Did not report device/velocity related outcomes |
|  | Appleby 2020 | Included | Meets inclusion criteria |
|  | Askow 2018 | Included | Meets inclusion criteria |
|  | Askow 2019 | Excluded | Did not report device/velocity related outcomes |
|  | Balsalobre-Fernández 2016 | Included | Meets inclusion criteria |
|  | Balsalobre-Fernández 2017 | Included | Meets inclusion criteria |
|  | Balsalobre-Fernández 2018 | Included | Meets inclusion criteria |
|  | Balsalobre-Fernández 2019 | Excluded | Did not report device/velocity related outcomes |
|  | Balsalobre-Fernández 2021 | Excluded | Did not report device/velocity related outcomes |
|  | Balsalobre-Fernández 2023 | Included | Meets inclusion criteria |
|  | Banyard 2017 | Included | Meets inclusion criteria |
|  | Banyard 2017 (1) | Excluded | Did not report device/velocity related outcomes |
|  | Banyard 2017 (2) | Excluded | Did not report device/velocity related outcomes |
|  | Banyard 2018 | Excluded | Did not report device/velocity related outcomes |
|  | Banyard 2021 | Excluded | Did not report device/velocity related outcomes |
|  | Bardella 2017 | Included | Meets inclusion criteria |
|  | Bautista 2016 | Excluded | Did not report device/velocity related outcomes |
|  | Beck 2020 | Excluded | Did not report device/velocity related outcomes |
|  | Beckham 2019 | Included | Meets inclusion criteria |
|  | Benavides-Ubric 2020 | Excluded | Did not report device/velocity related outcomes |
|  | Bendic 2021 | Excluded | Did not report device/velocity related outcomes |
|  | Boehringer 2019 | Included | Meets inclusion criteria |
|  | Callaghan 2022 | Included | Meets inclusion criteria |
|  | Carroll 2017 | Excluded | Did not report device/velocity related outcomes |
|  | Cetin 2021 | Included | Meets inclusion criteria |
|  | Chapman 2022 | Excluded | Did not report device/velocity related outcomes |
|  | Chéry 2019 | Included | Meets inclusion criteria |
|  | Comstock 2011 | Excluded | Reported Force/Power related metrics only |
|  | Conceição 2016 | Excluded | Did not report device/velocity related outcomes |
|  | Cormie 2007 | Excluded | Did not report device/velocity related outcomes |
|  | Courel-Ibáñez 2019 | Included | Meets inclusion criteria |
|  | Courel-Ibáñez 2020 | Excluded | Did not report device/velocity related outcomes |
|  | Crewther 2011 | Excluded | Reported Force/Power related metrics only |
|  | Cuadrado-Peñafiel 2014 | Excluded | Did not report device/velocity related outcomes |
|  | Cuevas-Aburto 2020 | Excluded | Did not report device/velocity related outcomes |
|  | De Sá 2019 | Included | Meets inclusion criteria |
|  | Dorrell 2019 | Included | Meets inclusion criteria |
|  | Dragutinovic 2023 | Included | Meets inclusion criteria |
|  | Drinkwater 2007 | Excluded | Reported Force/Power related metrics only |
|  | Elsworthy 2021 | Excluded | Did not report device/velocity related outcomes |
|  | Fernandes 2018 | Included | Meets inclusion criteria |
|  | Ferro 2019 | Included | Meets inclusion criteria |
|  | Feuerbacher 2022 | Included | Meets inclusion criteria |
|  | Freitas-Junior 2021 | Excluded | Did not report device/velocity related outcomes |
|  | Fritschi 2021 | Included | Meets inclusion criteria |
|  | García-Pinillos 2019 | Included | Meets inclusion criteria |
|  | García-Ramos 2018 | Excluded | Did not report device/velocity related outcomes |
|  | García-Ramos 2018 | Included | Meets inclusion criteria |
|  | García-Ramos 2019 | Excluded | Did not report device/velocity related outcomes |
|  | García-Ramos 2020 | Excluded | Did not report device/velocity related outcomes |
|  | Garnacho-Castaño 2015 | Included | Meets inclusion criteria |
|  | Gilic 2022 | Included | Meets inclusion criteria |
|  | Goldsmith 2019 | Included | Meets inclusion criteria |
|  | Gomez-Piriz 2013 | Included | Meets inclusion criteria |
|  | Gonzalez 2019 | Included | Meets inclusion criteria |
|  | Held 2021 | Included | Meets inclusion criteria |
|  | Hojka 2022 | Excluded | Did not report device/velocity related outcomes |
|  | Janicijevic 2021 | Included | Meets inclusion criteria |
|  | Jennings 2005 | Excluded | Reported Force/Power related metrics only |
|  | Jiménez-Olmedo 2021 | Included | Meets inclusion criteria |
|  | Jovanovic 2022 | Included | Meets inclusion criteria |
|  | Kasovic 2021 | Included | Meets inclusion criteria |
|  | Külkamp 2021 | Excluded | Did not report device/velocity related outcomes |
|  | Lake 2017 | Excluded | Did not report device/velocity related outcomes |
|  | Lake 2019 | Included | Meets inclusion criteria |
|  | Lambert 2018 | Excluded | Did not report device/velocity related outcomes |
|  | Laza-Cagigas 2019 | Included | Meets inclusion criteria |
|  | Lopez-Torres 2022 | Included | Meets inclusion criteria |
|  | Lorenzetti 2017 | Included | Meets inclusion criteria |
|  | Lu 2023 | Included | Meets inclusion criteria |
|  | Martínez-Cava 2020 | Included | Meets inclusion criteria |
|  | Martinopoulou 2021 | Included | Meets inclusion criteria |
|  | Mateo 2020 | Included | Meets inclusion criteria |
|  | McGrath 2018 | Included | Meets inclusion criteria |
|  | McMurray 1998 | Excluded | Did not report device/velocity related outcomes |
|  | Menrad 2021 | Included | Meets inclusion criteria |
|  | Merrigan 2021 | Included | Meets inclusion criteria |
|  | Mitter 2021 | Included | Meets inclusion criteria |
|  | Morán-Navarro 2019 | Excluded | Did not report device/velocity related outcomes |
|  | Moreno-Villanueva 2022 | Included | Meets inclusion criteria |
|  | Muniz-Pardos 2020 | Included | Meets inclusion criteria |
|  | Muyor 2018 | Included | Meets inclusion criteria |
|  | Olaya-Cuartero 2022 | Included | Meets inclusion criteria |
|  | Oleksy 2023 | Included | Meets inclusion criteria |
|  | Orange 2019 | Included | Meets inclusion criteria |
|  | Orange 2020 | Included | Meets inclusion criteria |
|  | Orser 2020 | Included | Meets inclusion criteria |
|  | Pareja-Blanco 2020 | Excluded | Did not report device/velocity related outcomes |
|  | Pearson 2020 | Excluded | Did not report device/velocity related outcomes |
|  | Pelaez Barrajon 2020 | Included | Meets inclusion criteria |
|  | Peña García-Orea 2021 | Included | Meets inclusion criteria |
|  | Peña García-Orea 2021 (2) | Included | Meets inclusion criteria |
|  | Pérez-Castilla 2019 | Included | Meets inclusion criteria |
|  | Pérez-Castilla 2020 | Excluded | Did not report device/velocity related outcomes |
|  | Pérez-Castilla 2021 | Excluded | Did not report device/velocity related outcomes |
|  | Pérez-Castilla 2021 | Included | Meets inclusion criteria |
|  | Pérez-Castilla 2021 (2) | Excluded | Did not report device/velocity related outcomes |
|  | Pérez-Castilla 2021 (2) | Included | Meets inclusion criteria |
|  | Pérez-Castilla 2021 (3) | Included | Meets inclusion criteria |
|  | Pino-Ortega 2020 | Included | Meets inclusion criteria |
|  | Pueo 2021 | Included | Meets inclusion criteria |
|  | Qu 2024 | Included | Meets inclusion criteria |
|  | Rodrigo-Carranza 2023 | Excluded | Did not report device/velocity related outcomes |
|  | Rodriguez-Perea 2021 | Included | Meets inclusion criteria |
|  | Ruf 2018 | Excluded | Did not report device/velocity related outcomes |
|  | Sánchez-Pay 2019 | Included | Meets inclusion criteria |
|  | Sañudo 2016 | Included | Meets inclusion criteria |
|  | Sato 2015 | Included | Meets inclusion criteria |
|  | Sindiani 2020 | Excluded | Did not report device/velocity related outcomes |
|  | Stock 2011 | Included | Meets inclusion criteria |
|  | Suchomel 2023 | Included | Meets inclusion criteria |
|  | Thompson 2020 | Included | Meets inclusion criteria |
|  | Thompson 2021 | Excluded | Did not report device/velocity related outcomes |
|  | Tomasevicz 2020 | Included | Meets inclusion criteria |
|  | Tous-Fajardo 2016 | Excluded | Did not report device/velocity related outcomes |
|  | Van Den Tillaar 2019 | Included | Meets inclusion criteria |
|  | Vanzant 2023 | Excluded | Did not report device/velocity related outcomes |
|  | Vanzant 2023 | Excluded | Did not report device/velocity related outcomes |
|  | Weakley 2020 | Included | Meets inclusion criteria |
|  | Weakley 2022 | Included | Meets inclusion criteria |
|  | WeakleyJJS 2017 | Excluded | Did not report device/velocity related outcomes |
